# The whole genome sequence of the Mediterranean fruit fly, *Ceratitis capitata* (Wiedemann), reveals insights into the biology and adaptive evolution of a highly invasive pest species

**DOI:** 10.1186/s13059-016-1049-2

**Published:** 2016-09-22

**Authors:** Alexie Papanicolaou, Marc F. Schetelig, Peter Arensburger, Peter W. Atkinson, Joshua B. Benoit, Kostas Bourtzis, Pedro Castañera, John P. Cavanaugh, Hsu Chao, Christopher Childers, Ingrid Curril, Huyen Dinh, HarshaVardhan Doddapaneni, Amanda Dolan, Shannon Dugan, Markus Friedrich, Giuliano Gasperi, Scott Geib, Georgios Georgakilas, Richard A. Gibbs, Sarah D. Giers, Ludvik M. Gomulski, Miguel González-Guzmán, Ana Guillem-Amat, Yi Han, Artemis G. Hatzigeorgiou, Pedro Hernández-Crespo, Daniel S. T. Hughes, Jeffery W. Jones, Dimitra Karagkouni, Panagiota Koskinioti, Sandra L. Lee, Anna R. Malacrida, Mosè Manni, Kostas Mathiopoulos, Angela Meccariello, Shwetha C. Murali, Terence D. Murphy, Donna M. Muzny, Georg Oberhofer, Félix Ortego, Maria D. Paraskevopoulou, Monica Poelchau, Jiaxin Qu, Martin Reczko, Hugh M. Robertson, Andrew J. Rosendale, Andrew E. Rosselot, Giuseppe Saccone, Marco Salvemini, Grazia Savini, Patrick Schreiner, Francesca Scolari, Paolo Siciliano, Sheina B. Sim, George Tsiamis, Enric Ureña, Ioannis S. Vlachos, John H. Werren, Ernst A. Wimmer, Kim C. Worley, Antigone Zacharopoulou, Stephen Richards, Alfred M. Handler

**Affiliations:** 1 Hawkesbury Institute for the Environment, Western Sydney University, Sydney, Australia; 2 Justus-Liebig-University Giessen, Institute for Insect Biotechnology, 35394 Giessen, Germany; 3 Department of Biological Sciences, Cal Poly Pomona, Pomona, CA 91768 USA; 4 Department of Entomology and Center for Disease Vector Research, University of California Riverside, Riverside, CA 92521 USA; 5 Interdepartmental Graduate Program in Genetics, Genomics & Bioinformatics, University of California Riverside, Riverside, CA 92521 USA; 6 Department of Biological Sciences, University of Cincinnati, Cincinnati, OH 45221 USA; 7 Insect Pest Control Laboratory, Joint FAO/IAEA Programme of Nuclear Techniques in Food and Agriculture, Seibersdorf, Vienna Austria; 8 Department of Environmental and Natural Resources Management, University of Patras, Agrinio, Greece; 9 Department of Environmental Biology, Centro de Investigaciones Biológicas, CSIC, 28040 Madrid, Spain; 10 Human Genome Sequencing Center, Department of Human and Molecular Genetics, Baylor College of Medicine, Houston, TX 77030 USA; 11 National Agricultural Library, USDA, Beltsville, MD 20705 USA; 12 Georg-August-Universität Göttingen, Johann-Friedrich-Blumenbach-Institut für Zoologie und Anthropologie, 37077 Göttingen, Germany; 13 Department of Biology, University of Rochester, Rochester, NY 14627 USA; 14 Department of Biological Sciences, Wayne State University, Detroit, MI 48202 USA; 15 Department of Biology and Biotechnology, University of Pavia, 27100 Pavia, Italy; 16 USDA-ARS, Pacific Basin Agricultural Research Center, Hilo, HI 96720 USA; 17 DIANA-Lab, Department of Electrical & Computer Engineering, University of Thessaly, 382 21 Volos, Greece and Hellenic Pasteur Institute, 11521 Athens, Greece; 18 Department of Entomology, University of Illinois at Urbana-Champaign, Urbana, IL 61801 USA; 19 Department of Biological Sciences, Oakland University, Rochester, MI 48309 USA; 20 Department of Biochemistry and Biotechnology, University of Thessaly, Larissa, Greece; 21 Department of Biology, University of Naples Federico II, 80126 Naples, Italy; 22 National Center for Biotechnology Information, National Library of Medicine, National Institutes of Health, Bethesda, MD 20892 USA; 23 Institute of Molecular Biology and Genetics, Biomedical Sciences Research Centre “Alexander Fleming”, Vari, Greece; 24 Department of Biology, University of Patras, Patras, Greece; 25 USDA-ARS, Center for Medical, Agricultural, and Veterinary Entomology, 1700 S.W. 23rd Drive, Gainesville, FL 32608 USA

**Keywords:** Medfly genome, Tephritid genomics, Insect orthology, Gene family evolution, Chromosomal synteny, Insect invasiveness, Insect adaptation, Medfly integrated pest management (IPM)

## Abstract

**Background:**

The Mediterranean fruit fly (medfly), *Ceratitis capitata*, is a major destructive insect pest due to its broad host range, which includes hundreds of fruits and vegetables. It exhibits a unique ability to invade and adapt to ecological niches throughout tropical and subtropical regions of the world, though medfly infestations have been prevented and controlled by the sterile insect technique (SIT) as part of integrated pest management programs (IPMs). The genetic analysis and manipulation of medfly has been subject to intensive study in an effort to improve SIT efficacy and other aspects of IPM control.

**Results:**

The 479 Mb medfly genome is sequenced from adult flies from lines inbred for 20 generations. A high-quality assembly is achieved having a contig N50 of 45.7 kb and scaffold N50 of 4.06 Mb. In-depth curation of more than 1800 messenger RNAs shows specific gene expansions that can be related to invasiveness and host adaptation, including gene families for chemoreception, toxin and insecticide metabolism, cuticle proteins, opsins, and aquaporins. We identify genes relevant to IPM control, including those required to improve SIT.

**Conclusions:**

The medfly genome sequence provides critical insights into the biology of one of the most serious and widespread agricultural pests. This knowledge should significantly advance the means of controlling the size and invasive potential of medfly populations. Its close relationship to *Drosophila*, and other insect species important to agriculture and human health, will further comparative functional and structural studies of insect genomes that should broaden our understanding of gene family evolution.

**Electronic supplementary material:**

The online version of this article (doi:10.1186/s13059-016-1049-2) contains supplementary material, which is available to authorized users.

## Background

The Mediterranean fruit fly (medfly, *Ceratitis capitata*, Diptera: Tephritidae) is one of the most destructive agricultural pests throughout the world due to its broad host plant range that includes more than 260 different fruits, vegetables, and nuts [1]. Host preferences vary in different regions of the world, which can be associated with its ability to invade and adapt to ecological niches throughout tropical and subtropical regions. While the species originated in sub-Saharan Africa [2, 3], it is currently endemic throughout Africa, the Middle East, European countries adjacent and proximal to the Mediterranean Sea, the Hawaiian Islands, the Caribbean, and Central and South America [4]. Thus the worldwide economic costs due to crop damage, export control due to quarantine restrictions, and control and prevention of medfly infestation reach many US$ billions each year [5] (for an overview of medfly biology, ecology, and invasiveness, see: http://www.cabi.org/isc/datasheet/12367).

Medfly has also been an established lab organism for several decades and is notable as being the closest non-drosophilid relative to *Drosophila* subject to intensive genetic analysis, with broad chromosomal syntenic relationships established. These studies have been largely driven by efforts to use genetic manipulation to improve the sterile insect technique (SIT), which is the primary biologically based method used to control medfly as a component of area-wide multi-tactical integrated pest management (IPM) approaches, which include the use of natural enemies and insecticide/bait formulations. Current SIT applications are based on the use of a classical genetic sexing strain that incorporates female-specific activity of an embryonic temperature-sensitive lethal (*tsl*) mutation. Resultant males are mass-reared in billions per week for sterilization and release in North, Central and South America, Australia, South Africa, and Mediterranean countries including Spain and Israel, to not only control existing populations but to also prevent new invasions [6]. As such, medfly has served as a model system for developing genetic analyses and manipulations that might improve these population control programs that are applicable to a large number of tephritid fruit fly species throughout the world, which range from similarly polyphagous species to ones that are more highly specialized.

Previous studies in medfly mapped ~30 cloned genes and ~40 microsatellite sequences by in situ hybridization to larval salivary gland polytene chromosomes [7, 8]. It was also the first non-drosophilid insect to have its germ-line efficiently transformed by a transposon-based vector system [9], an approach that has since been applied to several orders of non-drosophilid species. This has included functional genomics analysis, new vector systems for transgene stabilization, genomic targeting, and transgenic and *Wolbachia*-infected strains created for potential population control.

To further our understanding of this critical agricultural pest and its genomic organization in comparison to *Drosophila* and other dipteran/insect species, we now present the results of the medfly whole genome sequencing (WGS) project. This is one of 30 arthropod genome sequencing projects that have been initiated as a part of a pilot project for the i5K arthropod project [10] at the Baylor College of Medicine Human Genome Sequencing Center (BCM-HGSC). Notably, the quality of this analysis is unusually strong for an insect genome, comparable to the more compact genome of *Drosophila melanogaster*, where half the 479 Mb medfly genome sequence was assembled in 35 scaffolds larger than 4 Mb (NG50). A thorough automated structural annotation of the genome was conducted, aided by RNA sequencing (RNA-Seq) data, which allowed a curation community of 20 groups to make key sequence assignments related to genome structure, orthology, and genetic regulation, and to manually annotate key gene families related to invasiveness and adaptation, insecticide resistance and detoxification, and aspects of sex-determination, reproduction, and cell death.

This extensive resource is expected to provide a foundation for continued research on fundamental and comparative studies of insect genomes and gene family evolution and the high-quality reference genome assembly should have far-reaching practical applications in pest management research. It will be instrumental to the development of methods for the identification of genome-wide polymorphisms that can be used for population genetic analysis and source determination of medflies identified in ports of entry. Furthermore, its extensive annotated gene set will facilitate identifying the molecular basis of mutations in strains used for SIT (e.g. *tsl* sexing strain) and the identification of novel targets that can be utilized to facilitate higher efficiency and efficacy of IPM programs.

## Results and discussion

### Genome sequence, structure, orthology, and function

#### Whole genome sequencing and assembly

The medfly WGS project reported here is a continuation of an initial project initiated at HGSC that is summarized in Additional file 1: Supplementary material A. Briefly, the initial 454 sequencing project used mixed-sex embryonic DNA from a long-term caged population of the ISPRA strain maintained at the University of Pavia, Italy. This approach yielded relatively low N50 values for both contigs (~3.1 kb) and scaffolds (~29.4 kb) that are presumed to be the result of high levels of polymorphism and repetitive DNA. Thus, the subsequent sequencing attempt reported here used DNA from 1–3 adults that arose from ISPRA lines inbred in single pairs for 12–20 generations. This DNA was used to create 180 bp to 6.4 kb insert-size libraries for Illumina HiSeq2000 sequencing followed by an ALLPATHS-LG assembly (Additional file 2: Table S1; see “Methods”). This yielded a highly improved assembly (GB assembly acc: GCA_000347755.1), though it was determined that 5.7 Mb comprised endosymbiotic bacterial sequences (Enterobacteriaceae and Comamonadaceae; see Additional file 1: Supplementary material C) localized to 18 scaffolds. The majority of the contaminant sequences represent the genome of *Pluralibacter gergoviae* that was recovered in two contigs (see Additional file 1: Supplementary material D and Additional file 2: Tables S2 and S3 for the *P. gergoviae* genome details and annotation). After removal of these bacterial sequences, the new assembly (GB assembly acc: GCA_000347755.2) revealed a final genome size of 479.1 Mb, corresponding to the initial estimated size of 484 Mb that included the bacterial sequences. The 479 Mb assembly size is slightly less than earlier estimates of 540 Mb and 591 Mb, derived from Feulgen stain [11] and qPCR [12] studies, respectively, due to the difficulty of assembling highly repetitive heterochromatic sequences. Re-estimation of the genome size by k-mer analysis, using Jellyfish [13], of the 500 bp insert library sequences obtained a value of 538.9 Mb, in agreement with the Feulgen stain study. Using this estimate, we presume the remaining 11 % of the genome is repetitive heterochromatic regions that could not be assembled with our short read procedure.

The revised assembly yielded 25,233 contigs with an N50 of 45,879 bp assembled into 1806 scaffolds with an N50 of 4.1 Mb (Table 1; see Table 2 for additional assembly features). Using BUSCO [14] on the final genome assembly, it was determined that the assembly correctly identified the full sequence of 2556 genes from a total of 2675 (95 %) found to be conserved across most arthropods. Furthermore, partial coverage of 91 (3.4 %) genes was identified, with only 28 (1.0 %) missing, and an additional 153 (5.7 %) being duplicated. For comparison, the same analysis run on the *D. melanogaster* genome sequence (v. 5.53) identified 98 % of the genes as complete, 0.7 % partial, 0.3 % missing, and 6.5 % duplicated (see Additional file 2: Table S4 for comparisons to *Drosophila* and tephritid species).

**Table 1:** Medfly genome assembly metrics for NCBI Genome assembly accession GCA_000347755.2 that replaces assembly GCA_000347755.1 after removal of bacterial contaminant sequences

Genome assembly	Contigs (n)	25,233
	Contig N50	45,879 bp
	Scaffolds (n)	1806
	Scaffold N50	4,118,346 bp
	Size of final assembly	479,047,742
	Size of final assembly - without gaps	440,703,716 bp
	NCBI Genome Assembly Accession	GCA_000347755.2 http://www.ncbi.nlm.nih.gov/assembly/GCA_000347755.2

**Table 2:** Medfly genome NCBI annotation features for the assembly Ccap_1.0 (see http://www.ncbi.nlm.nih.gov/genome/annotation_euk/Ceratitis_capitata/101/ for details and additional features)

Feature	Count	Mean length (bp)
Genes and pseudogenes	14,652	_
Protein-coding	14,162	_
Non-coding	385	_
Pseudogenes	105	_
Genes with variants	3527	_
Genes	14,547	16,014
All transcripts	24,125	2903
Messenger RNA	23,075	2979
Miscellaneous RNA	238	3506
Transfer RNA	416	74
Long non-coding RNA	396	1074
Single-exon transcripts	2833	1193
CDSs	23,075	2198
Exons	77,742	465
Introns	62,132	4117

#### Curation and gene ontology

Automated annotations were performed using three approaches (see “Methods” and Additional file 1: Supplementary material B): (1) Maker 2.0 [15] at HGSC with the assembled genome and adult male and female RNA-Seq data used to improve gene models; (2) at NCBI using the Gnomon pipeline; and (3) our in-house Just_Annotate_My_genome (JAMg) annotation platform that makes use of RNA-Seq data and de novo predictions (http://jamg.sourceforge.net). Preliminary analysis showed that the NCBI and JAMg annotations broadly agreed and had fewer false positives than Maker 2.0. For manual annotations, curators were provided with the WebApollo manual curation tool [16, 17] hosted by the U.S. Department of Agriculture, National Agricultural Library (USDA-NAL), and data from the JAMg annotation pipeline (and associated tools) with NCBI Ref-Seq derived models. The annotation of 20 key gene sets has resulted, thus far, in curation of 1823 gene (messenger RNA [mRNA]) models, making medfly one of the most highly curated non-drosophilid insects. This has allowed in depth genomic analyses that have revealed divergent genes exhibiting rapid evolutionary rates. These data have been integrated into the dipteran phylogenetic framework by undertaking orthology and synteny comparisons, especially with the closely related species, *D. melanogaster*, and the housefly, *Musca domestica*.

#### Orthology to other arthropod genomes

**Fig. 1:**
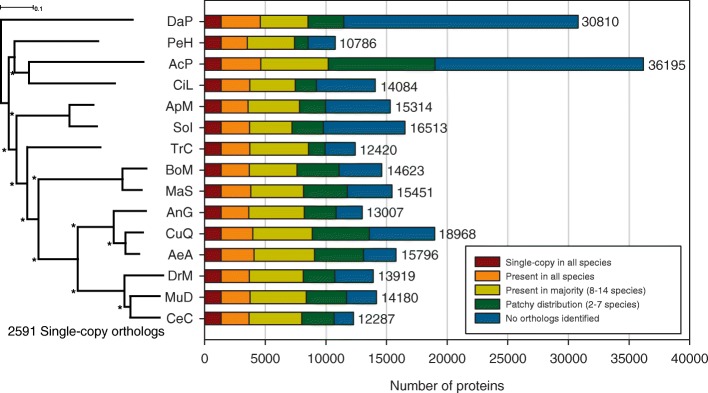
Genome-wide phylogenomics and orthology. The phylogenetic relationship of *C. capitata* and 13 species in Arthropoda was estimated using a maximum likelihood analysis of a concatenation of 2591 single-copy orthologous protein sequences, 1000 bootstrap replicates, and rooted with *D. pulex*. The *scale bar* represents 0.1 amino acid substitution per site and the *asterisks* represent nodes with a bootstrap value of 100. *Horizontal bars* for each species show the absolute number of proteins that are: single-copy orthologs in all species, present in all species (not necessarily in single-copy), present in the majority of species in the analysis, present in a minority of the species (patchy distribution) in the analysis, and unique to the species. Species/strain designations are: *Acyrthosiphon pisum* (AcP), *Aedes aegypti* (strain Liverpool) (AeA), *Anopheles gambiae* (strain PEST) (AnG), *Apis mellifera* (ApM), *Bombyx mori* (BoM), *Ceratitis capitata* (CeC), *Cimex lectularius* (CiL), *Culex quinquefasciatus* (strain Johannesburg) (CuQ), *Daphnia pulex* (DaP), *Drosophila melanogaster* (DrM), *Manduca sexta* (MaS), *Musca domestica* (MuD), *Pediculus humanus* (PeH), *Solenopsis invicta* (SoI), and *Tribolium castaneum* (TrC)

To assess the conservation of protein-coding genes between *C. capitata* and other arthropods, complete proteomes from 14 additional arthropod species were used with *C. capitata* to determine orthology. The analysis of 254,384 protein sequences from 15 species identified 26,212 orthologous groups (defined as containing at least two peptide sequences), placing 202,278 genes into orthologous groups while failing to allocate 52,106 (unique) protein-coding genes into any group. The majority of the unique proteins were identified in *Acyrthosiphon pisum* and *Daphnia pulex*, while interestingly, *C. capitata* had the largest proportion (87 %) of proteins placed into an orthologous group (Fig. 1). This could have been influenced by the larger sampling of dipteran genomes relative to other taxa.

The distribution of proteins among orthologous groups is presented in Additional file 2: Table S5a. When examining conserved proteins, 1345 orthologous groups were found to contain a single-copy protein from all species, while an additional 1879 orthologous groups were found to contain multiple members in one or more species. Moreover, there are 5767 orthologous groups unique to Diptera, 224 of which are present in all dipterans. Within *C. capitata*, 1608 putative peptide sequences could not be placed into any orthologous group, thus identifying them as more recently evolved orphan genes (see Additional file 2: Tables S5b and S5c for orthologous groups and the number of groups for analyzed species, respectively). The distribution of these orphans across the genome is relatively uniform, with no clear pattern or clusters of genes. While these orphan genes are unique to *C. capitata* within this analysis, we would expect this to be less likely if more dipteran species were included within the analysis. Current work is ongoing to provide a more robust orthology of proteins within the family Tephritidae compared to related taxa in Diptera.

#### Chromosomal assignment of scaffolds

**Fig. 2:**
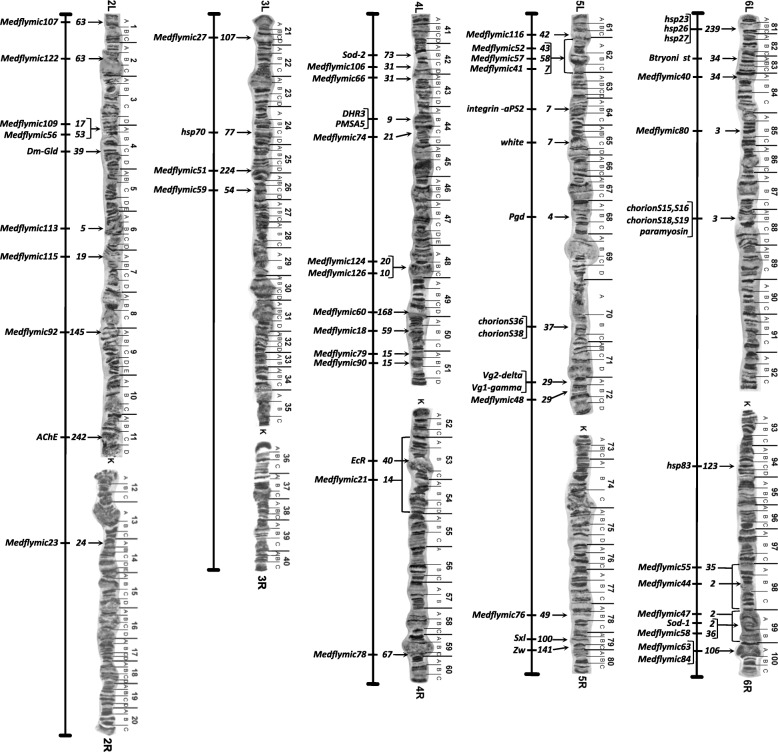
*C. capitata* genome scaffold map based on scaffold linkage of annotated genes and microsatellite (*Medflymic*) sequences previously localized to map banding positions by in situ hybridization to autosomal polytene chromosomes (chromosomes 2 to 6). The larval salivary gland polytene chromosome map [193] presented includes left (*L*) and right (*R*) autosomal chromosome arms linked at a centromeric region (*K*). *Arrows* with adjacent scaffold numbers point to mapped loci positions of designated genes/microsatellites, with *bracketed positions* used for less precise mapping. See Additional file 2: Table S6 for sequence and scaffold accession numbers and sizes, in addition to map positions for sex-linked (chromosome 1; X and Y) genes/sequences mapped to undefined loci on mitotic non-polytenized chromosome spreads [20, 21]

A physical map of the genome that assigns scaffolds to chromosomal loci helps to refine and verify the genomic assembly and allows the analysis of syntenic relationships between species for evolutionary comparisons [18, 19]. It should also aid in the design and analysis of genetic manipulations (e.g. genomic targeting, creation of chromosome inversions and translocations) and especially gene-editing approaches. Similar to *Drosophila*, *C. capitata* is among the few species subject to genomic analysis for which a larval salivary gland polytene chromosome map is available that has been subjected to cytogenetic analysis by in situ hybridization of cloned genes and microsatellite sequences [7, 8]. This has allowed the initiation of a physical genome map by assigning 43 scaffolds, linked to these genes and sequences, to defined loci on five autosomal chromosomes (chromosomes 2 to 6) and a single scaffold to the X (Fig. 2, Additional file 2: Table S6). Four repetitive DNA clones proven to be Y-linked by in situ hybridization to mitotic chromosomes [20] were associated with three scaffolds, though neither the X nor Y have defined polytene mapped loci. Thus, *ceratatoxin* (*ctx*) genes, linked to a single 6.4 Mb scaffold, were also mapped to chromosome 1 (X chromosome) by mitotic chromosome hybridization [21]. Scaffolds with a combined length of 42.6 Mb were linked to chromosome 2, 7.2 Mb to chromosome 3, 60.9 Mb to chromosome 4, 49.1 Mb to chromosome 5, and 45.8 Mb to chromosome 6. The Y-linked sequences could be assigned to more than ten scaffolds, though all were relatively short with the four mapped sequences comprising 0.81 Mb. Thus, more than 212 Mb has been mapped, representing nearly 45 % of the genome, allowing a large proportion of the curated genes to be localized to chromosomal map positions.

This initial step in the development of a physical map already provides significant support for the assembly since localization of 14 (of 45) mapped scaffolds were supported by two or more mapped genes/sequences, and in no case was discontinuity by intervening scaffolds observed. This includes the 15.8 Mb scaffold 3 (NW_004523802.1) on 6L, to which *Medflymic80* at 85B and the *chorion S15-S19* cluster/*paramyosin* genes at 88B are linked. The extrapolated length spanning three map sections (~4.8 Mb/ map section) is also consistent with approximately 90 % of the scaffold size and the high quality of its linkage. The assignment of the 11 Mb scaffold 7 (NW_004524245.1) to 5L-62 to 65C by linkage to three in situ hybridized sequences (*Medflymic41*, *integrin-αPS2*, and *white*) is also consistent with scaffold length and integrity.

**Fig. 3:**
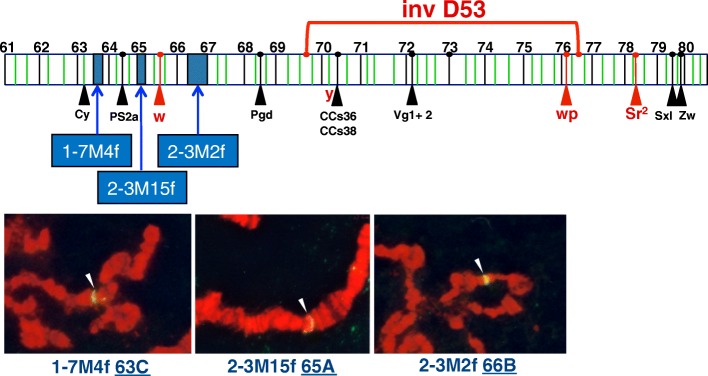
In situ hybridization *mapping* of *piggyBac* transformation vector insertions on chromosome 5 having the D53 inversion used in the VIENNA-8 *tsl* genetic sexing strain. *Top*: a *schematic* of chromosome 5 showing the *piggyBac* vector insertion sites along with other mapped genes and the D53 inversion breakpoints. *Bottom: images* of the yellow fluorescent-tagged hybridization site loci (*arrows*) on third larval instar salivary gland polytene chromosome spreads

Continued scaffold assignment to linkage groups by genome-wide SNP analysis and continued chromosomal hybridizations of annotated genes should aid in the further expansion of the physical map and assembly confirmation. This may also be facilitated by insertion site sequencing of transformant vector integrations that have been localized by in situ hybridization, especially a series of *piggyBac* vector insertions [22] mapped to chromosome 5 of the D53 inversion strain used (to suppress recombination) in the *temperature sensitive lethal* genetic-sexing strain (Fig. 3). Insertion site sequencing for these transformant lines, among others using several transposon-based vectors, should allow further scaffold assignments to these loci.

#### Transposable elements

Mobile or transposable elements (TE) are major constituents of many metazoan genomes that play a significant role in the generation of spontaneous mutations, either by insertional mutagenesis or imprecise excisions that delete critical coding or regulatory sequences [23], making their presence and activity a major contributor to the evolutionary potential of most organisms. The ability of many functional autonomous elements to undergo lateral inter-species transfer, expansion in the new host genome and then accumulation of mutations rendering them inactive, also provides several means of defining phylogenetic relationships.

The presence and activity of TEs (especially Class II elements), in species such as *C. capitata*, has particular significance since medfly was the first non-drosophilid insect to be genetically transformed using a transposon-based vector system [9] and has been the subject of genetic manipulations for improved functional analysis and sexing and sterility strains for improved SIT [24–29]. In this regard, expansion of transformation-based manipulations may depend on the further identification of new TE vector systems, as well as ensuring the stability of previous and new transgenic strains by identifying the genomic presence of potential cross-mobilizing systems [28, 30, 31].

Approximately 18 % of the assembled *C. capitata* genome contains sequences of TE origin (see Additional file 2: Table S7). Of these sequences, 55.9 % are composed of retrotransposons (15.7 % long terminal repeats [LTRs] and 40.2 % non-LTRs) and 44.1 % are DNA transposons. This is higher than the proportion estimated for the euchromatic region of *D. melanogaster* (5.3 %), but not much above the combined estimate as a proportion of the total euchromatic and heterochromatic *D. melanogaster* genome (10–15 %) [32]. Among Class I elements, the *RTE* and *Jockey* non-LTR TE subclasses occupy 5.27 % and 1.47 % of the genome, respectively, while the *Gypsy* LTR TE subclass occupies 1.4 % of the genome. Among DNA transposons, all the major superfamilies are represented with sequences derived from the *Tc1/mariner* superfamily comprising a large majority (82.1 %) of the identified genomic sequences (approximately 6.8 % of the entire genome). This is consistent with the observation in *Drosophila* species in which *Tc1/mariner* elements are a large percentage of DNA transposons, and also with studies from other tephritid species [33, 34].

The large *Tc1/mariner* element representation is of interest since the *Tc1*-like *Minos* element from *D. hydei* [35] was used for the first germ-line transformation of *C. capitata* [9] and the *mariner Mos1* element has a similar potential [11, 36]. BLASTp with the *Minos* transposase amino acid sequence provided no significant alignments, though tBLASTn provided several high similarity alignments including a 1040 nt translated sequence on scaffold 145 (NW_004523255.1) having 55 % identity (1e^–102^).

Medfly has been most commonly transformed with *piggyBac*-based vectors [22] and many of these lines have been stably maintained under artificial marker selection for ten years or more. It is thus unlikely that *piggyBac*-related elements have cross-mobilizing activity, which is supported by relatively low similarities to *piggyBac* at the nucleotide or amino acid sequence level. This is in contrast to nearly identical *piggyBac* elements (>95 % nucleotide coding-region identity) that exist in a broad range of *Bactrocera* species, and throughout the *Bactrocera dorsalis* species complex, though none have been shown to be functional [37, 38]. Indeed, amino acid sequences in the medfly database that are most highly similar to *piggyBac* transposase by BLASTp include the human Pgdb4 isoform X2 (1e^–27^; XP_012161257.1) and *piggyBac*-derived protein 4-like (1e^–25^; XP_004524835.1) sequences, all of which belong to the transposase IS4 family having the DDE_Tnp_1_7 domain. Alignments to the *piggyBac* transposase by tBLASTn, however, yielded multiple sequences with e-values <1e^–10^, with a translated 1281 nucleotide sequence on scaffold 297 (NW_004523799.1) yielding the most significant e-value of 7e^–148^.

The *hAT*-family *Hermes* element from *M. domestica* [39] has also been used to transform medfly [40], and similar to *Minos*, significant similarities in the medfly genome have yet to be found to the complete nucleotide sequence by BLASTn, nor the transposase amino acid sequence by BLASTp. However, tBLASTn provided alignments to two translated nucleotide sequences on scaffold 50 (NW_004524024.1) having significant identities to *Hermes* transposase (43 % identity, 4e^–128^; and 39 % identity, 1e^–116^). Notably, overlapping sequences on scaffold 50 also show relatively high similarity to the *D. melanogaster hobo* (HFL1) *hAT* element that, thus far, has not shown vector function beyond drosophilid species. Partial sequences of several *hobo*-like elements were previously identified in medfly [41, 42].

As expected for members of Class II transposon superfamilies, related elements (or sequences) exist in medfly, though the relatively low levels of identity to transposon vector systems functional in this species, especially in the transposase-coding regions, suggest that significant divergence has occurred during vertical inheritance. If functional, the potential for cross-mobilization would require empirical evidence given the relative stability of transformant lines created with the three vector systems thus far. However, it may be assumed that transposon vectors currently in use will remain stable within the medfly genome with respect to potential re-mobilization by a cross-mobilizing transposase. This is a critical consideration for evaluating strain stability and environmental risks for transgenic strains used in field release programs.

#### microRNAs and microRNA machinery

microRNAs (miRNAs) are small (~22 nts) non-coding RNAs that play a critical role in gene regulation by inducing mRNA degradation and/or translation suppression for genes involved in reproduction, metamorphosis, aging, and social behavior, among numerous other aspects of insect biology [43]. This RNA interference (RNAi) mechanism for regulation of gene expression has facilitated the development of novel strategies for population control. Notably, the role of *Cctra* and *Cctra-2* in medfly sex determination has been clarified by RNAi studies where chromosomal XX females have undergone a sex reversal to phenotypic males [44, 45]. This result has also provided a unique approach to creating male-only populations for SIT release.

To identify precursor and mature miRNAs in *Ceratitis*, a step-wise annotation methodology was utilized. In brief, Hexapoda miRNA precursors and mature sequences were aligned against the assembled genome, while novel miRNA precursors were detected by a modified version of the Maple algorithm [46]. In total, 158 mature miRNAs were identified with high confidence along with 83 precursors. A total of 129 presented significant homology with known Hexapoda miRNAs (Additional file 2: Table S8). The number of identified miRNAs is considerably less than those annotated in *D. melanogaster* (n = 430), but comparable to *Aedes aegypti* (n = 125), and twice the number found in *Musca domestica* (n = 69). A set of 13 putative clusters comprising more than one pre-miRNA have been identified. The majority of clusters (10 out of 13) range in size between 260 and 8514 bp, while the remaining three have sizes of 14,436 bp, 18,467 bp, and 25,941 bp, respectively. Thirty-three of the 83 precursors (~39.7 %) belong to a cluster. The genomic localization of the identified pre-miRNAs (based on the NCBI Gnomon annotation) are putatively detected as six read-through pre-miRNAs (7.2 %; same strand orientation within 4 kb of a start/stop coding region), 30 intronic (36.2 %), three antisense (3.6 %), and 44 intergenic (53 %).

In addition to the mature and precursor miRNA sequences, genes associated with RNAi have been characterized, including *dicer1*, *drosha*, *dgcr8* (*pasha*), and *argonaute-2*, which play key roles in miRNA biogenesis and function [47, 48] (Additional file 2: Table S9). Other important identified miRNA-related genes include *snd1*, *gawky*, *dcp2*, and *ccr4-not. Snd1* is a member of the RISC complex [49, 50], while *gawky* is required for miRNA-mediated gene silencing, promoting mRNA deadenylation and decapping via *ccr4-cnot* and *dcp2* recruitment [51–53]. Moreover, genes encoding *exportin-5* and *ran* proteins, which are responsible for miRNA precursor nuclear export [54] were also characterized.

The identified miRNA-related proteins and the number of predicted miRNAs, exhibit a well-formed layer of post-transcriptional regulation through miRNA-induced translation suppression or mRNA degradation. The high number of conserved miRNAs indicates regulation networks conserved in Hexapoda. No *Sid-1* homologue, which is involved in systemic miRNA, was found in the *C. capitata* genome, similar to other dipteran species. Genes related to siRNA and piRNA biogenesis and function were also identified, including *prkra*, which is required for miRNA/siRNA production by *dicer1*, and *piwi*, *argonaute-3*, *aubergine*, *hen1*, *spindle-e*, *tejas*, *vasa*, *maelstrom*, *deadlock*, *cutoff*, and *tdrkh*, which are part of the piRNA pathway [55]. Interestingly, only *deadlock* and *cutoff* were identified*,* while a *rhino* homologue was not detected in the medfly genome. These three proteins form the proposed *rhino*, *deadlock*, *cutoff* (RDC) complex and loss of *rhino* leads to loss of piRNAs from dual-strand, but not from uni-strand clusters in *Drosophila* [56]. Furthermore, *Yb*-like genes were not identified and it is of interest to determine how the lack of highly homologous *Yb* and *rhino* genes affect piRNA germline transcription or if there are proteins presenting similar functions. Nevertheless, the numerous piRNA-related proteins signify a well-structured pathway.

### Gene families associated with adaptation and invasiveness

Unlike *Drosophila* that inhabits and feeds on rotting and decaying organic matter, and *Musca* that feeds and develops in excreta, carcasses, and other septic matter, medfly is an opportunistic phytophagous species whose survival and dispersion is tightly dependent on its interactions with its different host plants [57]. Adult medfly reproductive behavior is very elaborate and involves the use of sexual pheromones [58, 59] and adults must seek out a rich diet based on carbohydrates and proteins to support a high reproductive rate [60], which involves chemoreception and vision to detect appropriate plant hosts and adaptation to aqueous larval environments. In addition, all invasive insects require adaptation to new and diverse microbial environments requiring immunity mechanisms that defend against multiple pathogens. These are especially important to medfly which oviposits its eggs in microbiologically rich environments. These different resource exploitation and survival strategies are reflected in adaptive differences in the chemoreception, water transport, and visual and immunity system pathways of these species. Indeed, the impressive biological success of medfly is supported by these and additional adaptive traits involving larval to adult life stages in which plasticity for these pathways play a fundamental role [61].

#### Chemoreception

Insect olfaction and gustation is the product of a signal transduction cascade that includes four major gene families [62]. These include the water-soluble odorant-binding proteins (OBPs) that bind to the membrane-bound odorant receptors (ORs) [62, 63], the gustatory taste receptors (GRs) [64], and the ionotropic receptors (IRs) that evolved from the ionotropic glutamate receptor superfamily that respond to amines, acids, and other odorants not perceived by the ORs [65, 66].

**Fig. 4:**
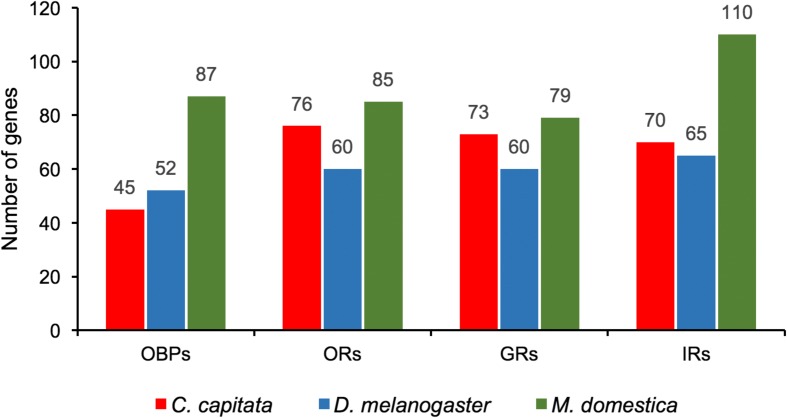
Comparison of gene numbers for odorant-binding proteins (OBPs), odorant receptors (ORs), gustatory receptors (GRs), and ionotrophic receptors (IRs) in *C. capitata*, *D. melanogaster*, and *M. domestica*. Gene numbers provided above each *bar*

The *C. capitata* OBP and chemoreceptor family repertoires (ORs, GRs, and IRs) were compared with those of *D. melanogaster* [63, 65, 67] and the housefly, *M. domestica* [68] (Fig. 4). The total of 46 OBP genes encoding 48 proteins augments the 17 OBP transcripts identified previously from a transcriptome study [58], which is less than the other flies; however, medfly has chemoreceptor repertoires of intermediate size between the lower and higher counts of *D. melanogaster* and *M. domestica*, respectively (see Additional file 3: Figure S1 and Additional file 2: Table S10). The 17 previously identified OBPs were characterized for their transcription activities in different body compartments of each sex and a subset showed transcriptional changes related to maturation, mating, and time of day [59, 69]. Further biochemical assays indicated that CcapObp24 (previously named CcapOBP83a-2) showed high affinity for (E,E)-a-farnesene, one of the five major components of the medfly male pheromone [59]. Detailed examination of the gene family relationships in *C. capitata*, *D. melanogaster*, and *M. domestica* revealed expected patterns of birth-and-death gene family evolution typical of environmentally relevant genes.

**Fig. 5:**
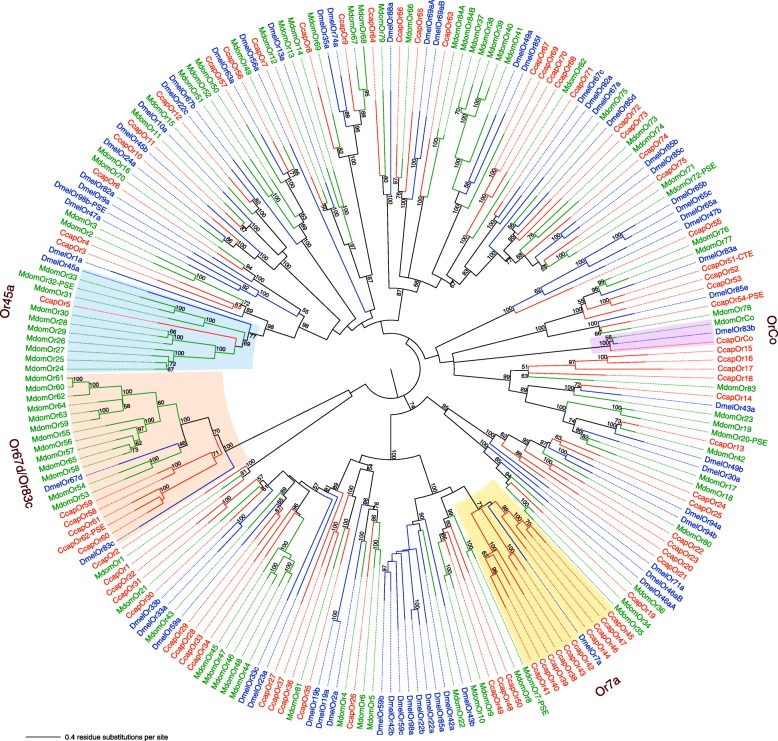
Phylogenetic relationships of OR proteins from *C. capitata*, *D. melanogaster*, and *M. domestica*. The unrooted maximum likelihood (log likelihood = –140908) tree was inferred using the Le and Gascuel model [208] with a discrete Gamma distribution and some invariable sites. Bootstrap values greater than 50 % (1000 replications) are shown. Suffixes after the gene/protein names are: -CTE, C-terminus missing; -PSE, pseudogene

**Fig. 6:**
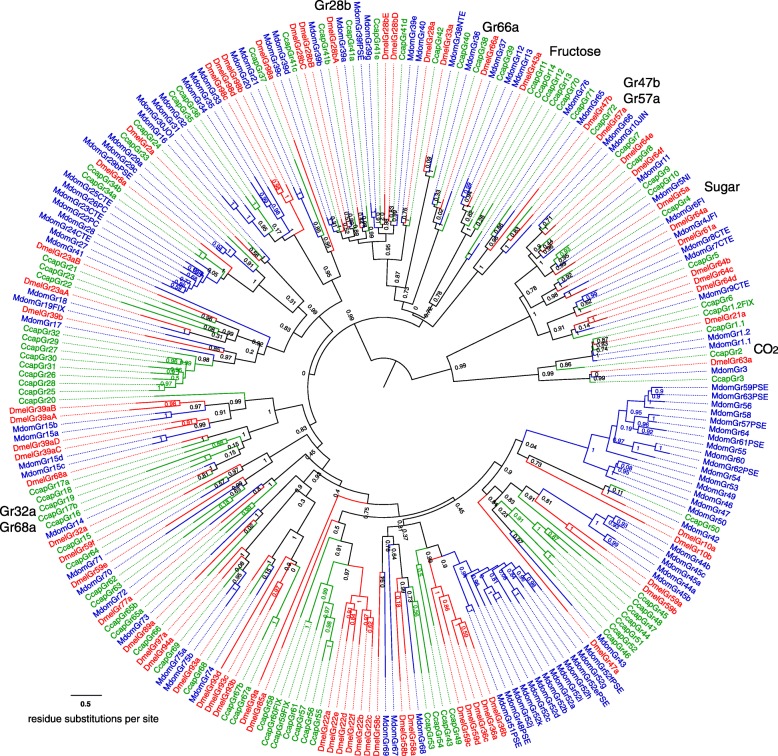
Phylogenetic tree of *C. capitata* GR proteins with those from *D. melanogaster* and *M. domestica*. The maximum likelihood tree was rooted by assigning the carbon dioxide and sugar receptor subfamilies as the outgroup. Clades discussed in the text are indicated on the outer edge

Medfly shares the highly conserved members of these families, such as the Orco protein that functions with each specific OR to make a functional olfactory receptor (Fig. 5; see Additional file 2: Table S11) and the equivalent Ir8a/25a proteins, along with the conserved suites of sugar and carbon dioxide GRs and some bitter taste GRs. In contrast to *Musca*, which exhibited expansions of several lineages of candidate bitter taste receptors in the GR and IR families, as well as expansion of the DmelOr45a lineage implicated in repulsion from aversive chemicals in larvae [70], *Ceratitis* is more similar to *Drosophila* in having few differential expansions of candidate bitter GR and IRs and a single ortholog of DmelOr45a (Fig. 6; see Additional file 2: Tables S12 and S13 and Additional file 3: Figure S2). It also has an expansion of the DmelGr32a and 39b lineages (single orthologs in *Musca*), as well as an ortholog for DmelGr68a (lost from *Musca*), all of which are implicated in pheromone perception [71]. *Ceratitis* also has an expansion (CcapOr58-62) equidistant from the DmelOr67d and DmelOr83c lineages that have, apparently, very different functions. DmelOr67d is involved in the perception of a male-produced pheromone [72] whereas DmelOr83c is specific for farnesol, a component of citrus peel [73], as well as a major component of the medfly pheromone [59]. An extensive expansion of the DmelOr67d lineage is present in *Musca* (MdomOr53-65) suggesting that the housefly, and perhaps medfly, may have more complex pheromone repertoires than *D. melanogaster. Ceratitis* also differs from the other flies in having four genes related to the DmelGr43a lineage, which is a fructose receptor [74] expressed in both mouthparts and the brain, and details of the expression patterns of these four genes may reveal sub-functions for this lineage. Finally, the DmelOr7a lineage that includes a receptor for fruit odors in *Drosophila* [75] shows a medfly-specific expansion to ten genes, while this lineage was lost in *Musca*, which presumably does not require fruit detection. *Ceratitis* has a similar expansion of seven GRs related to the DmelGr22a-f expansion, and two proteins related to DmelGr93b-d, both lineages lost from *Musca*, raising the possibility for the bitter taste receptors involvement in fruit perception.

In summary, *Ceratitis* is more similar to *Drosophila* than *Musca* in its repertoire of chemoreceptors, most likely due to a more similar ecology, despite being more basal phylogenetically [76]. *Ceratitis* is also distinctive in having several separate expansions of receptors implicated in fruit detection and courtship in *Drosophila*. See Additional file 4 for further results, discussion, and protein sequences for the OBP/OR and GR/IR chemoreception gene families.

#### Opsin genes

In addition to odorants, field experiments have shown that the visual system plays a significant role in medfly host fruit detection, for which medflies are equipped with prominent, colorfully patterned compound eyes [77]. This has allowed the development of visual traps for both trapping and monitoring that are effective in both sexes, particularly using coloration in the yellow-orange wavelength range [78, 79]. The attractive effect of specific color hues, however, appears to vary across populations, which reflects genetic plasticity that fine-tunes color preference to the range of available host fruits [80]. In addition, shape recognition represents another vision-guided component in host fruit detection [81] and vision is further assumed to generally play a role in the courtship of tephritid flies [82]. Physiological measurements revealed sensitivity peaks in the UV range (365 nm) and in the yellow-green range (485–500 nm) [77].

These data are compatible with the apparent absence of a member of the blue sensitive opsin subfamily in the *Ceratitis* genome, while the repertoire of long wavelength-sensitive (Rh1, Rh2, and Rh6) and UV-sensitive (Rh3 and Rh4) opsins is conserved between *Ceratitis* and *Drosophila* (Additional file 3: Figure S3). In *Drosophila*, the blue-sensitive *opsin* gene Rh5 is specifically expressed in ommatidia sensitive to shorter wavelengths (“pale” ommatidia), whereas the long wavelength-sensitive opsin Rh6 is present in ommatidia sensitive to green (“yellow” ommatidia) [83]. Given that the blue *opsin* gene subfamily is equally conserved in winged insects [84], as are the UV and green *opsin* gene subfamilies, the absence of a blue opsin from *Ceratitis* indicates unexpected regressive differences in the organization and genetic regulation of differential *opsin* expression compared to *Drosophila*, deserving further exploration.

#### Aquaporin genes

**Fig. 7:**
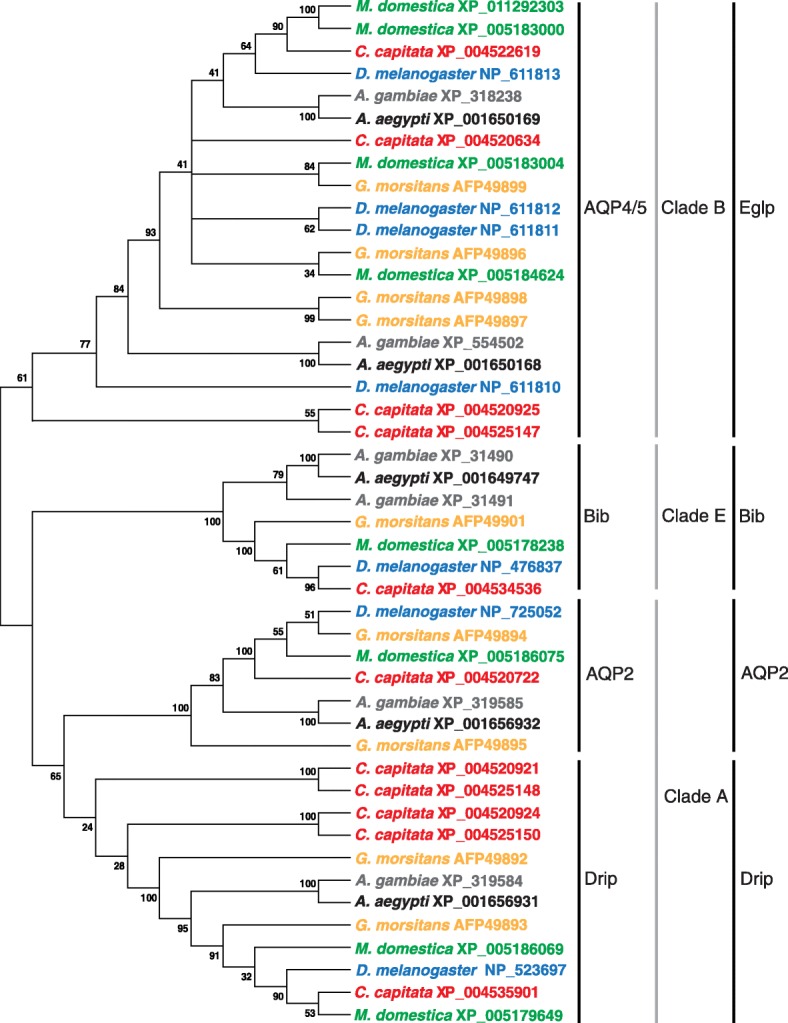
Comparison of predicted AQP amino acid sequences from *C. capitata* and other indicated Diptera. The *neighbor-joining tree* was produced using MEGA6 using Dayhoff Model and pairwise matching; *branch values* indicate support following 3000 bootstraps with values below 20 % omitted. Classification is based upon Finn et al., Benoit et al., and Fabrick et al. [87, 88, 226]. *Drip Drosophila* integral protein, *Prip Pyrocoelia rufa* integral protein, *Eglp* entomoglyceroporin, *AQP* aquaporin. Unorthodox AQPs are not included in this analysis

Twelve putative *aquaporin* (AQP) genes have been identified from the *C. capitata* genome and predicted gene sets (Additional file 2: Table S14), which is the highest number of AQP genes documented for any insect thus far (two more than those discovered in *Glossina morsitans* [85] and four more than those in *D. melanogaster* [86]). This increased gene number is the result of an expanded water transporting Drip/Prip (Clade A) family, which has been demonstrated in other brachycerans such as *Glossina* and *Musca* [85] (Fig. 7). In addition, four putative entomoglyceroporins (Clade B), a recently described Prip-like channel capable of transporting glycerol and urea due to a single mutation of transmembrane domain 5 from a charged histidine to an uncharged amino acid [87], are present in the medfly genome, which is similar to *Drosophila*, *Glossina*, and *Musca* and twice the number found in mosquitoes (Fig. 7). As expected, at least one gene for each of the five previously identified insect AQP groups are present in the *C. capitata* genome [87, 88] (Additional file 2: Table S14). The identification of expanded Drip/Prip genes among most higher flies suggests increased or specialized water transport, but functional studies will be necessary to establish the role of these additional AQPs in relation to brachyceran physiology.

#### Immunity-related gene*s*

The immunity response includes melanization, phagocytosis, encapsulation, clotting, and biosynthesis of antimicrobial peptides and proteins (AMP) by the fat body [89]. Four main signaling pathways are involved in pathogen recognition and defense response: the Toll, immune deficiency (IMD), JAK/STAT, and JNK pathways [90]. The recognition of bacteria in insects is achieved through two families of pattern recognition receptor (PRRs), peptidoglycan recognition proteins (PGRPs), and Gram-negative binding proteins (GNBPs), that bind microbial ligands and activate the immunity pathways [91–93]. Genes representative of all four pathways were identified in the *C. capitata* genome.

The *C. capitata* genome annotation initially identified 413 putative immunity genes by a search of the innate immunity databases for *D. melanogaster* and *An. gambiae* (see http://bordensteinlab.vanderbilt.edu/IIID/test_immunity.php) [94]. Of these, 63 showed direct homology to both *D. melanogaster* and *Anopheles gambiae* putative immunity genes, 77 solely to *An. gambiae* genes, and 122 solely to *D. melanogaster* genes (Additional file 2: Table S15). The remaining 151 genes were identified, via a Hidden Markov model (HMM) analysis, by possessing all the aforementioned components of the insect immune system. The availability of additional insect genomes now allows for more comprehensive comparisons of the various insect immune systems. The total number of medfly immunity genes (n = 413) is somewhat higher than *D. melanogaster* (n = 379), but less so than the housefly (n = 771), which must cope with a pathogen-rich environment [68]. Nevertheless, the enormously diverse host-choice for medfly and its cosmopolitan nature has resulted in a robust immune system providing a defense for the diverse pathogens encountered in the various hosts and environmental conditions.

Specific gene families provide several interesting insights. The antibacterial ceratotoxin peptide family (seven genes), for example, is thus far unique to medfly and several other species in the *Ceratitis* genus, where they exist in the female accessory glands to protect the reproductive tract from bacterial infection during mating. They are also found on the surface of oviposited eggs where they may create a microbiologically controlled environment that favors early larval development [95–98]. These genes are clustered together on the X chromosome and apparently arose as a result of gene duplication [99]. The *spätzle* gene family is also highly expanded in medfly, where it is an immune response effector that activates the Toll signaling pathway [89, 100, 101] induced by Gram-positive bacteria and fungi [102]. Indeed, fungal infections commonly follow fruit punctures after oviposition, which may contribute to the expanded *spätzle* family in medfly. In this respect there is a significant expansion in the Toll receptor family, having 17 genes, relative to nine in *Drosophila* and *M. domestica*. The clip-domain serine protease gene family, required for *spätzle* activation [103, 104], is also expanded in medfly, having 50 genes relative to 45 in *D. melanogaster* and 28 in *M. domestica*. However, *Toll* is also involved in embryonic development [105–107] and, therefore, the observed expansion may involve other systems in addition to immune response.

### Gene families associated with insecticide resistance and detoxification

The emergence of resistance to insecticides is recognized as a major challenge for IPM control of economically important tephritid flies such as *C. capitata* [108]. Thus, a high priority is the identification of genes associated with insecticide resistance, including the three major detoxification enzyme families (cytochrome P450, carboxylesterases, and glutathione S-transferases), known receptors/targets for the main groups of insecticides (cholinesterase, cys-loop ligand-gated ion channel, and voltage-gated sodium channels genes), and cuticle proteins. This analysis also provides significant knowledge relevant to the role of these genes and their gene families in biological processes fundamental to development and behavior.

#### Cytochrome P450 genes

The P450 enzymes, including mixed function oxidases and cytochrome P450 (CYP450) mono-oxygenases, have a highly diverse array of functions including synthesis of hormones critical to insect development and reproduction, as well as chemical metabolism that facilitates host plant adaptation and survival in toxic environments (e.g. insecticide detoxification). This array of functions is achieved, typically, by a large number of related, though structurally independent P450 proteins, whose total number and rate of expansion is influenced by species-specific physiology and environmental challenges.

**Fig. 8:**
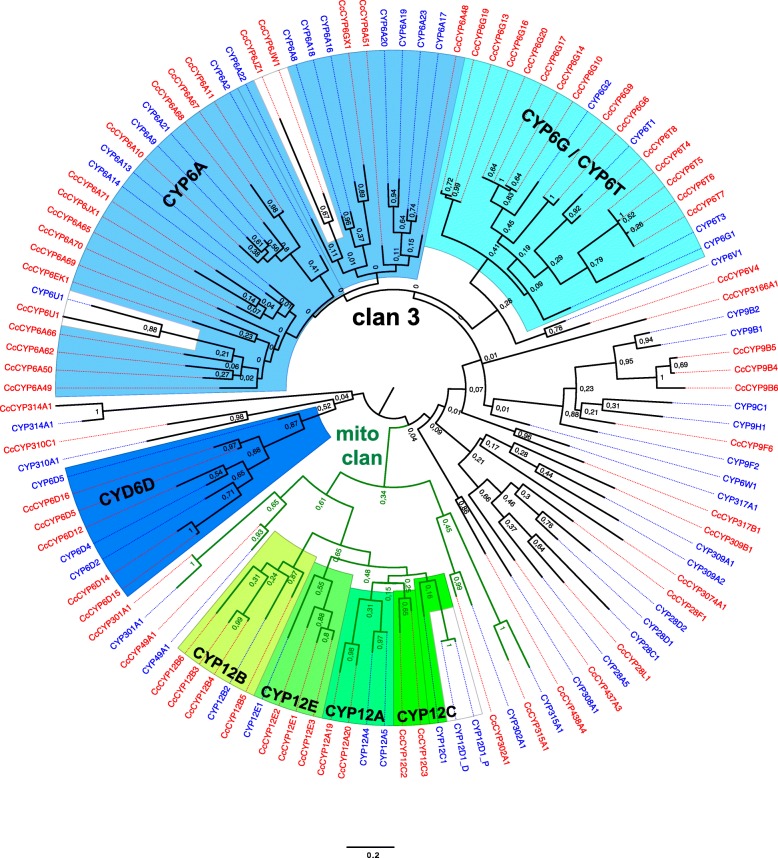
Bootstrap PhyML tree (http://phylogeny.lirmm.fr/) performed with protein sequences of the CYP3 and mitochondrial clans of cytochrome P450 genes found in the genome of *C. capitata* (*red*) and *D. melanogaster* (*blue*). Expanded CYP6 and CYP12 subfamilies are highlighted. Branch length scale indicates average residue substitutions per site

The *C. capitata* cytochrome P450 family is composed of 103 genes and nine pseudogenes (Additional file 2: Table S16) having a greater level of expansion compared to *D. melanogaster* where 88 CYP450 genes and three pseudogenes have been identified (http://flybase.org/). This expansion is mainly found in CYP6 (clan 3) and CYP12 (mitochondrial clan) genes (Fig. 8), but is less expanded than the respective clans found in *M. domestica* [68]. The medfly CYP6 family is composed of 40 genes and four pseudogenes, almost doubling the 23 genes found in *D. melanogaster*, by the notable expansion of subfamilies CYP6A (14 genes), CYP6G (nine genes), and CYP6D (five genes). Interestingly, members of the three subfamilies have been previously associated with insecticide resistance in higher Diptera and clan 3 has been previously characterized as proliferating rapidly by gene cluster duplications [109]. Indeed, a cluster of 18 consecutive CYP genes (13 of which belong to the CYP6A subfamily) is found in the *Ceratitis* genome. Two related, but shorter clusters of two and nine consecutive CYP6 genes are found in the *D. melanogaster* genome separated by 6 Mb. Notably, orthologs for the flanking genes upstream (*mtt,* FBgn0050361) and downstream (*Kank*, FBgn0027596) of the first and the second cluster, respectively, in *D. melanogaster* are found flanking the CYP6 gene cluster in *Ceratitis* (Additional file 3: Figure S4A). The CYP6A51 gene (XP_004534861), whose overexpression had been previously associated with lambda-cyhalothrin resistance in *Ceratitis* [110], is located at one end of this cluster.

The two in tandem CYP6G genes in opposite orientation followed by one CYP6T gene found in *D. melanogaster* is also found in *Ceratitis*, having the same conserved gene order and orientation. However, the tandem array in *Ceratitis* includes three CYP6G genes and one CYP6T, and is repeated at least three times resulting in nine CYP6G genes, four CYP6T genes, and four CYP6G pseudogenes (Additional file 3: Figure S4B). We have also found two more CYP6D genes in *C. capitata* than in *D. melanogaster*.

Finally, the CYP12 family is formed by 11 genes and one pseudogene in *Ceratitis*. Homologs for five of the seven genes of this family identified in *D. melanogaster*, except for Cyp12d1-d and Cyp12d2-p, are found in *Ceratitis*. Interestingly, the additional genes found in *Ceratitis* most likely resulted from duplication events, since they are located in four clusters of two CYP12A (+1 pseudogene), four CYP12B, two CYP12C, and three CYP12E genes (+1 pseudogene). These duplications may be explained by their participation in environmental responses, suggesting that these genes should be of interest to studies on P450-mediated resistance in *Ceratitis*. Indeed, genes of the CYP12 family have been associated with insecticide resistance in both *Musca* and *Drosophila* [109].

Among cytochrome P450 genes there are also components of the ecdysone biosynthesis pathway. 20-hydroxyecdysone (20E) plays a critical role in both early development and female reproduction in most, if not all, insects and, thus, can be targeted by highly specific insect growth regulators (IGR) for population control. Of particular interest are orthologs of the four P450 Halloween genes that act in the final steps of ketodiol conversion to the active hormone [111] (Additional file 2: Table S16). Those found in *Ceratitis* include: *phantom* (*CcCYP306A1*), *disembodied* (*CcCYP302A1*), *shadow* (*CcCYP315A1*), and *shade* (*CcCYP314A1*). By contrast, one of the two genes that may participate as stage-specific components in 20E biosynthesis in *D. melanogaster* (*Cyp307a1*, *spook*, and *Cyp307a2*, *spookier*) is absent in medfly. The gene found in *Ceratitis* contains an intron and has been consequently named *CcCYP307A2*, considering that *D. melanogaster Cyp307a1* is an intronless mRNA-derived paralog of *Cyp307a2* [112] that is only found in the Sophophoran subgenus of *Drosophila* [113].

#### Carboxyl/cholinesterase genes

**Fig. 9:**
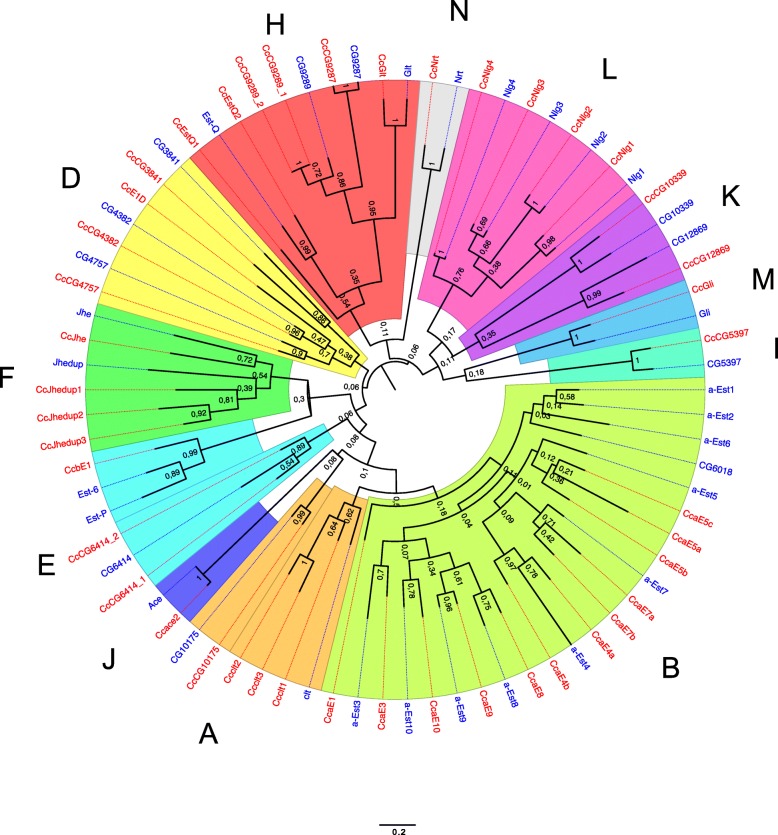
Bootstrap PhyML tree of *C. capitata* (*red*) and *D. melanogaster* (*blue*) esterase protein sequences (http://phylogeny.lirmm.fr/). Clades are indicated by letters, A–N. *Branch length scale* indicates average residue substitutions per site

Insect carboxyl/cholinesterases have been classified within 14 clades (A to N), while two of the clades (C and G) have yet to be found in higher Diptera [114]. A total of 35 carboxyl/cholinesterase genes were identified in *D. melanogaster* while 43 were found in *C. capitata*. Orthologous genes for all members of the acetylcholinesterase and non-catalytic neurodevelopmental clades (I to N) are found in *Ceratitis* with no additional gene duplications or deletions. The differences in the number of carboxyl/cholinesterases genes in *Ceratitis* compared to *D. melanogaster* are found in clades A, D, E, F, and H (Fig. 9; see Additional file 2: Table S17). Three tandem copies of a *cricket-like* gene (clade A, FBgn0000326), putatively influencing male mating behavior in *D. melanogaster* [115], are found in *C. capitata*. The microsomal alpha-esterase gene cluster [116] (Clade B) involved in detoxification is also found in the *C. capitata* genome, and has two extra genes compared to *D. melanogaster*. Here we find two copies of the “*aliesterase*” gene (*alpha-7* or *E3*) that has been associated with organophosphate resistance in *M. domestica* and *Lucilia cuprina* [117]. Interestingly, an unknown mechanism of malathion resistance mediated by an alteration in aliesterase activity is also being studied in *C. capitata* [118]. The cluster of *Est6* and *Est7* genes (Clade E, beta-esterases) conserved in the *Drosophila* species group [116] is not found in the *Ceratitis* genome, which possesses only one of the two genes. Nonetheless, the total number of genes in Clade E is preserved in *C. capitata* due to the tandem duplication of another gene, similar to CG6414 (FBgn0029690). Finally, a single *juvenile hormone esterase* (*JHE*) gene and three tandem copies of the *juvenile hormone esterase duplication* (*JHEdup*) gene, which encode proteins lacking the GQSAG motif found in active JHE [119], are found in *Ceratitis*. A proliferation in the *JHEdup* gene by a duplication event in cactophilic *Drosophila* has been associated with ethanol degradation in rotting fruits [120], although the exact function of these genes remains uncertain. Nevertheless, *JHE* and related genes involved in JH metabolism may also provide important targets for IGRs.

#### Glutathione S-transferase genes

Glutathione S-transferases (GSTs) are a large family of proteins that are involved in metabolic detoxification. We have identified 28 cytosolic GST genes in the *C. capitata* genome and 1 pseudogene (Additional file 2: Table S18), which is fewer than the 36 GST genes found in *D. melanogaster* and equal to the number of genes in *M. domestica*. Phylogeny-based classification has allowed their grouping into six GST subclasses (Additional file 3: Figure S5): seven *Delta* GSTs, 14 *Epsilon* GSTs, one *Omega* GST, three *Theta* GSTs, two *Zeta* GSTs, and one *Sigma* GST. As in *Drosophila*, many of the insect-specific genes of the *Delta* and *Epsilon* subclasses, putatively involved in insect responses to environmental conditions [121, 122], are organized within clusters in the *C. capitata* genome. Genes of these two subclasses have been involved in insect responses to xenobiotics and in insecticide resistance [121, 122], such as OP-resistance in *M. domestica* mediated by gene amplification of *MdGSTD3* and DDT-resistance in *D. melanogaster* associated to over-expression of *DmGSTD1*. However, resistance to insecticides mediated by GSTs have not yet been reported in *Ceratitis*, which may be related to the few cases in which resistance mechanisms have been elucidated for this species when compared to *Musca* and *Drosophila*.

#### Cys-loop ligand-gated ion channel superfamily genes

Members of the cys-loop ligand-gated ion channel (cysLGIC) superfamily [123], including the highly conserved nicotinic acetylcholine receptor (nAChR) subunits and the GABA receptors, GluCls and HisCls, are targets for insecticides. In the medfly genome we find orthologous genes for most of the cysLGIC members described in insects (Additional file 2: Table S19). Interestingly, an additional divergent nAChR subunit gene, coding for an α subunit receptor (nAChRα8) that conserve the two adjacent cysteine residues involved in acetylcholine binding, is identified in *Ceratitis* (Additional file 3: Figure S6). Orthologous genes for this divergent subunit are only found in other tephritid flies such us *Bactrocera cucurbitae* (XP_011189556), *B. dorsalis* (XP_011213957), and *B. oleae* (XP_014090995). This represents a minor expansion of the *C. capitata* nAChR group, which has also been observed in *Apis mellifera* (nine α and two β subunits), *Tribolium castaneum* (11 α and one β subunits), and, more noticeably, in *Nasonia vitripennis* (12 α and four β subunits) [124] (Additional file 2: Table S19). Recently, resistance to spinosad, a major natural control compound for medfly, has been selected in a *C. capitata* laboratory strain [125]. Since spinosyns target nAChR subunits, the functional characterization of this group should be important to elucidating the molecular mechanism involved in this resistance.

#### Voltage-gated sodium channel genes

The voltage-gated sodium channel, the target site for DDT and pyrethroid insecticides, is composed of a pore-forming subunit encoded by a unique gene with extensive alternative splicing and RNA editing, which generate a large collection of sodium channel isoform variants [126] interacting with auxiliary subunits that modulate their function [127]. In *Ceratitis*, orthologs are found for the *D. melanogaster DmNa*_*v*_ gene (formerly *para*) and the auxiliary subunits: *TipE* and four *TipE*-homologous genes (*Teh1*, *Teh2*, *Teh3*, *Teh4*) [127, 128]. The genomic arrangement of the TipE gene family members in *C. capitata* coincides with the one observed in *D. melanogaster* (*TipE*, *Teh2*, *Teh3*, and *Teh4* genes in a cluster separated from *Teh1*), which is believed to be conserved among the Insecta [129]. A well characterized mechanism of resistance to pyrethroids is target site insensitivity mediated by mutations in the voltage-gated sodium channel gene [126], often referred to as knockdown resistance or “kdr resistance.” The information acquired after sequencing the genome would be highly valuable to study this complex target. Resistance to pyrethroids has been reported in both Spanish field populations and a laboratory selected strain of *Ceratitis* [110]; however, this resistance is suspected to be mediated by P450 detoxification as mentioned above.

#### Cuticle protein genes

Using sequence motifs that are characteristic for several families of cuticle proteins [130], 202 genes coding for putative cuticle proteins were identified (Additional file 2: Table S20). These genes were analyzed with CutProtFam-Pred, a cuticular protein family prediction tool [131], and 195 genes could be assigned to one of eight families (CPR, CPAP1, CPAP3, CPF, CPCFC, CPLCA, CPLCG, and TWDL) (Additional file 2: Table S21). The remaining seven proteins lack a defining conserved domain but possess characteristics commonly associated with cuticle proteins, including the repeated low-complexity sequences (AAP[A/V]/GGY). Many of the genes (~77 %) are arranged in clusters of 3–28 genes (Additional file 2: Table S22 and Additional file 3: Figure S7) that are primarily specific to the type of cuticular protein. However, in several cases multiple family types were found in a single cluster. The size and number of clusters was similar to that observed in other species [132, 133] and may be a common feature of cuticle proteins in arthropods. Clustering may be important for the coordinated expression of these genes during critical points in development [134] and it has been suggested that clustering could facilitate the development of insecticide resistance [132].

**Fig. 10:**
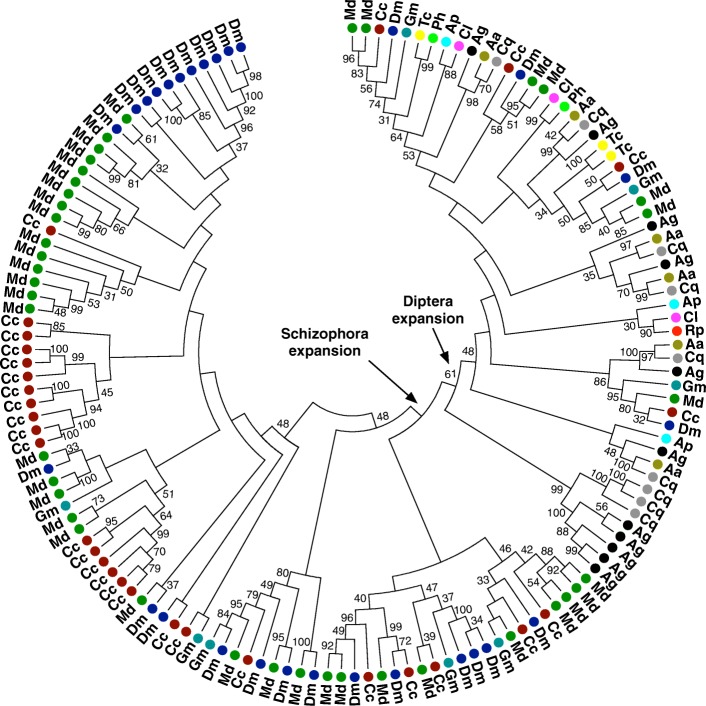
Phylogenetic tree demonstrating relationships of Tweedle proteins from *Ceratitis capitata* (Cc), *Drosophila melanogaster* (Dm), *Musca domestica* (Md), *Anopheles gambiae* (Ag), *Aedes aegypti* (Aa), *Glossina morsitans* (Gm), *Culex quinquefasciatus* (Cq), *Cimex lectularius* (Cl), *Rhodnius prolixus* (Rp), *Tribolium castaneum* (Tc), *Pediculus humanus* (Ph), and *Acyrthosiphon pisum* (Ap). The tree was constructed using the neighbor-joining method in MEGA6; Poisson correction and bootstrap replicates (2000 replicates) were used

Similar to other insects, the CPR family, with the RR-1 (soft cuticle), RR-2 (hard cuticle), and unclassifiable types, constitute the largest group of cuticle protein genes in the *Ceratitis* genome. The 110 CPR genes identified in medfly are comparable to the number in *Tribolium*, but slightly less than the 137 genes found in *D. melanogaster* [134, 135]. The number of genes in the protein families CPAP1, CPAP3, CPF, CPCFC, and CPLCG are similar to the number in other insects [134, 136]; however, *Ceratitis* shows an expansion in the Tweedle (TWDL) and CPLCA families similar to that in *D. melanogaster* and *M. domestica* (Fig. 10; see Additional file 3: Figure S8). Most insects have only 2–5 members of the TWDL family, while dipterans have an expanded family with mosquitoes (Culicidae) possessing 6–12 Tweedle genes, and *Drosophila*, *Musca*, and *Ceratitis* exhibiting a greater expansion of ~30–40 Tweedle genes. Similarly, *Drosophila* and *Ceratitis* also show a greater number of CPLCA genes (13–25 genes) than that found in other species (1–9 genes). The notable exception to the expansion of these gene families within Schizophora is *Glossina* (Fig. 10). The expansion of cuticle protein families likely reflects adaptive evolution [133, 135] and the lack of expansion in *Glossina* may reflect the difference in developmental strategies among these dipterans, with larval development occurring in utero in *Glossina* females. However, the precise role of these protein families, and the functional implications of expanded gene families, requires further study.

### Gene families associated with sex, reproduction, and population control

SIT is the major biologically-based tactic for the control of medfly populations, in addition to several other tephritid pest species. Genomic data related to sex-determination, sex-specific gene expression, reproduction, and programmed cell death have already proven essential to the development of transgenic strains that improve the efficiency of female-lethality for male-only populations, male sterilization, and genetic marking systems for sperm and trapped males. Continued development and improvement of these strains will depend on new strategies that result in: (1) the suppression of testis-specific genes to induce male sterilization (as an alternative to irradiation); (2) the manipulation of sex determination genes in chromosomal females resulting in their development as sterile phenotypic males; (3) the identification of lethal effector genes for tissue and sex-specific conditional lethality; and (4) defining the molecular effects of seminal fluid proteins on female physiology and behavior, which may provide mechanisms that interfere with medfly reproduction.

#### Sex-determination, sex-linked, and sex-specific genes

Sex determination is a fundamental developmental process that regulates male-specific and female-specific sexual differentiation, and thus, various sex-specific aspects of fertility, courtship behavior, and, in some species, dosage compensation. Given the importance of these functions to medfly IPM population control, the sex-determining genetic constituents in this species, and their interactions, have been studied for many years [44, 137]. This has revealed many commonalities with *Drosophila*, including most of the identified sex-determination genes, yet several important distinctions exist [138].

In *Drosophila*, the *Sex-lethal* (*Sxl*) > *transformer/transformer-2* (*tra/tra-2*) *> doublesex/fruitless* (*dsx/fru*) sex-determination gene pathway hierarchy initiates female-specific differentiation when *Sxl* > *tra* transcripts encode functional splicing factors resulting in *dsx*-female expression. In the default state, when *Sxl* > *tra* non-functional products result in *dsx*-male expression, male-specific differentiation ensues. Orthologues to all of these genes were previously identified and tested in *C. capitata*, resulting in a similar hierarchy of activity [44, 45, 139, 140], except that the *Sex-lethal* ortholog (*CcSxl*) does not act as the upstream regulator of *Cctra* in females. Indeed, *Sxl* does not have any apparent sex-determining function in *Ceratitis*, similar to other other non-drosophilid dipterans including *Musca* [141, 142]. Alternatively, it has been shown that *Cctra* activity is required in XX embryos to establish the female developmental pathway, initiating *Cctra* positive auto-regulation by maternal *Cc*TRA, while male differentiation is regulated by a male determining factor (M-factor) that prevents *Cctra* activation [44, 143].

To further elucidate and confirm the relationship between sexual differentiation in *Drosophila* and *C. capitata*, 35 cognates of *Drosophila* genes were identified in the medfly genome that are directly or indirectly involved in sex determination or sexual differentiation (25 genes including *Cctra*, *Ccdsx*, and *CcSxl*), six sex-specifically spliced genes, and four genes having somatic sex-specific functions such as dosage compensation [144, 145] (see Additional file 2: Table S23). A tBLASTn analysis showed sequence conservation for all 35 orthologs, having amino acid sequence identities in the range of 35–98 %, with 20 genes expressed at early embryonic stages [142]. Novel sex-determining genes have evolved from gene duplications in other insects (e.g., *complementary sex-determiner*/*feminizer* in *Apis*, *Nix* in *Aedes*, and *Sxl/sister-of-Sex-lethal* [*ssx*] in *Drosophila*) [140, 146, 147], though paralogs of the *Ceratitis Sxl*, *tra*, and *tra-2* genes have yet to be identified in the medfly genome. While *Nix* is part of the male-determining M-locus in *Aedes*, its relationship to sex-determining genes in medfly (and other species) appears to be limited to the RNA recognition motif (RMM) most often found in *tra-2*. Thus, the molecular nature of the upstream splicing regulator(s) of *Cctra* and the putative Y-linked male determining factor have yet to be clarified, which remains a high priority [137, 142].

Known Y-linked genes are highly limited, and while none are known to encode the M factor, they do provide scaffold identification for sequences that are potentially related. These include four 1–6 kb highly repetitive Y-linked genes (GB acc: AF071418.1, AF154063.1, AF115330.1, and AF116531.1) first identified in a phage library and found to be male-specific and Y-linked by southern blot and mitotic chromosome in situ hybridizations, respectively [20] (see Additional file 2: Table S6). Y-linkage was later confirmed by a Bowtie mapping analysis of >10^8^ male and female genomic reads against the four sequences [137].

For X-linked scaffold identification, the *ceratotoxin* (*ctx*) genes (GB acc: CtxA2, Y15373.1; CtxC1, Y15374.1, and CtxC2-CtxD, Y15375.1), previously mapped by in situ hybridization to the mitotic X chromosome [21], were found on the 6.4 Mb genomic scaffold 23 (NW_004523725), which otherwise provides very low gene content (14 transcribed regions), as expected for a highly heterochromatic chromosome. Within a 0.8 Mb flanking region of the *ctx* family, only the orthologs of *Drosophila carboxylesterase 4* and *tolloid* exist (*Cc*CG4757-like).

The identification of key medfly sex-determining genes has been important to novel sexing strategies for SIT population control, that have incorporated the *Cctra* sex-specific first intron splicing cassette into cell death genes to achieve female-specific lethality [24, 26, 27, 148]. Of particular interest has been the potential use of conditional knock-outs of *Cctra* or *Cctra-2* to transform chromosomal females to phenotypic XX males [44, 45] for high level production of male-only populations for SIT release programs.

#### Seminal fluid protein genes

Insect seminal fluid proteins (SFPs), transferred from males to females during mating along with sperm, are powerful modulators of multiple aspects of female reproductive physiology and behavior, including sperm storage and use, ovulation, oviposition, and receptivity to re-mating [149–153]. These proteins belong to functional classes that are rather conserved across different insect species, and include proteases and protease inhibitors, lipases, sperm-binding proteins, antioxidants, lectins, and prohormones [154, 155]. However, their identification based on sequence similarity searches is challenging, as many have been shown to undergo rapid evolution and gene expansion [156]. This can be explained by the critical roles they play in sperm activation, gamete interaction, and ovulation. Only limited information is currently available relevant to the molecular identity and functional roles of medfly SFPs [157–160]. Recent transcriptomic analyses on the testes and male accessory glands identified transcripts that exhibit mating-induced changes in abundance, most likely related to replenishment of their protein products after multiple matings [159] that are frequent in nature [161–164]. Patterns of sperm use in twice-mated females have also been investigated, revealing that sperm are stored in the female fertilization chamber in a stratified fashion, mostly likely to initially favor the fresher ejaculate from the second male [165]. Studies on the effects of SFPs on female physiology and fertilization dynamics may provide the key to understanding how sperm mobilization within the female reproductive tract is regulated.

A total of 459 genes were annotated in the medfly genome and grouped into 17 functional classes based on the categories defined for *Drosophila* SFPs [166] (see Additional file 2: Table S24 and Additional file 3: Figure S9). The most abundant class corresponds to predicted protease genes, genes involved in lipid metabolism and chitin binding, and sequences with yet unknown function, respectively. Comparison of transcriptional levels between male (ISPRA SRR836190) and female (ISPRA SRR836189) whole body RNA-Seq libraries, as well as reproductive tissue datasets revealed that 37 of all annotated genes are male-biased, with 31 of them being predominantly transcribed in the male reproductive tissues (see Additional file 2: Table S24). These features make them particularly interesting candidates for further functional analysis, although it is noteworthy that SFP-encoding genes do not necessarily display a male-biased expression profile [166, 167].

Proteases also represent a major class among *Drosophila* SFPs, which are thought to be involved in the regulation of female post-mating responses [168], including cleavage of inactive molecules into their active forms [169]. A previous analysis of medfly testes and male accessory glands expressed sequence tags (ESTs) found that one of the proteases, *trypsin alpha-3*, is a mating-responsive gene [159]. This gene, indeed, displays a significant increase in transcript abundance immediately after male copulation, including after successive matings. This may indicate that the depletion of its protein products may trigger transcription to replenish the proteins to be transferred upon mating.

Lipid metabolism genes that may encode SFPs are also abundant in the medfly genome and include sequences that may be active in the breakdown of complex energy sources to be used by stored sperm, or in the remodeling of the sperm phospholipid membrane for capacitation [170]. The high number of genes encoding proteins with predicted chitin-binding activity may be related to antimicrobial roles, and indeed, chitin-binding abilities have been reported for several antifungal peptides [171]. Proteins with such chitin-binding activity have been previously identified not only in *Drosophila* [166], but also in *An. gambiae* [172]. We also identified genes putatively encoding proteolysis regulators, which are a highly represented protein class in the seminal fluid of multiple species [173]. This finding supports the notion that proteolysis-mediated sperm activation might have broad phylogenetic conservation and that proteolytic activity is essential for male reproductive success [174].

The identification of genes encoding proteins involved in odor perception is in agreement with several studies reporting the expression of such genes in the male accessory glands and testes of multiple species [175–182]. The identification of the medfly putative orthologs for Obp56e and Obp56g, which encode proteins found in the *Drosophila* seminal fluid, suggests that medfly OBPs may act as carriers for physiologically active ligands, such as hormones, that are transferred from the male to the female upon mating.

Approximately 10 % of the putative SFP genes annotated could not be associated to a specific functional class. Interestingly, several of these genes (n = 19) displayed a male-biased transcriptional profile and a particularly high abundance in male reproductive tissues. Among them is *CG5867-like*, for which previous ESTs analyses revealed a transcriptional profile possibly related to the replenishment of ejaculate components after mating [159]. Its *Drosophila* ortholog has a hemolymph juvenile hormone-binding domain that has been suggested to be involved in the regulation of hormone levels. Among the genes with unknown functions, of particular interest is the Uncharacterized protein LOC101454281. While lacking significant sequence similarity to known sequences, the presence of multiple glycosylation sites allows us to speculate on its potential mucin nature. In *Drosophila*, mucins have been shown to participate, together with other proteins and lipids, in the formation of mating plugs, often produced within the female reproductive tract during or shortly after mating [149, 183]. Medfly does not produce a plug, but mucins may have a role in protecting sperm and assisting their movement through the female tract, as occurs in mammals [184, 185].

These data lay the foundation for deeper proteomics-based investigations aimed at identifying and quantifying the peptides delivered to the female reproductive tract by medfly males. A deeper understanding of the identity and functional roles of the medfly SFPs will allow their exploitation for manipulating female reproductive physiology, behavior, and fertility. This could possibly lead to the development of novel environmentally acceptable species-specific chemosterilants [186, 187], capable of mimicking the behavior-modulating effects of an SFP by impeding correct sperm storage or interfering with female re-mating.

#### Programmed cell death genes

Pro-apoptotic proteins from the *reaper* (*rpr*), *hid*, *grim* (RHG) gene family, first described for insects in *Drosophila*, are primary regulators of programmed cell death by their negative control of the inhibitor of apoptosis (IAP) proteins, thereby allowing caspase activation resulting in cell death [188]. As such, they have critical roles in development, especially in the larval to adult cellular transitions during metamorphosis, and the removal of cells damaged by environmental stress. In *Drosophila*, their vital roles in development have been demonstrated by lethality resulting from *hid* and *rpr* null mutations or their ectopic misexpression from transgenes [189]. Ectopic misexpression of *hid*, in particular, has been used as an effective lethal effector for uni-sex and female-specific conditional lethality for improved SIT in several species, including medfly where the *Drosophila* cognate was found to be functional [28]. In the caribfly, *Anastrepha suspensa*, the native RHG genes were isolated and functionally validated using cell death assays [190], with the *A. ludens* (mexfly) *Alhid* cognate subsequently used for highly effective conditional lethality in *A. suspensa* [25].

Conservation between the *Drosophila* and medfly apoptotic cognates was first tested by performing the BLASTn algorithm on 95 *Drosophila* genes from four Gene Ontology (GO) groups against the medfly genome: programmed cell death (GO:12501), germ cell programmed cell death (GO:35234), negative regulation of apoptotic process (GO:43066), and apoptotic processes (GO:6915) (Additional file 2: Table S25). Eighty-one genes were highly conserved, of which 57 had e-values less than 1e^–30^, while 14 genes did not show significant similarity to medfly sequences. Notably, the medfly *reaper*, *hid*, and *grim* genes were identified by similarities to multiple regions of the orthologous protein, while *sickle* was only identified by homology of two conserved protein motifs after an additional tBLASTx algorithm search—one being the N-terminal IAP motif and the second being the GH3-binding motif. Both motifs are essential for apoptotic function in *D. melanogaster* and *A. suspensa* [190–192] and their conservation suggests a conserved pathway for the two species.

**Fig. 11:**
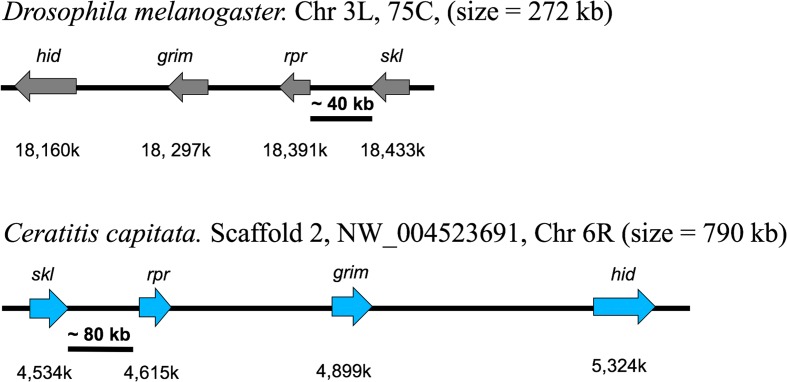
Pro-apoptotic RHG gene group syntenic relationships and relative distances in *D. melanogaster* (*top*) and *C. capitata* (*bottom*). A comparison between the RHG regions, including the *hid*, *grim*, *rpr* (*reaper*), and *skl* (*sickle*) genes, located on chromosome 3L (75C) in *D. melanogaster* and on chromosome 6R (scaffold 2; NW_004523691) in *C. capitata*, reveals a similar organization of genes in the two species. The RHG region in *C. capitata* is 2.9-fold larger relative to *D. melanogaster*, which correlates approximately to the relative total genome size of the two species

Comparisons of the genomic structure of *Drosophila reaper*, *hid*, *grim*, and *sickle* to their medfly orthologs revealed conserved synteny and genomic organization of the respective regions. In *Drosophila*, all four genes are located within a 272 kb region on chromosome 3L, while in *Ceratitis*, the region is located on scaffold 2 (NW.004523691) that maps to chromosome 6R (see Fig. 2, Additional file 2: Table S6). These loci are syntenic based on the polytene map [193], and the region in medfly is nearly three-fold longer, consistent with relative genome sizes (Fig. 11). Nevertheless, the orientation and relative distances among the genes are conserved between the two species.

## Conclusions

Here we report the whole genome sequence of the Mediterranean fruit fly, *C. capitata*, which is one of the most highly invasive and destructive plant pests throughout the world. Of particular interest are the comparative relationships of gene families between this species and two closely related dipterans, *D. melanogaster* and *M. domestica*, having implications for the adaptation and invasiveness of medfly and presenting specific methods and targets for control of its population size. Current research may also utilize the genome assembly as a foundation to characterize population structure of this pest insect throughout tropical regions of the world and the genomic context for genetic sexing strategies for SIT can now be explored utilizing this as a reference genome.

The final 479 Mb genome assembly size varies from our 538.9 Mb k-mer value and an earlier 540 Mb Feulgen stain estimate [11], and we presume this is due to an inability to assemble highly repetitive heterochromatic sequences that account for approximately 11 % of the genome. The high quality of the assembly, however, is reflected in a contig N50 of 45.7 kb and scaffold N50 of 4.06 Mb, and the integrity of 11–15.8 Mb scaffolds consistent with physical mapping. We conclude that this resulted from the use of genomic DNA from highly inbred single and small-pooled flies to minimize polymorphisms. This is in comparison to an initial sequencing attempt using DNA extracted from non-inbred flies from the same laboratory strain, which yielded low-quality assemblies. This protocol has now been established for all species in the i5K pilot project and should serve as a guide for future projects.

The adaptation of medfly to diverse fruits and vegetables, and its successful invasion of associated habitats, may be related to specific gene expansions relative to *Drosophila* and *Musca*. The more similar behavioral ecologies between *Drosophila* and medfly, for instance, seem to be reflected in more similar expansions of the IR and GR taste receptor gene families, as well as receptors for pheromone attractants, compared to the housefly disease vector, *M. domestica*, which is considered to be more closely related to *D. melanogaster* [76]. On the other hand, the larger number of cytochrome P450 genes and the common expansion of CYP6 subfamilies in medfly and *Musca*, relative to *Drosophila*, may reflect their cosmopolitan nature requiring an increased need for adaptation, as well as their more pestiferous behavior. This comparison is consistent with the higher number of immunity system genes relative to *Drosophila*, with the notable expansion of the *Toll* and *spätzle* families, and the unique existence and expansion of *ceratotoxins*, thus far specific to medfly, and both of which may enhance protection of eggs oviposited into diverse microbial-rich environments. Adaptation and invasiveness for medfly may also be reflected in the expansion of the TWDL and CPLCA cuticle protein families, and the highest number of *aquaporin* genes reported thus far in insects.

Another critical role for the medfly genome analysis is defining potential gene targets and genetic reagents relevant to the IPM control of its behavior and population size. The functional characterization of chemoreception molecules, especially those implicated in courtship/pheromone or fruit detection, may permit the development of new synthetic ligands that act as attractants, repellents, or antagonists to disrupt oviposition or mating behaviors. These molecules may also be used as lures for trapping or pest population monitoring. Knowledge of the spectral sensitivity of opsins should also guide the use of optimal trap colors, and while there is conservation for most of the *opsin* genes with *Drosophila*, the absence of the typically conserved blue *opsin* subfamily in medfly is a notable distinction that should be explored. More generally, an improved understanding of the genetic systems involved in fundamental biological processes should aid in the development of specific and more ecologically sound insecticides. The genomic data generated will also facilitate the development of specific tools for the detection of incipient resistance and the implementation of appropriate resistance management strategies.

Genetically modified strains are currently being developed for control of medfly and related tephritid species, and further advances in the effectiveness and safety of these strains will depend on the identification and isolation of stage, tissue, and sex-specific genes and their promoters, and the lethal effectors they will control. The RHG pro-apoptotic cell death gene family was identified in medfly and found to have a genomic organization analogous to *Drosophila*. Functional conservation of these genes is also expected, raising the possibility of using the *reaper* and *grim* genes, along with *hid*, for redundant secondary lethality [194]. SFPs present a large repertoire of potential targets for controlling reproduction, including peptides that might be modified for improved suppression of female multiple matings to enhance SIT.

Central to current gene modification protocols is the use of transposon-mediated germline transformation, for which new and highly efficient TE systems are an ongoing need. Thus, the relatively high euchromatic representation of all the DNA transposon superfamilies is encouraging for the discovery of new elements that may be used as vectors. However, these elements may also have the potential for cross-mobilizing related elements, some of which are in current use as transformation vectors. The full genome sequence now allows the identification of potential mobilizing and cross-mobilizing systems, which is critical to evaluating potential instability of transposon vectors for genome modification and mitigation of associated risks. Therefore, the lack of apparent mobilizing systems in the medfly genome for current vectors diminishes the potential for transgene instability and possible lateral inter-species transfer. This alleviates a serious concern for an environmental risk related to the release of genetically modified medflies.

The relatively high quality of this genome sequence is also a prerequisite for highly specific gene editing and the identification of specific genomic sites that can be used for insertional targeting that avoids deleterious genomic effects. Routine use of such target sites could be made most efficacious by initially introducing small recombination sites by gene editing that could be subsequently used for repetitive recombinase-mediated megabase transgene insertions and deletions [29, 30]. This would allow for genomic modifications that avoid position effects on transgene expression and insertional mutations that debilitate the host strain, resulting in enhanced functional studies and modified strains for the most effective and ecologically sound means of population control.

## Methods

### Genome sequencing and assembly

The second attempt to improve genome sequence quality was initiated by consecutive single pair sib inbreeding of the *C. capitata* ISPRA strain for 20 generations, to achieve a high level of genome homozygosity thereby reducing sequence polymorphisms that were the likely cause of previous weak assemblies (Additional file 1: Supplementary material A). The more highly successful genome assembly reported here was thus, importantly, dependent on this level of inbreeding, and it was used as a strong recommendation to perform inbreeding for 10–12 generations for all i5K projects [10], or as many generations as possible. The assembly reported here used an enhanced Illumina-ALLPATHS-LG sequencing and assembly strategy that is being used for other species in the BCM-HGSC i5K pilot project, which enabled multiple species to be approached efficiently in parallel.

Details for the insert library preparation and sequencing are available in Additional file 1: Supplementary material B. Briefly, the sequencing read data were assembled using ALLPATHS-LG (v35218) [195] and further scaffolded and gap-filled using the in-house tools Atlas-Link (v.1.0) and Atlas gap-fill (v.2.2, https://www.hgsc.bcm.edu/software/). Alignments were conducted as part of the ALLPATHS-LG assembly process and the true insert size of mate pair libraries was estimated, with the 8 kb library adjusted to 6.4 kb. This assembly was 484.7 Mb in total length with a contig N50 of 45,711 bp and a scaffold N50 of 4.06 Mb; however, initial annotations revealed the presence of significant bacterial sequence that was identified as 5.7 Mb of endosymbiotic bacterial sequences localized to 18 scaffolds (see Additional file 1: Supplementary material C for details). Analysis of the metagenomics content was conducted using Blast [196] and Kraken [197] and is described in detail within Additional file 1: Supplementary material C. Removal of the contaminant sequences (with the GenBank assembly accession iterated to GCA_000347755.2) revealed a final genome size of 479.1 Mb with a contig N50 of 45,879 bp and a scaffold N50 of 4.12 Mb, which has been deposited in the NCBI: BioProject PRJNA168120 (see Table 1 and Additional file 2: Table S1).

### Genome annotation and downstream informatics analysis

To facilitate annotation, RNA-Seq data were generated from three samples, including mixed-sex embryos and whole body male and female adults, using RNA extracted with Trizol reagent (Life Technologies) followed by DNase treatment (DNAfree, Ambion). A total of 5.3 Gb sequence data were produced for the embryo, 4.9 Gb for the female, and 7.8 Gb for the male samples (see Additional file 2: Table S1 and NCBI BioSample: SAMN02055687 - SAMN02055689). These data were aggregated with data contributed by the community (see Additional file 2: Table S26; complete dataset including experimental procedures available at GEO, accession number GSE80605). The assembly was annotated by the consortium using three distinct approaches: (1) Maker 2.0 [15] at HGSC with the assembled genome and adult male and female RNA-Seq data used to improve gene models; (2) at NCBI using the Gnomon pipeline; and (3) our in-house JAMg, all publicly available with details for Maker 2.0 and Gnomon described in Additional file 1: Supplementary material B. Briefly, JAMg was used to produce automated annotations which made use of GSNAP, Trinity RNA-Seq de novo, Trinity RNA-Seq genome-guided [198], PASA [199], Augustus [200], and other tools before deriving a consensus gene set using EvidenceModeler [201]. Then PASA was used again to annotate UTRs and generate alternative splicing isoforms. Annotations from the three platforms were provided to the curation community using the WebApollo JBrowse tool as hosted by the USDA, National Agricultural Library (https://apollo.nal.usda.gov/cercap/sequences).

### Orthology assignment

The final annotation set for *C. capitata* (CeC) was compared to other arthropod genomes to characterize orthology. First, the following annotation sets were extracted from genomic databases for the arthropod species: *Acyrthosiphon pisum* (AcP), aphidbase.com ACYPI OGS 2.1B; *Aedes aegypti* (strain Liverpool) (AeA), vectorbase.org, OGS AaegL3.3; *Anopheles gambiae* (strain PEST) (AnG), vectorbase.org, OGS AgamP4; *Apis mellifera* (ApM), hymenopteragenome.org/beebase, OGS Amel_4.5; *Bombyx mori* (BoM), Ensembl build 29, GCA_000151625.1.29; *Cimex lectularius* (CiL), i5k.nal.usda.gov, OGS v1.2; *Culex quinquefasciatus* (strain Johannesburg) (CuQ), vectorbase.org, OGS CpipJ2.2; *Daphnia pulex* (DaP), genome.jgi.doe.gov, OGS FrozenGeneCatalog20110204; *Drosophila melanogaster* (DrM), flybase.org, OGS r6.08; *Manduca sexta* (MaS), i5k.nal.usda.gov, OGS v2.0; *Musca domestica* (MuD), vectorbase.org, OGS MdomA1.1; *Pediculus humanus* (PeH) vectorbase.org, OGS PhumU2.1; *Solenopsis invicta* (SoI) hymenopteragenome.org/ant_genomes, OGS 2.2.3 w/ HGD-IDs; and *Tribolium castaneum* (TrC), NCBI WGS, OGS GCF_000002335.2_Tcas_3.0. For each gene set, the longest peptide sequence was selected for each gene model from all available isoforms, removing low-quality and short sequences. The final counts of proteins for each species is indicated in Fig. 1. The OrthoMCL pipeline (v2.0.9) was followed to define orthologous groups of proteins between these peptide sets [202]. Briefly, after formatting the peptide sequence file for each species, an all-by-all BLASTp search was performed between all proteins from all species. The resulting blast hits were loaded into the OrthoMCL schema within a MySQL database. Ortholog groups were calculated using the scripts provided with OrthoMCL and the MCL clustering algorithm [203]. This results in sets of orthologs, co-orthologs, and in-paralogs defined between all peptides from all species. From this, counts of shared proteins between species were calculated and summarized in Fig. 1. To place the species within a phylogenetic context, single copy orthologs were identified between all species using BUSCO [14, 204]. A total of 2591 single copy orthologs were used to generate a multigene alignment. Peptide sequences from each species for each orthologous group were aligned independently using MUSCLE [205], trimmed using trimAl with parameters “-w 3 -gt 0.95 -st 0.01” [206], and trimmed sequences were concatenated using ElConcatenero (https://github.com/ODiogoSilva/ElConcatenero). Phylogenetic analysis in RAxML was performed using the PROTGAMMA amino acid substitution model and 1000 bootstrap replicates [207]. This substitution model was selected due to its use of empirical base frequencies and the LG substitution model which is a general amino-acid replacement matrix that was demonstrated to produce a tree topology with a higher likelihood than trees produced using an alternative amino acid substitution model such as WAG or JTT [208]. This tree was rooted with *D. pulex* and was visualized using Dendroscope 3.2.10 [209]. The resulting tree was used to order the species in Fig. 1.

### Manual annotation of specific genome characters and gene families

#### Transposable elements

##### DNA transposons

The assembled *C. capitata* genome was analyzed for potential DNA transposon sequences using the program RepeatModeler and a custom library of DNA transposon sequences from available publications and databases (http://www.repeatmasker.org). The output of the RepeatModeler program was aligned to custom protein database of DNA transposon sequences using the fasty36 program [210] with e-value cutoff of 0.5 to further identify potential genuine TE sequences [211]. Duplicate entries were identified using the program BLASTclust [212].

##### LTR elements

LTR annotation was both structurally and homology-based. First, a structurally based LTR search was performed by running the LTR_STRUC program on genomic scaffolds [213]. Second, a homology-based annotation of the repeat families, which were generated by running RepeatModeler on the scaffolds was compared to a database of known, RepBase Drosophila LTRs using tblastx searches via the CENSOR program [214].

##### Non-LTR retrotransposons

A modified version of the homology-based TESeeker [215] was used to identify non-LTR retrotransposons. TESeeker was run with representative TEs included with it, as well as those identified by RepeatModeler. TEs were classified with an in-house classifier that uses reverse transcriptase conserved domains to classify based on the open reading frame of the TEs. tBLASTn searches were then performed using the classified TEs to help reconstruct a full-length element.

#### microRNAs

Mature miRNA sequences along with miRNA precursors were retrieved from miRBase 21 [216]. Precursors were subsequently aligned against the assembled genome using BLASTn, with positive hits presenting e-values <1e^–10^. Mature miRNA sequences from Hexapoda were aligned against the assembly using Bowtie v1 [217], allowing up to one mismatch. The aligned mature miRNAs and precursors were selected and annotated, and for homologous loci, the secondary structure of the region was further investigated using RNAfold [218]. A modified version of Maple module from the ShortStack [46] algorithm was utilized in order to identify characteristic pre-miRNA features such as complementarity at 5’ and 3’, a 3’ overhang, 3’ and 5’ bulges. Sequences having the most stable structure were selected for each locus.

To identify homologous transcripts in Hexapoda, mature miRNAs presenting identity in seed region and total sequence, allowing 1–2 mismatches in 3’ or 5’ (excepting seed region) were collapsed into clusters. Clusters with >3 Hexapoda members were marked as presenting high homology in the subphylum. Only miRNAs marked as having experimental support in miRBase were included in order to avoid sequences identified solely by homology studies, and to enhance the robustness of the analysis.

#### Chemoreceptor genes

For the annotation of the OBP and OR gene families, tBLASTn searches were performed on the genomic scaffold sequences, using *D. melanogaster* and *M. domestica* OBPs and ORs as queries. The putative proteins encoded by the identified gene models that produced hits (<1e^–10^) were used to query, using BLASTx, local protein databases of the *D. melanogaster* and *M. domestica* OBPs and ORs. The gene models were modified, where necessary, in WebApollo. The medfly genes were named using a numerical system, with genes on the same scaffold numbered sequentially. However, for the OBPs, the sequential numbering system was modified to permit sequential naming of the different OBP subfamilies. The medfly genes and their encoded proteins are detailed in Additional file 2: Tables S10 and S11 and putative protein sequences are provided in Additional file 4. Pseudogenes (suffix PSE) were translated as well as possible so that they could be aligned with the other proteins for the phylogenetic analysis. In the case of the OBP protein sequences, the signal peptide sequences were excluded before alignment and the phylogenetic analyses. For each family the amino acid sequences were aligned using MAFFT v7 [219] with the E-INS-i strategy, BLOSUM62 matrix, 1000 maxiterate and offset 0. The most appropriate model of molecular evolution for each dataset was determined using MEGA 6.0.6 [220]. Phylogenetic relationships were estimated using maximum likelihood with 1000 bootstrap replications using MEGA 6.06 retaining positions present in at least 75 % of the sequences. The resulting mid-point rooted tree was drawn using FigTree v1.4 (http://tree.bio.ed.ac.uk/software/figtree/) and iDraw (www.indeeo.com).

The GR and IR gene families were manually annotated and analyzed with the aid of maximum likelihood phylogenetic trees. BLASTp searches were performed on the JAMg Consensus Gene Set v1, as well as high-confidence and low-confidence protein sets from NCBI. tBLASTn searches were also performed using all *D. melanogaster* and *M. domestica* relatives as queries. If the gene models appeared to be intact or were easily repaired in the WebApollo tool employed for manual gene annotation, they were manipulated and named therein, but more difficult gene models were manually assembled in TextWrangler before being modified in WebApollo. All of the *Ceratitis* genes and encoded proteins are detailed in Additional file 2: Tables S12 and S13, with protein sequences provided in Additional file 4.

Only a few difficulties with the genome assembly were encountered in these two gene families, such as truncation of exons in gaps between contigs within scaffolds or off ends of scaffolds (suffix NTE in the figures, tables, and proteins). In most cases these gene models were corrected using raw reads (suffix FIX). One IR model was designed that spans scaffolds, with no support other than the agreement of the available exons on both scaffolds, and their appropriate relatedness to similar genes (suffix JOI). Pseudogenes were translated to an encoded protein as a best match for alignment with intact proteins for phylogenetic analysis (suffix PSE). Pseudogene translations included in the analysis were limited to those having at least half the average length of related proteins and several shorter fragments were not included. Protein families were aligned in CLUSTALX v2.0 [221] using default settings with the relevant families of *D. melanogaster*. Problematic gene models and pseudogenes were refined in light of these alignments. Less obvious pseudogenes (e.g. those with small in-frame deletions or insertions, crucial amino acids changes, or promoter defects) would not be recognized, so the provided functional protein totals might be high.

For phylogenetic analysis, the alignments were trimmed using TRIMAL v1.4 [206] retaining only positions present in more than 80 % of the sequences. Phylogenetic analysis was performed using maximum likelihood methods in PHYML v3.0 [222] using default settings, and trees were prepared in FIGTREE v1.4 (http://tree.bio.ed.ac.uk/software/figtree/) and Adobe Illustrator.

#### Immunity-related genes

*D. melanogaster* and *An. gambiae* immune-related genes were retrieved from the Insect Innate Immunity Database (see: http://bordensteinlab.vanderbilt.edu/IIID/test_immunity.php) [94] and aligned against *C. capitata* gene models using Blastp [196]. Medfly genes that showed the best Blast hit against *D. melanogaster* or *An. gambiae* were assumed to be putatively involved in the medfly immune system and were annotated manually.

An additional HMM analysis was performed in order to enrich the medfly immunity gene repertoire. Thirty-four curated multiple sequence alignments of potential immune-related genes from *D. melanogaster*, *An. gambiae*, *Ae. Aegypti*, and *C. quinquefasciatus* were retrieved from ImmunoDB [223] and HMMs were built using HMMER software, version 3.1b1 [224]. These HMMs were used to calculate the likelihood of having any of the 34 domains for each of the *C. capitata* predicted proteins, with calls having an e-value <10^–2^ annotated manually.

#### Seminal fluid proteins genes

To annotate putative medfly SFP genes, we queried (tBLASTn, e-value <10^–10^) the genome scaffolds using the amino acid sequences of the 146 characterized *D. melanogaster* SFPs [166, 225]. Sixty-four of the *Drosophila* SFPs gave no significant hits to the medfly genome, whereas the remaining sequences resulted in multiple hits. The predicted amino acid sequences of the identified medfly gene models were considered for annotation if they gave significant reciprocal BLASTp (e-value <10^–10^) hits in the NCBI nr database to sequences belonging to known SFP functional classes. In addition, we queried (tBLASTx, e-value <10^–10^) the genome scaffolds using the ESTs previously derived from medfly male testes and male accessory glands [159]. The predicted amino acid sequences of the identified gene models were considered for annotation if they gave significant reciprocal BLASTp (e-value <10^–10^) hits in the NCBI nr database to sequences belonging to known SFP functional classes.

## Additional files

Additional file 1: Supplementary material A. *C. capitata* genome sequencing approaches, B. Automated annotations, C. Detection of bacterial sequence contamination and D. Screening the *C. capitata* genome sequence for potential horizontal gene transfer events. (DOCX 601 kb)

Additional file 2: Supplementary Tables S1–S26. **Table S1** *C. capitata* genome and RNA-seq source material and sequencing runs. **Table S2** *Pluralibacter gergoviae* genome metrics. **Table S3** *P. gergoviae* genes associated with general functional categories. **Table S4** BUSCO genome assembly comparisons between *C. capitata*, *D. melanogaster*, and *Bactrocera* species. **Table S5a** Orthology tables - Copy numbers. **Table S5b** Orthology tables - Orthologous groups. **Table S5c** Orthology tables - Counts by species. **Table S6** Chromosomal positions for mapped scaffolds. **Table S7** *C. capitata* transposable element sequences. **Table S8** *C. capitata* microRNA sequences. **Table S9** microRNA/siRNA/piRNA machinery in *C capitata.* **Table S10** *C. capitata* odorant-binding protein (OBP) genes. **Table S11** *C. capitata* odorant receptor (OR) genes. **Table S12** *C. capitata* gustatory receptor (GR) gene assignments. **Table S13** *C. capitata* ionotrophic receptor (IR) gene assignments. **Table S14** *C. capitata* aquaporin genes. **Table S15** Immunity-related gene comparisons for *C. capitata*, *D. melanogaster*, and *M. domestica.* **Table S16** P450 genes in the *C. capitata* genome*.* **Table S17** Carboxylesterase genes in the *C. capitata* genome. **Table S18** Glutathione S-transferase (GST) genes in the *C. capitata* genome. **Table S19** CysLGIC superfamily genes in *C. capitata* and other insect genomes. **Table S20** *C. capitata* cuticle protein genes. **Table S21** Putative cuticle proteins per family in the *C. capitata* genome. **Table S22** Cuticle protein gene clusters in the *C. capitata* genome. **Table S23** *C. capitata* sex-determination gene orthologs. **Table S24** Putative seminal fluid protein (SFP) genes in the *C. capitata* genome. **Table S25** *C. capitata* genes related to the apoptotic pathway of *D. melanogaster*. **Table S26** Community RNA-Seq data for the genome assembly (XLSX 6240 kb)

Additional file 3: Supplementary figures S1–S9. **Figure S1** Odorant-binding protein (OBP) genes phylogenetic tree. **Figure S2** Ionotrophic receptor (IR) genes phylogenetic tree. **Figure S3** Opsin genes phylogenetic tree. **Figure S4** CYP gene clusters A and B. **Figure S5** Glutathione S-transferase genes phylogenetic tree. **Figure S6** cysLGIC genes superfamily phylogenetic tree. **Figure S7** Cuticle protein gene clusters. **Figure S8** CPLCA cuticle protein genes phylogenetic tree. **Figure S9** Seminal fluid protein functional classes. (PDF 9425 kb)

Additional file 4: Supplementary material: *C. capitata* chemoreceptor genes. (DOCX 194 kb)

## Abbreviations

20E: 20-hydroxyecdysone
AMP: Antimicrobial peptides
*ctx*: *Ceratotoxin*
CYP450: Cytochrome P450
cysLGIC: Cys-loop ligand-gated ion channel
*dsx*: *Doublesex*
EST: Expressed sequence tag
*fru*: *Fruitless*
GNBP: Gram-negative binding protein
GO: Gene Ontology
GR: Gustatory taste receptor
GST: Glutathione S-transferase
HMM: Hidden Markov Model
IAP: Inhibitor of apoptosis
IGR: Insect growth regulator
IPM: Integrated pest management
IR: Ionotropic receptor
JAMg: Just_Annotate_My_genome
JHE: Juvenile hormone esterase
LTR: Long terminal repeat
nAChR: Nicotinic acetylcholine receptor
OBP: Odorant-binding protein
OR: Odorant receptor
PGRP: Peptidoglycan recognition protein
PRR: Pattern recognition receptor
RDC: *Rhino, deadlock, cutoff*
RHG: *Reaper, hid, grim*
RMM: RNA recognition motif
*rpr*: *Reaper*
SFP: Seminal fluid protein
SIT: Sterile insect technique
*ssx*: *Sister-of-Sex-lethal*
*Sxl*: *Sex-lethal*
TE: Transposable element
*tra*: *Transformer*
*tra-2*: *Transformer-*2
*tsl*: *Temperature-sensitive lethal*
TWDL: Tweedle
WGS: Whole genome sequencing
