# Erratum to: The whole genome sequence of the Mediterranean fruit fly, *Ceratitis capitata* (Wiedemann), reveals insights into the biology and adaptive evolution of a highly invasive pest species

**DOI:** 10.1186/s13059-017-1155-9

**Published:** 2017-01-18

**Authors:** Alexie Papanicolaou, Marc F. Schetelig, Peter Arensburger, Peter W. Atkinson, Joshua B. Benoit, Kostas Bourtzis, Pedro Castañera, John P. Cavanaugh, Hsu Chao, Christopher Childers, Ingrid Curril, Huyen Dinh, HarshaVardhan Doddapaneni, Amanda Dolan, Shannon Dugan, Markus Friedrich, Giuliano Gasperi, Scott Geib, Georgios Georgakilas, Richard A. Gibbs, Sarah D. Giers, Ludvik M. Gomulski, Miguel González-Guzmán, Ana Guillem-Amat, Yi Han, Artemis G. Hatzigeorgiou, Pedro Hernández-Crespo, Daniel S. T. Hughes, Jeffery W. Jones, Dimitra Karagkouni, Panagiota Koskinioti, Sandra L. Lee, Anna R. Malacrida, Mosè Manni, Kostas Mathiopoulos, Angela Meccariello, Monica Munoz-Torres, Shwetha C. Murali, Terence D. Murphy, Donna M. Muzny, Georg Oberhofer, Félix Ortego, Maria D. Paraskevopoulou, Monica Poelchau, Jiaxin Qu, Martin Reczko, Hugh M. Robertson, Andrew J. Rosendale, Andrew E. Rosselot, Giuseppe Saccone, Marco Salvemini, Grazia Savini, Patrick Schreiner, Francesca Scolari, Paolo Siciliano, Sheina B. Sim, George Tsiamis, Enric Ureña, Ioannis S.Vlachos, John H. Werren, Ernst A. Wimmer, Kim C. Worley, Antigone Zacharopoulou, Stephen Richards, Alfred M. Handler

**Affiliations:** 1 Hawkesbury Institute for the Environment, Western Sydney University, Sydney, Australia; 2 Justus-Liebig-University Giessen, Institute for Insect Biotechnology, 35394 Giessen, Germany; 3 Department of Biological Sciences, Cal Poly Pomona, Pomona, CA 91768 USA; 4 Department of Entomology and Center for Disease Vector Research, University of California Riverside, Riverside, CA 92521 USA; 5 Interdepartmental Graduate Program in Genetics, Genomics & Bioinformatics, University of California Riverside, Riverside, CA 92521 USA; 6 Department of Biological Sciences, University of Cincinnati, Cincinnati, OH 45221 USA; 7 Insect Pest Control Laboratory, Joint FAO/IAEA Programme of Nuclear Techniques in Food and Agriculture, Seibersdorf, Vienna Austria; 8 Department of Environmental and Natural Resources Management, University of Patras, Agrinio, Greece; 9 Department of Environmental Biology, Centro de Investigaciones Biológicas, CSIC, 28040 Madrid, Spain; 10 Human Genome Sequencing Center, Department of Human and Molecular Genetics, Baylor College of Medicine, 77030 Houston, TX USA; 11 National Agricultural Library, USDA, 20705 Beltsville, MD USA; 12 Georg-August-Universität Göttingen, Johann-Friedrich-Blumenbach-Institut für Zoologie und Anthropologie, 37077 Göttingen, Germany; 13 Department of Biology, University of Rochester, 14627 Rochester, NY USA; 14 Department of Biological Sciences, Wayne State University, 48202 Detroit, MI USA; 15 Department of Biology and Biotechnology, University of Pavia, 27100 Pavia, Italy; 16 USDA-ARS, Pacific Basin Agricultural Research Center, 96720 Hilo, HI USA; 17 DIANA-Lab, Department of Electrical & Computer Engineering, University of Thessaly, 382 21 Volos, Greece and Hellenic Pasteur Institute, 11521 Athens, Greece; 18 Department of Entomology, University of Illinois at Urbana-Champaign, 61801 Urbana, IL USA; 19 Department of Biological Sciences, Oakland University, 48309 Rochester, MI USA; 20 Department of Biochemistry and Biotechnology, University of Thessaly, Larissa, Greece; 21 Department of Biology, University of Naples Federico II, 80126 Naples, Italy; 22 Environmental Genomics and Systems Biology Division, Lawrence Berkeley National Laboratory, 94720 Berkeley, CA USA; 23 National Center for Biotechnology Information, National Library of Medicine, National Institutes of Health, 20892 Bethesda, MD USA; 24 Institute of Molecular Biology and Genetics, Biomedical Sciences Research Centre “Alexander Fleming”, Athens, Greece; 25 Department of Biology, University of Patras, Patras, Greece; 26 USDA-ARS, Center for Medical, Agricultural and Veterinary Entomology, 1700 S.W. 23rd Drive, Gainesville, FL 32608 USA

## Erratum

After publication of our recent article [1] we noticed that Monica Munoz-Torres had been omitted from the author list. We have now added her, and the updated Funding and Authors’ contributions sections are below.

## References

[1] Papanicolaou A (2016). The whole genome sequence of the Mediterranean fruit fly, Ceratitis capitata (Wiedemann), reveals insights into the biology and adaptive evolution of a highly invasive pest species. Genome Biol.

