# Supplementary material for: The whole genome sequence of the Mediterranean fruit fly, Ceratitis capitata (Wiedemann), reveals insights into the biology and adaptive evolution of a highly invasive pest species
Source: Genome Biol. 2016 Sep 22;17:192. doi: 10.1186/s13059-016-1049-2 (PMC5034548; doi:10.1186/s13059-016-1049-2)
Supplement: Supplementary file 1 — Supplementary material A. C. capitata genome sequencing approaches, B. Automated annotations, C. Detection of bacterial sequence contamination and D. Screening the C. capitata genome sequence for potential horizontal gene transfer events. (DOCX 601 kb) [file 13059_2016_1049_MOESM1_ESM.docx]

**Additional file 1: Supplementary material: A. *C. capitata* genome sequencing approaches, B. Automated annotations, C. Detection of bacterial sequence contamination, and D. Screening the *C. capitata* genome sequence for potential horizontal gene transfer events**

**A. *C. capitata* genome sequencing approaches**

***A cautionary tale from an initial medfly genome sequencing attempt***

The Mediterranean fruit fly, *C. capitata*, is one of 30 arthropod species sequenced as a part of the pilot project for the i5K 5,000 arthropod genomes project at the Baylor College of Medicine Human Genome Sequencing Center (BCM-HGSC). The assembly reported here (see below and main text) represents the second attempt at HGSC to sequence the medfly genome. The initial attempt used isolated genomic DNA of the ISPRA medfly strain from mixed-sex embryos reared without additional inbreeding to increase genome homozygosity. The flies came from a strain maintained as an inbred colony for over 20 years at the University of Pavia laboratory, and it is likely they were subjected to unintentional selection for vigor as a laboratory population and avoidance of inbreeding depression effects. Thus, the isolated DNA contained a relatively high proportion of polymorphisms both within and between individuals. This initial genome sequencing used a Roche 454 approach that had been successfully used on other arthropod species including inbred *Drosophila* species  [1, 2].

An initial 454 fragment library was prepared and sequenced using 11.5 454 XLR Titanium platform runs generating 5,179 Mb of sequence (~10 X coverage of the ~500 Mb genome) with an average read length of 347.8 bp. An initial assembly of the 454 fragment data using Newbler version 2.3 (PreRelease-9/14/2009) produced more than 215,255 sequence contigs longer than 500 bp. The average contig size was 1,852 bp and the N50 length was 2,709 bp, with the largest contig being 70,001 bp. Efforts to further improve the assembly using additional 3 kb paired end 454 data, and early Illumina data to error correct 454 homopolymer errors helped to improve the assembly, but only marginally. The final 454 assembly had a contig N50 length of 3,115 bp, a scaffold N50 of 29,360 bp with a total assembly size of 543,139,776 bp (including captured gaps between scaffolds; 462,301,804 bp excluding gaps). This 0.1 assembly is available at <https://www.hgsc.bcm.edu/arthropods/mediterranean-fruit-fly-genome-project>, but has not been deposited into public databases due to its low quality.

***High quality medfly genome sequencing of an inbred strain***

Given the low contiguity of the resulting 454 assembly, we suspected that sequence polymorphism within the sequenced strain and dataset was restricting the potential contiguity of the assembly. Based on this hypothesis, it was decided to breed the strain specifically for genome homozyosity using a sib-sib single pair mating scheme for 12 to 20 generations. During this time period the Illumina sequencing technology and associated tools became both more reliable and cost effective for genome assembly, so an Illumina based strategy was utilized in parallel with the other i5K pilot species.

Six libraries were sequenced of nominal insert sizes including: 180 bp, 500 bp, 1 kb, 2 kb, 3 kb and 8 kb at genome coverages of 52.4x, 47.8x, 23.4x, 16.6x, 39.0x, and 12.8x, respectively (assuming a ~500Mb genome size). These raw sequences have been deposited in the NCBI SRA: Bio-project PRJNA168120 (for accessions see Additional file 2: Table S1). To prepare the 180 bp and 500 bp libraries, a gel-cut paired-end library protocol was used. Briefly, DNA was extracted from individual adult males or females from inbred lines using the Qiagen DNAeasy Blood and Tissue kit following the manufacturer’s supplementary protocol for purification of total DNA from insects. 1 µg DNA was sheared using a Covaris S-2 system (Covaris, Inc. Woburn, MA) using the 180-bp or 500-bp program. Sheared DNA fragments were purified with Agencourt AMPure XP beads, end-repaired, dA-tailed, and ligated to Illumina universal adapters. After adapter ligation, DNA fragments were further size selected by agarose gel elution and PCR amplified for 6 to 8 cycles using Illumina P1 and Index primer pair and Phusion® High-Fidelity PCR Master Mix (New England Biolabs). The final library was purified using Agencourt AMPure XP beads and quality assessed by Agilent Bioanalyzer 2100 (DNA 7500 kit) determining library quantity and fragment size distribution before sequencing.

Long mate pair libraries with 3 kb and 8 kb insert sizes were constructed according to the manufacturer’s protocol (Mate Pair Library v2 Sample Preparation Guide art #15001464 Rev. A PILOT RELEASE). Briefly, 5 µg (for 2 and 3-kb gap size library) or 10 µg (8-10 kb gap size library) of genomic DNA was sheared to desired size fragments by Hydroshear (Digilab, Marlborough, MA), then end repaired and biotinylated. Fragment sizes between 0.8-1.5 kb (1 kb) 1.8-2.5 kb (2 kb) 3-3.7 kb (3 kb) or 8-10 kb (8 kb) were purified from 1% low melting agarose gel and then circularized by blunt-end ligation. These size selected circular DNA fragments were then sheared to 400-bp (Covaris S-2), purified using Dynabeads M-280 Streptavidin Magnetic Beads, end-repaired, dA-tailed, and ligated to Illumina PE sequencing adapters. DNA fragments with adapter molecules on both ends were amplified for 12 to 15 cycles with Illumina P1 and Index primers. Amplified DNA fragments were purified with Agencourt AMPure XP beads. Quantification and size distribution of the final library was determined before sequencing as described above. Sequencing was performed at the BCM on the Illumina HiSeq2000 platform generating 100 bp paired end reads.

**B. Automated annotations**

***Maker 2.0 pipeline optimized for arthropods***

The medfly is one of 30 i5K pilot genome assemblies that were subjected to automatic gene annotation using a Maker 2.0 annotation pipeline tuned specifically for arthropods. The pipeline is designed to be systematic providing a single consistent procedure for the species in the pilot study, scalable to handle hundreds of genome assemblies, evidence guided using both protein and RNA-Seq evidence to guide gene models, and targeted to utilize extant information on arthropod gene sets. The core of the pipeline was a Maker 2 instance, modified slightly to enable efficient running on our computational resources. The genome assembly was first subjected to de-novo repeat prediction and CEGMA analysis to generate gene models for initial training of the ab-initio gene predictors. Three rounds of training of the Augustus4 and SNAP5 gene predictors within Maker were used to bootstrap to a high quality training set. Input protein data included 1 million peptides from a non-redundant reduction (90% identity) of Uniprot Ecdysozoa (1.25 million peptides) supplemented with proteomes from eighteen additional species (*Strigamia maritima, Tetranychus urticae, Caenorhabditis elegans, Loa loa, Trichoplax adhaerens, Amphimedon queenslandica, Strongylocentrotus purpuratus, Nematostella vectensis, Branchiostoma ﬂoridae, Ciona intestinalis, Ciona savignyi, Homo sapiens, Mus musculus, Capitella teleta, Helobdella robusta, Crassostrea gigas, Lottia gigantea, and Schistosoma mansoni*) leading to a final nr peptide evidence set of 1.03 million peptides. RNA-Seq from *C*. *capitata* adult males and females was used judiciously to identify exon-intron boundaries but with a heuristic script to identify and split erroneously joined gene models. We used CEGMA models for QC purposes: for *C.* *capitata*, of 1,977 CEGMA single copy ortholog gene models, 1,961 were found in the assembly and 1,935 in the final predicted gene set. Finally, the pipeline uses a nine-way homology prediction with human, *Drosophila* and *C. elegans*, and InterPro Scan5 to allocate gene names. The automated gene set is available from the BCM-HGSC website (https://www.hgsc.bcm.edu/arthropods/mediterranean-fruit-fly-genome-project) and at the National Agricultural Library (<https://i5k.nal.usda.gov>)

***NCBI Gnomon annotations***

The Gnomon automated annotation pipeline at NCBI, and details for the medfly annotation are described at the following website links, and details for the Reference sequence (Ref-Seq) database can be accessed at O'Leary et al.  [3]:

- Gnomon pipeline  [3]: <http://www.ncbi.nlm.nih.gov/genome/annotation_euk/process/>
- Annotation release 101: ftp://ftp.ncbi.nih.gov/genomes/all/GCF_000347755.2_Ccap_1.1

**C. Detection of bacterial sequence contamination**

The improved assembly was deposited in GenBank (Accession GCF_000347755.1), but during annotation it was determined that a significant amount of bacterial sequence was present. Two methods were used to identify these endosymbiotic bacterial sequences, comprising the *Pluralibacter gergoviae* genome (see below): first was a naive annotation analysis using Augustus and then a BLASTp versus Uniref100 to acquire taxonomic assignments of each scaffold, a second that used BLASTn of scaffolds segmented into 1 kb sections to a reference set of 1,097 bacterial genomes  [4], and a third used Kraken software on partially overlapping 5,000 bp segments of the scaffolds. Comparisons of these outputs, in consultation with collaborators at NCBI (T. Murphy, pers. comm.), were then made, and the BLASTn and Kraken approaches were found to be most sensitive and accurate. A final inspection aggregated these data into 18 bacterial scaffolds having a total size of 5.74 Mb, and a new assembly created that is available at GenBank (Accession GCF_000347755.2). The identified bacterial sequences were deemed gut endosymbionts and are described below, along with a search for *Wolbachia* sequences.

**D. Screening the *C. capitata* genome sequence for potential horizontal gene transfer events**

In prokaryotes and unicellular eukaryotes horizontal gene transfer (HGT) events are common [5-8], while, in contrast, in multi-cellular organisms HGT events are relatively rare [9]. However, several insect genomes have been reported to contain chromosomal insertions of bacterial origin, primarily from the most widespread symbiotic bacterial species in insects, *Wolbachia*. This includes the mosquito, *Aedes aegypti* [10, 11], the longhorn beetle, *Monochamus alternatus* [12], filarial nematodes of the genera Onchocerca, *Brugia*, and *Dirofilaria* [13, 14], parasitoid wasps of the genus *Nasonia* [14], the fruit fly *Drosophila ananassae* [14], the pea aphid *Acythosiphon pisum* [15, 16], the bean beetle *Callosobruchus chinensis* [15, 16], and the tsetse fly *Glossina morsitans morsitans* [17, 18]. A comprehensive *in silico* screen was therefore conducted in order to search the medfly genome for putative HGT events for *Wolbachia*, *Arsenophonus*, *Spiroplasma*, *Cardinium*, and *Rickettsia*.

***Symbiotic bacterial sequences***

The WGS data of *C. capitata* were used to screen for the presence of *Wolbachia* sequences, with more than 20 million reads examined during the screening process that generated more than 300 Gb of data. *Wolbachia* specific sequences were filtered from the whole genome sequence reads for each sequencing technology with MIRA, using five *Wolbachia* genomes, *w*Ri, *w*Mel, *w*Bm, *w*Pip, and *w*Gmm, as reference sequences  [18]. The cut-off value was set to 55%. All putative *Wolbachia* reads filtered from the medfly whole genome sequence data were used to perform a *de novo* assembly and were further examined using custom Blast databases, which did not reveal the presence of *Wolbachia* sequences in the medfly genome.

A similar mapping procedure was used to screen for the presence of sequences from *Arsenophonus*, *Spiroplasma*, *Cardinium*, and *Rickettsia* (see Table 1). For *Arsenophonus* we used the *Arsenophonus nasoniae* DSM 15247 as a reference genome  [19], from which 6,798 sequences were identified. For *Cardinium*, the *Cardinium* endosymbiont cEper1 of *Encarsia pergandiella* was used as a reference genome  [20], from which 690 *Cardinium* sequences were identified.


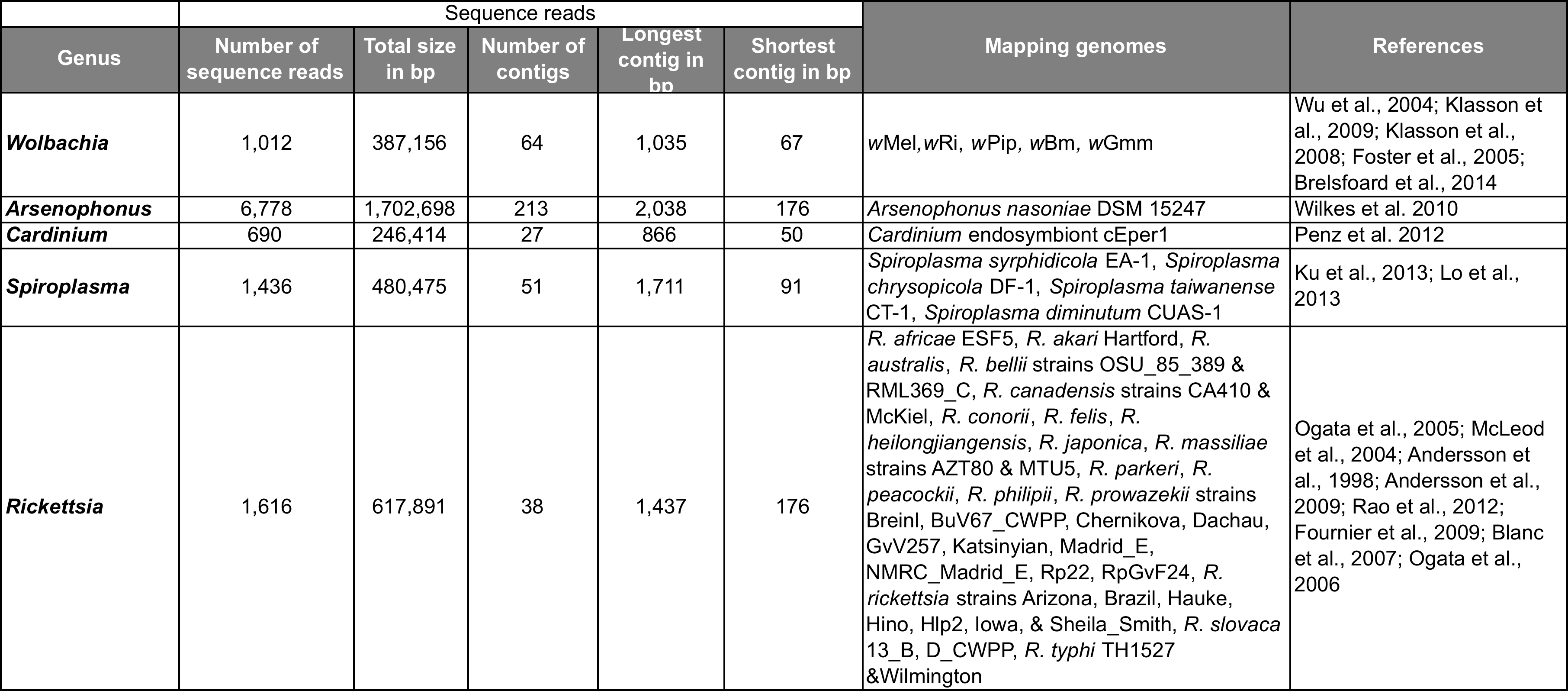
 For *Spiroplasma* we used the following complete genomes: (a) *Spiroplasma chrysopicola* DF-1, (b) *Spiroplasma syrphidicola* EA-1, complete genome, (c) *Spiroplasma taiwanense* CT-1, complete genome, and (d) *Spiroplasma diminutum* CUAS-1  [21, 22]. At least 1,436 *Spiroplasma* sequences were identified. For *Rickettsia*, 39 complete genomes were used as reference genomes from which 1,616 sequences were identified. For all identified sequences a *de novo* assembly was carried out and the final number of contigs per symbiont, together with the size of the longest and shortest contig provided in Table 1. Further examination with custom BLAST databases indicated that these contigs did not contain *Arsenophonus*, *Cardinium*, *Spiroplasma,* or *Rickettsia* specific sequences, but they were detected due to sequence homology with ribosomal genes.

Table 1. Metrics for identified symbiont sequences

***Annotation of the* Pluralibacter gergoviae *genome***

Annotation of the *P. gergoviae* sequence was performed, which was recovered in two contigs as apparent contaminants of the medfly whole genome sequence. Comparisons with *P. gergoviae* and the *Enterobacter cloacae* strain CAV1669 revealed 100% and 99.8% sequence identity, respectively.

*Genome properties.* The genome is organized into two scaffolds (38 and 52) of 3,030,794 bp and 2,627,467 bp, with a GC content of 52.7% and 53.8%, respectively. There are 4,505 protein coding genes (CDS) and 23 tRNA genes. Of the total CDSs, 61.2% represent functional categories. The distribution of genes into functional categories is presented in Additional file 2: Table S2.

*Annotation*. The genome was annotated with XBASE and RAST  [23, 24], followed by manual curation. Putative protein-encoding genes were identified using GLIMMER and Prodigal  [25, 26]. Non-coding genes and miscellaneous features were predicted using tRNAscan-SE [27] and RNAmmer [28] (Additional file 2: Table S3).

**References**

1. Chipman AD, Ferrier DE, Brena C, Qu J, Hughes DS, Schröder R, Torres-Oliva M, Znassi N, Jiang H, Almeida FC, et al: **The first myriapod genome sequence reveals conservative arthropod gene content and genome organisation in the centipede Strigamia maritima.** *PLoS Biol* 2014, **12:**e1002005.

2. Heliconius Genome C: **Butterfly genome reveals promiscuous exchange of mimicry adaptations among species.** *Nature* 2012, **487:**94-98.

3. O'Leary NA, Wright MW, Brister JR, Ciufo S, Haddad D, McVeigh R, Rajput B, Robbertse B, Smith-White B, Ako-Adjei D, et al: **Reference sequence (RefSeq) database at NCBI: current status, taxonomic expansion, and functional annotation.** *Nucleic Acids Res* 2016, **44:**D733-745.

4. Wheeler D, Redding AJ, Werren JH: **Characterization of an Ancient Lepidopteran Lateral Gene Transfer.** *PLoS ONE* 2013, **8:**e59262.

5. Hacker J, Kaper JB: **Pathogenicity islands and the evolution of microbes.** *Annu Rev Microbiol* 2000, **54:**641-679.

6. Koonin EV, Makarova KS, Aravind L: **Horizontal gene transfer in prokaryotes: quantification and classification.** *Annu Rev Microbiol* 2001, **55:**709-742.

7. Duron O: **Lateral transfers of insertion sequences between Wolbachia, Cardinium and Rickettsia bacterial endosymbionts.** *Heredity (Edinb)* 2013, **111:**330-337.

8. Andersson JO: **Lateral gene transfer in eukaryotes.** *Cell Mol Life Sci* 2005, **62:**1182-1197.

9. Kurland CG: **Something for everyone. Horizontal gene transfer in evolution.** *EMBO Rep* 2000, **1:**92-95.

10. Klasson L, Kambris Z, Cook PE, Walker T, Sinkins SP: **Horizontal gene transfer between Wolbachia and the mosquito Aedes aegypti.** *BMC Genomics* 2009, **10:**33.

11. Woolfit M, Iturbe-Ormaetxe I, McGraw EA, O'Neill SL: **An ancient horizontal gene transfer between mosquito and the endosymbiotic bacterium Wolbachia pipientis.** *Mol Biol Evol* 2009, **26:**367-374.

12. Aikawa T, Anbutsu H, Nikoh N, Kikuchi T, Shibata F, Fukatsu T: **Longicorn beetle that vectors pinewood nematode carries many Wolbachia genes on an autosome.** *Proc Biol Sci* 2009, **276:**3791-3798.

13. Fenn K, Conlon C, Jones M, Quail MA, Holroyd NE, Parkhill J, Blaxter M: **Phylogenetic relationships of the Wolbachia of nematodes and arthropods.** *PLoS Pathog* 2006, **2:**e94.

14. Hotopp JCD, Clark ME, Oliveira DCSG, Foster JM, Fischer P, Torres MCM, Giebel JD, Kumar N, Ishmael N, Wang S, et al: **Widespread Lateral Gene Transfer from Intracellular Bacteria to Multicellular Eukaryotes.** *Science* 2007, **317:**1753-1756.

15. Nikoh N, Tanaka K, Shibata F, Kondo N, Hizume M, Shimada M, Fukatsu T: **Wolbachia genome integrated in an insect chromosome: evolution and fate of laterally transferred endosymbiont genes.** *Genome Res* 2008, **18:**272-280.

16. Kondo N, Nikoh N, Ijichi N, Shimada M, Fukatsu T: **Genome fragment of Wolbachia endosymbiont transferred to X chromosome of host insect.** *Proc Natl Acad Sci U S A* 2002, **99:**14280-14285.

17. Initiative IGG: **Genome sequence of the tsetse fly (Glossina morsitans): vector of African trypanosomiasis.** *Science* 2014, **344:**380-386.

18. Brelsfoard C, Tsiamis G, Falchetto M, Gomulski LM, Telleria E, Alam U, Doudoumis V, Scolari F, Benoit JB, Swain M, et al: **Presence of extensive Wolbachia symbiont insertions discovered in the genome of its host Glossina morsitans morsitans.** *PLoS Negl Trop Dis* 2014, **8:**e2728.

19. Wilkes TE, Darby AC, Choi JH, Colbourne JK, Werren JH, Hurst GD: **The draft genome sequence of Arsenophonus nasoniae, son-killer bacterium of Nasonia vitripennis, reveals genes associated with virulence and symbiosis.** *Insect Mol Biol* 2010, **19 Suppl 1:**59-73.

20. Penz T, Schmitz-Esser S, Kelly SE, Cass BN, Muller A, Woyke T, Malfatti SA, Hunter MS, Horn M: **Comparative genomics suggests an independent origin of cytoplasmic incompatibility in Cardinium hertigii.** *PLoS Genet* 2012, **8:**e1003012.

21. Ku C, Lo WS, Chen LL, Kuo CH: **Complete genomes of two dipteran-associated spiroplasmas provided insights into the origin, dynamics, and impacts of viral invasion in spiroplasma.** *Genome Biol Evol* 2013, **5:**1151-1164.

22. Lo WS, Ku C, Chen LL, Chang TH, Kuo CH: **Comparison of metabolic capacities and inference of gene content evolution in mosquito-associated Spiroplasma diminutum and S. taiwanense.** *Genome Biol Evol* 2013, **5:**1512-1523.

23. Chaudhuri RR, Loman NJ, Snyder LA, Bailey CM, Stekel DJ, Pallen MJ: **xBASE2: a comprehensive resource for comparative bacterial genomics.** *Nucleic Acids Res* 2008, **36:**D543-546.

24. Aziz RK, Bartels D, Best AA, DeJongh M, Disz T, Edwards RA, Formsma K, Gerdes S, Glass EM, Kubal M, et al: **The RAST Server: rapid annotations using subsystems technology.** *BMC Genomics* 2008, **9:**75.

25. Delcher AL, Harmon D, Kasif S, White O, Salzberg SL: **Improved microbial gene identification with GLIMMER.** *Nucleic Acids Res* 1999, **27:**4636-4641.

26. Hyatt D, Chen GL, Locascio PF, Land ML, Larimer FW, Hauser LJ: **Prodigal: prokaryotic gene recognition and translation initiation site identification.** *BMC Bioinformatics* 2010, **11:**119.

27. Schattner P, Brooks AN, Lowe TM: **The tRNAscan-SE, snoscan and snoGPS web servers for the detection of tRNAs and snoRNAs.** *Nucleic Acids Res* 2005, **33:**W686-689.

28. Lagesen K, Hallin P, Rodland EA, Staerfeldt HH, Rognes T, Ussery DW: **RNAmmer: consistent and rapid annotation of ribosomal RNA genes.** *Nucleic Acids Res* 2007, **35:**3100-3108.
