# Supplementary material for: The whole genome sequence of the Mediterranean fruit fly, Ceratitis capitata (Wiedemann), reveals insights into the biology and adaptive evolution of a highly invasive pest species
Source: Genome Biol. 2016 Sep 22;17:192. doi: 10.1186/s13059-016-1049-2 (PMC5034548; doi:10.1186/s13059-016-1049-2)
Supplement: Supplementary file 3 — Supplementary figures S1–S9. Figure S1 Odorant-binding protein (OBP) genes phylogenetic tree. Figure S2 Ionotrophic receptor (IR) genes phylogenetic tree. Figure S3 Opsin genes phylogenetic tree. Figure S4 CYP gene clusters A and B. Figure S5 Glutathione S-transferase genes phylogenetic tree. Figure S6 cysLGIC genes superfamily phylogenetic tree. Figure S7 Cuticle protein gene clusters. Figure S8 CPLCA cuticle protein genes phylogenetic tree. Figure S9 Seminal fluid protein functional classes. (PDF 9425 kb) [file 13059_2016_1049_MOESM3_ESM.pdf]

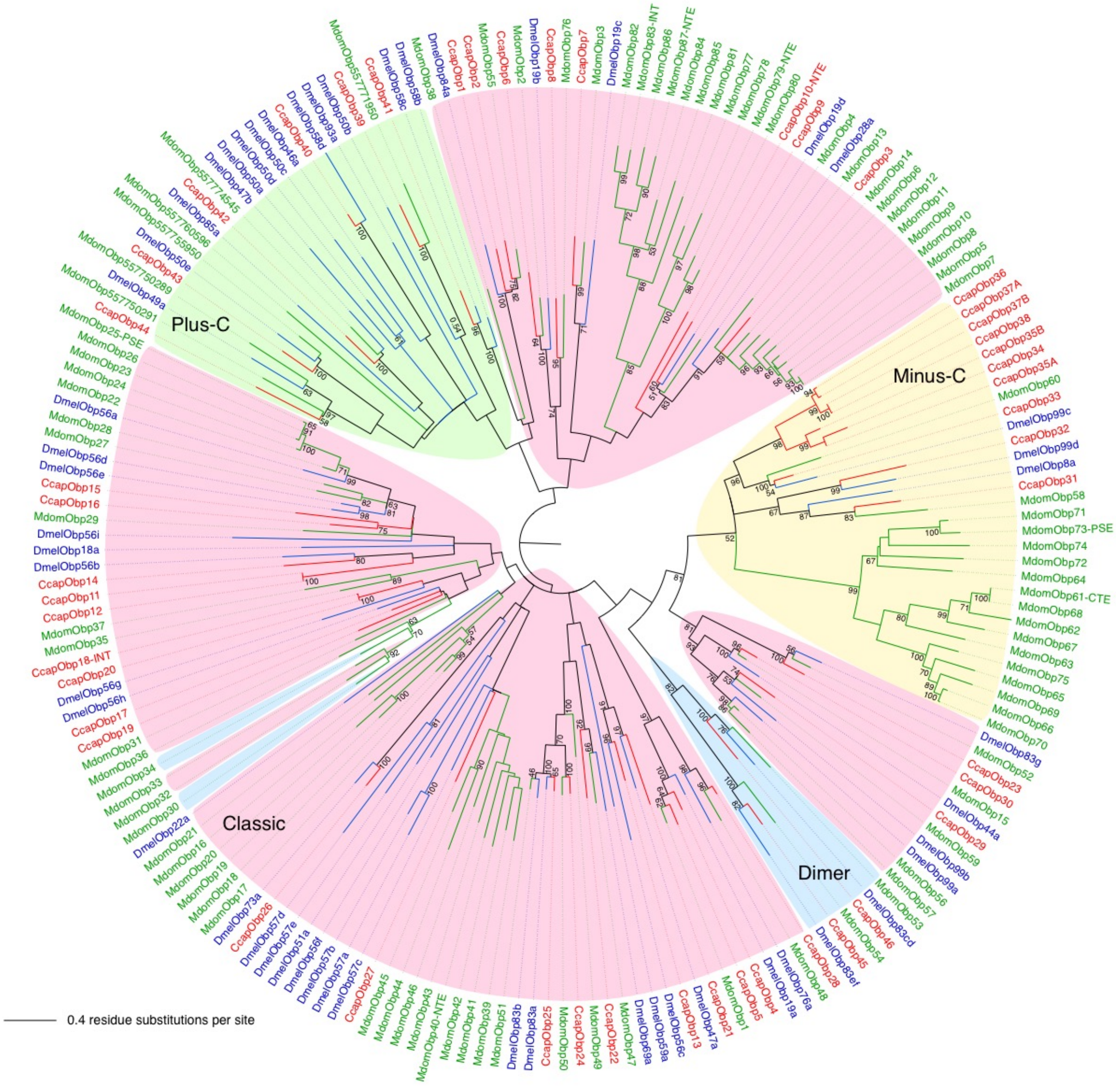

Figure S1. Phylogenetic relationships of OBPs from *C. capitata*, *D. melanogaster* and *M. domestica*. The midpoint rooted maximum likelihood (log likelihood = -33834) tree was inferred using the Le & Gascuel model [208] with a discrete Gamma distribution and some invariable sites. Bootstrap values greater than 50% (1000 replications) are shown. The different subfamilies of OBPs are highlighted by colored shading. Suffixes after the gene/protein names are: -NTE N-terminus missing; -CTE C-terminus missing, -INT internal region missing, -PSE pseudogene.

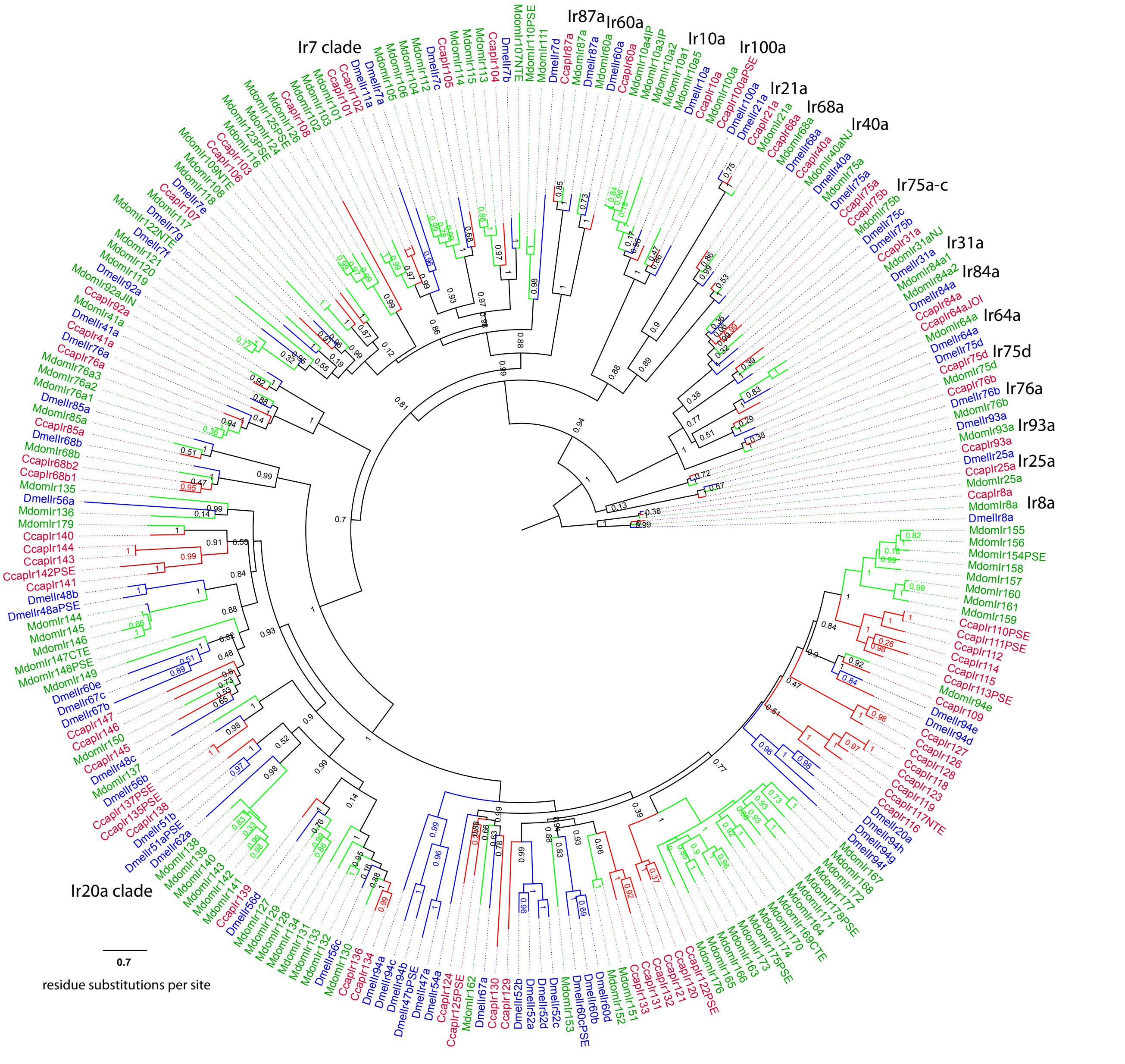

Figure S2. Phylogenetic tree of the *Ceratitis capitata* IRs (red) with those of *Drosophila melanogaster* (blue) and *Musca domestica* (green). This maximum likelihood tree was rooted by declaring the Ir8a and 25a lineages as the outgroup. Lineages discussed in the text are labeled on the outside.

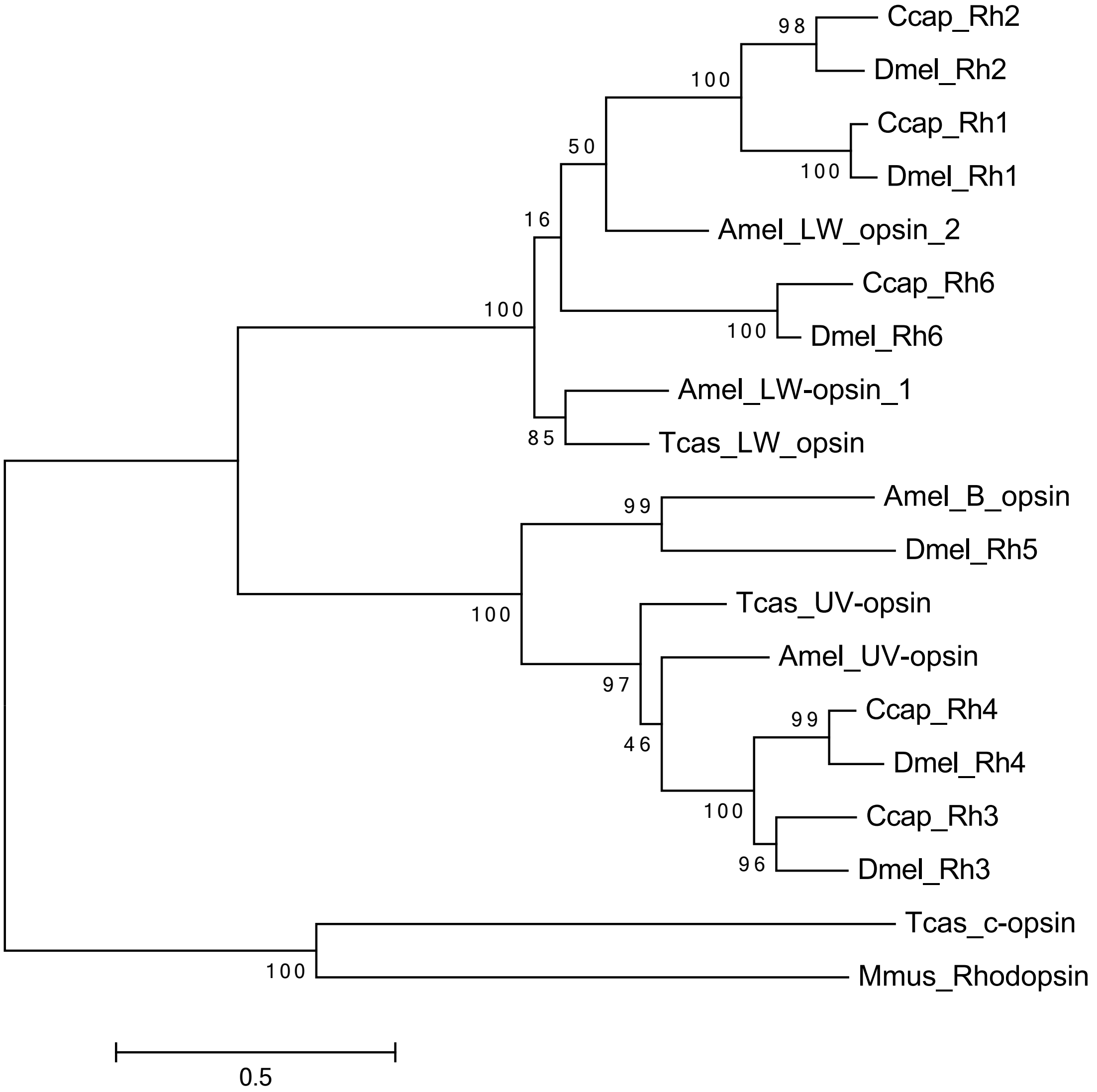

Figure S3. Maximum likelihood tree of *D. melanogaster* and *C. capitata* opsin genes. Protein sequences were aligned with Webprank [2]. Ambiguous alignment regions were filtered using TrimAl (v. 1.3) [1] as implemented on the Phylemon 2.0 server [3] applying User defined settings (Minimum percentage of positions to conserve: 10, Gap threshold: 0.9, Similarity threshold: 0.0, Window size: 1.0). Maximum likelihood tree was estimated in MEGA version 6.0 [4] applying the Jones-Taylor-Thornton (JTT) model of amino acid sequence evolution and assuming Gamma Distributed substitution rates across sites with 3 categories. Numbers at branches represent nonparametric bootstrap support from 100 replications. Species Abbreviations of species that were investigated using genome draft annotations: Amel = *Apis mellifera*, Ccap = *Ceratitis capitata*, Dmel = *Drosophila melanogaster*, Mmus = *Mus musculus*, Tcas = *Tribolium castaneum*. Alignment available on request. References: **[**1] Capella-Gutiérrez S *et al.*, *Bioinformatics* **25**, 1972-1973 (2009); [2] Löytynoja A, Goldman, N, *BMC Bioinformatics* **11**, 579 (2010); [3] Sánchez R *et al.*, *Nucleic Acids Res.* **39**, W470-474 (2011); [4] Tamura K *et al.*, *Mol Biol Evol* **30**, 2725-2729 (2013).

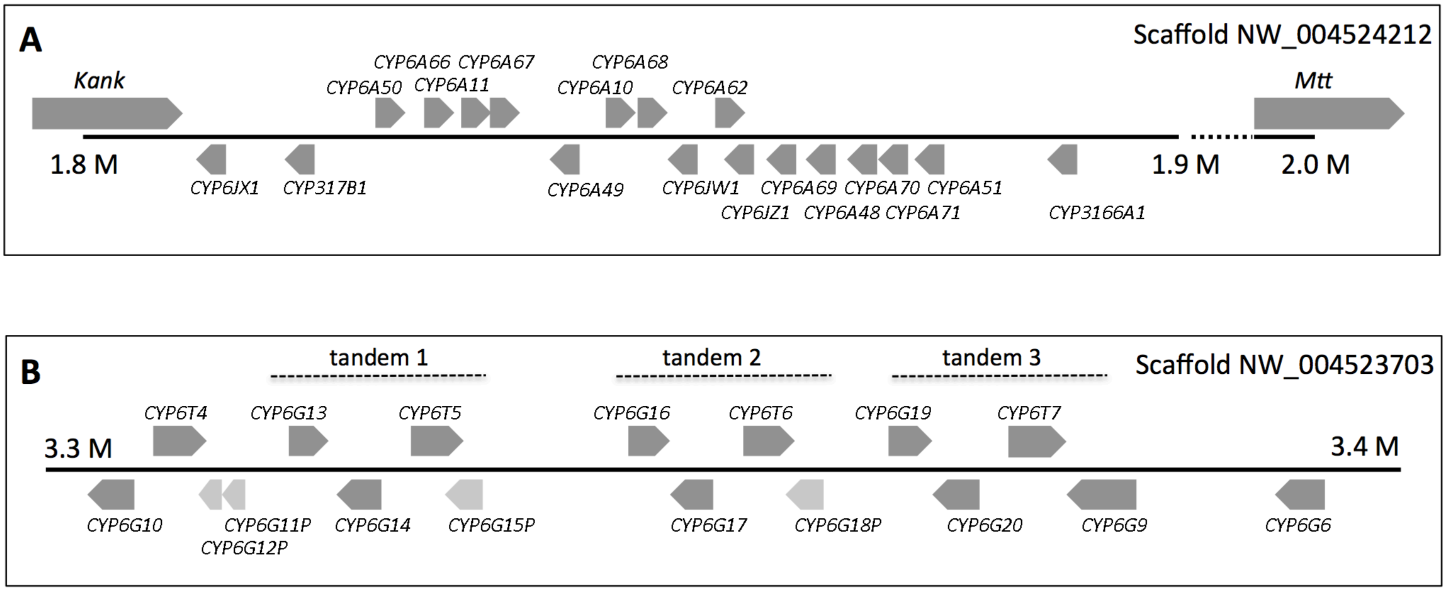

Figure S4. Schematic representation of two CYP clusters in the genome of *Ceratitis capitata*. **A)** Eighteen consecutive CYP genes in tandem flanked by orthologs ot the *Kank* and *Mtt* genes, and **B)** CYP6G/CYP6T cluster; the dotted lines indicate the tandem repeats of two CYP6G genes in opposite orientation followed by one CYP6T and one CYP6G gene or pseudogene. Pseudogenes are shown in light gray.

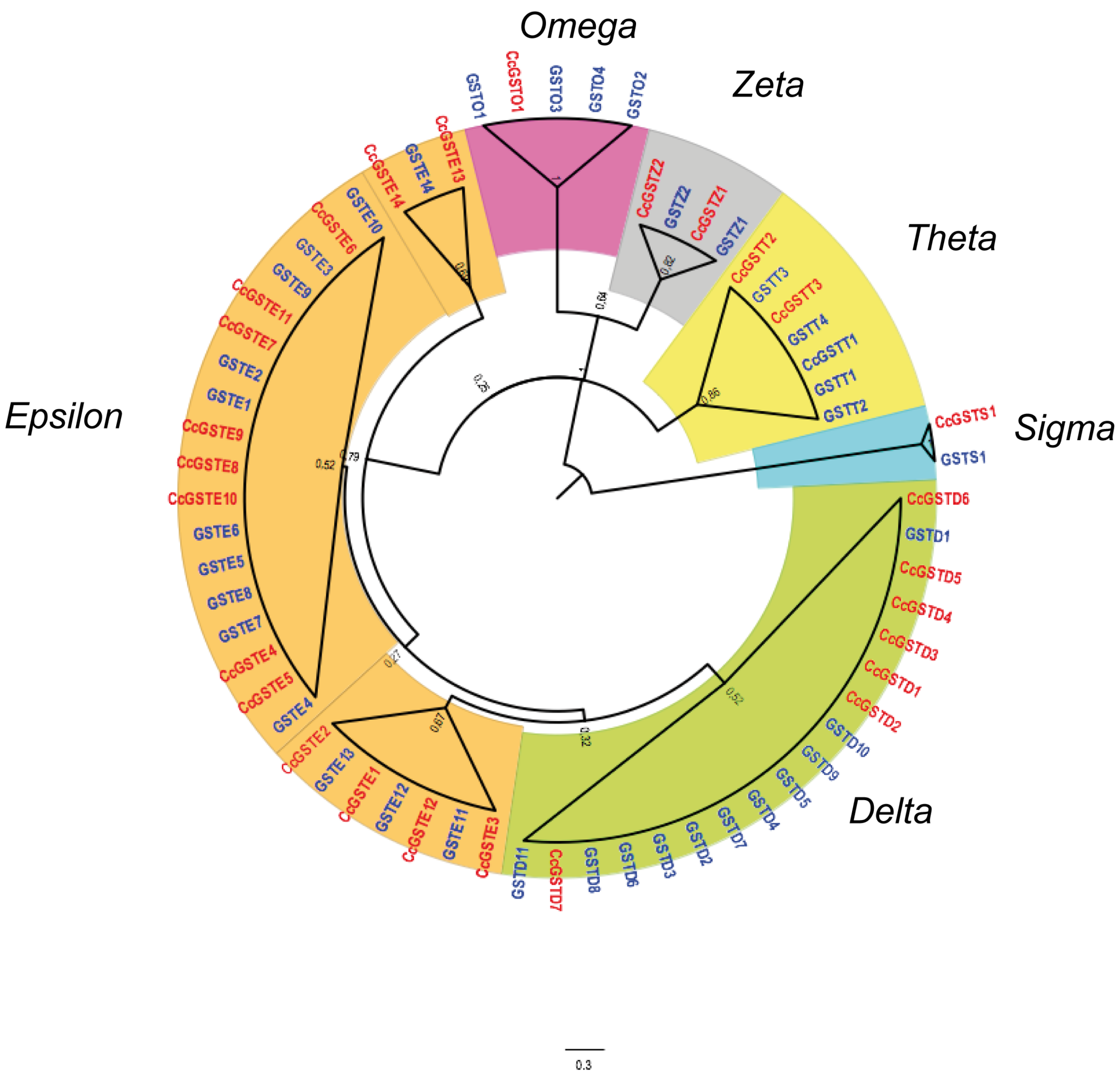

Figure S5. Bootstrap PhyML tree of *C. capitata* (red) and *D. melanogaster* (blue) (http://phylogeny.limm.fr) GST protein sequences. Branch length indicates average residue substitutions per site.

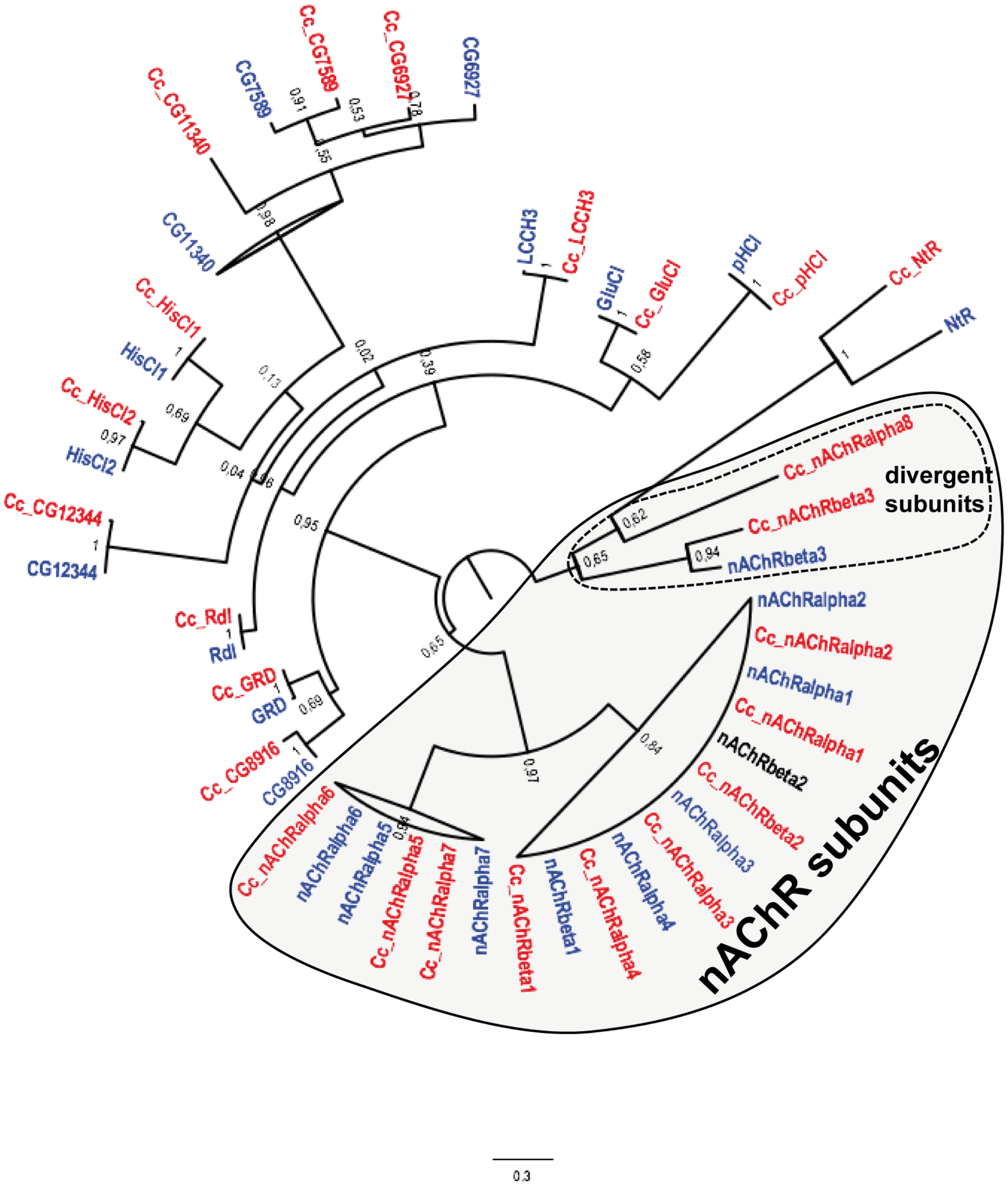

Figure S6. Bootstrap PhyML tree of *C. capitata* (red) and *D. melanogaster* (blue) (http://phylogeny.limm.fr) cysLGIC protein sequences. Branch length indicates average residue substitutions per site.

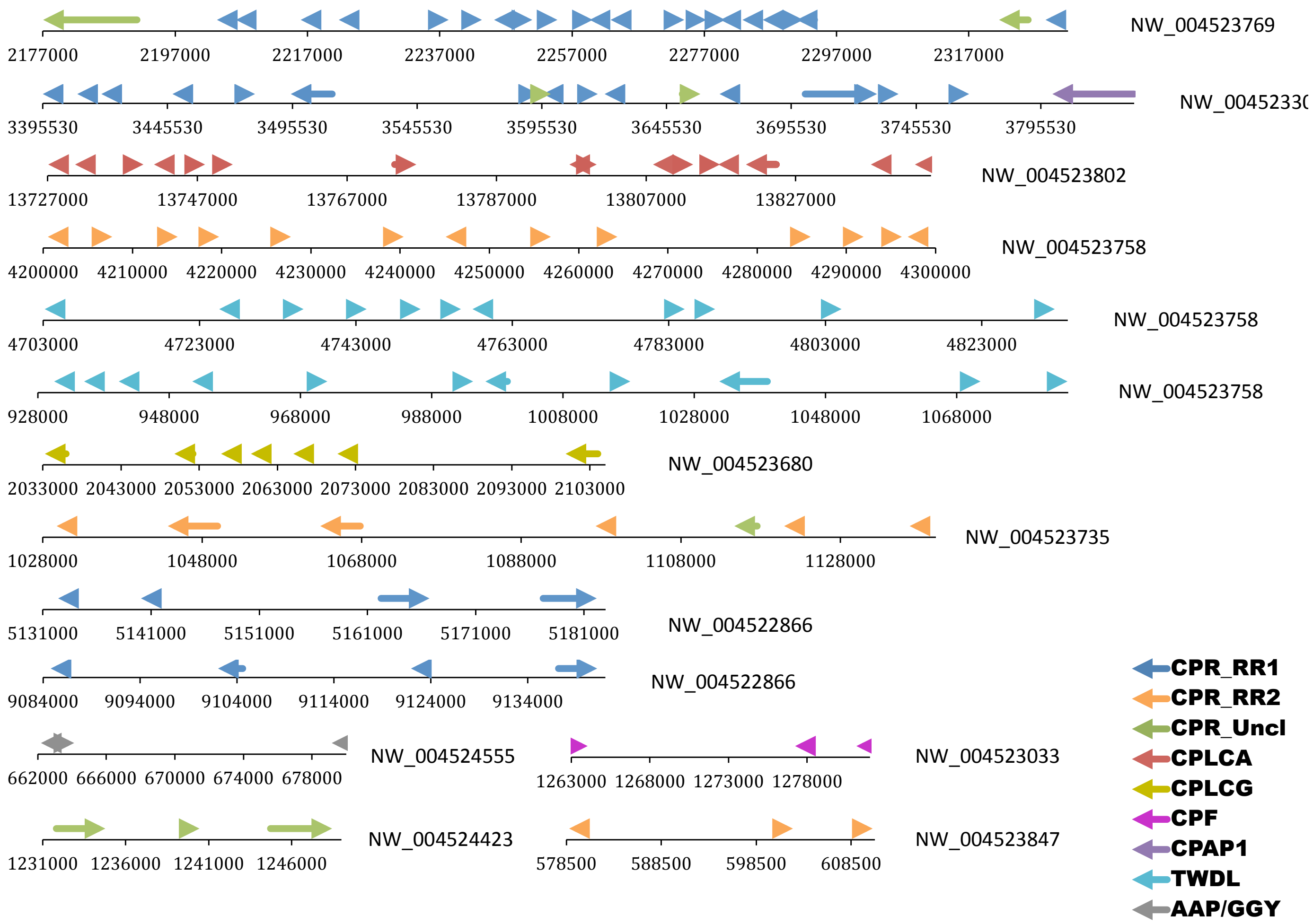

Figure S7. Gene clusters of cuticle proteins from *C. capitata*. Arrows indicate orientation of each putative cuticle protein gene. Scaffold numbers are shown to the right of each cluster and genomic coordinates are indicated below the scaffold (not to scale).

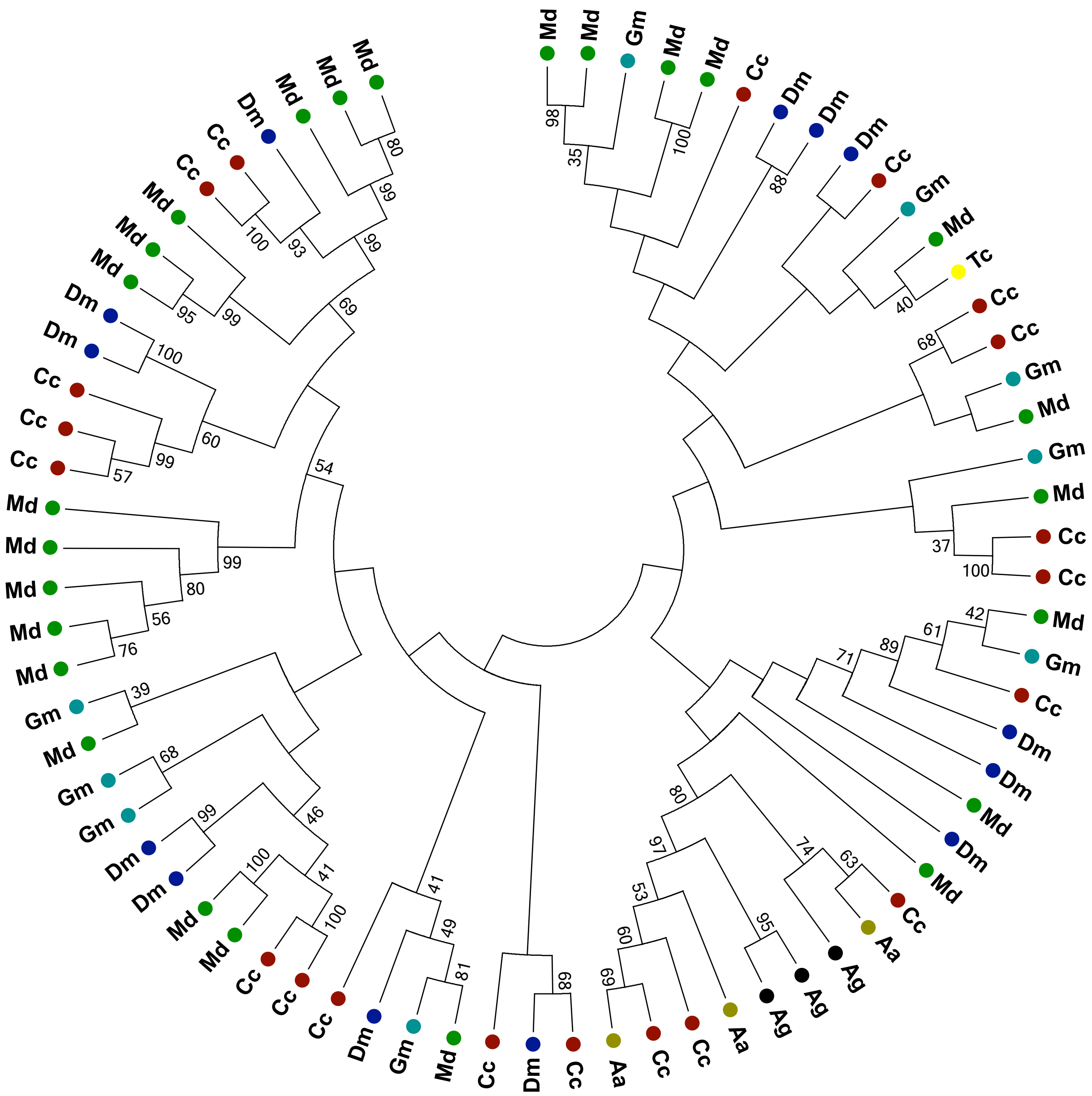

Figure S8. Phylogenetic tree demonstrating the relationship of CPLCA proteins from *C. capitata* (Cc), *D. melanogaster* (Dm), *Musca domestica* (Md), *Anopheles gambiae* (Ag), *Aedes aegypti* (Aa), *Glossina morsitans* (Gm), *Culex quinquefasciatus* (Cq), and *Tribolium castaneum* (Tc). The tree was constructed using the neighbor-joining method in MEGA6; Poisson correction and bootstrap replicates (2,000 replicates) were used.

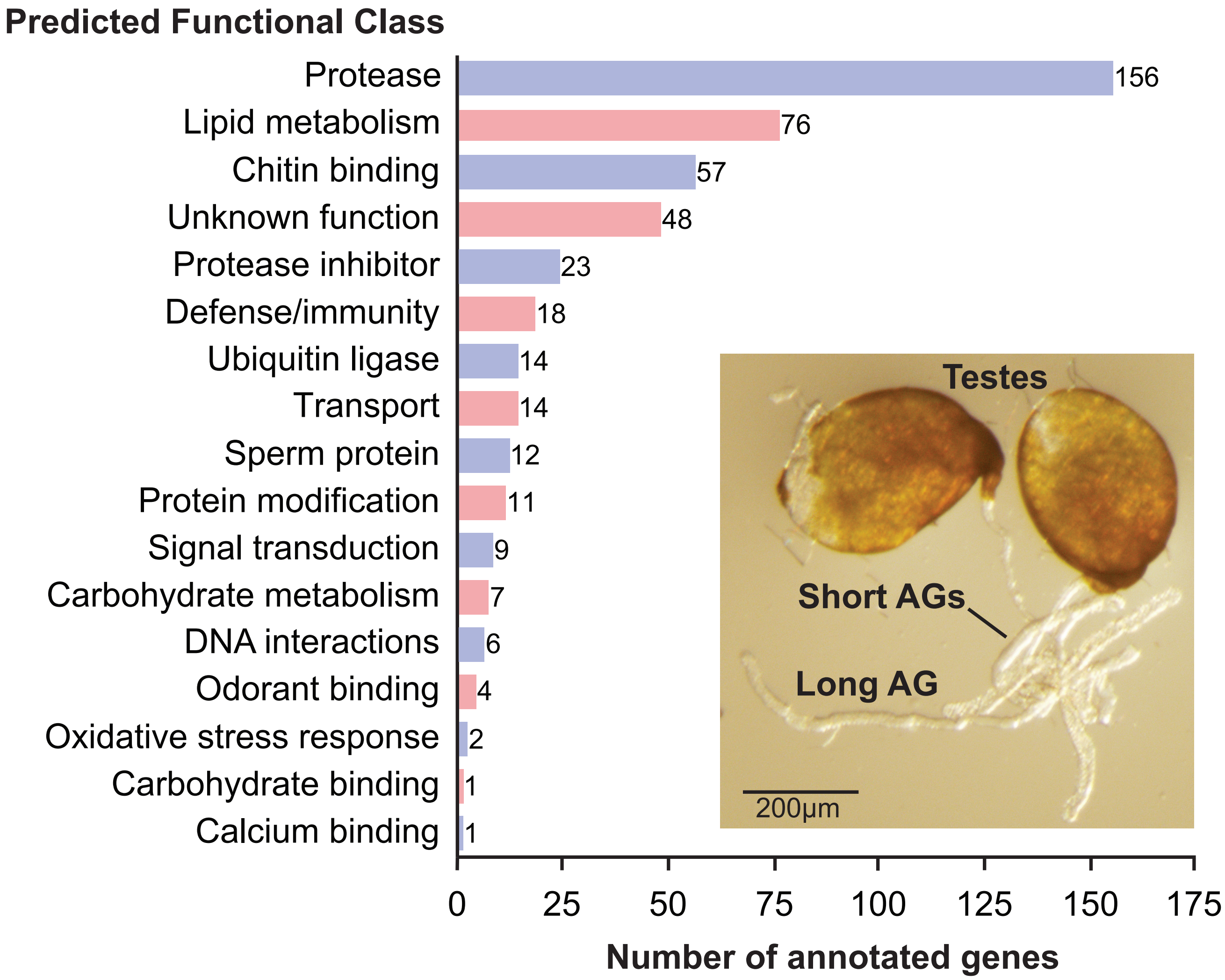

Figure S9. Proposed functional classes of putative Seminal Fluid Protein (SFP) coding genes identified in the medfly genome. The box figure shows a dissected male reproductive tract including the testes and the accessory glands (AGs).
