# Supplementary material for: The whole genome sequence of the Mediterranean fruit fly, Ceratitis capitata (Wiedemann), reveals insights into the biology and adaptive evolution of a highly invasive pest species
Source: Genome Biol. 2016 Sep 22;17:192. doi: 10.1186/s13059-016-1049-2 (PMC5034548; doi:10.1186/s13059-016-1049-2)
Supplement: Supplementary file 4 — Supplementary material: C. capitata chemoreceptor genes. (DOCX 194 kb) [file 13059_2016_1049_MOESM4_ESM.docx]

**Additional file 4: Supplementary material: *C. capitata* chemoreceptor genes**

Insect olfaction and gustation is the product of a signal transduction cascade that includes four major gene families [1]: the insect odorant binding proteins (OBPs) that are small, globular, water-soluble proteins secreted into the sensillar lymph by basal support cells. The OBPs bind and solubilize odorant molecules and transport them through the aqueous lymph to the membrane-bound odorant receptors (ORs) that act as ligand-gated ion channels [1, 2]. The gustatory receptors (GRs) act as taste or contact receptors, and are involved in the perception of sugars and bitter tastants including carbon dioxide [3]. The ionotropic receptors (IRs) are the most ancient and highly divergent family of chemoreceptors, which evolved from the ionotropic glutamate receptor superfamily and respond to amines, acids and other odorants not perceived by the ORs [4, 5].

**The Odorant Binding Protein (OBP) family**

Insect OBPs are small, globular, water-soluble proteins that are secreted into the sensillar lymph by basal support cells. The OBPs bind and solubilize odorant molecules and transport them through the aqueous lymph to the membrane-bound ORs  [1, 2]. However, not all OBP are restricted to chemosensory appendages and it is likely that they may be involved in other physiological functions unrelated to chemoreception  [6-8].

The OBP family is composed of different subfamilies of proteins. The members of the Classic OBP subfamily generally contain six highly conserved cysteine residues that form three disulphide bonds. Members of the Minus-C OBP subfamily contain four conserved cysteine residues and only two disulphide bonds. The Plus-C subfamily contain additional conserved cysteine residues  [9], while Dimer or Double OBPs have arisen where two “classic” OBP domains have fused into one protein  [2].

Forty-six OBP genes were identified in the medfly genome (Table S3), compared to 52 in *Drosophila melanogaster*  [2] and 85 in *Musca domestica*  [10]. The N-terminus of CcapObp10 is missing due to a gap in the genome sequence, but its complete sequence has previously been determined  [11]. The central part of the sequence of CcapObp18 is interrupted by a gap in genome, but again its complete transcript has previously been described  [11]. The predicted translations of the complete OBP transcripts ranged from 123 amino acids (CcapObp20) to 336 amino acids (CcapObp13). Alignments of the conceptual amino acid sequences of the medfly OBPs with those from *D. melanogaster* and *M. domestica* permitted their classification into different subfamilies. On the basis of the conserved cysteine domain profiles, 30 were classified as Classic subfamily OBPs (6 conserved cysteines, CcapObp1-30), eight as Minus-C OBPs (4 conserved cysteines, CcapObp31-38), six as Plus-C OBPs (more than 6 conserved cysteines, CcapObp39-44), and two as Dimer OBPs (with a double domain profile, CcapObp45-46). The medfly appears to lack orthologs of a number of *Drosophila* and *M. domestica* OBP genes, but it also shows at least one example of gene lineage expansion. The *Drosophila* Minus-C subfamily DmelObp99c has only one ortholog in *M. domestica*, but in the medfly this lineage has expanded to include six related minus-C OBPs, CcapObp33-38 (Figure S1). This group has expanded ever further, as two genes, CcapObp35 and CcapObp37, appear to encode two different OBP proteins by differential splicing (CcapObp35A/B and CcapObp37A/B, respectively). As in other insects, many of the genes are in arrays of multiple genes. Their gene structures are fairly complicated, with 0-4 introns.

The phylogenetic tree reveals that there are fifteen simple 1:1:1 orthologous relationships, for example, CcapObp28 is an ortholog of MdomObp48 and DmelObp76a/LUSH  [12]. Another three have simple duplications in one of the three species (e.g. CcapObp1/2 are duplicates of DmelObp84a/MdomObp55). Unlike the extensive gene lineage expansions in *M. domestica*  [10], there is really only case of considerable gene expansion in the medfly (CcapObp34-38) all in a single array with no clear orthologs in *D. melanogaster* or *M. domestica*.

The Dimer OBPs CcapObp45 and CcapObp46 are clearly orthologs of the DmelObp83ef/MdomObp54 and DmelObp83cd/MdomObp53 Dimer OBP genes, indicating that these genes are older than the split of the three fly lineages. In contrast, neither *D. melanogaster* nor *C. capitata* possess orthologs of the MdomObp30 and MdomObp34 Dimer OBPs, reinforcing the hypothesis that such genes can arise as a result of fusion of two duplicated “classic” genes  [10].

**48 CcapObp proteins in FASTA format.**

**Classic OBP subfamily**

>CcapObp1

MFRKAVCIKLPLLLILVYYIVASIQDHAKDNGDIYVQHKQDSKCFNNLEREANATIPLQISNSTGNELEDVVRLCNSSFSIPTDHIMQFNTDGALADTVDKTGMCFIRCYFEKSGLIQNWKLNKDQIVQKMWPLAADSVDFCESEAKQEMNACVRTYAIAKCLMQRRFEGTCNPSAV

>CcapObp2

MLDHRHLNIILLLLQFGIMSSGAADAQTNLTNNASDQLQAVENTTPMVEEEHEMGFDFDAVVRTCNASFAIPLELIQRFNETAELPNTTDKTGMCFLKCYMEGTGLLRNWQLNRSLIRQTMWPATGDSIPVCQEEGSRESCPCKRTYAIAKCLMIRALVDARNKPIV

>CcapObp3

MAKFILIAALCILSAALTKAAFNKEEAIKTFMTRAEECRGEAGAADSDIQDLITKSPAAGKEGKCMRSCIMKKYGVMDSNGKFVKSVAMEHTKTYTDGDADKLKIANEIIDACADIAVPDDHCEATELYGKCFMEQTKAHGIDKFEF

>CcapObp4

MLPRMNLFVLPAVLIVLTLNTDRVYGGATEEQMIAAGKLMRDVCLPKFKVPTDVADGIKDGIVPETKEVKCYINCILEMMQTMKKGKFLYESSLKQVDLLMPDDYKDQYKSGFAACKDSPNGIKNNCDASYALLICMRDKITKFVFP

>CcapObp5

MKYFKVLLTSCIMVLFFIAPTFAGVTEEQMWATAKLMRDVCLPRFPKIATELADQLRDGNIPDNNKDVKCYINCVLEMMQTMKKGKFLYEASLKQVDLILPDSYKDDYRAGLLKCKDASAHIKKDNCESAYTVLKCLRGEIKKFIFP

>CcapObp6

MFHLCVAAFLISMMGDVLADEMMTLPIGMLIEGVEPFVQDCDPKPELEHMEELFLNKEDAQQTTKCLRHCLMAQFELFKEGDTAVESEKLVGFMALLYPEKADELNEIVDGCNQRNKDMGLNEMCEVAHSFGMCMLKEMQSREYEIPIVEQ

>CcapObp7

MYSNKTLLFASLICLLAVESINALNANSYKLSGKRKPLATREDATIIEDYKRTKRQLSQPMQEFQAFITTSKGQCASEMGFKANEMEKSLLYEELPTPKEKCMMECILKRMEVMDRDNMLSTPAIGRIADIIGNNNALITSIAMASADNCKKFITAEDPCERAYQINKCIATEMKMRKIKLIY

>CcapObp8

MTLVVADSKPPHYSSLRLMAEDAIGDCYEDAARSVKVEITDEGFDELLKGSRDNLSRNAKCLRYCIMRKNGLLNELNSVDEEKIIQIFQIIHPQIEREKLLSVIRKCSEETEKQTDNCERAFLAAKCILLELKEDGVTDI

>CcapObp9

MKFLNICLIVCAALIANTKCDDFEGVKLTAQECKDEVGATDEDVETMFKHEPAGSAEAKCLHACVMKRFGLMNDDGKMDKEKATDILKIIASGNEEQESLGLEVLETCVDIDVDEDHCEAAEDYRVCMHTKAAENGFTMARV

>CcapObp10-NTE (section in brackets derived from transcript from Siciliano et al. 2014a)

**[**MKLILTFLLVCGLIISNTE**]**ANDLVDKVTKLAQECKGSTGASDADYENLLNYQAPNTDKGKCLTECVLKKSGALGENNKINEAVMVEFIKAGAAGDASIEKIGMEGLQACLKVPQHPNACENAAALNACFTKNLRAKGFKGLPA

>CcapObp11

MKSFIVCCMLATTLLSFCVLSTEAGLRKPKKLTPELEAKFEVLTAWIAYRLNLKHAKEACIGEYGFTDELATNLVKIKVANPSANEKCYVNCLYNKLVFYKDSTINKQAMKESLYEIVGEERLMNIVNSCMNAGGANDCDKVYKFHACASPEFDKVRSDIFLPDE

>CcapObp12

MKSSIICCILATVVLSFCVLSTEAGLRKPKKLTPELEAKFEVLTAWIAYRLNLKHAKEACIGEYGFTDELATNLVKIKVANPSANEKCYVNCLYNKLVFYKDSTINKQAMKESLYEIVGEERLMNIVNSCMNAGGANDCDKVYKFHACASPEFDKVRSDIFLPDE

>CcapObp13

MIYTISLLCMLISAAVQNVKTRTITFSVNMSLTMDEQRQRPLLCNGQAQQRTRPSITAAVLRSCMKETELSMAELRRFRLSLLSHEPQEANAFDESTTEQPSLGEMADADFSEEEDIDGIVYETESAVVMEPTSIAVEHEEPVAFKIDGKSDESLQCFAYCLYEQLGLISKGVYMEEELFAKLYAVVGRERHLVKECMDLNTNNKCESSYKMHLCYARLKTLEAEKRLHDVLGPIEGEEKDREEVEMTQKITTLESVETTMETEPNTKPNPTVEADTTLESKSMMEPEKTMYPFIFQDYAEDEYTKLNMKKKMKKFIKKLKSEDLTNRSYNELLGR

>CcapObp14

MKSFGLLQSFLVILIIGPISELLAADDKTARESCIKEVNLSAADANSVRGTAMISKLVQNNSESLKCFQLCYYKQLGFLDASGKTNGQKVLEYMSQASGITDTAKLVAALSGCESVNGASQCDKVYQFEKCALGKLGV

>CcapObp15

MNSNIFLITFLVLFALAAANELKSHKPARNHKIYEDCMKESGASVAQLEALKKGDFNSIDNKAKCFLKCLEDNKGILTNGMPNEAGIRKKVHAPPAGNGVSKDLLAKCNGLKGADECDTAYQIYKCFMQEKVPLI

>CcapObp16

MKFFTVAIVLAFVAAAAAQELIKLTDEQKQKVHALGAECIKETGAAEEAVRAAGKGDYSQVDGKVKCFAKCLQGKLGYVEDGKLNEAAIQASLGKIVGDEQIKAIQAKCNGVKGADDCDTAFELHKCYLAENANIQI

>CcapObp17

MQKIYLLTIMAALASVAVSQLPADVEKYHRACLDESKVSDDELKQFFQNGMRASDATENIKCHNKCMMQKMGIFKDGVFDADAKMKELMQNPSMKGHEAEIKQALNDCKNEKGANECDTSFKITMCLKDFSKPQHA

CcapObp18-INT (section in brackets derived from transcript from Siciliano et al. 2014a)

MKSYFALTLFVISCAVVM**[**CHPPPDPEMRKYIDECTKDHNVTPKEFHEFMEGKAASPSENLKCSSHCVMLKQGIIDEAGNF**]**KPDVAKSKMPDDKLAAAIDDCKDLSGSSACDTAFKITECVLSHK

>CcapObp19

MKILYILITFAALATFALCDLRVEMRKFFSYCQFEANVTETQLNKFIDNGMLASEAKSNIRCLVKCTMERYGLIRNGTYDTERAIRDFASKVPKRKGLEEDVKEAVNKCKAEKGADDCDRAFKITMCIKEFKTRFPEV

>CcapObp20

MKCFKKLFIYKKLPFHQCHPPPDPEMRKYIDECTKDHNVTPKEFHEFMEGKAASPSENLKCSSHCVMLKQGIIDEAGNFKPDVAKSKMPDDKLAAAIDDCKDLSGSSACDTAFKITECVLSHK

>CcapObp21

MSIDLSISEDQHRINSDMVHLCALETDLFMEELRKFSENDFNDTVKVTQCFTHCLYEHMGLVRDGVFVQRDVISLLGDTIDSKHIIEHECFNQCSENKCKRAFLIHQCYHGDGGKGIISSAQQFEAAEDKLNEDEKEDDSEAASDVSLPRTLIESTMPVKAGTERQIILKKLLAKRQPKIH

>CcapObp22

MNYRRLAILLIIYQCVLLRNVQSLEVPKHMRSGAKKLTNLCIKETGVTEDLFIEAQETGKMPNNQRLKCFIHCVLDKIGLIDADNIVHLDNLLEILPPEFVPIVEELHTTCGTQSGADGCETAFLTTECYIKTNPVILKLLFTTFSE

>CcapObp23

MKIQLILLLACVALVAGKFELRTAQDALAAHEACRDEFRIPDDIYEKYLNYEFPPHKRTNCYVKCFVERMGLFTEEKGFDEKAIIAQFTAKNSKNLAKVSHGLEKCLDHNEHDSDTCTWANRVFSCWISVNRPIVRKTYIAN

>CcapObp24

MYILRTILGALLWCSVLLNLIRAQKELRRDETYPPPELLEALRPVHDKCVAKTGVTEEAIKEFSDGEIHEDEPLKCYMYCVFEETDVLHEDGEVHLEKLLDSLPNSMHNIALHMGKKCLYPKGDTKCERAFWLHRCWKESDPKHYFLI

>CcapObp25

MALNGSRRSQAFNAFLIVALALSSTLLMHVVQAQEPRRDDKWPPPAVLKMAKIFHDICVEKTGVSEEAIKEFSDGQIHDDEALKCYMNCLFHEIDVVDDNGDVHLETLYNTVPGTVRDKLINMAKGCEHPEGDTLCHKAWWFHQCWKKADPVHYFLP

>CcapObp26

MGNNAIFRISFCITLSVSINAKLFTNSSQGHYLTKNRCLNPPRTARRVENFIQECQEEVKNKILNEAFLILKDEAYKDYSTLDMNNYNMNLGGNSGTIESLRHVIQEPSSVHMPSAAPYPTGSPRPSIRRLLHGIQRIAHDPANAVYHPTIVSYEDKRIAGCLLHCIYARNNAIDKLGWPTLDGLVDFYSEGVNEHEFFMATLRSVNLCLHAITMKYRINRRKLPEHGESCDLAFDVFDCISDQITGYCFDQYN

>CcapObp27

MMLASNGRGMSLPGLTWLVLLAAILMFFLPIDKVAASPTASTRSFMEACQVKHNITMEELDEFPDEPDADEVEMKFKCYAHCLLFGMGLLDENGKLNVEYMHDVGILTDPSYESMLECKAANDMEDDPCEYSFGMMLCARMLGTEEGGSEENIEDIEEEVEAKEEEERRK

>CcapObp28

MQEKYKAMILNTNGIKYFFTLLACASVGAVTMQQFEASLDMMRNGCAPKFKVSVDILDKLRAGEFTENNNDLRCYTRCIAQLAGTLTKKGDFSVQKALAQIPIILPPEMQDAAKEALNACKDVQKNYKESCDRVFYTTKCVRDYDPTTFKFP

>CcapObp29

MKFCLALLSLFFAVVVADHGDHSDYVVKTGEDLARYRDQCVAKLSIPADLVEKYKKWEYPDDEKSRCYLKCVLESFGLFDDAKGFDVHKVHHQLGGGDVDHSNELHGKIENCAKEGDAAGEDACTRAYRGALCFFKENLALVKQNVASK

>CcapObp30

MKYIVAVLLALFALAAAEEYKLRNQDDLLKARKECMEAKKVPAEHIEKYKKFEFPDDEVTRCYIECIFNKFQLFSPTEGFKTQNLIAQLGHNKENKDAVKADIEKCADKNEQKSDSCTWAYRGFKCFISKNLPLVQESLKKN

**Minus-C OBP subfamily**

>CcapObp31

MTEAMKCSTIAFTWIIILLFNFCNFIGADFEEKTEEDFLTASERCFQRERLAASYQRQFDNFVYPDEAPVHRYVHCIWNELKLWNDRTGFNVEHIAALYRDKANTEVLVPILSDCNRNTNNAPTLKWCYNAFKCVLNSRVGQWFKEDVERKLHERRTGNHVA

>CcapObp32

MFTTRYLLIFSLLVICAISSSFTAVANPTSISDYEQPSRRLYKAHELCLPIVTDVSSAESEQGMQRAATYIRCMVSNMGLWTDSVGYKAKRVAKFFMKQHSEHEVMALVEYCNQKNQQENLDLWAFEAYRCATAGRMGTWLGEYVLSAKL

>CcapObp33

MKFLIVVLAVVALAYADDEWTVKQAAEIKEIRQECLKENPLGDEYIQKMKSFEYPDEEPVRKYLLCTAKKLGIFCVHEGYHVDRIAKQFKMDLDEAEVVAIAEGCVDKNEQGSSADVWAYRGHKCLMASKIGDRVKSYIKKNVEEAKKQ

>CcapObp34

MKYFILILAAVVLAQAQDDWKIKSANEVNDIRRECHKEHPFNEELQKHEEVLRFPDEDVVRNYEVCVFTKWGVFDPETGFKKDRLVRQFEPVLKREEIDEIIGRCADKNEQGSPVDVWVYRFQQCVSRSEIPPNFLKIIGKL

>CcapObp35A

MKYFILLLAAVLLAQAEGEWKVKTVDEFNDMRRDCHKEIPFSEELQKKEEVLRFPDEEVVRKYLLCIAKTWEVFDEENETGFKKDRLVRQFEPVLKREEIDEIIGRCADKNEQGSPVDVWVYRFQQCVSRSEIPPNFLKIIGKL

>CcapObp35B

MKYFILLLAAVLLAQAEGEWKVKTVDEFNDMRRDCHKEIPFSEELQKKEEVLRFPDEEVVRKYLLCIAKTWEVFDEENGFNMDRLIVEFELLLNRDEVLPIAKRCVDKNEQGSSPDVWVYRVQQCVGKTKLGPLFLEALGKLSK

>CcapObp36

MKYFIVILAAVVLAQAADDDWVPKTPEEFNAIRRECHKEFPFSKELQKQEDELDFSDDETVRKYEVCVFRKWGIIDADDTFHGERLVKQFEAVLDGVEGIEQKVNNCVDKNEQGSPIDVYASRIQQCIDKTDIAPKLLKVIGKL

>CcapObp37A

MKYFIVILAAVVLAQAADDDWVPKTPEEFNAIRRECHKEFPFSKELQKQEDNLDFSDDETVRKYEVCVFRKWGIIDAEDNFHGERLVKQFDAVLDEVENIEQKVNNCVDKNEQGSPIDVYASRIQRCIDKTDIAPNLLKVIGKL

>CcapObp37B

MKYFIVILAAVVLAQAADDDYKLKTPEEFNAMRRECHREFPFSKELQNQEDNLDFSDDETVRKYEVCFYRKLGILDADNNFNGERLVKQFEAVLDVEGIEQKVNNCVDKNEQGRPVDEYVSRIQRCIDKTDIAPNLLKVIGKL

>CcapObp38

MKYFIVILAAVVLAQAADDDYKLKTPEEFNAMRRECHREFPFSKELQNQEDNLDFSDDETVRKYEVCFYRKLGILDADNNFNGDRLVKQFEAVLDVEGIEQKVNNCVDKNEQGRPVDEYVSRIQRCIDKTDIAPNLLKVIGKL

**Plus-C OBP subfamily**

>CcapObp39

MKCVVSLLLMLVLLGVNAYDFDDSAFNEYLFKELQSLYDDDDVSHVHTQRTRREAIDSKECDKRSWKKDMHCCKDSNANGEQLALFRSVKKECIAELKGEPADDAFNPFDCEKMKQVKEQMICISECIAKKLKSLDEEGELKRDVILEGLRAQIGDAQWKIDAVEGYVDTCLAVVKEKREQREKDGESTEGCSRSPLTFHGCMWRQFWKGCPAELRVDSPKCNKLRERVTNGDVSFFGKHFLNKYYPGPRDED

>CcapObp40

MKICQFLGLLLTLEFFAITLSADTDVGTLDESTCSKTRTECCGEINMLLDEPTAKCFVLHENNIPKEDDKDPIKLFGFISCLVECIYTKRHYIGKNSETLNMKAVKADAENLYAARPKEKQYHVELYEFCRKEAFKLYTLIKASPAGLVIFKNSCKPYFVFVYLCQIEYHKKKTCPYFMWQGNEDDETMLSQCKAAKEQCYKIDGLTPPEQK

>CcapObp41

MGNLAIIIFAFGLALKPTSSLKIDCDNPDSIQEDHIHYCCKHPDGYQEFIDACVKETGFQYGKPEEEAMVDITVDRAITGTCFGKCVFSKLEFLKDNELDMSAVRKHFEDKFKTDPEYAREMINAFDHCHDKSVEHTTKFLSNPLFRATNGEFCDPRSSVILACVIREFFHNCPADRWSKTEECSTVLEFSKKCKDALTTI

>CcapObp42

MKVRIMLQWKFCVFFLLLAVFGFGRSKAVEIDCQRPPRLVDPAQCCKDGGRDQIVEACAQRMGLASQRGNGPPTVEAATCFAECILKQLQYMQEPEKINYEAMRKHLQMKFSNDTIYVNTMLQAYQKCEPSVQQKMQILKQTPLGGGAAALRRGCSPFSGMLLGCTYMEYFKNCPAHRWNENEQCALAKQFVTKCSFNA

>CcapObp43

MTVTYILTVLSACLIANTQAANFNCSEPPNLGHFDVHSCCRMPEIHLGEAPAKCSAHIKTLTAQTKSQMGGMKSDGQAQDGMNTANDNQNNNSHSEVEYPAYAHICYPECIYRETGSLVENEFNMENVKKFLNKSVNKRDKDIIPQIVRSFESCLDNIKSHMAAVGIKTYAKLPMGCSPLASMMYSCVNAETFLHCPDKMWKNDEHCNVAKSFAAQCNPLPHVPLPIG

>CcapObp44

MKASLVYFAVIFAFAGHVKADDANNATVDCTKPPRFVPLHLCCPVPDLNTEELMEQCADYAKLGPPPPPPPMGNRGPPPRGGPHRHGPHHHPCLTECIFNQTGVLDATRELNVDKFSELLDTAVKDNEEMAAIMEESFETCSAKAAEFKVKIAEKAKDPGFAERMAQHKSLNCSPLAAMIMACTSMETFKNCPTSSWNDSVECNTARDFVKACKRDGTPRI

**Dimer OBP subfamily**

>CcapObp45

MKFRKASRVFLLITLSICIQMVNSATTTRAAATAGKSTHTDAEILRKCLRQVGSIDLASELQKVQRYSKWTKEEIPCFTRCLATEKRWFDADASKWHKQQIADDLGADMFNYCRYELDRYNEDGCEFAYTGLRCLKQAEFYLPQTLKNILSCASELNVTMNELKKYAGFPEKEVVPCLFQCLAKKLNFYTSDYEWNFDNWIRAFGPMRQDRLASIVCKVSAEHIATRDKCEWMYEEYNCLERLNYNTDGSYPLTETTTLSAALASKNSKTMEVVNTS

>CcapObp46

MEYICIFLLAGALLATLAAAVVQPNVQEGLIIADCLKNYGGLTEDKAQRLVRFKDWSDHYEEIPCFTKCYIQNMFEMFDESEGFKEEQVIKQFGQPLYKACKHRMVPAADTCQQAYNGFHCIVSLEDDPFVLIESMKNVSTEAKTAMKDCLHRYDRYEWEHMKDYAANPVREPIPCFTKCFVEHLQVFNQKTRQWNIPLLRAKLGVPAVGADIKHCLERRRNRNVCGWMYQDFTCFGLASV

**The Odorant receptor (OR) family**

The odorant receptor (OR) family of seven-transmembrane proteins mediates most of insect olfaction, together with a number of gustatory receptors (GRs)  [3, 13] and ionotropic receptors (IRs)  [14-16]. The medfly odorant receptor gene repertoire consists of 76 genes (Table S4), including OrCo, compared to 60 in *D. melanogaster*  [17, 18] and 85 in *M. domestica*  [10]. The medfly OR co-receptor, CcapOrCo was, as expected, highly conserved sharing 88% and 90% amino acid identity with DmelOr83a/OrCo and MdomOrCo, respectively. Two pseudogenes were identified (CcapOr54 and CcapOr62), while CcapOr51 was truncated at the C-terminus due to a gap in the genome sequence. No CcapOr genes with alternative splicing were identified.

There are 10 cases of simple 1:1:1 orthologous relationship across all three species, i.e. DmelOr82a/MdomOr70/CcapOr6, DmelOr24a/MdomOr16/CcapOr10, DmelOr10a/MdomOr11/CcapOr11, DmelOr22c/MdomOr15/CcapOr12, DmelOr13a/MdomOr12/CcapOr7, Dmel88a/MdomOr79/CcapOr64, DmelOr85e/Mdom78/CcapOr54, DmelOr83b/MdomOrCo/CcapOrCo, DmelOr43a/Mdom23/CcapOr14, DmelOr49b/MdomOr42/CcapOr13 (Figure 5).

There are numerous examples of gene duplication in one or more of the species lineages, as well as large gene line expansions and losses, all examples of the birth-and-death mode of evolution of these gene families. There are several large species-specific expansions that may reflect major differences in the chemosensory ability of each fly. The most evident of these in the medfly are ten ORs (CcapOr38-47) related to DmelOr7a, a receptor for numerous fruit odors  [19] and, surprisingly, Bombykol  [20]. There is also an expansion (CcapOr58-62) that is almost equidistant from the DmelOr67d and DmelOr83c lineages. In *Drosophila* these two ORs have, apparently, very different functions, DmelOr67d is involved in the perception of the male-produced sex pheromone [Z]-11-octadecenyl acetate  [21], whereas DmelOr83c is specific for farnesol, a component of citrus peel  [22]. Incidentally, farnesol is also a major component of the medfly male pheromone  [23, 24]. An extensive expansion of the DmelOr67d lineage is present in *M. domestica* (MdomOr53-65) suggesting that the house fly, and perhaps the medfly may have more complex pheromone repertoires than *D. melanogaster*  [24]. Like *D. melanogaster*, the medfly has only one ortholog of DmelOr45a that has been shown to mediate repulsion from harmful compounds in larvae  [25]. *Musca. domestica*, however has a large expansion of MdomOr24-33 related to DmelOr45a, perhaps associated with the diverse and potentially toxic food resources and larval habitats of the house fly.

**76 CcapOr proteins in FASTA format.**

>CcapOrCo

MQPSKYVGLVADLMPNIRLMKYSGLFMHNFTGGSGLFKKIYSSMHLVLVLVQFLLILVNLALNAEEVNELSGNTITVLFFTHCITKFIYLAVTQKQFYRTLNIWNQVNSHPLFAESDARYHSIALAKMRKLFTLVMLTTVVSAVAWTTITFFGESVKFAFDKDTNSSITVEIPRLPIKSFYPWNAGSGMFYIISFAFQCYYLLFSMVHSNLCDVLFCSWLIFACEQLQHLKGIMKPLMELSASLDTYRPNSAALFRSLSANSKSELINNEEKEPTDLDVSGIYSSKADWGAQFRAPSTLQTFNGMNGTNPNGLTRKQEMMVRSAIKYWVERHKHVVRLVAAIGDTYGGALLLHMLTSTIMLTLLAYQATKITGVNVYAFTTVGYLCYALAQVFHFCIFGNRLIEESSSVMEAAYSCHWYDGSEEAKTFVQIVCQQCQKAMSISGAKFFTVSLDLFASVLGAVVTYFMVLVQLK

>CcapOr1

MDKLEALSSRIFPSDPSIGKIGSIEYNVWLAQLFGVPVLGLKKETPRMRIALAVYGVVATLVVTFLYTGFEIYDMIFCWPNLDKLTQNICLSLTHVAGALKVINIIYRLKEVAGVVRKIEYAARYYVISKNQLKAFYRGEFENKIPLTIYASLVGFTGILGIAYLLHNPTGVAGEIFPYRVKLPHWMPFGLQLAYMGFSVLVFALQIVAIDYLNVTMINQIRFQLKILNLAFEELKFVSGQAAHELSLDRRLRTIVDHHNLLRNLRNEVEEIFRLPVLVQFFTSLIIFAMTGFQAIVKSENSNGASLIYCYCGCIFCELFVYCWFGNEVSEQSKTLTTSGYNCHWYQFGPRYKKSLLIFMFNSQKPIVFTAGGFMALSLPSFTGILSKSYTVIALLRQFYGR

>CcapOr2

MHRLLCKFEQVAMRVFPSDVRRGQIGSIEFNIWLSQMTGVPLPPSFVPLRRRFVSNCLLLLCGSFLCFVHFMYTIFEFYDLYIVWPDFYAFTQNFCLTMSHFAGALKIINLLCRLKTVQRAILKLKQATKTYIRTDAQRQIFDRAEVENKLFLVIYATVIGFTGFTAMILVFINPDMAGKIFPYRVALPAWLPLPVRVAYIGTTDFMFAVQIVTVDYLNIGMMNLLRCHLNVMKSSFDELNFNVKCMKSDIKRIRDPNERLADIVRHHCVLKSVRDDVEQIFRLPVLLQFFTSLVISAVTGFQATIYSSNFKSELIIYFYCFCIFTQLFGYCWFGNEVNEQNKTLAARGYSSSWYYFDNRFRKSLAIFLVNAQQPFDFTGGGFVALSLPSFTGIMSKAYSFIAVLRQMYDR

>CcapOr3

MRIIVLKDITPVVDAFACIGFNIDLNKAKGSFSQPVRYFVLLIGVIAWTAALAAYTMQYLTDVDKMVAAMTINVQLFLTTSKNLIFLARRKRFLHLNEALERLALNGNESERILWNTTNRFVLPITRAYRISSELTVSFCVLLPILKLLYYYIFHSEVVLTLPLPGIFPYNYTLPFYFILTTILTILLVYLCAYTIVAIDGLFGWFIYNISAHLQIMSLRLEQILQLPIEDPRFHRHFVDLVNYHKEIIRLSLELDAVYAPIIFLEVTSSSLPICFLAYQLSYLSDPANVPFMCLLMSSIVIQLMIYCFGGEKVQSECDQLCENIYLLIPWQNLPQKHCRLLLNPLIRSQRVLVLTGYFFTANRSLLVWIFRTAGSFTAMLFALKEKDV

>CcapOr4

MSKYLRIQYFSFRMIGINLWAKRDQRIMSAPYRYCIWSMATAIITILMGFFIYSNEQDQAITVLTVFLQGVLSVFKSGMFVLKSGRFIELIRNLDMLAEKEHSAWRLENDWQQRIVSVYYFCCTSTGILYCTVPALVLLYTRYFREQTVFILPFEASFPYDTNQPLFYILSYIWCISFIIYAIHAIVAMDSLFCWFIFNISAHFRILQQKLTDVSVSPNASTDHAIFQQDITQTLFYHQRIIELSAEFDELYAPIVFVEISVSYLKLCFSAYNLINLDDISSLPVIAVGLVTITFQLCIYCFSGEKIKNASEQFANHIYLSFPWERVPPSLRRLLFVPIMRAQRPMHLTGFLFIVDHSLLVWIFKTTGSIIGFLSATKKENTNI

>CcapOr5

MFHSDLVINGYFQLQKHTFQRLGIDMTTRNASITHIYFLALQIVALVTITMPMVIYSCQHLQEIAEVTNAMAPFMQATITLWKIWRVIYRRKMMAELVEDIYSVSTKATKQELTHLRRENNRERIMNTFYYYSVFNTGVLALIAPVLVSFIQYLRLGEFSYIVVLKATYPIELARPLNYVLIWLWSAIAIYGVIYGSVSVDSLYSWYIHNLVGNFKILQTKFVTAELIAEVSERRASIYYCLGYHQRLITMSEQLNIIYQPIVLVQFSLNALQICFLAYQIGSGDVAVVDLPFLFLFMTSVGIQLMIYCYGGQYLQNESENVAKFISQTINSTIWPIDLRKVLLFSMARAQRPCKLTGIFFDVDLRLFLWVWRTAGSYVTLLRSVDQQSS

>CcapOr6

MPEDLFRIQRNCLRLMGHQDIYDDDGDDNNSSNNNDTEFSSVERKSLRQRLCFRHLQTMKYVLLLLLMMSAQLPMMDYIIYHIDDLELATACLSIVFTNILTVIKTSTFLAYKREFKSLMTEFELMYDELRGAGAKPLLVTVNVGAKRFVKLYFYSVSCTGLYFTIKPLIGMFWAKFQEKPLLLELPMPMRFPFDFESTPGYQIAYVYTILITIVVVMHATSVDGLFVSFTTNLRGHFQALQYFIETNTFNKSEARLQKELRFYVEYHVRLLGLAQSVQRIFKPIIFGQFLMTSLQVCVIIYQLVMNMGVIMEMVIYCTFLSSILLQLLIYCYGAEFLKIESSAVGTAVQMSQWYNLPPRHRHVLRLMMVRSQREIIISAGFYEASLANFMSILKAAMSYITFIQSIE

>CcapOr7

MLFNPKPLKDPVNFKFPLQCIWLKLNGSWPVNSKTSSSFKNYCRFLYSLWAWYVVAMVGITIGFQSAFLAKSFGDIMVTSENGCTTFMGVLNFVRLLHLRLHQRDFHQLIAQFVKDIWITKSTHPAVEQSCARTMRVFQVISVLQSCLITMYCILPLVELYMLNANLDQESLAHIDKPFPYKMLFPYDANHGWRYALTYLFTAWAGVCVVTTLFAEDSLFGFFVTYTCGQFRILHTQIDNIIPAAYAATRAGRGTEADYQRECVRRLDKIAGKHSILFNFVSRMEEFFSPIFLVNFLISTVLICMVGFQLVTGNNMFIGDYVKFLVYILSSLSQLFVLCWNGDKIIQNSLEMANHLYACNWESDIVLTNTHTNNNQQMKNPTRIIYYSTGAVFRKNLQFMIMRSQRQTCITAMKFSILSLSSFSGLMSSSMSYFALLQSFYEDEEN

>CcapOr8

MRYLPSSYHKPLLPNGRHPPIDWQLYGFVCSNCWPLARDITKTRRIIDMVITAMQFMSESMVLSGETIAMRNNLDDISFVCMVLAPYLILIELMLRAYNIIYKRNSFRTHIEEFYKKIYVQRTWNPELFEQIRRQHLPTKYSTFTYIITLVTYVYVPISGLVKNERLVPFPIQFSFDFTVPWPRYVVFLIMSIWTGFAVVGPLVSEANMLAMQILHLNGRYSLLLRDLRKIAREAIEEHEKCKGRDKISVTQRFRYSLFDIIRRNVELNEFAKSLQDQYSFRVFVMMALSATLLCVLGFLTATLGLTAENIRFVSWIIGKVVELLIFGRLGTTLSTTTNDLSTSYYCCDWEEIIFHSADAEENRKTMKLIALAIHLNSNPFQLTGLNFFVVNYETVVSILRGAGSYFTVIYAYR

>CcapOr9

MLYRPRLPNGKLIPLSWPLAAYRLLNNICWPLRDNANRLERLFDRFCWALGFFIFIQHNDAELRYILSNNNNLDQMLICGPTYLILVEAHLRAFQLGLKKNNFKNFLKRYYAEIYIDKPTHPKLYANIQKRLRPIWFYSFLYYSTLFSYVITPLTNYLKNVKAPLFKMYYPFDITPNPIYVAVVLSNIWVGFTVISLVAGEDNILSEVMLHLNGRFLLLQQKLRQNADRLLHNTDGRYIADALQDQIIEAIEENVRLYEFAKGFEREFSFRIFVNLSFSAGLLCVLCFKVYTNPMASYGFMFWICAKIMEMILVGQLGSTMIYTTNEVSSTFYECNWELVLMKSTDTKANVRLLKTLALAISTSGKPFVLTGFNYFSVSLTAVLKILQGAGSYFTFLTSMR

>CcapOr10

MSIKFLTQSYPTEKSLFLIPKFVLRIVGFYPEQEKSTIRRNAWTMFNLIMLIYGSYAEFMYGVHYLSIDAVRALDALCPVASSIMSVVKLSFLWWHRVELERLIRRVSVLTAEQDSRLKNNYKRRYFTIATRFSAALLCLGTCTSTLYTIRAALANYFSYVRGENVPYETPFKMIFPQTLLSKWIFPVTFTFSHWHGYITVAGFTGTDGLFLCFCMYFGTLLKALQIDLKDLLKDMDCGQHEGLSERDIEECMKKTVMRHNEIIDLIGDFSAVMSSITLTQFVLSSVIIGTSVVDMLLFSDYGILLYFVHALAVTTELFLYGIGGTTVIECSSQLATAVYDSNWYSHNVEVKKMVLFMILRTQRSLVIKVPFFAPSLPALTSILRFTGSLIALVKSVV

>CcapOr11

MKFKFLSRTFPLRDYYFYVPQLCLGSMGFWPMDTCRQQAANVGAWMNLIILAIGVFTEIHAGCTVLRTDLELALDTLCPAGTSAVTLLKMTLIYYYRQDLAWVLERMRSLVYERDGERIIRAHAVMAARLNFIPFVMGFITCTSYNLKPLLITLILYMQGQQPMWKLPFNMTMPAFLLRAPYFPFTYIFTAYTGYITIFMYGGCDAFYFEFCSNAAALLKLLQEDLKSIVSFEEQLVFTAQESTLLEWRLVRFIMRHNDIIELTRFFCKRYTIITLAHFVSAGLVIGASIFDLMTFTGFGIVIYIAYTIAVLGQLFIYCYGGSLVAESSVQLATVAFGCDWYACNPKLRRYVLMIIIRSQRAISMSVPFFSPSLITFTSILQTSGSIIALASSFK

>CcapOr12

MLQPLLGSQVPIEQSFFRIPRISARIAGFWPQPAVRPRTWLTVLRFCVNTFAVAVGGFGEVTYGFFYLYDLFSALEAFCPGVTKVISLLKMTIFFGRHERWQRVIHGLHTLLLLDTSAGKRRIMEPLASFASVLSFVLLASGSLTNTFFNVLPLLKMAYFKWRALDMQLLLPFNVILPEVLVNLPYYPATYLVLTLSGAMTVFTFSAVDGFFLCACVYATALFRILQHDIRNAFAELQEQESSSFEQNMRIQHRLSVLVERHNKIIDLCSDFAAEFSLIILMHFLSAALVLCFSILDLMLNSASIGVLIYIFYSIAALTQLVLYCIGGTYVSESSLSIAEVIYDVDWYKCDVRTRRMLLLMMCRAQRAKTIAVPFFTPSLPAFRSIVSTAGSYITLLKTFI

>CcapOr13

MFDDLQLIQMSVRILRFWSLIYEHTWRRYACLSMTTFLVFTQFYYMFRTSEGIDSIIRNSYMLVLWFNTILRAYLLLYDREKYEELLRDLENFYYDLKKSKDFYIQDLLNEVNSTGKYMARGNLFLGLLTCFGFAFYPLFATERVLPFGSMIPGVDEYKSPFYEFWYIYQMIVTPMGCCMYIPYTSLIVAFIMFGIVMCKALQFRLKTLHRCRHVDGLIHKNVKECIRYQLSIIDYIARVNGFTTYIFLLEFLAFGTLLCALLFLLIIVDSSAQAIIVCAYIAMIFAQILSLYWYANELREQNLAIAAAAYDTEWFTFPIPVQKYILLMILRAQKPPAIMVGNTHPITLELFQSLLNASYTYFTLLKRVYI

>CcapOr14

MPITRIEDNPLLAINVRLWKFLSVLFARNWLRCAAFVAPVCLMNAMQFVYLYQQWGDLATFILNTFFATSIFNALLRTCLVIKNRDKFEALIQELVTMYDDIEATGNDYAKRELAAATKAARKISIFNLSASFCDIIAATLFPLFQENRIHPFGVALPGIDVTRSPLYEIVYISQLPCPFTLTSMYMPYVSLFASFAMFGKVALKVLQDNLRNLCDNMQHKSEMELFNLLRSNISYHARISKYVNDSNELVTYMVLIEFMLFSCVICSLLFCINITNSTAEKISIVMYIGTMLYVLFTYYWQANGILEQSLLVSDAAYEMQWYKCSQRFKRTLLIFIGRTQKPLQIRVGQMSPMTMEVFQSLLNSSYSYFTLLHNLYND

>CcapOr15

MKKLNFVTKLFGWNIKLWQRERFLDEHKRWIAYIALFVPAIMIPPIFCNLYLGHDDLGEAIYDFFTAMIDITGLIRSIVILRKQRKFLNLFGVVESWYEDLKQPNEHKALETLNQIVAKVQLYSKCCLYSLLMVDVTYAFEPIITHYGKLVVELQLPSIDLHQSPVYEMVYLIQALWLVPLTSVNYVSYSNSLLIFTIFGVFATRHLQQKLMEISQMEDDEALANLKQCVVYHSKIIKFGENLEELYSLMSLLDISLYCISVCLMLVYLTMDFTWPLLFKGVIVILFLTTLIFLTYHVADVLTHESMNIAELAYNTNWMDRDKEFRSCIQVIIVRSQRPLMLTAGGFQPMNMKTFLAIMRASYSFFSVLRSTV

>CcapOr16

MATERKSMPNVERLFNWNLEAWTRLGYLDKRKRLLACTIISAPIVVSMFVMPTFFMGVNTFEQHIYNFYMIIVTSSSVARAILIIIKQRKILDLLNDMENWFVEVQEQNDNNALETLNKLTQKVRRYSKYTLWWVIIFGAFLSFQPICTGYGKFVYDTQIPGIDLHQSPLYEIMYGFQSLWVIPMACVSSISYADTLLIFISFGIFATKQLQRKLKDISQMDEQQGLENIKKCVQYHWKIIKFGEDLENAYSLMCLLDFSLYCVTLCMLLFYSVMDFTWALMFQAVVVELILTLLIFLTTFLADIFTQESLNVAQTAYDMNWLQRDKAFRVAVLLIILRSQRPLILTAGGIQPLNLETFLAIMRSSYSFFSVLRGVM

>CcapOr17

MLYSEEIYNWNLVFMRSIGYLGNRRRAYFLLSLPVLICFGAIYGTYKMWSDFDKVIINLFKTSGLLTVTLRSFVIIQKEKKLYDFFDYISQLYRELQAEGDETTLKRMHEFARKTKRYTKGLFILMIACTFYISLIQVMSTFGIGLKKFLIEMELPFISVNENPYWDIFSTLQAVWLAPSILLSYISYLCIIFTTISFGILLMKDLQFKLGNMNEKNDLEAYEYIKNCVKQHVMIIKYHRQMEVLFSLGSCAEVCNFCIIPCVIIVYSTMDYDLAFLMTDIQLAVIAVSSTFFNFWLANNFCVESLNIAYAAYNSNWIDRNKEFRKYIVLIMTMSQKPLQLTAAGLKPINMEFFLAILRAAYSLFTVLQ

>CcapOr18

MVIEKPTESNTLIKHAYQNLPLYSANVKLLIKWGYIGTPSRLQRFLLGLLPVLTLIGQVINIFKSPDADMGETGMNFFLLAIMTNSILKHFTITRKDEYFQRFLQSMQQWCNAMELYEDQRIPGLILDITRRSQKLSRITFYGSVVGTVCGVAYPFTFEHRKFIFDVQYPLFDIKRTPFYEINFLLQAFVLVPSFLCVYMTFTNLLFTFLMFGEVTLLDLRLKLQNISKDDQTKMLKDFKDCIAYHNQIIDFRDDLENLVSMALFFEVALFGLMFCMLLFFISLVHDYQLIFTAVTFIAVTLYMIAVSFYFASKFTSESLEIANAAYDTPWYDGNLEMRKCVLTMIARSQRPLLITAGGIYPMTMENFQAILRVSYSYFSMLQGLN

>CcapOr19

MITPRKASSIPIIKYTRITMEEPKKIVNCFYERQFKFFKFLGLFGLPPNYSRFCQILFKLYFWHVTVVWMLLFDISMWVKVIGNITDLNEIVNVFYICSMAIAVMAKFVHIRKKNSRYVAFFARMHNDDLLPANPSELKKFIKSVHLSCAVRNCYMGLSLTSLALVFVPKLISDPGELPLSIYIPLNVEHTLCFLVAYIFQFVGLSLCCFLNIAFDSLSASFFIYLKGQLDILSNRLENIGKFQNITQDVITLQLKECIRYYAKLRYITDIMEDLLCIPMSVQVISSVLVLVANFYAMTFLTDPSDYGTFMKFLVYQLCMLSQIFMLCYFANEVSLRSAQLSYALYSSEWTHCNQINRRLMMLMMAQFDVPIRIKTINRCYSFNLPAFTSIINSSYSYYALLKNMKD

>CcapOr20

MNNSRLLITMLFILGLWSTTRIPDSWFKPYYRCYKSFVNSTLVYSFILLQFLEFILNENMENADEDLKLLITEICYLAKSLNLFYHLELAVELLHEWEISDSFNLRRSAEREMWLQGERAFRKVIYIYIFFCLLTVFLAIGSTTLSGTPTLLYQFWLPAKWREYNIWPAFFYESLSLNFNCMCNVVLDSFQCYLLYHLSLYTRLIGMRMERLGYEKTNVETTSSLENIIDMHQRLKAMTRCCEKILSVPLLTQITLSAFTICFSIYSLRSLSFIEHPMSLLSAVMYLLCMSIQIFMPCYFANNLTAESQNLSSHLYNCNWVDLSVYNRRQIFLYMEYLKLPLFVYAGNYFKVGLLVFTKIMNNAYSLVALLNNINNDDQ

>CcapOr21

MTFDNIANSRFLTRALVLLGLWPVITVHGSWLQRNYCFYQLFLQITFTFSFTFLMLLEVICSESLDHATEVLKFLLTEMALVFKILNTWYYARKAAAFLHEWETGEMFVLRSNAEKNMWAKKQSTFRKIMLGYIYWSISSAVCALLSCLFINDQALPFPYWTPNHWLEDYYWLMYFYELLTMPFTCLCNIEIDVFQCYLLLHLALCLRVVGMRLERLANAGDENAITREFLKTIKMHRRINDMARNCEQIISLPVTIQIMLSALIICFIIYRMQSVHFSDNPTEYLAMFQYVVAMSMQIFLPCYYANELTVQSQNLSKSLYNADWTGMSAYNRRLMLLYMQYLKLPLVLYAGSFFQIGLPIFSKTMNNAYSLLALLLNVSDDKDKK

>CcapOr22

MDLQEYDNNSGGRRIIQVMKLLGLWYYEGSAKMPYLLYSCLLHFTISIPFTIFMAMDVVHATDLEKFTNIMYLTLTELGMVAKLFNVWFYAKLLVDFFETLSGDKYFELREKDERLKWQHAQRTYARIVLFYVFIGLGAMFTGFVGVLFSAKYELPFPYAPPFNWHTPHGYWCAYLYELLAMLITFFANYGFDMIQCYMLLQLSLCFKLICGRLECMGELRSGTAVSRGFSEQQLYRQFVDIVKLHARIKNLSRLCQTYISFPFLIQIMCSSFVLCFSAYRLQKLSILSDPMQFLTFVQVNLIMILEIFLPCYYGNEVIAQSSALNNATYNSEWFRCSPGLRKYLVIYMAMLQRPLRVRAADFFDISLEIFTNTMKNTYSLMALLLNMN

>CcapOr23

MEYDNINGARRVIRVLQYIGLWRFDTPWQPLYATYSYILHITCTFAYTIMMWLDVVQASDLEKFTYIMYMSLTELALLTKVANVWRYSKLFVNFFHTLADDDVFRLRNAVERTLWQTVHRYYGWIALMYFTMSITLVTSAFVGVLFSAEYELPFPYAPPFEWRNERGYWYAYFYELIAMPVTCFSNCALDMIQCYMLLHLSLCYKMIGLRLEAMGKWQEQLRCSSCDFSELKFLDEFVDIAKLHGRTKKLTEDCETFVSFPFLIQIMCSSFVLCFSAYRLQKVSIFENPSQFCTLILAVIVMILQIFVPCYCGNEIILSSGALNNAIYHAEWLQCSPKVRKYLIIYMEMLQVSIKVRAGSFFEIGLPVFVKTMNNTYSLMALLLNMNK

>CcapOr24

MATAIELQERIGAARVLMRMLQYLGLWPISQTGLQRTRHTQCRARLAHYYRYLLHLPLTFTYNTFMWIEALTRWERADHILYISITEVGMMALTLNFWRMERRAWQFMQEISHDSGLTLCNEVERIWWRKQQRTFTLIVICYIGGGAGVLFTAFGATLLMSGYELPYDYWLPFEWHNARNYWYAYGYELVAMSLTCIANVTMDMMMCYYLFHVALLYKLIGMRLIALQYLSEPFAVQKLRNIIELHKKVKRLTVQCESLVSLPILVQILLSAFILCLSAYRLQNMQINENPGQFLAMLQFASVLTLQIFLPCYFANEITINSDALMTCVYSSNWEGFSPATRKQMYLYMELLKKPICIKAGNFFMVGLPVFTKTMNNAYSLLALLLNMSK

>CcapOr25

MTKNSRSRILSPNSRISSGRIIVYVLQAIGLWQWAEDRRQYERNRNLQRLQRLYGLLLHLPLTFTFILLMLTAALLAHDLEETSGVLYMLLTELALVVKILNIWRQGSSAWLYMDELAHEVLYSLRQESERLQWQSELRSFDIIAYSYTLTSIGVVVFACVGVLLTATDIYVLPFDYYVPFEWRHPRNYWYAWSYCSVSMMMTCISNVMLDMIFCYFMFHLSLLYKLIGWRLAALRRSRADNGVGGRGDEDGVHREMREIFQLHMNVKRLTTQCESLVSLPVLAQIILSAFILCFSGYRLQRMHIMENIGIFLSTIQFVSVMTLQIFLPCYYGNAVTANSNALTNDIFNSDWTGFDMRSRKFMIFYMELLKRPATLKAGGFFQVGLPIFAKTMNNAYSFFALLLNMNN

>CcapOr26

MSVKVNSWEAFKYHWRVWELSGFLGPQKGSFWHVPYKLYTIVITLLFPIYYPICFTVESILTDNLNDFCEVIYIAMAVVTLNIKFFTLFMVRNQLLQLQPILKRLDGYAQTDEEIHVLLEGSKSAKKCFLTILKLFYSAFVTSQLMVIFSSETRLMYPAWYPFDYKASRINFWIAYGYQSVGFLVQCTQACSVDTYPQAYMRILIAHMQALSLRIERIGLNANDNTSTSLKYLTKEEMKRNYYELVSCIKDHKTIIELFSTIQKPISGTSMAQFVCTGVAQCTIGVYMLYVGFNLSIMLNMAVFFISVTMETLILCYYGDLFCQECEKLSKAIYNCNWTIQSSEFKRALCIFLLRSQRVNVLMAGNWIPVKLPTFVMVVKSSYSIFTLLSNFK

>CcapOr27

MQIPPPTPSSSLAATVTTTTVDSSHFFRMPVAISWLCTPAALFNSTNLNNILQMLPMPAAALACSVKYISYYRRLDLVRQVEQIFNALHERVQLAEDRDFYAGIQRRANLIMKTLRAVSFFFFLITVMSFVSSIEERSLAFIVELPFNWRASTKAYMCAVLLELLLLTCDLLQSLVNDSFPPTALCVLSNYTRRLGERLARIGYGGSKDVHGSIAELRRCIVDHQHLYSSLNAIIEDIISVPVFVRYAVTAFQDCFTLVTFIFYTDTTSDKVLYFTYLLALQLQIFPTCGSDCAQSSADLLQRVYASNWMEQTRAYHRLMRVFSPRSMKSTTTYAVGSIPIHLGIFVRTLQGAYSCYKFVNGMRKL

>CcapOr28

MSSPLISSSSSSPSPSPSPSPSPSPLPFPLPQRLAATAVDTRSFFKLHWACFKVLGVVAPTADVFYLVYSVLLHLLVNLCYPLHLALMLFRSPNSSANIQNLAVCVTCMACSVKFVIYTMKMWRIRELESIVAALDARACSPRERGYFLKLRKDMRRITIGFLSIYAFVGVTAELMFIFCNEHNLLYPAWFPFDWRASKLKFYAAHFYQIVGISYLLLQNFVNDCFPTMALALLSAHIKLLGIRVSQIGHEAKSLDANELELLRCIKDQEHFMLNTIQNIISLPMFLQFTVTAINICLAMAALFFFVDAPFDRLYYLAYFLSMPLEIFPTCYYGTDFQLLFETLHIEMYASNWVEQTQKFRKHMILFNERSLKKEVAMAGGMIRIHLDTFVSTCKGAYSLLAVIMKMNE

>CcapOr29

MQQPIDSSVFFKIHWLGFRICGGDSSVRKYRLLDYIYAFAISTLVTICYPLHLALALFRNNSLASDIKNLAVCVTCVACSLKFVIYTRKLRLVHKIEQTFAELDARVCSEVERKHFDRMRSSVKNIFYLFVCAYTAVGVTAELAFLMSEERGLLYPAWFPFDWRTSARNYSVANVYQIVGISYQIFQNFIDDSFPPITCCLLSGHIKLLGIRVSRIGYECADVLENERELVRCIKDQKNLYRLFDLLQAVMSLPMLIQFTVTAFNICVAMVVLLFYVDTPFERAYYFIYFISMPLEIFPICYYGSSLQLLFGQLQYEVFRCNWMDQTPRFKKHMILFTERALKVIIALAGGMIKIHLDTFFATVKGAYSLFAVIMKVR

>CcapOr30

MLKNVFIFKPAGNAAVDSVACFDIFWMCWKLNGIAVNSNKWYITLYDIAVNIFITIFYPIHLTVGLFMVPTLADVFKNLAINITDVACSTKHYLFRYKLPKIRELQRLLKQLDERVLAPNEREYFDKKIRLGVRNIMLLFCASYAADALASAIDVLSKNERELMYPAWFPFDWSANRFTYYGAVFYQIFGVSLQIVQNLAHDTFAPVGLCVISGQVRLLAMRVSKVGYDESKSLAQNEQELNECIEDHKKLLRIFDLMQDVFWYTQLVQFSSVGLNICLTVVFLLLFVDNLFGYVYYTVYFISMAIELLPACYYGSNMQEEFQNLPYAIFKCNWIPQRRGFQQNLRIFTELSHKQLTPTAGGIINIHLTSFVATCKMAYSLYTMLMNI

>CcapOr31

MFQRQIETRAIFRRLFMTWRVLGIILWPFNKYLRIIYDILMNIFITFAFPVHLTLGVIFSSNQEQFFTNLIIGIASVSCTFKHLLWRSRLAEMQQINEILAQLDDRVRVREDYEYYKRSIERLCNFMINFFTRCYFSVGVTALFIALITGELLYPAFMPLQWRTSFWNYVAAILFQFVGVMLQIVQNIANDVYGPVVLCMISGHVHLLANRVSRVGHDTEENTQSNYEELSKCIEDHKLLMSTSKTVERIASLSYLVQFVAVGINLCIGLVYLLFFADNYFAYVYYTIHITAIMIELFPCCYFGSMLECEFHDLSYAIFSSNWPTQPRPFRRNVVSFTEMTLREVTMYAGGMIRINLDSFFATCKMGYSFFTVIQTMK

>CcapOr32

MSKSKVDAVSIFVRLIFFWRVLGFTTNYNKHLVRIYDVFVTIIVTLAFPMHLMLGVIFAKDKETIFINLAIGISSIACTAKHFMLRPSIDKILYVNNILRQLDERVQNVADTDYYVKQMREKAIFMINFFIVVYFSVAIMALLSALWTGKILYPAYVLLDWQSGTWRYLAVMTFQTFGLNMQIVQNLTNDAYGPMVLCMLSGHVHLLSNRVIHIGHDHETDVDDNYAELVRSIEDYKLLMSTTKLVERIISSSYMVQFTAVGVNVVVGLIYLLFFADNLFAYCYYLFHIVAIMIEIFPCCYYGSMVQAEFHALSYAIFRSNWLTQPRKFRRTAITFTELSLKDVTMTAGGMMRIHLDSFFKTCKMGYSIFTVLQSLK

>CcapOr33

MAFEENPESVGSLFRTHWIVWKCLGQVPDPRYPKLFKVYAVLLNVGFGLGYPLHLLLGQLGLQTLEEVLLNLTISVPVAVCALKFFNIWRNLRKVRHLEKMFNTLNTRINQRDEWIYYRKVTIPNALKVLHLFYFICVGTALASELTLLIMGFAYEWRLMYPAYFPFDPYATTGGYVVAHTFQIIGLLVQLAENLVSDTYGGMCLALLAGHAHLLGKRVAIIGYDNQKTEMDTGRELANCIVDHNMLFDCHSILGEIIGIGMFAQIISASLIMGIVVIYMVFYVGNAFEYVYYSIYLFGCAMEVFPTCYYATNFEFEFDKLTFMLFSCNWMDQNQSFKKSLMISIEQSLKTRSFRVGGMFRINLQIFFATCKGAYSVLALALKFK

>CcapOr34

MIQSDKISTIMSATLEETFVTGLSVFHFHDVTWLYLGQMPPSTPLYRYLYYLYSLLLNIIITIGYPTHLMIGLIRSEKQSDVFKNMSINFTCLACSIKTFAFWWRLAEVQKIYAIISKLEKHISELDDFRLYKTIALGRAKSILYFVLFIGLGAAITSEVATIIAGILGDWHLMYPAYFPFDTDRSIFGYTTAHFYQCFGVTAQIFQNLINDTFPPMALAMLAGHVRLLNLRVARVGHASSGGSTKQTHNAQFIECVEDYKDLLEFRIAIQRICSLGTFVQILVTAFNMGVVIFYLIFYVNDIFSYVYYVVFLLAMPLEVFPLCYFGTSAQMEFEQLSYAIFSCNWVAQNTAFKKNLLIFTEQSLRKQIVIAGGMFAVNMDTFFATLKFAYSLFAVVVQMK

>CcapOr35

MCSARLLQINDNGTPLNSAHAFRYLWLNWRLIGMHATRRHRLAYHIYSGFINLTFGILLPATMIAKLFFIENLSQLIGLLYLGVTLTMATAKQYSLWLHRPQLLAVNSYLAKLDARCDRHAVDRQHILTAIRICHLFYFAYMFTYELSSSGFAYMGFSLRQLVYEAWFPQFFADPAKNLTLTLLYQNFAVMTFFVLQNVNNDMYPQCYLAIMIGHLRALAARISRIGKDDGRLSDEENIVELINCIEDHKNLLGYFACIGPVISRTIFMQFGITAFVLCLTAVNYVAFERDTAQMLIAATYIFAVLIEALPCCWYVNSLMEECAQLTTAMYECRWYEQNRKFRKMLIIFMQRSQLTLALMAGNLVPITLQTFVNIIKFSFSMYTILKG

>CcapOr36

MSNKAATAAQSTTPLQSSDAFKHIWLCWRYLGMHPTTRHRHLYLISSLLVHLFTGLLYPSLYLASIFVDIDFSDKLANLSVAMPLYYTAVKQLVMFYYIQTDLPHATKHLQALDRRVEERPEDYVYLRQTVRYGAWSFFAIFLGFWSALISYGLIGLLRHRLPFEGWLPFDWKNSMGAYVGAGLIQLFAVGMLLTNAVCCDSYPLVYLSLLVAHLRILNKRIARLGTSRKATAVEHYQQLAACVEDYRECMSYYQCIRASIGGTIFVQLVSTALSLSTPAVTFISGDFNFSQMLKFLLFSSAVIVEAAPCCWLMDVVLLEMRSLTHSIFSCHWHVQNARFRRSLIIFMQISQKVDPLLAGHIVPVSLDTFTNIIKFAFSLFTLLNQIKSK

>CcapOr37

MTKQEQATPTRLDSGDATRYVWLFWSIIGIHPFKKHRTLYWLYSVLLNFCCSAFFIAFYAVTFFVVSDLLEILANLSVMVPLIYNTAKQLVIFYHIRRTLPQAALHLHALDRRAEQEPAAREQLKRLVQLSHRIFLTALTGIGICLTLYAMGGILRHRLPFDGWLPLDWEHSVGAYVAACAYQLFCLIVQCIAALCNDTYTVIYLLLLATHLRILNARIASLGHGECTEVENYRQLAACVRDHWACMNFYNSIRPAIAATLFIQFFSTAITLCTSAVAFVNAEDSVAQLFKFLPHLLVVVCEILPCCWLMDKAALEMQDLTKSLFACRWYEQNQKFRRSLLIFMQRSQKVEKILAGDLVPVSLETFVNIIKFTFSLFTLLNQFK

>CcapOr38

MRKIGDLCYGRGKNNVYIKESFRLLFFSWSLTGIAPTKTPRLFNTIFMIICWCGILMCPYCFIAGAVNSMKTSVITVTLVNLQAALNGIALPLKAITIAVNVKRLRSIDNIFKELDNSYTDPVHHELIKKSVMRCTRLFVVFLTVYWLYGITSCTAALFSHKYPHSMQIPFIDWLPDSDVKYWLHYILEASYFFFLLLVNLTNDVFPAIYIKAIRTHLYLLTERVSTIGKKSETTAEQNYDTLVECIISHQKLLRISDTVGDVISKSIFFQLAVYSTILCICMLNMLIFADTTYILVTLVYLIPVLSQTIPSCYQASMLEAESTKLSVAIFHTNWWNLDKRCHKLLIYFIQRSQQEMVFTAVKLFQISLKTNLTIAKFSFTLYTFINKMGIGETWKN

>CcapOr39

MRKFSELFYGEGKENFETNESFELLYWNWTFLGTRLVKPYRVRNLMSSAFAWTCLLTSPLFFFVGIAKMVNTSSMTELLTLIQAALNVITLPLKTIVIAYYMKRLSSVKPMFKRLDERYNTPREREQIKESVKYSTRIFAIYFIAYFIYGTMTALLGLTLHSQPLNSWLPFTDWIPMQTLRFWLHFCYEQFTVYLLLLNQVSNDAYACVYIHALRTHINLLAERVSRLGTNSEFDDEQNFKELIDCIAAHQELLEIVKTVANVFSLTVFMQFTVAAAILCVCMLNIFIFADTFHQVVTVIYYMCVSLQTLPTCYEASMLEAESAQLALAIFHCNWVDMDKRSRKLLIYFIQRAQEEISFTALKIFQINLRTNLSIAKFSFTLYAFMNEMGFGENLKDKK

>CcapOr40

MPQLSASIVISSKTGFNNWLVGKIICKINKKFFRNQAKMDKLRAVIFDRVGLSTKDSFDLLYLNWWLNGNTSWKPHRLGHILHMTICWCLKFFAPVTYFKGFLIALSTSTITTALYNLQATLDVMVAPFKAVVIAKHMHRLRTLTEVFNRLDDRYHNPRERAQIDEGVIICRQIICFYCAVYSGYAVMTWLGALIAGKMPHYLWFPYFDSIPNETLRYWLQFTFEALFIHFMLNVSYTNDVFPVIYMRALRTHVKLLAERVSRVGSNPELSAEEHHRELVDCIVAHREILYIVDVVGAITSLTIFLQFAMAAATLCACMLNVLIFAERIGQIITIIYYMGVLLQTGGSCYQASMLEAESSSLATAIFHCNWLNLDKRSRTLLVYFMQRAQEDIAFTALKLFQINLKTNLSLAKFSFTLYTFMNEMGLGNDLAKSQS

>CcapOr41

MHRVMEYIFGRRRLVVKSGTNDSFELLFLIWKIIGVEHSRSYGFFQLFHVFCCWALLLYSPAAYNMGFLRALKTLPMASALNILQTDINVSILLFKVVIIKFHLKRLRSLRDIFKRLDERYHNPEERAQIDESVAICRRIIYIYIFVYFTFAFLSWITAIMAGELIYSLWLPFVELIPHQGWQYWARFSVEAFYLYFLILVCLICDVYPAVYIRAIRTHVHLLAGRISRLGSNPDLSAEENHQELVDCILSHQELMRVVEVVSAVTSLTLFLQFTVAAMILCVCMLNALIFADRAGQIMTVGYYMGVLLQTGGACFQASMLEAECVKLPLAIFHCQWLNLDRHSRSLLTFFMQRAQVNVCFTAIKLFQINLRTNLSLAKFSFTLYTFMNEMGFGGDTNEKIS

>CcapOr42

MSDLLFGRGQIVYKSRHALTYLFNIFTFVGTNPLGGQSYAYYSLYYLYSLLVNFVCCIFCPISFHIGYIKLLNVLNTNELLSAIQNAIQVSGIPIKIIVIAWYMKRLQSVCEILDKLDENYKRAEDLNSIRRCVRSCCKIIAAFCVPYYGFELSTIAFGVYQNRAPLTIWLPYFDATRTTWEYWTHVGWDVFIMLFLLAHQLGSDTYPPVFISVIRLHMQLLVERVKRLGTNKALCREKRYAELLVCINTYGQILSIANIVAPVISITLFTQFATTATTVLNWFGNMKFPDNIIPMAFFSCQIMQILPCCYYASQLIADCEQLPYAIFHSDWLEEDRRYRKTILFFLQHTQTPIRFSCLKLFGVTLATSISIGKFAFSLYTFIQGKNH

>CcapOr43

MLDLLRGRGRRVYKSRHALTYLFNVFTFVGTNPGKTRTHKYYTLYYTYSLTVNFICCLFCPLSFHIGYIKSWHLLNTTELLAAIQNAVQVTGIPIKIFFITWNMRRLQSVIPILDELDENYKSAKDLLKIRKCVRGCCKMIGFFCLPYYSYEITTIALGVWQNRAPLAAWVPYLDGQRAAWEYWTIVVWDIFVMFFLLSHQLGSDTYPPIYINIIRTHVQLLVERVELLGSDKTKSAEEHYAELLGCIRTHGQIKRIVNLIAPVISVTLFTQFATSATTLLNWFGDVEFPENIISFAYFTCLTLQILPCCSSASYLISDCELLPNAIFHCNWIERDRRFRKTLLFFLQRTQTPLRFSCLKLFVVKLETSVAIGKFAFSLYTLIQGTEVGGKTEN

>CcapOr44

MFELLSGRGIGNCPSSYAFIYLFNTFTILGTNPPSDAGPLYYIWSAFLNTFCIIFSPFLCTVGFIMKYMQSTITTMQFLSGIQAGTNVLGIPPKCLTLAFSLKRMRSIEPLLDVMDARYTDPEDVALIRQAAIMGNRLVFGFGMTYLTYMLLTITPPLISGNVPLSIWIPFLDENQSTLHHLMQVVMDLFLMFFLLFHQVVNDSYGTVYIYVIRTHLRLLIRRVERLCVNGEKSVEDNMAELVDCVTTHQQILSLLTIIEPIISVTMFTQFLIIAIILCVTMVNMFIFADLSTQIASTFYFMCVLMQTSPCCYFATELKADSERLPLAIFHCRWMDQDQRFRKVIIYFMHRAQSPIELMAMKLFPINVATNISLAKFSFTLFTFIKEMGVGQDARE

>CcapOr45

MFDLLKGRGYRELNSRDALIYLFNMLSFVGLNPTAHCRLLYYFYGSIITLFVVVLSPLIFNIGWIRDRNVLSIMEILNCVQAALNVIGVPIKSIALMLCLDRIHSVEPLLLKLDAHYSKWDDMLRIRQCAIMGNRLVFSYIVPYMMYETLTVVSAVLGGHAPLTLWLPYVDWHRSSREYWLQVCFDAITLFYLLCHQIINDSYPAVYIYIVRTHVQLLERRVSRLGYVPQKSEHENCQELQECIVTHQEILRLVHTIQPIISITMFVQFIIAAAIMSITMINIFIFADLATRLASFVYLICVVLQTAPSCHQASYLQGDCEKLSSSIFHCNWIAQDKQFKKLLIYFLQRSQADMPLIALKMLPINLATNVSIAKFSFSLYTFIQKMGLGAHLND

>CcapOr46

MFDLIKGRGRTVFASRDAVIYLFNSFRYLGINPPDKYRIPYFLYSAIITFFAVLFSPVIFNVGWLRDRNKLSVMEILTCVQASLNVMAVPLKCITLAMAQNRLRSIEPMANELDDYYRQPADKVKIKKCAVTGNRLVFGFAVSYLMYETLTVVSALVGGHAPLSLWIPHVDWHRSTWEYWLQVSFDAAVLFFLLYHQVLNDSYPAVYIYIIRTQVQLLAHRVENLGYDETKSDDENYKDLLECIVLHQKILKIVSIVEPVVSVTVFTQFLVAAAILGVTMINIFIFADLTTKIASVTYFFCVLLQTSPTCYHASYLLADCDELRLSIFNCNWIAQNKRFNNLLIYFLHRSQDSIPFFALKLVPINLATNLSIAKFSFTLFTFIQEMGLGENLKG

>CcapOr47

MFDLLKGRGRSVFASRDAVIYLFNIFRFLGLNPPPQCRFLYFFYGSIITLFAVLLSPFIFNVGWIRDRHILSIMEILNCLQAALNVIGVPIKSITLALSLGRLRSAEPLLMKLDARYTDPEDVARIRGCAITGNRIVFGYIISYMMYETLTVVSALMGGHAPLTLWIPYVDWHRSAREYWLQVSFDAAMLFFLLFHQILNDSYPAVYIYIIRTQVQLLTNRVRRLGTGGSSRDDAYHELQDCIITHQEILNLVGVVEPIISATMFVQFFIAAAILGTTMINIFIFADFATRIASVTYLFCVLLQTSPTCYYATHLQSDCERLSMSIFHSNWLAQGKRFNQMLIYFLHRSQADIPFFALKLVPINLATSVSIAKFSFTLYTFIQKMGVGKNLKQ

>CcapOr48

MNRFRKQSARAAETENNKLALALSKEKLSGLKNGEEQRIARKPAKPASAVREKQGEFVASPASSKEATNYFFKAAFGMGIMLPTRHRILYILYSFAVNSMATLYFPIGFTLIFFTLPEDDLDVSNLLTSLQVTFDVYAGSIKLIIMAFLLGKLRTSEIVFQQLDNRCRTPDEMNELRKMQQFGRKVIIFYMTIFLIYSSSTFLGSVTFGYPPYSLYFPFLKWRRSRIEFIIASLLEFLIMDLACLQQTVNDGCPVVYVNILRTHMKILRSRVEKLCTNAALTKEQNLLELKLCIKDHQLLLELYEIIASIVSITLFLQFTVSAICVGTTLINFVIFANGFSTRVACFCFILAVLIEIYPICYYSQCLITESEGLSDVIFHSNWIEQNKEYRQLLIFFIQNAQRPMSLTAGKLYPVTLSNFISIAKFSFSLYTFIEKMNLKERLGIE

>CcapOr49

MADTAINSQRLQTDTLNNGLKKKGDQLAVRTEHATNYLFNGFRVLGIYMPARRKWLYSLYSLIPNSLVTLWLPLSFVFSYFTMSAEDLVPSSLLTSIQVAINVIGCSVKIVVMAFLLPKLRKANAYMDRLDARCKDEDEIAELRKIVKQGNRFVVLFAMSYWSYASSTFIGSVTFGRPPYDLYNPFIDWRKSKLEFVAASLIEFALMDVACFQQVVDDSYAVIYVCILRTHMNILLKRLGKLATCTEMSLEQNLEELKLCIRDHKNLLGLYNIVAPIISITIFIQFMITASILSATLINIFIFANQFSTQVASCFYILAVVVEVFPLCYYAQCLMDDSDRLSQQIFHANWIEQDVRFRKMLIFFMQRTQRVMELNAGKIFPITLGSFLSIAKFSFSLYTLIEKMGIRERLGLE

>CcapOr50

MSNLLQRLLNQLLPSRTTQKSIDVVKQVSPSLSNAELIRIQFERATRTPKERSAPVGRPYQAVHDIHSRDGLIYLYRSFSALGVLMPDKHKILYCLYALLPLGLITFYLPISFALSYFYLDYSTVKIGNLLTSVQVFIASIVGGVKLIVMAFKLPKLRASEAIMHQLDARCKDEDEIEVLRKVVRQGNRVFVLVLICNLIYSTSTFLAAASKGRPPYNLYNPVVDWRKSKGAFLWAALWEFILMDGLCTEEAITDSYAPIFVCIMRAHMKTLLMRIQKLGSNPERTLDENYEDLKMCIKDHKLLLELFDVVHPIISTTYFLQFMTTSLMVGCTLLNIMIFAVDNLARVGHLAYVMALLMEVYPLCYYGQSLLDDSNRLANTIFHANWIKQNEKFRKMLVVFTQHTQKPMELLAGKLIPINLTTFVSIAKFSFTLYSFINNMGVKERLEGL

>CcapOr51-CTE

MSTDKPEPQANAEHMGSPLDVQRRDMFTFIRWSLWFTAMCRLPLEYYLPTSLRFLASTLNWIYEVFLYFTIIHIDILFMCTIYLYKDKGDLALLVNSLIQAIIFMWTLIIKVYFKRIKRKKIEELMQFLNNEYRTQSAAGFTYVTMKESVDLSNTWTKVFLISCYVGGAFWLFVPFLRRDRSLPLPCWYPFDYKSPIVYESIYLLQCLAQMQMAAAFASTSAFYLLVAVVFSGQLDVLNCSLKNVIATTYLNLRKPKSELIKLREEHNIENFEINQYYCAEEHKTDLDCLPHLLDGENPEPQNFYAAFKQAFKHCVTHHQYILCGLQMLEDIYSYLWYLKTMKATVLACLFA

>CcapOr52

MALADRKTPPTDTEHIGCPVYSTQRRDMFRFMRWNLWFTAMCRLPLEYYFPTCLRCLANTLDWTYEVFLYFTLLHIDILFMCTIYLNKDKGDLALIVSCMIQTVIYTWALIIKVFFKRVKPKRVAKLMQYLNNECRTHSAAGFTYVTVKDSVELSKNWISIFLICCYAGVTFWLFVPIFNADRSLPLACWYPIDYKANVPVIYESIYLLQTIGQLQIAGAFGCTSAFYLLACVLFSGQFDVLNCSLKNILATAYINLGKSKSELCKLRDKQYIADKELNQYYCSKEYKSDLDCLPHLMNVATPKPKTLYAAFKQAFIPCITHHRYILYGLQMLEDIYSLLWLLKTMEVTVLVCLVAFAWVKSTTAKSFLSILSLSQYLLLALWEMFMICYSGEIIFLNSQRCDEALQRSPWYLHANEIKQDTLFFILNAQRPFRLTGGKMFDLNVEKFRSILTTSFSILTILQKMDVRPTQPK

>CcapOr53

MILTDAPTAHAAAQLCASPNGCLSNVKRRDLFRYVRWLMWGAAIRPIPFENHLPRRLGNYSAIINVILEIFLLLTVIHIFVLFILTLYLNYGSGDLEFFIGCSIQSMLYFWAIIIKIIFRRVRPELVRDIMDYVNEKYIVHSAVGFTYVTMNECLEQAERGIKYFVLSSLVAVIFWLFQPVVYEERTLPLPCWYPFDYKAPFIYPLAYFLQVIAQLQLALTFVTNSIYFTVLCFLLCGQFDVLNCSLKNILATTYILMGASRKDLIELREHQCNADDEINQYFVAEELHINLDCIPHVLTPATTAGTMNFRDAFHCALGQCVDHHIFILNALRKTEKLFSMVWFFKTLEVTFAICTIAFDVVKSTDDKSFLQVLSLGQYMILVLWEMFMICYGGEIVYINSQRCDLALLRSPWYLHSREMRAEILFFLLHAQRAFALTGGKFYPLKLEKFQAILTTSFSFYTLLQNMDQRN

>CcapOr54-PSE

MQNTAGACSIILYSEKDKPRVCDLFMAQVLAFKATGQIPFNWRWRLGYIYCFIVIAQTFLLAVLFLKSSYIMLLSGKLEEITDALTMTIIFWFSVYAACYWLLRWRRLMAFLELINQQYWHHSLPGLSFVSWQRTYLLAKRMTIVWTVACVLGTVLYGLAPLVMGVRALPLKAWYPFDPLQPYVYELVYVMQLSAQIIMGATFGNGSALYVSLVILMCGQFDVLYCSLKNLSHSARLRCCSGVEILRKEQAALPKSPDDELNQYMYCREHLTNLSILQHLYTQQPALTLPEALHLGVVQCVQLHRFILDACKELEELFNPYCLVKSIQVTLQLCLLVFVGVAGERSMVRIVNLAQYVTLTLVELLMFTYFGELLRGHSVRCGEAFWRZQWWTHTIPIRQDILILLANSKRAVRLTAGKFYAMDIERLRSVVTQAFSFLTLLQKLAAKNQK

>CcapOr55

MLHECSDKSTISNTIFNHQSYRRRSRKYSSSKLETILAPIRTLKEMLLSGDAAHPSHTCLYYVRAYIRLLGLWPSQRGVEQPMYYAYNVLIMTVFSFFVATIIADLYVASSDFVLLGEDLVVALGLYLILFKMILFRMSTADVDVIVDEFDALHMKFARDTSDSPHIRRIRQLQRSFFLGEASFFCGFFFLSLFLFAAMSLQPLLTHQALPFRCVFPFGLHDPDKHHITFVCVYAFQCFCTLYMLVSIVVMDSLGGNSFNQTTLNLQILCESIRHIGYAGGRSTTITEAVLWRELRENVEFHVKIIELVDGINHTFYWNYVSQMGASTFMICLTAFEALLAKDQPMVAMKFQTYMFSAFMQLFYWCFMGNRTYYDSMEVATAAYEVYAWYEHSPRLQRNLLFMIKRAQKPLEFRSKPFFGFTFASFNSILSTSYSYFALLRTMND

>CcapOr56

MIRAQQLNGHKVPSDVVTMILEEVEDVYKRNYNSIKVLIRVSFGLGVNLTAPTRFKDSLRIFNVILVTTSILSLYAHWCYLIRHFDNIPLLAETVCTALQTLISAVKMVYFLFTQRTFYRLIDQALTHEVIRKIEIFKYDFPINRQLKQEIDDIMSGVWRHARRQILFYFCCCIGIVFNYFFGAFFVNLYHQLKQTPNYNFILPFPALYPFWEAKGMTFPYYHLQMYMTGSAVYIAGICAVSFDGVFIVLCQHAVGLVKVHNLLVLRATSPLIPPERRVDYLRYVIFTYQRVRVYVQQVQTIYKHVSLSQFVLSLIVFGFVLFEMSFGLESSITIFIRMIMYISAGGTQIIIYCYNGQQLTSVSEEMPLAFYSCGWYEESKKFKQLLRMMIMRTNRHFYLEVSWFTLMNLATLIALFRMSGSYFLLLRNLQES

>CcapOr57

INVLLIIASCIALYPHWLMIEHAEGNLSLIAETSTTALQTTTALVKMAFILFKQHRLHELLYKAEYHELLQGIQIFMTDMPIRISLKNEAIRIMDTTWQEARGQLLFSLISCICIQANYFFYAFFKNLYHHLQGTPNYVYILRMYSFTGYPMFHHKGMSSFYYIMDMFFGACSLHCAAMCAICVQCTFMVLCKHCCGLVQVQCLMLLRSTSPLVPKVRRVEYLRYCVIQHQQILRFMEGINQLFRHICLSHFLHSLAIYGFVLFEMSFGLESNKVIFVRMMMYLCAALTCDCMFYVNGQFLSTELEKIPLACYSCEWFHESREFKMILKMIIMRSNKPFYFQISWFTVMSLATLMGIFKASGSYFVLLRDIDEA

>CcapOr58

MATKTIRPTQKFAKMIKIVRFISSLVGADVADENYRINIVTVLVILCIIIYFIFTGTTVASVFAENWKYLLEASCMVGSVLQGITKLISGVGCTKMISSICKELENLYQHYETKGEAYCKVLNEGCERVWYSIKMVGHIYAAAIYGILLLTGFLILTTNEKVYVMHFFIPGVDVETTYGYLFTLAVHTVVFLAGAFGLFAGDLFFLIFLGQTQLFRDILVLKVKALNEAAAENAKNTESLLIDIIEWHQYYTDYNKRCNDVFYYIITMQIVTSGISIICTMYILLMGDWPGAYLYIFVAFCGLYLYCIIGTSTQTCNAEFFDEIYNINWYELDVKCQKMMVFIIKKSQSPAEIKIGGVLPLSVQTALQITKSIYGLFTMIIGVIEENN

>CcapOr59

MAAEKVSPSESFAKIVKIFRLICSLVGADVCDVNYRINIVTAIVIFCIIIYFIFTATTVASVFAENWEYMLEASCMVGSVLQGITKLTSGIAFAKEICAMRFELEDLYRLYETRGEEYTAVLHLSCKRVWQVIKMVGQIYLAAGVGILFMTAIFIVATDEKVYIMHFFIPGLDVHTQMGYLLTMAVHTVVFLAGAFGLFAGDLFFLLFLGQPMLFLDLLTLKVQALNVAADRCSNEAERLLIDIIEWHQYYTDYNRRCNHLFYYIITMQIVTSGISIICTLYILLLGDWPGAYLYIFVAFSGLYLYCIMGTKIQICNDAFCEELYNIDWYKLNVKSQKMLVFILKKSQKPAEIKVGGFLPLSVQTALSITKTIYGIFTMMLRFLDEEN

>CcapOr60

MKSQVKRTLEPSESFSKIVSVVRFCSRLIGVDVFDKDFKINPKTYFVMVAFAAYYLCALHTLSKYIATDWTVLLDIFSPVSCTTQGLVKFISALLYPSLYHRLSVEIGDIYEKYQWMGKKYEDKLMEWNKSMKKILLSCAILYFLTALIVICTPVVLYVLKGERHLTLLCEVPGFDVTTTQGYLVTNGFNTVCILIAAFGLYAGDLFLFIFLTHSIFFYDILALKISDLHEIIEENDRDERLGKMVDDIVEWHQFYLGFNDTCNLLFFWTISAHIVCTTLGILSTLLIIMLKDWPGAYAYILVCFLWLYMYCLLGTRVEICNDQFCTGIYDINWYALNVRNQKIVRLMLMQSQAPRNITIAGVEPLSVSTALKITRTIYSLVMMVLRFQSK

>CcapOr61

MQQPTTKRPSDKYNKLLSIIRFSSTLIGVDVIAENYKFNWVVGFVFAAIGWNFFCSSYTIWKDVTTDWTVLLDVFSPISCAAQGTIKLFSLVCYPKLYRKLALDLGEIYEKYQLLGQKYEKKLLTWNKDMKRLLIIGGLIYFASAWIALITPLGLYILKGEKHLIIMCQMPYIDGSTNQGYFILIGYNLICVFVASFGLYAFDLYVFLFLTHSIFFYDIFALKVDDLHEVLRQNDKDKRLLSLVNDIAEWHQYYLEFNDQCNLIFFWPITSHILCTTLGILSTLLIIMLKYWPGAYPYIFVCFVWLYMYSFLGTRVEICNDQFCDGIYDIKWYDLDLRNQKTVCLMLTESQVPRIITIAGVEPLSMNTALKITRSIYSLAMMVVQFNE

>CcapOr62-PSE

MFSPKQPSENHAQLIKVLRFCARQIGCDVLEENFKFNRTMGAVLIAIIWYYLCSVYQIAKDFATDWTILLDVCSPVSCTTQGLVKLISVLLYPKLYYQLAGEIKETYEKYEKMGTKYKDILHRWNKAMKKIIVFLAMVYLLTAVLMLSTPLALYIFKGERHLILLLQMPFTDVTTTRGYLSATIFNVMCIFIGSFGLFAADSFLFLYLSHSLFFYDIFAQKIEDLHELLEHNRQDERKTALMNDIVQWHQFYLRFTDNCNLIFFWTISAHVICSTTGILSTLLIIMLKDWPGAYVYILVCFIWLYMYCVMGTWVEIV

>CcapOr63

MNAIKRNTNFLRFTAGPVKYFKVIGICLQPPETLSIKFARFLTVLTTLLLFVHQIAFLVTPGRTFVELSAAVGLLNYTTVGAGKILFLILNRHLLLKSYTQLQAIYPSEAVERHYKLDRYLLIYKRVETLLYNFFKYILIVYLVSPIVQSFYDLWSNGAYSYRMPTIIWYPVPLEESLLEYVVYLIFESYTSFSLGTIILSADLCLFSSVSQLMLHLDLLAQRILELQPAEQGSMNDLKAIIEYHQRILTLAQDVNSIFAPSIVFSLASSSFILCFSAYQLLEDVSFIFALKVLLLLGYEMKQVVITCYYGDKLMDSSANLFNVVYAHDWTDGTPAYKRLVLIMLIRTYRPIALNVAGIADVSLITLKQVLSTSYQIFAVLKTA

>CcapOr64

MALQAGRGGEPKLRSIDDLCAILHPIQRYLSINFLDFTRINGRFAIPSSMLLNVGIVLSVLDCMGNITKVCMAINDRDLTKAQETFAVLGMAFVMTMRGMMLARSRVRLSELYNSIDRIFPNSSELQTHMEVAKTHDYIKRRFFLLHQGLSFALVLFCTMPAVKLVFFYDFEAQEPVADEFHVNPSWVPFQVKETISSYGYIYVYEVILALVAVNMIITWDEVFVVLISQLCMYYQYLAKLLTALDVREANDPKKAVAFFKRLHLYIYIHQYLNSLADELNDLFNLSILVSDMGTAMSICFNLFLVTGAKDYLQIPSYLTPCFVETWLIYDVSKWGTMLETVTARINEVLYEQKWYDSSVRFGKYTTMWLQGTNEPIRLTAYHIFDVNMKHFQDMMMLAYQMLTFMKSKS

>CcapOr65

MHFRIYNIKDYLIYPEFAFKLGCFEPFIWSGNRHRHQQTVSYHLKSVICIFDASTVICQIFALVISLFVPRTTAEAELYEDSESRVFEAVALICYFACGFYKFWNIFWRRNDIGLVLEELKNLFPSVSKQKMQAEAKANSGEKNDAIACYRLAYYEDKSRIMMQRLTRYFMFAYFYYNIIPILQLFAEVISHQEVITFKSQSNAWYPWHNHNHHSTFVGFVFSFLIQASAEFAAISFIMSGEFLFCFLNTQLQLHFDYLTGALSALDARSPNALLHLKTLINYHNQLLRLSKLINSIFNFTFALDLITTTFAISLMGLTMVMVRFGQAVMFSAGFSFFLLLGFLFCNNGDELINATKNLGAAIFYSNWYEGSSEYRRMIIFFIMRTKTPCEYRAFGYMSLSMETYMRILKLSYQLFTSFRAIE

>CcapOr66

MSGVFSMNHFLKYPNFTLDLAWSNPFAWSGARQYGYHMLWIRRLLFTFGAANLVYQNFGMMIYLCMPHEVSNESTIGQITETGGIMGLTMVGASNMFVMYWHADRIALLLEKFQRLFPTKQLQWRAKHANGRLRGVKFPHSVEYFALKSNKLMKIATTAYLFAFSYYNSLPIVEYLYESLTPGVELKYHYQSNTWYPWQNAHNRKSFIAFLAAYICQVQSSLTGVAFIMAAEFMLCFFITQLQMHFDYLANALETIDAAGENANEELKFLIHHHGRLLSYSKEINAIFNISFLVNIFTSSIAICLMGFSMVMISVIHACKYCIGLLSFIVFTFFICYTGKELTDASDKLLYAAFYGNWYEGDLAYRKMILFLIMRCRIPTVLRAYKFTTVSMPTFTAILRSSYSLFTFFQAMGK

>CcapOr67

MDFEKIFWLPNTLYLVVGYDFRQVSKSYLKKILMTAFLILTNITGICIRIYMLIQLRELVLSGDMLNSFRLGVYISYAFDSIVKFFGFLHNAHRLRKIYESLATEFPQTFSEQQFYQVHKYSFNRSRILIFAYLSVTNSILLGPIVQSIIMYIIDAFLYGLSGAKFQCLHPTPITYNFNFCSPRYYIPIYIVEYLNGHFLTTTSLGTDLYVCTFAAQVCMHLKYLGNSLEGYEPSADNSKADCAYLKEWIKKHQLMLRLCADINDVFGTTLLFKLISNCTVFCIIVVQLKLEGFGWGFLNFLCFFFVTVAQFFMVCHFGQKLINTSEDVSLCAYKNRWYNGSKAYKTLLFTIIARSQKSCKLTAKGFQPISLQTFQIVMTMTYRAFAVLQRALD

>CcapOr68

MAPYFHTREPAATIPDFVGIPFFLISLNGMQLFKWTPNEEASRRKLLLITAFSVIVTYDCVSMLSVFAFVKLERLDYTTFALYWGYALNSLMKGGTLWFGRRQLEFILKSMVEKHPKTIAERQEYHLVAYFTKIKSFNKYLTIFHLCTTSLFNIQPMVSSIVEYMGRQDKEEEFKYKLPFIMYYYYNERQPVLYLFSYFLQCMGGFYMSYLFLGGDLLLMTLVHLVNMHFEYLIRRIESLQPTEDSEKDLNLLGPLVTYHLEILDLSILLNYIASCLCLCLLGLQIVMGSDLVTVVKFFAFLVSTMVHVYYISHFGNNLIDLSTGISDAFYNHPWTNAKYKYSRMLVLPIARAQRYAHLTAFQFFEISMHSFKSLCTTSYQFFTLIRTSLEEDFH

>CcapOr69

MPFAFQQLCFELQLSLKYSVPAMPLKLANNEPAATIQDFVGIPLFLLTFMGVKLFKWTPEEASSKRQLIMLGVFCVFATYNFATMILYIMYEPLNSSLDITEIILFWGFSLNGMMKLAIMILYRNELKSILRGLGARHPQTAEERSIYRLVPYYNKILIYNKYLAAWHLSITTLFSFHPLVASILGYIFRRDSSDGYDFTLPFMMWYYYDTTKPILYIFSYVVQTFGAFWMSLLFLSGDLLLISLVHLVNMHFDYLIRHIESFQPNGTDEDMKVLGPLLAYHQEILDYAERIDSTFSLGTLLNYAGSCLVLCLIGLQIVLGSEFLKVVKFIAFLVSTIVQVFFVSYFGNNLMDLSIGMSDAFYNHPWYDGNYRYSRMLVLPIARAQRYAHLTAFKFFEISMDSFKSLCTTSYQFYTLLRTSLEEEAG

>CcapOr70

MPFAFQQLCFELQLSLKYSVPAMPLKLANNEPAATIQDFVGIPLFLLTFMGVKLFKWTPEEASSKRQLIMLGVFCVFATYNFATMILYIMYEPLNSSLDITEIILFWGFSLNGMMKLAIMILYRNELKSILRGLGARHPQTAEERSIYRLVPYYNKILIYNKYLAAWHLSITTLFSFHPLVASILGYIFRRDSSDGYDFTLPFMMWYYYDTTKPILYIFSYVVQTFGAFWMSLLFLSGDLLLISLVHLVNMHFDYLIRHIESFQPNGTDEDMKVLGPLLAYHQEILDYAERIDSTFSLGTLLNYAGSCLVLCLIGLQIVLGSEFLKVVKFIAFLVSTIVQVFFVSYFGNNLMDLSIGMSDAFYNHPWYDGNYRYSRMLVLPIARAQRYAHLTAFKFFEISMDSFKSLCTTSYQFYTLLRTSLEEEAG

>CcapOr71

MLPAARTFGEFIRIPIRFYQTIGEDLYEHRSPHRIRRLILKALLYIGFLNFNVLVLGEIIYFVKALNSFATVLEATGVAPCIGFSFVADFKQIALTVHRQTLREHLDQMEELFPKTVRQQAQYKLPQRERVMRRVMGVFTLLCLAYTTTFSVYPALKATVQYWLLGAPTFERNFGFAIWYPYNATGKTWVYWLTYMGQVHGAYLAGVAFLSADLILVASVTQLCMHFDYISRCLEDFAGASKKCAEEDIKYLQALVVRHAKCLELSEHVNSIFSFSLLLNFLTASLTICFIGFQVTASSTEDIVKYIIFLTASLVQVFVVCYYGDELMTASLRVGDAAYNQNWFECDTRYKRLLIILILRSQKPASIRAPTFPPISFNTYMKVISMSYQFFALLRTTYSDKGN

>CcapOr72

MSKPPIHFESFCYQANIFYTSIGLDVYDKPGERSTSKAIQLURQQLLGIFFAITIINMNIVLLSEFMYIFMAFVNNNHFVEAIVLSAFMGFVFVGDFKIYSIWRRRTHITVMMRDLYALYPQTPEEQSNYEVKMELQRYSRYAFAFIMLYELSFWSYNLFPLLNYLITDFLLGVRAVDRTLPYNCWTPFEWHTVNWRYYSMYLSQIAAGQACLSAQLANDLLLSAVAVQLIMHYRQLAKKIESHKAGSTTGKQWKETPFERNVDLQFLCDVIAYHQKILCLSQALNDVFGISLFISFASTSMIICFVLFQITIGASIDLMITLAFFLFCSLVQIFLICFYAQKLLKASEYISYAIYNHNWFDADLRYKKMLVFIMKRAQKPAIMQASSFVMVSMSTMTVLLQLSYKGLAVIRTIYAREPKGVSNK

>CcapOr73

MSTKIVTLDAFVEKASFWFSLYGVEAFDDFHDRSAEHHNWKWKNYLRKLFYYISFINVNWVLLHEAAFVVVNFLENGDFLQAARNLSFMGFVSVADIKILITMQQRTQLSKLMRKLYELYPKDVGDQRHYDLQRHLQHYSCISFLFSFSHTFTVWAYNSLPMINYLIHGHLLQQKTVERTLPYSCWVPFEWRDNWLYYLLYTSQAFAAHSCLAAYLATDLLFCAATVQLIMHFRKLAGDIKQYQPDYASGRQVGINTDLGKDLRFLSAVACYHHTVLEINQLINDIFGLPILINFISTSFVMCFLAFQFTVGVPLDALITLTCYMICSLVQIYMICSYGQELITTSEDIGHAVYNHNWLVSDIRYKKLLIMIIRRSQKPAVLRATSFLNVSMGTITDILQLSYKFFALIRTMYSR

>CcapOr74

MSIIIRFEEFLRLPSFFSRNIGIILWGQRGKLFDRFMFYFSSINLFLTLLAELWYIISTISTDFITAIMGLSYVSFVVLAEVKFYYLIKYDMKVSTVLKRLNALFPHTKEEQENIQLIKYLKMSKFYTLFYTVTFMLVIWTYNLYTVSQRFIYTKILQVREIERELPYPAIYFWNWQDNWSYFMLYISQSLAGWHATCAQILTDLLICILISHLIMHYDHIARSLLNYQSKFAELYGKESTMKCMPKLARVMMEERAVRADMKFLADIIAYHTELLSLTESLNDVFGVPLFMKFMSSSAIICFLGFQMTVNRGFDLLTKLALFFILSVLQVYLICHFGQLLIDASTNVSTALYSQDWTNADVRYQKMLVLIIKRAQRSATLKATNFIIISRATMTEIMQMSYKFFALIRTMYND

>CcapOr75

MCENIESFEAFLRIPSFFYRSVGVDLWNTNGGSIQRFIFYFGFLNVNLWLLSELIFAIITVSENFIQATMTLSYAGFVLVGSIKMYFMWRKKTEMTQFLKLMDEIFPRTAEQQKMMNLRRHLRQSTIVMSGFALIFMILIWTYNLYPFMQRQIYDCWLDTRSINKTLPYESYIPWNWHNHWSFYLYYVLQSIAGYHSAAGQIASDLVLCAMATQMIMHYEYVSHKIRSRYRGERKCVDSKSVSCLNALNHWTEEQVATHKDMRWLCETIAYHSNLLSLSDVMNDVLGVPLLVNFMTSSFVICFVGFQMTMDAEPDYMVKLFLFLFSSLAQIYLICHYGQLLIDASINVAAAVYDQDWFDLNVRYQHMLVLVVARAQKPAMLKATNFVRISRGTLTDIMQISYKFFTLIRTMYSD

**The Gustatory Receptor (GR) family**

The GR family of seven-transmembrane proteins in insects mediates much of insect gustation  [1], as well as some aspects of olfaction, for example, the carbon dioxide receptors in flies  [3, 13, 26]. In *D. melanogaster* the family consists of 60 genes encoding 68 proteins through alternative splicing of some genes  [18], while in *M. domestica* it is considerably larger at 79 genes encoding 103 proteins (of which 13 are pseudogenic)  [10]. The GR family is more ancient than the OR family, which was clearly derived from within it, and unlike the OR family is found in the crustacean *Daphnia* *pulex*  [27], a centipede *Strigamia maritima*  [28], the tick *Ixodes scapularis* (HMR, unpublished), and many other animals  [29].

The CcapGr gene set consists of 73 models, encoding 81 potential proteins through alternative splicing of five loci (see Additional file 2: Table S12). There are no long pseudogenes in this gene set, although a few fragments were noticed. Three gene models required repair of the assembly. As is the case for some *Drosophila* and *Musca* GRs, at least five genes appear to have an unusual form of alternative splicing in which multiple alternative long first exons are spliced into a shared set of C-terminal exons downstream of the last long first exon in these tandem arrays. The resultant proteins differ considerably in most of their sequence, and hence presumably bind different ligands. They are indicated with a lower case letter after the gene number.

The medfly has an intermediate GR family repertoire size compared with the smaller *D. melanogaster* and larger *M. domestica* repertoires. This is mostly the result of *M. domestica* having three large species-specific subfamily expansions, while *C. capitata* has only two relatively small species-specific expansions (see details below), independently of two relatively small expansions in *D. melanogaster* (Figure 6).

The carbon dioxide receptors are highly conserved within most of the holometabolous insects, except the Hymenoptera to date, with two proteins represented by DmGr21a/AgGr22 and DmGr63a/AgGr24 constituting the functional receptor  [30]. *Drosophila* species have, however, lost a third member of this subfamily, first recognized as AgGr23, which is present in *Tribolium*, *Bombyx*, mosquitoes, and *Glossina*, and known in those species as Gr2  [30]. This gene is an ancient paralog of the DmGr21 or Gr1 lineage. *Musca* does not have this Gr2 lineage, but *Ceratitis* does, so it was apparently lost between the divergence of the tephritid lineage from that leading to the *Musca/Drosophila* split. The importance of this protein is controverse with findings of either enhanced perception of carbon dioxide or the opposite  [26, 31]. Like *Musca*, *Ceratitis* also has a recent duplication of the DmelGr21a or Gr1 lineage, that have been named CcapGr1.1 and 1.2  [30], while the DmGr63a ortholog is called CcapGr3 (see Additional file 2: Table S12 and Figure 6). The only other known recent duplication of a carbon dioxide receptor gene is the Gr2 lineage in tsetse fly  [32].

The sugar receptor subfamily consists of seven genes in medfly (CcapGr4-10), and is a slightly larger set of eight genes in *D. melanogaster*  [33]. Study of these genes in other available insect genomes indicates that they represent four major lineages that duplicated in basal Diptera  [34]. One lineage, represented by AgGr16, was lost from *Drosophila* and *Musca*, and *Ceratitis* does not have it either, so this was an old loss in cyclorrhaphan or more basal brachyceran flies. The other three lineages are each represented by two or three paralogs in *Drosophila*, specifically DmGr61a and 64a, Gr64b/c/d, and 64e/f and 5a, all of which are proposed to have once been in a large tandem array, with the terminal Gr61a and Gr5a genes moving from that array  [34]. These genes were poorly assembled in *Musca*, but appeared to have a similar arrangement  [10], however *Ceratitis* has all seven genes in a single perfect tandem array, confirming this model (see Additional file 2: Table S12). *Ceratitis* has, however, lost the ortholog for DmelGr61a/MdomGr4, while duplicating its ortholog of DmelGr5a/MdomGr5 (Figure 6). These sugar receptors are expressed in complicated combinations in subsets of sweet receptor gustatory neurons in Drosophila  [33], so it is unclear how losses and duplications of them affect perception of different sugars.

The conserved DmGr43a lineage has been shown to be a fructose receptor that also serves as a nutrient receptor in the brain  [35]. While *Musca* has a species-specific duplication of this lineage (MdomGr12/13), it is expanded to four genes in *Ceratitis*. Although the tree suggests that only one of these is truly orthologous with DmelGr43a/MdomGr12/13) (Figure 6), separate phylogenetic analysis of this DmelGr43a lineage in diverse insects reveals that these four genes are actually a species-specific expansion in *Ceratitis*. Duplications of this lineage in other available insect genomes are not uncommon, with the hessian fly *Mayetiola destructor*  [36] and the silkmoth *Bombyx mori*  [37] each having duplicated it, while *Tribolium castaneum* has 10 paralogs  [38]. It is not obvious what role these duplications may play, but perhaps this is an example of subfunctionalization.

The DmelGr28b alternatively-spliced locus ortholog is relatively straightforward in *Ceratitis* (CcapGr41a-e), having the same five alternatively-spliced forms as in *Drosophila*, unlike *Musca* with multiple novel duplications (MdomGr39a-g) (Figure 6). The various splice forms of this gene are expressed in both gustatory cells and in the brain and elsewhere  [39], and some have taken on novel sensory roles, having been implicated in perception of both light and warmth  [40, 41].

Most of the remaining *Drosophila* GRs are implicated in perception of bitter tastants or have not yet been functionally characterized. They were named sequentially in *Ceratitis*, with no implications for orthology with the *Musca* genes. Some have simple orthologs across all three species, for example: DmelGr2a/MdomGr16/CcapGr24, DmelGr10a/MdomGr42/CcapGr50, DmelGr33a/MdomGr38/CcapGr42, DmelGr47b/MdomGr65/CcapGr71, DmelGr57a/MdomGr66/CcapGr72, DmelGr59f/MdomGr71/CcapGr64, DmelGr89a/MdomGr73/CcapGr66, and DmelGr93a/MdomGr74/CcapGr68. The highly conserved DmelGr66a protein required for detection of caffeine and many other bitter tastants  [42] also has a simple conserved ortholog in both *Musca* and *Ceratitis* (MdomGr36 and CcapGr38), however there is also an older duplicate of this gene in both flies (MdomGr37 and CcapGr39) that was lost from *Drosophila*, and presumably was also involved in detection of bitter tastants.

DmelGr32a is a particularly interesting candidate bitter taste receptor  [42], that is also involved in courtship through expression in a small set of gustatory receptor neurons on the male foreleg  [43), and mediates rejection of non-conspecific females as targets of male courtship {Fan, 2013 #69]. Like *Musca* (MdomGr14), *Ceratitis* has a single ortholog of this gene (CcapGr15), however it also has four related genes (CcapGr16-19) that are related to DmelGr68a (lost from *Musca*), which is another candidate pheromone receptor expressed on the foretarsi of males  [44]. These expansions of candidate pheromone receptors imply a more complex set of cuticular hydrocarbons and other pheromones involved in sex and species recognition in *Ceratitis*  [24].

Many of the remaining candidate bitter taste receptors reveal complicated patterns of gene duplication and loss in these three flies, however the *Ceratitis* genome reveals some interesting genomic history for some of these. For example, despite being highly divergent, CcapGr43-52 are in a mostly tandem array (two genes are in inverted orientation) on a single scaffold (see Additional file 2: Table S12), indicating that they evolved by duplication via unequal crossing over, much like many other chemoreceptors (see sugar receptors above). Remarkably, their entire set of relatives in *Musca* is also a complicated array on a single scaffold (MdomGr42-64)  [10]. In *Drosophila* their relatives (DmelGr10a/b; 36a-c; 47a; and 59a-d) are now on four different chromosome arms, presumably reflecting higher genomic flux in the *Drosophila* lineage after it separated from the *Musca* lineage. Similarly, CcapGr21-34 are two arrays on the same scaffold (Table 6), while the related expansions of Mdom16-19, 22-29, and 41 are on several scaffolds, and the related Dmel2a, 8a, 23aA/B, and 39b are on three different chromosome arms (Figure 6). The expansion of CcapGr55-61 are also in two arrays on a single scaffold (see Additional file 2: Table S12), while their DmelGr22a-f and 85a relatives are on two different chromosomes (Figure 6). In the tree most of the bitter taste receptors cluster together on the bottom half and upper left quadrants, except for the Gr66a and 47b/57a lineages which cluster with the Gr28a/b and Gr43a (fructose) receptors.

Finally, the comparison of *Drosophila* and *Musca* highlighted several instances of possible gene loss in one or other species amongst the many candidate bitter taste receptors. Addition of *Ceratitis* shows that some of these were artifacts of the phylogenetic analysis of these sometimes highly divergent proteins, for example DmelGr39b and MdomGr19 are now clustered in the tree. The addition of *Ceratitis* also allows improved understanding of some of the real gene losses. For example, DmelGr9a and 10b are missing from both other flies, implying that they were independently lost from the *Musca* and *Ceratitis* lineages. Two particularly intriguing examples are the gene set of DmelGr22a-f, which was independently amplified to seven genes in a split array in a single scaffold in *Ceratitis*, and DmelGr93b-d, which is an alternatively-spliced locus in *Ceratitis* (CcapGr67a/b) (see Additional file 2: Table S12 and Figure 6). Both of these lineages were lost from *Musca*, hinting that, like the expansion of genes related to DmelOr7a in *Ceratitis*, these gene lineages are involved in the common role of fruit tasting, a role *Musca* does not need.

In summary, while maintaining a conserved set of receptors for carbon dioxide, sugars, most bitter tastants, and light and warmth, with an expansion of fructose receptors, *Ceratitis* is more similar to *Drosophila* than *Musca* in maintaining and expanding receptors that might mediate perception of fruits, while being differentiated from both in having expansions of receptors implicated in perception of courtship chemicals.

**81 CcapGr proteins in FASTA format**

>CcapGr1.1

MAYWAIATRKGESPPMKITPVLNPNQREFLEDELLYREKLEILAENNTLSTDLFVRKFEDIDDPVLLDKHDSFYHTTKSLLVLFQIMGVMPIHRNPQKYGLARTGYSWTSKQVFWAIFVFTVQTTVVVLVLRERVNNFLNDTDRRFDEAIYNVIFISLLFTNFLLPVASWRHGPQVAIFKNMWTNYQLKFLKVTGAPIVFPNLYPLTWGLCFFSWGVSIAINLSQYYLQPDFKLWYTFAYYPIIAMLNGFCSLWYINCTAFGTASRALSASLELTLMSDKPAKKLTEYRHLWVDLSHMMQQLGRAYSNMYGMYCLVVFFTTIIATYGSLSEIMDHGATYKEVGLFVIVFYCMSLLYIICNEAHYATQSVGLDFQTKLLNVDLTAVDSATQKEVEMFLMAITKNPPIMNLDGYANINRELITSNVSFMATYLVVLLQFKITEQRNYSLKEAAML

>CcapGr1.2FIX

MSFWVKNADGSTDVEKPPRIMPLFNPGQREFLEDEQRLRLQMEKRALDGGKSFDYYIRKQSTLDDARLLDEHDSFYKTTKSLLVLFQIMGIMPIHRNPPDNNLPRTGYSWKSRQVLYAICVFSLETYIVVMVLRARVKSFIEQPDKHFDEAIYNIIFISLLFTHFLLPVASWRHGPQVAIFKNMWTNYQYKFWRVTGSPIVFPNLYALTWGLCIFSWTLSIAVNVSQYILQPDFEFWYTFAYYPIIAMLNCFCSLWYINCNAFGTVSEALAKNLELTLKSGKPAEKLTEYRYLWVDLSLMMQQLGRAYSNMYGMYCLVVFFTTLTAAYGSISEIMDHGATYKEIGLFVIMFYCMSLLYIICNEAHHASRKVGLDFQTKLLNVNLIALDTASQREVQMFLVAISKTPPIMNLDDYANINRELFSSNITFMATYLVVLLQFKITEQRGSHSNQDEFNTM

>CcapGr2

MKTGNLLFSEMLSKSPKSSGEKLSDHAMIGQLNSFLFDATDNDAVERHDQFYRDHKLLLVLFRVLAVMPIQRTSPGRMSFSWKSSATIYAFIFWTVMTIIVLIVARERIQILYTTKQFDEYIYAVIFVIYLIPHFWIPFVGWGVAGEVAEYKTSWGKFQLIFYRVTGTSLQFPHLKSTILAISVGCLLCAFLFLFALSFFLEGYPLWHTLAYYHIIIMINMNCALWYINSRAIKTASTALTTCFQKEIVSSHSSDVISKYRMLWLYLSELLQSLGNAYARTYSTYSIFMFINIVIAVYGAFAEIVDNTDVSRDSYKEVGLIVDGLYCSTLLFIFCDCSHNATLGVAKGIQKVLLEIDVRHIDRKAKSEIDLFIFATEMNPAIVSLKGYVNVNRELITSFIATITVYLLVLIQFKFTLN

>CcapGr3

MFNSYNRRKKHDTVFLNVKPTFNSQGGGLRKYSTGLLDKEENPFYNPNSSNASRASVGTITTLNENFRANIFYNNIAPIQWFLHLIGVLPITRRESGKAKFRINSVAFGYSLVFFILLSVFVAYVAKNRISIVTSLSGPFEEAVIAYLFLVNILPLLLIPLLWWEARKIARLWNDWDDFEILYYQISGHSIPLNIRRKTTLIAVILPLLSILSVVITHITMADFQLLQVIPYCILDNLTAMLGAWWFIICECLSMTANILGERFQRALRHIGPAAMVADYRALWLRLSKLTRDTGNATCYTFTFINLYLFFVITLSVYGLMSQLSEGFGIKDIGLAITAIWNVFLLFYICDKAHYASFNVRTNFQKKLLMVELNWMNSDAQTEINMFIRATEMNPSNINCGGFFDVNRNLFKGLLTTMVTYLVVLLQFQISIPTDSGKRMNISVAEFANDMLMDSAEIESTTTTTTTTTTPKPKITTTKAPARGRKGRN

>CcapGr4

MSKCIICTFLLQHIRVSFTILIYYITSIVLIIGQCFSLMPVLGVGQPNPRHVRFSLRTVRVMITLLFLVASTTLNLSMIKHLAKIGVNAKNLVGVMFFTCVQSSSILFFSLAPRWPRLIRFWTRTEMIFIRKPYERPQPDLSSRVRRAAATILSLSAVEHLLYLASAVVSQYRRAHLCAALNNTTVHFTFEDYTYKNYDYVYELLPNTTAVGSFILVGNFFCTFVWNYMDLFIMMVGKGIAYRFEQIKMRINKLLDKEVPESIFMEIRDHYIKLIELLEYVDEDLSGIILLSCANNLYFVCYQLLNIFNKLRWPINYVYFWFSLLFLIGRTAFVFLTAAAINDEAKEALGVLRRVSDKTWCVEVERLIFQMATTTVALSGKKFYFLTRRLLFGMAGTIVTYELVLLQFDEPNRSKGLPELCS

>CcapGr5

MSQKLQEQTFHKSVSKVLLVSQFFGLLPVSNILADDVQRIHYSWCSVHSVYSGAIIILNTLEFCTVGYLVFKAGLNFHNSGTISLFVVCMLEHFYFWRLATQWPLIMRVWRRTEEIFLRPPYHIYATYNMKARIYALSSLVMCSAMVEHSFLVFNSFHHSNIERVQCKYNVSFWESLYRRERPHLSRVLPFSIWYLPVVEWINLTLAYPRSFTDAFIICVSVGLAARFHQLYLRIEAVHNKALPTLFWTEVREHFLELLHLMRLLNDNIAPLILLACSNNMYFICFQLFNSFQNIGVDLIAVFAFWYSLFFAIFRTILTLLTASSVNDYSKKILLTLRSVPSTSWCTEAQRFSEQLAFDLTAWSGCGFFFITRQLILAMASTIFTYEVMVTDVINKGAIQQVTNYCRPIEYDTQSE

>CcapGr6

MAKVNPVEVSHEAARQNTNTMHLALRPFMMISQLLATLPVTGTWQKSSLEHVHFSWCTLIAFLSLIMITFSIIDVVLSTKVVVELGLKLYTVGPFSFSIISALSVSSFLQLARKWPNLIKHMDRCEQIFLQKSYGNKESRNFSHNVRKFGGILLFGAALEHSVYIMTAIFNNDFQIKQCNLTVDFWKNYYMRERLQIFSIFNYHAWLIPLVQWITISTTFAWNYVDIFLSMIFKCFAIRFRQMHWRIKRHVNKHMEDDFWHEVRNHFMVLVELLHLFDDGLSTLILVSCCNNLYFICVQIFHSFNNRDTFIKEFYFWSSLLFVLLRILTMMLSASAVHDEANKIMSTMYEIPTKFWCLELKRLNEIIVHDLVAFSGKSFFFLTRRLIFAMAGTIVVYELVLIDQVEDKDVVTDFCTSRNV

>CcapGr7

MDLKSWSAKPKIGKRHKRIIGVLRVRNIFRRGTKLDYQHSGSFLEAIGPVLLLAQIFALMPVCGILSKSASELYFSWKCVRTLYAMIIIFCLGPASLCTIAFAFRESFNFDTIEAIVFYVSIFLIALAFFQLARKWPALMVTWESIESKLPPLKTEMQKAALAHRIKMITLVATVCSVVEHLLSMLGIIYYVNGCPAMPGHPIQSFLFSNWAQFFYFFEYTNLAGIFGKVLNVISTFAWNFNDIFVMAVSVALSARFRQLNEHMLRAAKQPTTEKFWMDNRINYRNMCKLCEATDDTISIITLLCFSNNLFFICGKILKSLQKKPSWSHSVYFWFSLGFLLMRTLMLSLFSAEINDESKRPLVVFRSVPSKFWCPELKRFSEEVTTDVVALSGMKFFHLTRGLVLSVAGSIVTYELVLLQFNKEDKVNDCYEG

>CcapGr8

MKFTFKVGWKIGNVTADARLRVQQQQRKLKTQRLWRRQCERAKRRQSQSGSNAATKCDPLAQKHNRKALLIKRTCTEFIAQMELLHRQIDKPPTLPKSKKSNFQQDGSFHQAVGKVLLFAEFFAFMPIKGVTTAHPRQLSFSLKNLRTWYCLIFIMTTTIDLGLTMIKVLPKPINFNGVEPLIFRLSIIVVCSSAIVLARKWPALMLDWHEVECDLPEYLTQIEKGRLAYKLKMVTVVAMALSLAEHLLNITSHIIYSNSCPQTNDRIRDFFILTNQHLFELFPYSIYLALWGKTQNILCTFIWNYMDVFVMIVSIGLAAKFKQLNDNLYKFKGMRMPEEYWSTRRKQYRNLCELCTRVDAAISVITMISFSNNLYFICVQLLRSLNKMPSFAQAAYFYFSFFFLIGRTLAVSLYSASINDESRKPLRILRCVPKESWCTEVKRFSEDISSDLVALSGMKFFYLTRKLVLSVAGTIVTYELVLIQFHQESKLIECNSPFMRNGSRH

>CcapGr9

MERGKKSNSIIGHIKCVLGMKISLEAGYKRNQPQNQRKQPQITMDIQPSTHKQPTKQTRSKLGEKLEKGQEGNDSLSRRKGKTLLVIAQWFGLMPVRGILAVNAKSLKFSWVSFRTCYSVVYVILTSIDTGLTMNMVFRGAMEVKNISPLVFHVNALLVAICFLRLAVKWPQLMLKWQRVERQLPPHQMWRDREALALRVHKVTFVLITLALTEHLLSVVSGIHFAIHCSPNSDPIKSFFIAVSPHTFLIFNYSTWLAWCGKLLNVLNTFGWSYMDVFLMIIGLGLSSLFGQVQNSLNRVKGKTMPEAYWTRTRLQYRLICDLIEQVDGVVSGMIMLSFANNLFFVCIQLLKSINKMPSISHFVYFYASLSFLLGRTLAVSLYLSEVNDRSREPLLVIKHVPREGYCAEIERFGHEVAVDKVALTGMQFFNITRGLVLAVAGTIVTYELVLIQFHEEENLWACN

>CcapGr10

MFFLTCLTRKGGIRRATRADFIHNGGFHEAVGPLLVIAQCFCLMPVRGILAATPKGLSFRWKCFRTWYCILYILATIADTGLTINMVVKGVLDVRNIEPLIFHANILLASFGFLRLAAKWPQLMRKWQRVERQLPPHQSWRDREALSVRVHKVTFVLITLSLTEHLLSTISAIHFANYCPSRVDPIESYFMTVVSQIFFVFDYSTWLAWYGKILNVLNTFGWSYMDVFLMVIGVGLTSLFEQVQKSLECVKGKVMPESYWTRTRLQYRLICELIEQVDASISGLTMLSFANNLYFVCIQLLKSMNTMPSVAHFVYFYASLSFLLGRTLAVSLYLSEVNDRSRKPLRIIKLIPKEGYHPEVDRLCHEIGMDTIALTGLQYFNVTRGLVLTVAGTIVTYELVLIQFHEDQNLWNCN

>CcapGr11

MEITEPTLCIFYVNKFLALAPFSVRRNSKGALDIRRSVMFSVYSGSLCLIMVILTYQGLLFDANSQVPVRMKSATSKVVTALDVSVVVLACSAGVGCGLLGYRATRELNTRLRKIDDSMHSYSNFKRDRTMAILMMVLPLTAITSILGLDLSTWLRFAIGVRTPQDDTELNVQWYIPFYSLYFILTGLQINFANTAFGLGRRFRRLNVMLRNSFLKDNNQKDAPMKPPITTVKVVSQHPLALHQSLAKLTNDTLQGSGKQKVNLLRLLEENHESLGKCMRLVSNSHGFAVLFILVSCLLHLVATSYFLFLELLSKKDSGMVWLQVLWIIFHALRLILVVEPCHLATVESKKTIQIVCEIERKIHDPILAEEVKKFWQQLLVVDVEFSASGLCRVNRTLLTSFSSAICTYLVILIQFQNTNG

>CcapGr12

MEISATSRSVFYLSKILGLAPYALKTNTKAEIEIKRSKFFTTYSIFLCLSNVVLIIMGFKENCKVSLRANSTTLRVTTAIDVIIVALASIAGVGCGIFGLRSTQELNQRLRKFDSSLQLFTITKNERNKCGAMAIITLAVCVGLLCFDYFVWMEILSGQHTAEQKLKATLMWYLPFYGRYIAVIGSQILYANNVLGFGRRFQRLNKILEYEFLSEQMSSSHHQLTAELRCKAKTRAHILRTLAYNYGSVSKCVEIFSNTFGFSTLCDLTSCLIHLVITAHFILLALKNPIIDRYLINLTMWMVLHIFRLLLVVEVCQMATVQSEKTKQLVAEIKRTHYERLLAVELKSFLHQLLVFEVKFSVMGIFKINRNILTAFGSAIATYLIMVTQFQNSSG

>CcapGr13

MEINEKSLSVFYLSKFLALAPFSLKRNKKGDIEIKRSKFFTLYSIGLCMAIVIVMISGFGEYYFGPLRKHSAPIKMVFALDVSIIILTLVGGVGCSLLSIQPTQELNSRLRKIDAFLHLFPSHKNDHTITKILATLPIILTIGIFGFSCFLWMRYPLADFDRKHILKTAYHFYVPFYVSHLVIIALHIMYGNNALVLGGRFHRLNTILKYNFLYGKRYIPITWLINPGLGGGNELNTDAICKVSLLRSLADIYLSLSKCVDIFSNTFGFGLICGFTLCLVQLVLTSHFLILFITRAIWDFRIFNELMWLSIHLFRLLIIVESCHKAAAESKKAVEIIYEIRRKFDDPSLVEELEKFSHVLRAFEVKFSVMAAFNIDRNILTTICSAIATYLIMVTQFKNFKN

>CcapGr14

MEINESSFSVFYLSKFLALAPISMRRNIKGKIELKRSILFSLYSITLGVAMVILCYEGIIFDANSQVPLRMNSSTSKVVTALDVTVVVLSSVAGIGCGIFSVKPSRELNLRLRKFDAALHSFSNFKRDRIISRVMTVIPMAISAALIGFDIWTWILHVELAPKKGSKNSIKWYLPFYLLYFVMIGFHILFANTAFGLGRRFRRLNGMLKCHYLADSKPYSTIKTQLNSIKITPDHAMSLHESLNTESLPKESLGKTRVMLLRSLADNHESLGKCVQIFSNTYGIAVLCILVSCLLHMVATSYFLFLALLDRNVTGYVWGQVMWIILHILRLLLVVEPCHIATAESKKTIHIVTEIERKIHEPILAEEIKKFWQQLLVVDVEFSALGLCRINRNILTSFSSAIATYLVILIQFQKASG

>CcapGr15

MKHSNWFNNMPYNKVKPSAVQLHNIGYNEFLHRIPRDSSIFNDIRTTLFILKATGLLPFYEEISSYEVGPPTKPNIFYSYFIRGVVQALTIFNLYNLVTSGSAQLFSSYSDTDNVNKWIEFLLCMLAHSTTVIICARNSKSFLKIINEILKVDEDVFDRFGTAFENRCGFSLKFIVGICICQWYLIILTVLEASDSLNMNSYIFIMFNAVQNGMAAIFIVFAAALLRIVKVRFAHLNSILSGYTYNQQRKQEHFAGRERFRNTMETFPEESLFTYRMHNKLLRIYRSINDCCSLILVAYMGYAFYTITTTTYKLFVQITTQSYPQISYNVLQTCFTWLAMHTCVLALLSRSCGQVTDEANWTSQVLARVYSKSKDHQNIVDKFLTKSVKQEVQFTAYGFFVIDNSTLFKIFSAVTTYLVILIQFKQLEDSKTVNNSM

>CcapGr16

MHNCKRRTHPKSNTRDNEEQRAIHPTLAYLKWHLLLLKLYGIIPFYTILSLYEIGPPTRRVTRITRLMLLAKAVMNIVHVYIYLSSTILKILFLRSQTDGITNMLDVTFCMICDITITWTCVCNTTIIMEIINRFLKVDRLLKQYADSPAERPTSTNIFNRYLFFIYAYIFSLIISYLKRTYDLVSLYFWIYILFYQLENAISCAFVVFISALMHLLAERFRYINQLIAQYNRREIVRSSEYYTHDRLMPASDFDGKTTNRNEATLQLFATNSAIIYGLYVDLLDILKMINNYAGLGLLAYLLYACYGLLSCAYNSLLFNWSPGEDLYYMVWNFSWLPLYASILTILANNCAKATEEANKTPLILARVYGKGKKYQTIIDKFLTKSLKQTVHFTAYHFFPINRATLFQIFSAVTTYLVILMQFKELEKSKDKWNAEASKYNYE

>CcapGr17a

MKIINDMGSIRLVGQIFGLIPFYECVRGELRISIFGKYYTYIINTIFLLISLLVYLIVTKSGDEFNVFRNLRGADQSTEVLFSIISTVVIIVACASNTQRNCDIMREIGKLDELLLAKGFILHRYSCHILSTILALISFYMLGTACYYMQLLSSHFHYHQLVLLNIYGLQLFISNLYALCLRVLLGNIRQRIHLVNAKLELVAKSDLDVEHNWRQMSLFIELLCKFRFITDKVNTSNGIALFSYMSFAFYMLTNQSFMAFMTIVKPQGYEEKYDILGVSLAWIFVELVTILVICSACDDLSSEANRTSQILARVYGKGKEYQNIVDKFLSKSIKQDVQFTAYGFFAIDNTTLFKIFSAVTTYLVILIQFKQLEDSRIDN

>CcapGr17b

MTNSNRTFINVKSAKKGKPGTQTILSRLKWNLIALKVIGLLPFYTMRTRHEIATPTQCRIFIKRVIILSKIIMCILHINALLSPSIWRMLFVQSKTNGLTATLDVTFCMLCDMTITSTCAFNMDKIIAILNRFLKIDQFISELPTSDAAPQKSFKRTSGNKYIMWMFCYICLITIPIVHRLFETLSFEIVILAIAYQLENYTSYAFIVFISSLLHELTMRFQYVNHQIEKYNRKFPKSSEQANISSPRRLEHKTTTQDILNMSRFAENSVVIYSLYNDLLDLLKMVNQLADLALLTFLLYSCTGILTCTYTSTLFNFHKRNESFSLMWNVSWIVNFSAVVILLALRCDRVTKEANRTSQILARVYGKGKEYQNIVDKFLSKSIKQDVQFTAYGFFAIDNTTLFKIFSAVTTYLVILIQFKQLEDSRIDN

>CcapGr18

MQDETVSLNILHTKRQRYKLATAHTLVKDLKWILLYLKVLGLLPIFKITHTYQMAMPNNRSCAALYTHATHVFGFLLIISYIYIVCSPTANSVIFIYGEMDNATTAFQILLSITCYAIVFWKSANKAQPFLAIINQLLSVDQELQQYPRAPAALENKCDFYKGYLLMLLAHISTFWSMKWMNSHECNSSMICVCIFFIYLYMHAVINAYVVFVAILLHLLTMRFRYLNSFIGIYAAKRNAVGCAGVSTQCGDGTWAGCGGERTAEDVDFVVFSQDMIFFYRLHNHLLVIFETLNDYVHIALLVFIGYFLYGSTIAVYYFYFAAILRDDIQIISVIWCALFLCLHGPTAAVLMRKSDAAAKEANATSCHLARIYGRGLDQQKLIDKFLTKSIKQNIKFTAYGFFVINKSTLFKICSAITTYLVILIQFRQLEDSRAQNSKHRATDEL

>CcapGr19

MLRAKARSLKQALLATKRHCKAPRGKLTTAYCIFSEIKWLVYILTACGLLPFYEIISDSEIARKKGLFASLYTTIVRTLCLLVITTNSYTLFSLTTISYSFFGSVDLVNYVLHMFLNIFCFAVIIFSCAVKSRHFLRAANKLLLIDVQLQRYANNTDCLENECAFQWKYFIYLGIFTVITLQHIDFHKMYKTSYFLLFLCYYIETSICNFFIIFIALLCHLLFLRFRYLNRFVKSFTNGGRNQKLKQQLRAKHSTENPIFYITLPEERVRPKHEIQQESFLSVSSYIYRTHYELLKIYKQLNEYVGHALLVYFIYVFYLTSLAMYRMIYRAKSSKLSKFPLYGFASLVLHMSILVLVTRCCSKLTEEAQKIASIIANIYGRNEKSRKVTDKFLTTNLKADLQFTACKVFTIDNSTLFKLCCAVATYLAILGQFCLLEKSRSEH

>CcapGr20

MFQSNTHFRRVLQIERSIKLYTKFVMSFLNIIRALRFYNMLLRHLGIMPIKYCMRENKYILQKGWSFNITHWACQMVYLVLIGVLVIRREEFFKVEYSAIENNYWNVLVSAAFMAQFLINAWILALYRQHFNIISYCMYLMERLSFSAAQRKVGIFQSVMIAAVMGAVALNTYCMWQELAIPHSFLSICVYVTWQLYYIISSIILSIYLCIIQIITGHLNNIDTMNIVIVKNNHRQKRVTGIDLDNIQERLVAYDKLLLLCNEDISTSYGVVFVLITFISTLDITFIVYILNMTTAVGYLQTIGSTVKTAFFALPSIIFLCMMFLGSDIQEQANNTVRILAKLPRTGTGLDKMVDKFLLKNMRKKPILTAYGFFQLDRSALFKLFTAIIMYIMILVQFSDIENSLKKIN

>CcapGr21

MAIGNRENSRKSLLFHIFQFTVTLAGFCSAILACRCFPKSQHVYKFFAGYWILHTTFVLMYSVFFEYSHYNFDVSGIMYFLLLITQTLAQLGVLVEGFLMTAKRALLARTFTDFEKRFIREFGTNEGYTKFKREGWILTIICFCTILVTIPHTIISWTLYRTRVLKTLLFWHFLPSTLTLQFCIFDLMLTITHLNEYAAILRNDLNEMGERNIRRVFGHYLRGLATVKGSIVRVTPQKLQPSEEEDERRLRVYKKFYGDIYNMFKAISECFGWSLLFFIIMYFIYFVVNSYYVIQSIIVQLDESSTVLSNLGFLMIVVTLLWILCWQSQNSHEQSRQIGCIMFKLVKPSGNKSYNDLVTDFSLQTLHQQYVITAKEFFNLNLNLLGTMVASIVTYLVILIQFMFSEETRVERNLMENNRNCTVE

>CcapGr22

MDQLEACVNYLLKTTFVVYLINPLPRAAPWLNIFLLSAWLLFFINYCHLPIKMLLFSDRALVRWVRTCLSVGCAIISFCCMLETAVKWRQFAQIQQLRQQINYLISQQLKAHLRGRKRRSFGYLKTRLLPLLLILIICESIKFSSVKNMTNTYFGTVTIVMGLRLRYFHALSLMCEFNEYLCVLYETIDLLVTYNNTRPICEFNVWRPHNRWELEQLNLLRLLYGRLFEFFQLINDCAGWSMTLIAQLALSEFVCYTYWCLTYKLVNLGIGAVIFNLATIISLGCLHYQWFGLAEKINRKGQNIAALSTQLSKPLGSRRYNDLLLTFSIQTLHQKLIVTAKDFIRIDLKSLSATMHVFVNYLVFLLQFTYIKTKA

>CcapGr23

MFQCSRRPPILQSHLGMHSDINRKDKCLKQSLPLNTRSMSNRNIHASFARYCFLLLMYTSLLQPIPRLGRLMFILRLAWFVWLAISFTAVVYELLTNPYDSVGSIVGSALFIFNLLTNVVTFIESICKCEHYQELLRLEREVDTLLVNKIDVQTIIEVRVWHMAFYVVLIQIIYDVVQLVIAFTGYKSPVFYYVVVILFIQRARYAQITCAIARLNARSLCLITLLRVLVKANRPRHKYSSEVWQPYAVWEFEYLNLLRLIHGRLCELHRCVSNCFSWSIVILLFSTFFTTVSNLFWCIEIIRTNFQFGQFAYDALTILRLDTLAAVLLFTAEQAKKHNMLLGGLFLNLAKPLGNKTYNDLVSKFSLQCLQQGFMINVKGFFSLNLSLLGDLIVLSSTYLVILMQFDLYQGSNTKVNVIYNSMANEESMKTEKFPTIN

>CcapGr24

MDLIDSLKPLHISMLIGGLSQYLVDRKTGKVYRSRLLESYAICTFIISLAVLLYGTFSDNEFFRKSDNDIGHTVDYIQMIGIRVSHIVTILEAFLQRKGQGTFVKQIQEIDRIFECSLNVDVENRSLRRKMLRRGAIMVFIYMSTELLILIAYFFTKDRHFSIYWLFYLLPFCICGVRYFQIFAAIMIIRQRLDKLVETLNELNLLKAKPMQQMSEDSKTNIKFITATLPVYETTEYLKRKYNMDNPDMKRLLIIRDLYNRLWEVTVTVNNDFGVSILTNVGNDFISITSNCYWIFLNFKSYSGTLQDFLQIASSAIWSAPHLYNVLMLALLCERTIQKTTAIALGLHRMETNVWNDNHNTVIEQFSLQLLHQKLTFSAAGFFDINCTLLYRIVGATTTYLIILIQFHMSADKLTTS

>CcapGr25

MEQQLRAWLRYSIVIGLYVSPAQETTHFSSCKVAKAPLAGQRQAWSTKKSNVQLIKRIYCYAFLFGLCVIFVNGLASQKSTPGMVSWTASTILFASQAASTMFIAWEGYWRQQEQETFLRLLQEIEFSLKLRLKQDVRLDWFVSHMRWLFYYLLWLSIICYTCFVYYFTTAQYVGYFWHTTLYNITMRLRLIQLLIYVRVLQHYLECLSMKLRQIVAHRLAPCRQLLDVNYEKLQSLEYLVAIKEIYGLLFKATQLFNKFAGWSLFSIIVGYMLDYGCALCWSVLSWEGFLESHNYYVPCLWWICPMTLILWHLCHLCNRCKQLDRLIANLSCRVIIMHSSHSLHTYRIVLHQFAIQLQLQRIEISARSFFTLDMRLIMSIFTSIALYMVILIQFINIGNKYI

>CcapGr26

MDHHLLAWFRYAVILGIHTNDLDNYAIDLIELKTTLRRLFMHKLKLLICRCFNYLLLLCVFAVYINGLYWRKRISGIMLTWLVATILFTSQALTNLLIIIETIWKEQEHEAFLLLLQRIELTFKLRLRYATNKRLFAAKLRRYLYYLYTISLAGLVLFLVTTFWLQYIGYFWYGLWFIITMRIRIIQLLVYLRVLQHYLPCLALRLSGIVAYRMTPNQQILDVNNKRLASLDLLLAIREIYALLFEAFQLLNEFAGWSLFSIITCYMLDITCNIYWSLLSLDGFARRRYYYISSIWWLVPMLAMVWYVCQLGESCKKLDRLLASLLRKIFVANSARFVCDYRLVLQQFTMQSKLQHIEVCAKNFFILDTRLVMSIFTAVAAYLVIIRQFLNAQQIN

>CcapGr27

MEEQLCGWLRVCIVFGIYMRPPNDRVGDINGKASIRSTRQLLPPQQLYICLIALTLCALYIHGLCWCGSLPTLQLTWVASLILFSFQVLTNFLILMETVWRHRQHAAFLQLLAQIEVALKLRLRQCTHSAALQRNLCYIIVRLVVFSLVTLILFMITSIWLNYIGFFWNGLWSILTMRIRVIQLLLYVRMLQHYLECLCVKLQQVVEFHVSPQRQLLDIDYSRLTTVEYLLAIKEVYTLIHDAFQLLNYCAGWSLFGIVVCFMFDVSCNVYWTVLSLDNWQNRRYYYIAGPVALLPLLVIICYLCVLCGKCKELGRRIIYLLSSIKVLNSSQSAALYRQVLHDFSVQLHQQQIEVTAQNFFVLELRLLRILFFVTATNFVILLQFLILEIEMVGLTEPN

>CcapGr28

MEQQIRAWLRYSIVLGLYVSPTEATPYIHNSNHCPANTSALRGERQMWLKQRQAFQRERLKRIYLRLLMPLLCVLYVNGLLRRKPVPGTMLSWTVATIFFSSQALSNFLLAGEALWRQQEHETLLRLLHEIELSLKLRLRQDVRLDWLLPRVRWLLRYLLWTSVLFFVLFVYNFVELQYIGYFWYSIWFAIAMRLRLIQLLIYVRVLQHYLECLSMKLSQLVAYRVAPSRQLMDVNYARLQSLEYLVAIKEIYDLLFKAFQQLNDFAGWSLFSIILCYMLDYGCTLYWSLLSWEGYLERRNYYIACFWWLLPMSLITWHICHLCHSCRQLDRVIASMLCRMIIKRSSKSLRSYRILLQQFSTQLQLQQIEVSAKRFFTLDMRLIMSICTAIATYLVIIIQFLRI

>CcapGr29

MEQQLRVWLRYCIPLGVYLRENNENTAKIITIRNNKRDRDHKQEAWSQTRQQVTVNARLQQLHVSFLLCTICVLYTHGLYWRGYIPGLMLTWITSTVIFTSQICTNLLIIMETIWKRSQHETFLTLLDEIEVSLKLRLRQDVQRLALCRNMRAHLVHLLVISLAAMTLFIITSLWLNYIGYFWHGLWSIVTMRLRLIQIIMYVRILRHYLRCLCAKLCQIEAYRRAPDQQMLDINYERLASLEYLLAVKEIYSLLHRCFRLLNNFAGWSLFSIITCYMFDFSCNVYWTLLSFDGFARRRYYYIAGPAAMFPLIALICHLCYLCDNCMKLAQRAAHLLCRIQIMSNAAPLRSHRLVLYQLSTQLQLQRMEVTGKHFFTLELRFLMAIFTAIAMNLVILIQFLKS

>CcapGr30

MEQQLQAWLRYCVYFGIYVNATPSTERVSSTELCWATKQLYRKRNFYHPRAYIYFLACITCIAFALSLVWHTYPPDLQLNWIATCILFISRYVTNSLILLEALRKRREHDEFLKLLVEIEASLRIRLHWQVESRQLLAQIKGFLKFQLVLSFLGLLPFMVFSLIVVEYGGYFWQGLWFICTARLRTLQLLVYLRILRHYLRGLCLKLRQIMEFHMAPSCRLLDIDYGRLGTLKCLLAVKETYTLIHEAIQLLNYFAGLSLWGIIISCVLDLSSNFYWVLQSFDNFHGRRYYYLIDLWWFVPVTVLVWELCYVCDDCMRLLNVAYFQGCTLTQLLSKIIVSSTSGIGRNYRIVLQQFSMQMQLQRIEVSACNFFKLDMRFVMSICTAMAMHLVILIQF

>CcapGr31

MERQLRGWLRYCVYFGIYVGVYEDTSATTISETGGDTNNKIALCATQRCHGQRPQWYRGQQNNKIFRQQRLYLWLLTLVLYTVYFLGLYTRSSYPGISWVAATILFTVHVLTMTLMMREAWCKQAQHDLFLQILEKIETALRLRLRHNVQSAQLLCDLRRLIVYLSVISALGFALFALTTMWLSYVDYFWFGFWLIIIIRVRVIQLIVYLCILRHFLRCLCAQLRLIVAYHTARGHTILDVDGVHLRSLKCLLAVKEIYELLHREFQLLNEFAGWSLFTSISSYLLDAICTLYWMLLSLDGFVRRRNYEIAGIFVLLPLALFLWYLMFLCDNCKQLGRTVAHLLSKLILMSAKNPSLRLHRLLLYQFSAQLQIQEIDVTAHNFFVLEMRFLIAICAVIVANLVILLQILKS

>CcapGr32

MAEYEYLRVWLRCCTIFGIYVSSRSEHALREASQRQPTQRRAVWLSEPLRITFVLERMYVALLALIACTLYVHGLYARKIEDGLALTWLVATLVYTTQLLTHLAIFMEALWKRVENETFLQLLAEIEVSLQLRLSCDTRQHALRQSLRRWLLGLVTLSLVGFVIFTSVSVLLNDIGYLWHAAFSLLTLRMRILQLLLYACILRHYMECLCFKLRQLVAYRTAPERRVLDVNYEKLESLTFLLAFKENYALIFKAVGLFNNFAGWSLFGIIFGYMLDFTCHVYWSLLGLDGYGSRYTFVVGLPAVLPFSVIVWQLCFVCDQCKELGDNISHLVSRLVTVTSKPALKRYCAVVHQFSTQLQLQRIEITAQHVFVLDLRLIMSICTAIATNLVILIQFFKTEHAYD

>CcapGr33

MWQRKIKSMQPQLQSQSQSQSHSQLQPVSLDTFTLLQLRLFQLLGLCSVPLQSQADLAMANPRPPTTIIHLWTSVNHDSILYNSDSFGIFNDLLKFIATIISHFIILSETIIQRQYMEQFLLAYTRLHNKWSSHTFRPEYAIYRKYVWRSSICIVIVIIIDLAYIQEISKNRSWLAFFIPFVPSGLICHLRSLQIMYFMDMLRIEVMQLNRNVERLVTFSERHCVENSQRSEYFVRIVCAELQILMECYQNIYELSMLLKKAVGISLTCNYIKEYVMILSECYWSYWMVYNGQDVIEYSLMTPSALTIFLLLITSRNCMRSTNFLAHNVHKIRHDVEDLNISTRLQSFALQISHQRIIMDGFGFFVLNCNMARDILGSIATYMIFFIQFMPKFKTF

>CcapGr34a

MSLQVPSVVRFHIRLFQLIGCFDVSLHTQPREQRLAEQRLVAWTVLILLIFTLTIVNTFVHPSAFLFTSESFGYFVDALKVCMAHVAVTIIYLETIARRHALRNFWQRFAMLNVELSKNEKYLPAEESDWRTQIRAYNLFIYIFYGITVFDLILQIIYYNLREENEHLLQFLAMFTPYTYMVHLRNMEIIFHIAIIRHELEKLRKDVALLADYTRFSRRVAPFVGFESFVRQKLAEKQLTFQRIYEMYYYFQQSFGVSTIAVLLMTYTRLVVDAYFILYTYHQKKEPEFIENLLMLPAYLEIPSLLLTSQKCMNEVKFIAFELHNIRSSVDNSLISIQIQNFSLQILHQKIRIDGLGISALDGKMLVSILGSITTYMVFFIQFMPKFKNL

>CcapGr34b

MSIQVPSVLRFHIRFFQVIGCFDASLSTQPTQQRIVEQRLVVWTFLLLLIFSMTTVNTILCPDAFLFTGNRFGYFNDMLKVCIAQITVFIIYTETVLGRYALRNFWQLYALLNQKAEVAEKLTTPKPHWPLQLCTYRRFLCIFYGITIFDIIIEILFHAMRPAGEDTMIVMRFWFMFTPYVYMAHWRNMQIILHIDVIRHELEKLRNDIALLAEYTRFARRVVPFVGFESFVRQKLAEKQLVFQHIYEMLYFFQRAFSVSTMAVLLMIYVRVVVDTYFMFFNDAVGWRFLENLLMLPAYLEIPSLLLTSQKCMNEVKFIAFELHNIRSSVDNSLISIQIQNFSLQILHQKIRIDGLGISALDGKMLVSILGSITTYMVFFIQFMPKFKNL

>CcapGr35

MDSARSTESALIVALTPYIRTFSLFAVSMPPSIILHTIPTNRRGLLFIIRTLFALYVIIHLLVSFWIIYVYIMLIGTFLLENSLDLITCVFSVGIIIAQLLVQIAVYTQAVTGHYALRNILITGVQLEKDIRKQFTIGCSLSSIRWRLGVRAGLWLLICSTFVPYLSYRLTPQKLYPLKRGIIVFFSCLIQIKGVEYCVGVQLVQELLLLVQQQLIHLRHKLLRCERSEARCILYIELQANQQLLARIWNLLNQVERYFCTPMLMLFFYNGFAIIQAIHWAYINFLLDDLDLRLGRIVHTVMLIVALLLPCYLSQCCIDEYNRFGTMLHKLKTVGIDENLSMRLQEYSLQLMHQRMLFTCGGFFDINLKNFGAISLTITTYIVILIQFKLQAETENKSALGARFE

>CcapGr36

MRPATYTSSALVSALTPYFWAFSLFAVVLPPCVILRTVPTQRQWSLQLFRAFFALYILAQLATSCRVTYINSAMISRFVVRSSSDGITWLLSLGINVVQLIVQIVLYYQALTGHQLLTNMLFNVMELERDIRQHCKNECTLASIRWRFRLRIGVWFLIICIVFPYLNYELATTSLTPLPRVLTVLFCTIVQLKGVEYCMNAQLVQELLHLVQKQLLHLRHELLRCEDVERRRIIYTDLQTNQNLLSRIWDLLNQVERYFCIPMLTLFFYNGFSITQTIHWGYINFELDDLNLRLCRIAFAVVIITTLFIPCYFSQCCIDEYNRFGTMLHKLKTVGIDELLAMRLQEYSLQLMHQQMMFTCGGLFDINLKNFGAIILTITTYVVILIQFKLQAETERQSSFETRYE

>CcapGr37

MEKLESKIFDISLKPFRRLMVFLGIIPYTNYLTHKRGFCYKKVSKLSLHLLAVFTLTGFLINEHVKRLQEVIYNLDDIFSSVMDGLGFISLIAVQLIVHLESTWKSQIYQNIFNKFEQMRTLLLQKFEMEFDFPKLKSCIKALRNHLAIYCTFLLFFVILLALQPIPTVRFLVCLHAEIVLKIKIFEFMYFTVVFIVMLGDLCRAAKKQCRLLETARFENTFQQREYLLGFIALQDLHALLWENVQLLTDYFEWSLPDIVSSLIKYYKMLFIFVHAYFIHKDLIVIWLIRLSTLTMACYLCTMCERRDRELRSYLRNCLKDRRNQPLMKCFHRISMQLWQEPIKFDAGNFVTINMETLGKYFFTVSMYIVILIQFRLSV

>CcapGr38

MAHRTVQPILVHFGTLFMFCKVLGLYPHDLQAFQRMHTLQGSKTGTAFVCATILAVVVAYNFLIYSFADEDRELKEAQSTLTFVIGIFLTYIGLGMMLTDQWSALRNQSKIGELYERIRAVDEQLLRENIVVDISKTSQRILIMLALTVICEVTILVSTYIILVDYTKWISLLWIFSCFPTFYNSLDKIWFANTLNALRQRFFMINTALDGMVESHEKLKRWANTGGDSLLSTRKPSIANVSIDASIEYLYKDFTHMEAVKKFNMPRNKITPIAHSLNFYGESFETPKKPYKLSIQPPSFNMVYESELNRDIEKVEENLNNLCQLHDEICEIGKQLNELWSYPILVLMAYGFLIFTAQLYFLYCATQKQNIPSLFRSAKDPYITTIYLCYTAGKCIYLIYLSWKTSLESKRTGICLHKCGVVADNNLLYEIVNHLSLKLLNHSVDFSACGFFTLDMETLYGVSGGITSYLIILIQFNLAAQQAKDAIHNADESVSAMPTTDGGNTTALMELFTTTFLSSTHTTPY

>CcapGr39

MSEVNKQQSKPFLQSFNQIFYTCKILGIYPYDFHKFYYQGILQGSRTSCCVVIAVIFIIFVLFNITLFTFGDQHVVAKYNHLNLIINVVFTYMTPIAMFTDEITAMRNQKLLPKLFARIDYVDEDLGQLGITVNNKRVYRRIWLMIVLTFVCEFYMFFAMMWLIVDEFEWFNVLWIFTSIPTIYNSLDKIWFLGILLGLRDRFEAINTALDDITEEIEKNNLQKRKGLVYEQSVGVGELMLHTSLSVGSERISDIKLERLVCNAFGELVCERESNKSAAVIVSYENSSYNFAALQERFISLCQLHDSICRIAQQLNQLWAYPLLVLMAFGFVFITSLLYFAYCATWNQSIPLIFSPAKNIYTSFIVIGYIAGKCVSLMFFSWQTTQASRRAGICLHKCGVAADTNEVYEIVNHLSLKLLNHDINFSACGFFKLDMGTLYAVCGAITSYLIILIQFNMAGEQVRISKELAAANESTALTLAAENYTVMPLQTTMEVH

>CcapGr40

MDPRYTLFIEQQDFIFGLMPFNRTQVDSNIAVDYSLVLVMPLLYLFCYYTVNFSGFSVRHLPTCNSICSLSSKLLMHVGCFLYLTIYFLTLLRQKGFYIEFEKRLTEIDELVGECIKVADTRNKLFVPKKKRLYYFTWIIILATFAFAIFYDINEMSQYYGPYCFISNMVFTFPYVAGSIVQGMFASYVSVISERFSTLNILFEKINHESDKRNLPIAVMDIENDAHKDNTYSGPAVISDLARRKNRFQQKQKSVKSDDRSNNNDSDFAEDEDSADEAYSFDEYDETRLSDGKTSEENLPSLFKLHDKILSLSVLVNAEFGAQCVPYMAACFVITIFSIFLETKVMFIVGGKNHVMDYVIYLYVIWSFTTMMVGYFVLRLCCNTNAHSKQSAMIVHEIMQKKPTFMLGNDVYYNKMKAFTLQFLHWEGYFQFNGIGLFALDYTFIFSTVSAATSYLIVLLQFDMTATLKNEGLTPKVFSTESN

>CcapGr41a

MPLLRRAFRNLLDADNYYKSIQPLFFCTQILGLTSYVVSTNKSGVKSLTMSALSHIVALVQLVGFAYSSAYVVYFNATYIGQFLNTDITNGGEKSIVLASGATAVVVLITNFVRRRRLLWVLEYFPRIDKSFQRIGICWNYTKVLHCILSKIVFMICVLSIAFGIYVCCWRRLDVYPPLPIIFMSLNQQIGIYFTILLFSFVMLSTRVRMQALNKVLKNLAHQWDNSIVKPLPKQRSLQCLDSFSMYTIVTKDPCEIIQESMEIHHMICDAASTANKYFTYQLLTIISIAFLIIVFDAYYVLETLLGKSKRESKFKTGEFVTFFSCQMVLYLIAIISIVEGSNRAIKKSEKTGGIVHTLLNKAKNAEVKEKLQQFSMQLLHLKINFTAAGLFNIDRTLYFTISGALTTYLIILLQFTSSNTSDDHAAAHTHLNASSTAGIK

>CcapGr41b

MNSINSRTKLQLTTISALQPPPAAQEMSYRVQRMRRYFISKEVFATLQPLFLFTYIYGLTPFRIVRRRNGTSEIRASCFGYCNTAAYVILYGICFLNSLLSAESVVGYFFRTNISIVGDTLQICNGIVTGFVIYATALTQRSKMRRIIEVFNMLDLNYANIGVRVKYSRIYRYALLLTISKTLIIVIYCAGVYLLLRSVHVRPSICVCVAFLLQHSVLLVAVCLFCFIARGFERRLVILNKVLKNLAHQWDNSIVKPLPKQRSLQCLDSFSMYTIVTKDPCEIIQESMEIHHMICDAASTANKYFTYQLLTIISIAFLIIVFDAYYVLETLLGKSKRESKFKTGEFVTFFSCQMVLYLIAIISIVEGSNRAIKKSEKTGGIVHTLLNKAKNAEVKEKLQQFSMQLLHLKINFTAAGLFNIDRTLYFTISGALTTYLIILLQFTSSNTSDDHAAAHTHLNASSTAGIK

>CcapGr41c

MSQSRRADEDANSIEAAAAAAVADQDVCATHPQRVSGLRRFFHAHQLYESVQPIFIITFWHGLTPFFIKSDGTGNKKLKESIFGYLNTLLHITIYVGCYVMTLMNNFETIAGYFFNTGISRFADTMQIVSGLIGITVIYFTAILPKHRLQHSLRIMQDMDVLLHSVGVKILYSKLLRYSYLSLLVVFAVDTVYSCGNFLLLKSAELEPSTPLYIVFTLQHTVISIATALFQGIAKMLELRLVMLNKVLKNLAHQWDNSIVKPLPKQRSLQCLDSFSMYTIVTKDPCEIIQESMEIHHMICDAASTANKYFTYQLLTIISIAFLIIVFDAYYVLETLLGKSKRESKFKTGEFVTFFSCQMVLYLIAIISIVEGSNRAIKKSEKTGGIVHTLLNKAKNAEVKEKLQQFSMQLLHLKINFTAAGLFNIDRTLYFTISGALTTYLIILLQFTSSNTSDDHAAAHTHLNASSTAGIK

>CcapGr41d

MSFLKALFSPSDAYGAVQTVIWFNFLLGLTPFRLHNEPTSEQLRTLRISQLGYLNTLLQVIFFMYCFIHSLADQASIVGFFFKSEISQIGDTLQKFIGLLGMLTLFGISSCECRVIVSLCDTVAYVDKRFLNIGVIFNYRYIMKLTHLKLFLVITLNASYMSSCFWMLFHNDIWPTFQAMITFFMPHVFLLSVVVLFSTFLFRLQQHFDLINKVLKNLAHQWDNSIVKPLPKQRSLQCLDSFSMYTIVTKDPCEIIQESMEIHHMICDAASTANKYFTYQLLTIISIAFLIIVFDAYYVLETLLGKSKRESKFKTGEFVTFFSCQMVLYLIAIISIVEGSNRAIKKSEKTGGIVHTLLNKAKNAEVKEKLQQFSMQLLHLKINFTAAGLFNIDRTLYFTISGALTTYLIILLQFTSSNTSDDHAAAHTHLNASSTAGIK

>CcapGr41e

MFRRLSIGWRYLQRDLKHPRNIYASLRILFLLAHFYGLTPYHISVSPKTQRHSLHTSWFGYFNMATRCIFLHCNLMYSILHNHTVIDYFFITNVSHFTDSLQKFNGILGNLIAIAFCFFQRRQLCTLMQRMYRADERFYNVGVSFKQKFIAWRTNRFGFGLFALTICYFQLSMLGILARNKVYVSLPAAVSFYAPHLLVCGIVVVFNGFIYKLTQCYRALNAVLKNLAHQWDNSIVKPLPKQRSLQCLDSFSMYTIVTKDPCEIIQESMEIHHMICDAASTANKYFTYQLLTIISIAFLIIVFDAYYVLETLLGKSKRESKFKTGEFVTFFSCQMVLYLIAIISIVEGSNRAIKKSEKTGGIVHTLLNKAKNAEVKEKLQQFSMQLLHLKINFTAAGLFNIDRTLYFTISGALTTYLIILLQFTSSNTSDDHAAAHTHLNASSTAGIK

>CcapGr42

MAWQFLKRLTQSDSILQSLRPLAYISLIGLAPFRLSVNKEVRTSVFSLIAGIVHFIFYLLCFILSLYEGDSIISYFFQTSITRLGDLTLSLSGIIAMVMIFSSILLKRNLLLHIIQNCLVVDDIFLRLGLKLDYRKIMLYSFVTSLGLLLFNFVYLLVSYMLLRSAEIWPSFVVFTTFALPHLNISIMVFKFLCTTYLARSRFRMMNEILQDILDSHIEDHDAVELSPMHSVIRMHNHNNPHNHTPIVIQPTGATRPRYSVTSLVRQNPERALKQVSNVHNLLCDICYTIEDYFNYTMLAIIAISFLFILFDDFYILEAIITPSRVDKFEADEFFAFFITQMLWYVVIILLIVEGSSKTIKESNKTAAIVHKILNISDDSAVQDRLLRLSLQLSHRRVAFTGAGLFKLDRTLIFTISGAATCYLIILIQFRSTPSHWDRPSSSSVAGATSG

>CcapGr43

MLVWSKMSRVVGCFYRLAYYYMLAFGLLRFEYDFRNNDVRRTRRVALYSLCVNLLMLSQIPQQFQMDISTYTQQSKARDIYQFLHSLIVLSRIFACLLILVFYWLRRYEIIALVADFLRLRRAQSRYLSPSASALRRFHFALLIKFSFSITGDLVRFLMSWRSTNWQDRIIMMFTAIYIGLNNQVFNHFFVVSRLINLYLETINSELQHLVSAASELNSQRIQRRHILPWRSAQLMNKFNALMSLRQKLQRFALRVNRLLGPQIAGVMLALYCYNVGVCYIIYSLMKPHEASLLDISAWNMVVLVFGTVVYIADVTEFLVISQRLRQNFGLCMPITLQLLELQQLEKNWDRSIEQFYLREACFTYRLMLLGFISLDNKMAFTQFESVISKSIILVQYDYKNV

>CcapGr44

MSQLITLCLKYMNYSAILVGFTRLRIDYSNQRIIESKFTSYYVIILNIVTVVLLPAAHVISIKYITVNFNNNLLVFTDLANLFINYAVVVFSVVSRWRRECLYKELSQEIFKLDLYYFNKLSENLQIEKRANMVIFIKMCTVSLDALVPIFGVLNQAKHVDVFVFMLAMYSSLIGTILHAVLFLFFYMLWHVRRRIWRLNARLIELFHDLQKLQRNARDMLAPKSRTLQQLSGLAAEELREISGVHARLTVMLLRLNRVYRWQVIAVLLTYLITNISYGYYFILSLLAPSNSAQSQTASSIVVSIAASFIVFVDINLLYWTYIMDAQMKTRDGS

>CcapGr45

MRMHKQSYQSLYNYIAISLGMTSFRYNSITEKHKQMFWTQTFALLANVCTIISLPLIFWNAIKRVTAIQWNPTMTYATYITCTIRALVVLYTILKRSKIDLMIHESLKKLRTLQRTYLAHFPRVSSIERRMNRLYYAKFISMCLLLTLTAIIYHQAAEFEFTWLNCFIFAMSLQTTAILYITRFNFFWFLWSICSSLRYVEWQLRHLLAQMNESLELGQRWRLTALATDLEGILEAHWQLAKLVILVSLNYSVLILAGLMEQITSLVRQLYYGIIFTFDHRATNSQLTIGTLYFCALIFDMYLSLLVCDMTVKTYYASVEQLRCTNVMNAYCPALYEMLERWLLRIQCDGFRVDLYRTITIDKKRWLRIMQRVFYESITLVQFHLIFNANKNFN

>CcapGr46

MSSQLRKINKLSSLAEVCFTHAAIMASVKSIVFYSWYYFLLATGATSLCFDFRRKRLTSTMRRRYYNLLPNALLLGFLPFYYLKGMRYLDAYEEKPLLVTVGHFNIQMQTIVIAYTCVIHYWHDVEYYSIAKKLLYLERRYFERWSVYAQGPSYNWVFWLKFLSYFSQNASLIMGPMTVSAKRLLNWMDLLLNLYYFFIWNYLNSIIFSYCLTLLHIVRRYDVLNMHLRRELQRLSAEVLGMRSELSELRRVLQVHEELQVIVTGVNNLYRFHITWLLLIFFLSNTTAFYVGFIHLANWSTCHMFVLLSFSLFAIKSFDIFLINHCCELLVARHSVALELLRRQQHHLHAATEYEQMCVNFGLNATVTKFSINIYGMFDMDHRSTFVALRNTVMHALILIQFDYIMDAQMKTRDGS

>CcapGr47

MRTARRTELRNCIIGYANIGAIILGITCLRLNFPEQRIDRPRLLRLYVFSVNLLTILLVPFAYVLEAAQLPQGYNHNLSMLTDGMTMLITFSAVIITVLLRRKHETLYVDIAEALFQLEKDYFSKLRIDNAVEQRSDYLLFFKVFVFAAHTIAPFYGALVFSPTASWLNMFASFYYGFVYNLLFGTLFVYFYLMWLLRKRFSLLNTKLSDLLHSIQIWLEADNGFMQLQKLGVCGAEEIMMISKIHERLSTLTERLNADFKLQILAGLLTQLLNCICYGYYGFLLNEGFLKIKVSTPMGVLGVMFTVIMFVDGYLLYWITDTTARSYQRAAQLLSRFPLIQILCVEFEQQCELFALQLKLQNQKITIGGMFSLNLQASLAVLGFSFRHILILVQFEYEYRMLTAAESPVLEKIISLLRLAELGWK

>CcapGr48

MSRTQIAWLRLRIIGYFNVLVIFLGLTRFRIDFVAQRVVHSRLVAYYVICANIGFLFFLPIAYIYEVIDDSTYFGNNLLSLTGSVSRAINFAASAFSVFLRSLREAVHLEIAETIIVLDRRYFSKMPMLEAEEKVEAAQQCIWADRLLYFKMFALCMQILATIYYSLPTFDELDANEAWLDCYYVYTQSILASTHFMYCYQMWLLWKRFGLLNVKVSALLRRLQRRTIKAGCGLGGSDLRQGRKQLALEAVDILRGIAQTHACLSHLLERLNEGYLLQILVVLLASVIESITFGYSLSLVFDGSLEWEFDFDTYLFCMFICIMFIDFNLVYWISDRTVYAHGQITEILRRFQLLPFLGVDFEQHCELFVLQLKQQHRNTKLAGTFHLNRSSALALWAFVLTQIIVLLQFDFGSRLKTATENAVMSRLNKLFRIQAV

>CcapGr49

MHRVTYWVLLIHHYYMLAVGIVRFRYDFVADKPHTTTFITYYSVVANVLTICSLPWIYHTSFNVFLQHDRIGKLALYIFVGSRTLRVLAVVIAIVHNWLRREELLTVIREFNFLRWTFFSRFTVAAPAQRHFEIKIVRNILSGLMIDVSGFIMILFAYSEQASSEFILSSIMFTFVSNILYLVINQYYFALRTVILFGNLMNQQLTDIMKFTEHVASLQHKHRNHVQFKARCRLLSQELDELSVVYWQLQTLAERITDIFKWQGLGVLTNMYIFNVSLIYTVVSMLWTGEFTVSHYDWVAVLMATTSVVYLLDVREFLGLQLEHMDIFNLKFLHLETYSSLPPLDKALERSVERFHMQLSINKYEMSLLGLFKFNGEVGFSMFASMLLSSILVIQFEYQYFH

>CcapGr50

MSFWERHKHNIYKYGHIYATLYGLMVINYIPQRSTNTFAYRLANIYGHALSICLIVVLPIYFVRNISALTAAHDQRGQLLQLVNFANTLLKYITVVVTYIANFAHYAAIRAVTRERQQLEDDFDGSLRAATGYDEHPRRQFEAMLLFKFALINAMMVVQVGNILFQHFTGAHSVRVYIAIYTFVLWNYTENMADYFYFINSSALKFYQQLNQQLRQVVREAKLLHYFRLCGQRRGTVPHFCGLLCDRLDTLVQRYQQINRLYQDSLTMHQFQILGMVFITLISNLTNSFILFNLFVKHSGASASPAIVLNALHAIIFYVDTYIVALVSENISLEMQNINQTLRQFNQFSMLDVRLQQTLEALTLFVMNNKVEVRICGLFVLDRRLTYLTAATGLSYFITLVQFDLNLT

>CcapGr51

MQPHAAPFWQLYTYYFALLIGLTAFHVDIEQRRVYRCTFMQVYACTMNALVVVAVPFSLWTSLEFLDYLNLNALMKIVNEVNVKQIFFITGLTIIFRWQRERSIYEILTQLLQLERRYFEKCMPRSVKAHCRQYYSNNLLWLKYFSVISQSLWTLYNMFFYLPDKSVISLFYCVYVICIISAVLHMVLHYFLAMWYLWQRFCWLNARLQQILDVLRSLEAHKRCDQRVRKRAYQRLGQELMEVVRVHYQLTGFAERITNCYRFQIITVLFSKVINNISITYLGLKYGNNSFLKSLGVTYHIFSTSFFIITLSDSYLLDILCERVVGASHEAAEVLKRFDELFELDDAVEYACSIFSQHLRQHKLKINIFGMMNVNKRLSFLVFAGFVKNVLLLLQWDLAKQFEV

>CcapGr52

MPAMKKAAAVPRWYLYTYYFASLIGLAGFRVDFKERRIRRCTLMQIYACTLNTLTVCFLPLAYNTALKFLDHLNDNHLMKIVNSVNTVQIFFITSLGLIFRWKRENTIFEILTQILDLERRFFEKCVPQTVKAYCRQLHGNRLLWLKYFSVVTQIVYTLVNLVYFMPKISITWLLYSAHMFLLISMLLHMILHYFLAMWYLWKRFSWLNAQLRRIFNFLRLLEAHRLLGDGLASDAQRRLAGELLDVTRVHYQLTQFAEHLTKCFRLEIMGVLFSKIINNISIGYLCLKYNNNSYLKSLSVVFQMFSLMAIIITLSDSYLLDILCERVVGASHEAAEVLKRFDELFELDDAVEYACSIFSQHLRQHKLKINIFGMMNVNKRLSFLVFGGFVKNVLLLLQWDLAKQFEV

>CcapGr53

MSIKANNNSCSKYLHSGIAKGKPRNSKKYKMLRERLHRIVLNVLYYNSLLVGLLPAPLDRGTQAFRSSRFYLIHTVFAHSFCVLVTTYAGYYYYNHGFLTRDPVLQWTYSVTYLTKNLLVVVLVKELWCKRKRTMAVYLDYWLQEMGLEASLAASRCIIAGERIVAGLNEEVTGNQTPLQHNRDPQRHIENLIIFKFFLAYSLVVMNAYYFLNQHPGTDATYLPITCISFGLNTFVLTLSGLFYCVLSQLYRQFSQINIQLCLVLEQLRRQSSEAAEVQTALASRINSLGMLHLAGYRLTQEIFAIGELTLAALLLRLLAANMRTVYGACYFLSKRETENLWSQAEEILFTTLFFSDTAMVMSMLNAVLSECNGAGKLLREYTEFMDVKGCDCLRKALLTVDLCSFFQLDAFSTHLGCHKLRYMVCGLFEFNKMASLQFFLTILVKVTVLVQFDMQNKLRNNRNDINVV

>CcapGr54

MVHPTGHLLTRRLHYFTLRIAYYHGLCAGFMPSHYNWHNGELHQSQLYLIYSGCIQLGFALGTPYAYRKYLEKSSEIFGDEVKHWTDILTNSGRYLTFTMLALFVWMRRWEMFELWSVFWRLERRYERILPTDDGNTTMHTRWLLLYYFWTQHIVTPLTSIYVLLQFPRQTNAHIVMTFVSWALSTVQVMLAYYTLCLHCHLLQKFEAINCELQRVLTLLRNLASMERTRLRLLTQRLSCLANAHFAYFRLTQRLLGFGQLPFLLILIKCFIVNMTGMHTCVWHIFHLSSLYNLVALLLVVVLYGSLAPSIAGLTWLLSSCNRSGQILRDANVLVALRDNYELMAVLDRFAVYLRSHRLRYIVCGLLDFDLQQCFSLIVVVLVQVTVLIQFDMEGEINKV

>CcapGr55

MWSRCRATFSNCIINSSIWLSIALGLLPYGYCRRRKMFVQSNFGLVYSVIMDAVMISLALHLWYKEQRFNFKRIWDWSVIDLLTQALYAVNIYGMLAVLWTNLREYKSLLEIFKKFSALEQSYFGKYQYLSPKCVKFHNYIIWKGLALFMNNFLYFTHVFTLLGRYQVLSVRVIFVQLYRMLLSTILLLLALHFYRHVLLIYRYMRTLTEHLKYLANYGMSNAQAYTRWHEINEIVRVYVRLQQLSREFTGIYGKQVFFGIVAITLDNAHNTIVLLLIWKSQTSVWYFTFIIYVIVINCIDFWLIIVACELAVNAARDLSLVLRCFNDTTQLDVEAERELQLLALNCASNKPKFRVCGFMDLNFSTGLEISIIFILMLTYMMQKYYRGLQIFKN

>CcapGr56

MMEYRYDKVNKMFNCSENIFMSFKYRSAVALLSVVEFEMSSRWSRDELSCCIINSTVWLSIALGIFPYDYTGRRIVQSKCCLVYTLVVDVALAVFALHLWHEQQYLNLWRIANWSLMEVFTQFLYAVNIYGILAILWTNSWEYTYVWKVFKEFAALERSYFGKHQSLSAKCMTFHNLLTCKGLIVLMNIFFYLLIFTAMLIKARFIRTRIFVAVFSKFLNIVIILVIMHFYTHVMVTYRYIWVLKERLKHLSNFDTPHAQAQNLWREINEIVRIYMRLQQLSREFTRIYGKQVFFGIAALTAENAQNVLVLLFMLTPPAETWKIIFSAFFIAKNIVYFWLIICACELAVEAARELGQLLRCFNAMPQVDVEAERALQLLSFNCIINKPKFRVWGFVELSSSMGLEIILILVLQIIYLLQSNYTKMSLFSN

>CcapGr57

MWAQCRRAISDFTLNSAIYCSIALGILPYGYNRPRRGMVPSKAALVYSVCIDLLIICMALYAWPSARLIHFERIRHWSLLELLSQFVILVNVSALLNIMWTNWSEYKCILGLLNEYSSIERSYFIRHTSLMYECAAYEHYIILKGLAMLLKNLSFVYVALVLFKDVTWPIVAVHIMTMLLVNIISLVVMHFYIFILVTYRYIWIMRERLKCIANDLGLPHSSNLQSIRYELSEITVIYMRLLRFCEQFVRIYGKQMLLCISGVAGVNVLTLILLLFVWREVVHKLNVAYSWYVLVVNTLDFWLIISACELALGTAFDFVALQRSFSNYAPLSAALERELEMLALVCATSTPQFRLCGLLDLDYSTGLKVLMTTILYVIYLVQMNYKNL

>CcapGr58

MWLDTAKKLFCAARKSVAHTCVSTQLLLSIAFGLFPYKYNSKTRRLTTAKWLKYYWVLVNIVMVLITVYLNFFKRTPSATSFIIDKPLNKLLAYIHFLLGLVVFVVISSANFRHRAEVLILHNAILQLQQQQQRWQQKWSKKSNPQISALFENYIIAKNVMTFLQAASNVHAKLGFNPNPSFKYVCFTILTICVKNITLFTVSNFYLTLLYIYRLLQQLNWNFKEVVSFYSLRARDVPDTVPIEDTTEIAFDLKFATPCKKTSVECTGVDVAAIAELCRQYVRICQLIRRVCKVYEWQVLLFLIIILFGNVMSTFYFLVYLVGKMLPKELFSPTLFLELFFINVLDLCCYMVICERSMMSAKETSFILHQLCRLEELPDEIRNEIEMLSIFMAGETIRIRFCGLLEWNFRTGASFMIATILYLIVLVQFDYYN

>CcapGr59FIX

MSFRLSKRISRAFVKTTLVCSVCFGLLPFYYSERQRRFISTKSITIYNICINVIIVVLMAFLLAVYASLDDAFLTGDPLAMILNRLAAYMTFSALLPMWWVNWLRRQRLQDLFNEFAAIEADFFYPYRHLLADCTTYDNYLLWKGLASLLQNLSIFYTTTVETPSILLIIFMCLLTILTNVVLLVATHFYLVVLHTYRLIWALNRRLEAIAADNIMPRLCQRRHLLSVEIDTMASIYARLISLCERYTRMHQLHLLLVIGSVTACNIEVLFYVRLLWSGKIPERTAFNVFAVFQIFVVNLLDFWLTITICELALVESRKTSAILRGFSAKPKLTLNVERSLECFAIICSSTKLRFHICGLFDINHLTGLKVLSTMILYLIYLVQYYHDNL

>CcapGr60FIX

MSFRLSKRISRAFVKTTLVCSVCFGLLPFYYSERQRRFISTKSITIYNICINVIIVVLMAFLLAVYASLDDAFLTGDPLAMILNRLAAYMTFSALLPMWWVNWLRRQRLQDLFNEFAAIEADFFYPYRHLLADCTTYDNYLLWKGLASLLQNLSIFYTTTVETPSILLIIFMCLLTILTNVVLLVATHFYLVVLHTYRLIWALNRRLEAIAADNIMPRLCQRRHLLSVEIDTMASIYARLISLCERYTRMHQLHLLLVIGSVTACNIEVLFYVRLLWSGKVPERTSFNVFATFQIFVVNLLDFWLTITICELALVESRKTSAILRGFSAKPKLTLNVERSLECFAIICSSTKLRFHICGLFDINHLTGLNVLSTMILYLIYLVQYYHDNL

>CcapGr61

MWSRWRTDMARFIYNATMWSSIAFGILPFGYNGKLQKISPSKYSLAYSVCVNVLQVVVSIPALPTEDFLNINVVKRNKWMDLLTRVITIANIYTLLVIVYINWRHYKSVLNIFNEFADIECQYFAKYGLLAWKCSAYEKYIILKGLATLLRNLSFIYFLSGLAKVVTWNILLVLALALLLGNVILFVVLHFYNFVLTTYRYMWILKRRLEYLSEHDVVHTHSRQLANEVYEIIGIYLRLIKISKCFGRIYGKQLLFSFAIIACGNIQALYYLGLLWADQFVGLSLLEIFNVLHGVSVNIFDFWLTITVCEQALGMPKEVTQLLRRFTIVAELDVEFEKSLEILSVVSKTHKLEIRLCGIIELNHLMGLKALLTMTLYLIYLVQFSYNDF

>CcapGr62

MSFKRMRYNQNKNINTHSEPFLALISKLYLLCQLFATAPILDTPKNNRRILRYVRYVWCSTLVTYITVVNISYMSRIHDPLVNIVKAFFAFELINNPLIVAFIVFTTYYYTAEYRSIPSELLRIYKVYQRLQANISAEKEFFRVLHMEVLVMAGVICSIILSTFVVDYFRSERTLMRAILHASVFSVANILISLHICQFWLSLRFISHLYRGFNEVLRQRLRRFRVIDLTALDECNNDLHAYGREISWYQREFQNTCNAMATDSFEILDMMRDIYFDLDTISTQLTNVFGITLLFNFLGSCVSLSVQFFAVFKFFDTENFLNDVQAELYNRILWIIIHLGRIMTVLISNNAIIEEKCRTSCVLNELQVSSKEMERSINRFLLRLMTHQPPEMLCGILQLDLLVLCGITGAVSNYFIFLAQIDLGTLPIQQKTTNSTA

>CcapGr63

MTPSNPRKPPNSKGHKQIRPSPLHRLMHVLTVFSQVFCMAPLPKASAGRLNLLRGFHYLYGLLILAHIYILSWKQMTLTDTFILTINKIIYFGDIIVNNLVSTLAVCSTFYYAGLYREVPQKCLQILQALKRLQGVQRSNKWNSLSVLYKELCCYCCCITLVTVIILAFTYFSLTSEYYIFLLQLGSYHLPNILLNLKLGQYWFTLRFAYHIYKRLNETLRERLKCFDLSGSTPSENEELPMFVSLNSQPINDGEPASSYTRAYLSTHSCGHSDAEFLEHIRRLHTRVDALMAELAEVYGSIVLLNYLGSIFAVAINIFELYQGFYRPVLVLNIVLWQIQYMIRILPVLNASNAVVEEICRTTRILNALYITTKKMQLAVNRFLLQLQVRKPAESAGGVVNLDLMLITGFVSAIGQCVTFLVQIDLSKVHYKY

>CcapGr64

MLPSIVRGSRIKRKSQKIRPFFFGPVGQHITVDVLETDLYRVVQHFLLLSVFFVALPYGAHQYMPTNRSSDHFLLFLHIVWCICIYICLVFAVYGEYTQTLMSLPTIQKPLYFAEYLIYLIHIFHIAFMGYLARDKCRRFLRTIAEFDLALMSFGKKPKYQRLKSFIGAHLFLIFIFLITTAVVDYFYRDCVVASSIRSLTVYLLPNLILCVSLVQYYTLLYAIAQRSIRLNEILHEEISRQKCSRTLQRIRLLYSALQVFTKDVNSAFAPSMVLVYVGSFSNASVNLFLIYKYVDDIQGVAVWWILYSVIWTILHITKMFLILFYNYDVQRQKNSTILIINEIGRTQNKEVEETVTHFVLQLTINTRTNVVCGVAELNLSFITTLLMAMSTVFIFLLQYDITYEALTLTHSTGGPIRNN

>CcapGr65a

MQMHLNQRDLTSNSTVESVLKFAYRISACLGINAYVYHSAVPVDSWYNKRRQQRRSGNSQLRNRQRRRPDIGRPQLPAHISQVYFSRSLRLYSLAILLMVIIAIPFVAYTMYLNQLFLRNSRILSLVGFGRLTFLHICAVGTLLRQYCKQQELITCTNRMLRLQLNIRKLVQQLGRKTLAQPVGLRTHCDEQLTFMLWLMLIPLLLSPTWTVFIVKIENVLVRPAYLAGLLILYYCQLVLQLTLCNCFVCTLILAQQSNRLNMLLRAALHPTARHANVPQPLNIARRCHLNTLTYKIRQLQRLFEENSQINGLLFRLYGPQLVAFLGFVLTECTVQSFVLYYVSCSPHTALPYRYVGETCTIRWNLWAVIYVFGLLCNMCLVVGAAHQLQSRVQYIRIVLAEGSARMPLVKAMCRHTYSEVQLSRTQPKKGRRYVYFMICGLFAFERRLLFVILQTILNYLLILIQYDKVTK

>CcapGr65b

MRLQKGKQLQRNTLQPRLIHRLEIIIYHMTTLMGITTFVRERTTKYREPRAAQPHALRLNKQQWHRHNRVSLRSSHALRWYSFILLILILTAIPFVLYTLYLDMSFLALTRLLAVVGNMRYVLIQGSAIIVMLINCCRQSRLLRFLNRLLRMQSNLLALTRHLRISGVTLADLQSFDRSTCVLLCTKVLSAMVPLVWTVYVIYKELAWQRPRLLASLLFNWFCTVFLQMMVVLYYFCLLRVVHYGKQLNLLLQNAISEARGHLDAVRPLRVTRRNRVFCLCRALRRLQCLYKRNSLSGRELVHIFTPSLLCFSFFVVVICTVQLFVIYFLTCSHKAGKFRTDGTVTHRDRCSSPNFVGVIQVLTMFGDMCVVIWGTHCLQSRLSETRLMLAESSIELSRSRDVCGSRGCEAQLERVQPKKGRRYVYFMICGLFAFERRLLFVILQTILNYLLILIQYDKVTK

>CcapGr66

MTQWADRRRRFYSVAFFISIRSILLTAQVMLLAPIIRSKQRGIYRTHRVLSYLSFFLLLTMCTAAPFLLRIIASTYASVGVQYDALFVRIAMISQVSDISIVLITILSQIWQRQRLCGFLNRMQDTVQRVRSIYGRNFVRLRVLLPLWLQLGLTLYDVLMQLVFLVKLAIKIDPWQFAVNLASLYLQQCRATLQLLIMASILLLTACYAQLALRLEDFEQSKIVEFTYFEDLVALQKELDELLARLQCVFQLPLLLLVAGEFINVLANLYAQLYYYVITKAWWFAFVFYYAKISAELYLLIHTVYFCCVLRTVITHLFLDREVDFEDAPETSRYELTHSDVMWPQPTQFFILGLFELNNEFWLFLVTYSVNFIVIILQFGFFT

>CcapGr67a

MIKKWIGKGIQLIFFAIFYYVRAFGVCTFTYRKGQPPTANVQAFKNRGYSKRRPYYAIECTWFTWICTIFRILSACGYFYGSLMFWYTIDPFFNIITFLQSVVTVSGAIVMGVGLLRSRTIFVNIVNYFIKLFQRTQRLAPHKSIMCVQHFAFLLIIILCMTRDIVDLLVLFDYTDYRERAVAFAKLYLEISLIFTIHIGCIGYMSVGALYEFVNKYMREEFQPRARCLDYVLKRNHNIRSQRYQRKLIRLTRELNECACIYNNIYRAATSFHHHIRFHIFFSLIFTFSFLTTVIYSVLYLYTFYHGFIWWALFVCLQVFTELLIMIAAAKSAVQGGFSVNKLSLDSIYMAGNAEWNQSVEIFINRTNLYEFKPNLLGLFDISSNILLMFLSASVTYLTYILQNTMLSQH

>CcapGr67b

MANKWICFAIEVLLNLFFHFTRFCGICSLNRHKERRSDNRLNIAPYSKGPIYTVTQCTWHKWLFGIFRILSAIGYLYGNLAMWYTADQFFNLINILQSTLSVSGSIALSVVLMRSDVRFANNGNYFLHLYNRIQRLAPDKSMMGLHQLIFLLIIFVCMGNDLLGFCVLRDYKNVREMAVVAAKLFLDMCLVISMHVASIGFICIGALYGFMNAYMREIFEPRARCLDYVLKQKHNYRLQMNQRKLIRLTRELNECTSIYNDIYSAANTFHHSIRYQIFFALLFEFSLFTTIMYSMLYVRTVLHDFHWDAVVYSGQIFIGVLILILSVYSAVQSGLTTRNLSLDSVYMDGNAEWNRSVEIFINRTNLYEFKPNLLGLFDISSNILLMFLSASVTYLTYILQNTMLSQH

>CcapGr68

MSSYSRSERFAYLTLVALYYYGRLLCVFDWKLEKSKMLVRPTKCTNRILLLIWRIFITMIFISVMPSMMAPFQRLSIDSFLAFFANLQVITVTLFSVISFLIQELSERKIFKIIEKIVKIYKRIYGKSHVRQILGRTFVFSVITKLLLSFLGLIYEVPLILEGDQSVKSLCGIYLWLGTIYTLDACFLGFLIIGQMYTAMAAHLGEMVQAMSDYESEEPLSTLSKHERMKRLCVHSESIDDSNNIYFVLYGLTKQFHHIFRWQLIYSIYYNFVIILMVMHSFIWQYIYAGYVDFLALFSSLFKLWNLAMLILAAHGVVEKSQLPDLLNLDLVCSDIDARWDESVEAFICQRKVENLEIKVLGFFHLNNEFILVIISAIMSYLFILIQFALTSKYKYMHTYIYICMYIT

>CcapGr69

MTDLKWVKICVGSVIVYLMLFGFLAHSVSLRKGRFKDSHLMRLYSFAIATTFTMSYGRNMLIDLLKNRFNLENVIQLYSYMNIVAVIVNYLTALILSRTFVRFFNRVALYETIKFFDVNAKTLIVSVRLVLLKGLLIPVIYEVTLILQQIRNAPDKHLLWTFHTLLPMILSQMFPNLFFSALVICKTLMVSLNERLMEIVNEVNCLQNILQMSLQKPYYRMQRFCELADRLDLLAQKYDIICKQTTVYLHLLSAPLICSLLCNLCGITAGCFTQYLAIAETLVNNEPYDAFNALTNAVFLVISIVEVLLQGHICDDNRLKVQETGLILQRMNLTHADLRFKESVEAFSLLVLVTEYKIQPLGLLEINISLVQDVLSAVTSFLLIFVQSDLTLRFSLK

>CcapGr70

MSMFSWFWRTLHTYVTGNFHCSEFAYIAYPFGRIYKLLGFTPIQLRRGEFLRNGCGSRMQWDTKAWMWTAVNVFVYTMGFFSGVYQLQSADYKRGDGISRLTGWAQVYTLFTLGLVAWITSWTHMEEQCRLCALIMKIDQRLRDIVEIDFDYKTLRKQLFWQLGLELLIAFPLSMVNCIIIQPDEKSFMPMSACFWFICFAPISLLIFKQFQFCHLMRLLKTKFELINNKLCKFNRDTHFACGRRRIMTVRKMNSVSEVPLTATVATSAAVEAELTEEPNVDILQKLLTIYSNVSDGVDLVLSIFGCHLLCLTAVSFGVITVQSYNLFSLVSHTLKIHVYEIVFIVAWILVQIMAIAVNVMICSRTSRAMENTVSVLHKMRVSSNEASNASLFYQILQIFSMEVMQRKRNFNAAGFFDMDYKLITSILASSTTYLVIIIQFHLTNIPDCHFPKE

>CcapGr71

MKVYTENIDSIDLCFKWIYNILYYGGCLSFQLRRKTLQLTKGNIVYTHFIRSALILSFVGSIALKNSSGYGSKAMLDQLSPVLKLILGFETFTSTVTYIAVTSAMHANRYKHLKLLFQFKELDEQMQATYPKIKWNYHKTMRKFTPITLCGMFYYYAVSLIYVFNLSNCNCDYATTLMFALSYASITITPGCTFFLHLGMMDLQRIRYRLIQRLLRQEYCGLRKDASKQCKFKLRITRLIDYYKRYIELILQINDVFGVVCGISLFHDFTVLTNMTFLMCQKATESGTRSEEYVFIFLFMLPRIYKVTIYAVYGYVTQMEQRNCAHEIRMSSKYFRSSLVMRNKLSAFLHWQMQKKYTFLVGRMTRCNLILLYTTVNSIASSVIILIQLQFQQNSITERMKNGRMLQDVELI

>CcapGr72

MSWRRLFYQPESTYEANGLFTAIQFITCCNGFIYRRGHFIVNFWTKLYTMLMPFITILSLGLGIQQLINDPVESARFEETDQLWLAVCVLEMIMATVAYVLIVYSMVKHTRDHVELYDRISALDRLLLRDFGVNLKYHKLMRKNLIEYVILSIVYCVALCWTLFMVKPNKMAHACGMAYAYLAMTAGPHCSSYLQVNFAAMLRIRFRLLQKLLDEKFLLAKFPQSGLREVRLLKLVDVVRAFHELIDAINDVYRVTLTVGLAHDFTLVTIILYMLFGHSMGEAVDGVFFAFGGMWLAVPLHKFLTAPVYCNRAIEEGKRCLRLIEKIDICFPNFKSAKRIVTATMHWRLENKIQFTCGFNMIYNKTIITTITAVVFNYLLILIQFRMTQLMGKQIEEQKNILQDWIGDL

**The Ionotropic Receptor (IR) family**

In addition to the OR and GR families in the insect chemoreceptor superfamily of seven transmembrane proteins  [18], there is a second completely different family of olfactory and gustatory receptors in insects, the ionotropic receptors  [4, 45], which evolved from the ionotropic glutamate receptors involved in synaptic transmission  [46]. These proteins are somewhat larger than the ORs and GRs, and have three transmembrane domains, two in the middle and one at their C-terminus. They function as obligate heterodimers, usually of two and sometimes three different proteins. While some of these IRs are fairly well conserved, and have been implicated in olfaction, others are highly divergent and most of these are implicated in gustation, with most of the IRs in *Drosophila* function in gustation  [5, 47]. Like the ORs, and probably many GRs, many IRs probably function in complexes with some of the most conserved proteins, specifically IR8a and/or IR25a  [16, 48].

Naming and numbering of the *Ceratitis* IRs is complicated, but followed a method used for the termite *Zootermopsis nevadensis*  [49] as well as *M. domestica*  [10]. Following the example of the Benton group  [46], the conserved orthologs of most IRs in *Drosophila* are given those names, even though they have no cytological meaning beyond *D. melanogaster* (like the OBPs, ORs, and GRs, they were named in *Drosophila* for their cytological location). *Ceratitis* has one instance of multiple paralogs related to a single Drosophila protein, these are indicated with a numeral (CcapIr68b1/2). There are some *Ceratitis* IRs with no clear simple orthologous relationship with *Drosophila* IRs, either because the latter was lost, or they are simply too divergent, and these were numbered from CcapIr101 onwards, which avoids confusion with any of the DmelIrs, because the latter only go up to DmelIr100a.

The CcapIr gene set consists of 70 models, which is again intermediate in size between the 65 in *D. melanogaster*  [45, 46] and the 110 in *M. domestica*  [10]. Nine of these genes are pseudogenes, however, so the functional repertoire is comparable with *Drosophila* at ~60 (see Additional file 2: Table S13).

The IR family contains several conserved orthologous genes shared across insects. The co-receptor Ir8a and 25a genes are unusually highly conserved and since they cluster confidently in larger trees with the ionotropic glutamate receptors, from which they clearly evolved  [46], they were assigned as the outgroup to root the tree (see Additional file 3: Figure S2). Many of the other *Drosophila* IRs have simple single orthologs in *Ceratitis* and *Musca*, presumably serving similar roles in chemoreception, e.g. 10a, 21a, 31a, 40a, 41a, 60a, 64a, 68a, 75d, 76a, 76b, 84a, 85a, 87a, 92a, 93a, and 100a, however 100a is a pseudogene with a single stop codon in the first exon in the assembled genome sequence (raw reads support the assembly, however this might be a polymorphic pseudogene fixed in the inbred strain used for genome sequencing). There is one instance of recent duplication of a conserved gene in the *Ceratitis* lineage, CcapIr68b1/2 (see Additional file 3: Figure S2). *Musca*, in contrast, has expansions of the Ir10a, 76a, and 84a lineages. Like *Musca*, *Ceratitis* has two genes matching the trio of DmelIr75a-c  [50]. All of these genes are also those that show the highest levels of conservation and simple orthologs across the *Drosophila* species, and are implicated in olfaction  [46].

There are two remaining large subfamilies of IRs and both are involved in gustation. The relationships of these genes across these species are rather more complicated, and hence simple orthologous naming was not employed even though some orthology is apparent. The set of DmelIr7a-g (and Ir11a which is a *Drosophila*-specific duplicate of Ir7a) are barely expanded to eight genes in *Ceratitis* (CcapIr101-108), which is far fewer than the 26 related genes in *Musca* (MdomIr101-126), including 3 or 4 lineages that were lost from *Drosophila* or *Ceratitis* (see Additional file 3: Figure S2). Like the *Drosophila* and *Musca* genes, most of these are in an array on the same scaffold (see Additional file 2: Table S13). In *Drosophila* these IRs are expressed in gustatory neurons  [46].

The remaining *D. melanogaster* IRs are called the Ir20a clade of divergent IRs and are involved in gustation  [5, 47]. Few of these reveal simple orthologous relationships and instead show small species-specific expansions in *Ceratitis* and *Drosophila*, and much larger expansions in *Musca*. They are therefore thought, along with the expansion of the IR7 lineage and expansions of some OR and GR lineages in *Musca*, to be involved in detection of chemicals in the septic environment of that species  [10]. Although the ligands for only a few IRs are known  [4, 50], the similarity of the repertoire size and most IR gene lineages of *Ceratitis* and *Drosophila* suggest that they have more in common in terms of ligand detection than *Musca*.

**70 CcapIRs in FASTA format**

>CcapIr8a

MWLPQKFAILAIFSCVVANELKIAFWIDPLQAGIEADITSTVKEIEALDLQTKIQHYVMVVENEAKKREQNLEKFCTLLGTEGVSVVIDFTYHFWRDGLELLRNYQIPVLRVERIMAPYLKMFSQFVLEKSGHECIMIFQNEKETEEAIIQVIEGFSFRTLVLHAFDESDFVQKIRQLRPAPTCYAIFAGGTAMNNIFERISEGKLFERPREWHFIYLDPRDRVFKSKKLVDDATKFTLNPKTLCRALRMKDTYCLKGFTFQGAILLEILRGLIEIKQRNVYWLQSYMMECNATSPNENATTGFDILEQFPLSDFIYFTTDSTFPEDELELLPRLTYSPTININLYSSEHETATELAIWQYGQLRKINQTLSPPRRFFRIGTVEAMPWNYMKRDPNTDELVLDAYGNPIWEGFCIDSIKKLAERLNFGYILVPPTTGEFGSYDKSTGKWDGIVGDLTTGETDFAVTALKMYAEREEVIDYIAPYFEQTGISIVMRKPVRQTSLFKFMTVLRVEVWFSIIAALVGSAFMIWLLEKISPYSYRNNRAAYQYLCREFTLRESFWFALTSFTPQGGGEAPKAVSGRVMVAAYWLFVVLMLATFTANLAAFLTVERMQTPVQSLEQLARQSRINYTVVEGSSTHQYFINMKFAEDTLYRMWKELVLNVTDDFQRYRIWDYPIKEQYGTILLAINGSEPVKDAKEGFRKVNEHENADFAFIHDSSEIKYELTRNCNLTEVGEVFAEQPYAIAIQQGSHFADELSYALLELQKDRFFEDLKAKYWNISLIKACSVNEEQEGISLESLGGVFIATLFGLGLAMVTLVLEIIYYRRKYAIMHRFSEVIKVKPASASSTKQLVPKMNPLKKRIATIWHTGDNSPELKTPPPAFDAIKFRGKKIPPSITLGGHEFKPRRLGARQPSESLDGAEYEKRGLPPNRDDELPPYTE

>CcapIr10a

MGTREITSSDAMIYYLLLLFPINYAQAKVQQQRLQWQFSVIPNGTNNIESINLTKIGEELRKYTLQLPHINVLLRAKYLENDDANPYVRWFIRHSNLPVIIKSYDHKQIIKNQSRTLLPVSKNRDSFVISTDSQQLHNVAQRFVQRAGVFFFILSDAQLPQNSGDLHNVLHTLWIKYRSFKNFLLTFQGIFYFNPFAYEADTGKYGRLVLNDNTKPLDITMFRDMHGYPLRIQIFKSVYARPFLNMSNPSIKDVYGVDGRVAELLEQQMNFTMVLQEPDPNYFGERSKDGKYNGAIGSIIEDKIDLCLTGFFIKDYLVTEYMDFTVAVYDDKLCIYVPKAQRIPQSILPLFSVGYDVWLGFILTALVNCLIWYLIRRLNLYLNIRKCADVSLDRSQWWQFVRIYIDTWVLWVRVNIVRYPPFNSERIFIASLCLVSVIFGAIFESSLATVFIHPLYYNDINTLQELDESNLKIIYKYSSMADDLFFSETSPLYASLNKKLVHLQDLNADVILDIAKHGGKAGVTRANSLMLESLQFILAKQVYVVPECPKDYTISYIIPKDAPWEEAVNNLLLQFSSLGLITKWINDMKTEVDIDVMKSGLDLNQANTFKVLTISDLQLAFYVILMGNAIAGISLVMEICLKCGGQHSKGKLCSF

>CcapIr21a

MARQSDWSQFMTRLGWLLLCLQSTLGALLADGGLAEAGCVSATLIKRYNLNPQLYGHCEYRQRNVTVPLLPSRSRREIKPIFRGHPKPRGEILAHKFHMNSYNYDQTDSLVLLVNKIATEYLHKCPPVIYYDSFVKKSEGMILETLFKTFPISFYHGEINEHYKAINHRLKRRIDSHCKSYILFLSDPQMTRKIIGPQIESRVVLIARSSQWKLRDFLASETSSNIVNLLVVGESLTESSLRERPYVLYTHKLYTDGLGSNTPIVLTSWIRGAFSRPHVNLFPPKFTTGFAGHSFQVSAINQPPFIFRIKSLAAGGVSRTNWDGFEYRLLTMIASKLNFTIDIIEPPPRPGMKSIIDSIQLQVASRAADIGMCGLYITEGRMLDTDMSVGHSLDCASFITLASKALPKYRAIMGPFQWPVWVCIVIIYLGAIFPIVYSDRLTLRHLIGNWGEMENMFWYVFGMFTNSLTFSGRYSWASTQKTSTRLLIGSYWLFTIIITACYTGSIIAFVTLPAFPSTVDSVNDLLGLFFRVGTLDNGGWETWFQNSTHTPTVRLYKKMEFVSNLEEGIGNVTQSFFWNYAFLGSAAQLEFMVQKNFTDDNISRRSALHLSEECFALFQVGFLYPRDSVYKRKIDSMILLAQQSGLTNKILNEVKWTMQRSASGKLLQASSSSALRERIQEERQLTTADTEGMFLLMGIGYLLGAIALVSEIVGGIANKCRQIVRRSRKSISSAWSSRRNSEDGEGLRTAAEQLAHEQRKEDRRKNEKKGFGVREFNLTKKTLKELYGNYYKQEPNYVLKDGKLLLETEAFSTSSADCNSRGSSDELPAQMVPHLHSKKKAVLVAEVDVEREKYLQRQRERDLITAAAEESLAALDECLKMQRDTSSSESERGGADVADEYELFGSFVESEGPLAAKLNDLSLFNVAADEITQLENDPKVD

>CcapIr25a

MQYAYTKSCRNIFIFFIFLKIILLGSFATAQTNQNINVFFINDADNEPASKAVTVVSTYLKKNPSYGISIQVDQVEANKTDAKTLLESICSKYAESIDRKQPPHVVFDATKSGIASETVKSFTQALGLPTISASYGQEGDLRQWRDIDENKQKYLLQVMPPADLIPEVVRSIVRKMNITNAAILYDDTFVMDHKYKSLLQNIQTRHVITGIAKEGKREREEQIEKLRNLDINNFFILGNLMSIRMVLESVKPSYFERNFAWHAITQSEGEVSSQRDNATIMFLKPMSYAQNRDRFGRLKTTFNLNEEPQIMSAFYFDLALRTFLAIKDMLQSGAWPKNMQYIGCDEFQGGNTPERNIDLRSAFSMIQEPTSYGLFELVTQPGKPFNGYSYMKFEMDINVLQIRGGNSVNTKSIGTWTAGLDSPLVVKDEEIMKNLTADTVYRIFTVVQAPFIIKDDKAPKGYKGYCIDLINEIADIVHFDYTIQEVEDGKFGNMDEKGEWNGIVKKLMDKQADIGLGSMHVMAEREIVIDFTVPYYDLVGITIMMQRPQVPSSLFKFLTVLETNVWLCILAAYFFTSFLMWIFDRWSPYSYQNNREKYKDDDEKREFNLKECLWFCMTSLTPQGGGEAPKNLSGRLVAATWWLFGFIIIASYTANLAAFLTVSRLDTPVESLDDLAKQYKILYAPLNGSSAMTYFQRMANIEQRFYEIWKDLSLNDSLTPLERSKLAVWDYPVSDKYTKMWQAMQEAQLPATLEEAVERVRNSTSATGFAFLGDATDIRYLVMTNCDLQIVGEEFSRKPYAIAVQQGSHLKDQFNNAILTLLNKRQLEKLKEKWWKNDETQAKCDKPEDQSDGISIHNIGGVFIVIFVGIGMACITLVFEYWWYKYRKNPRIVDVIEANSGVKDGKVDSVILGQAGKEYGKSGNTVLRPRFHQYPTTFKPRF

>CcapIr31a

MLWNCKIFIFALGAALILSHNARGDYHNALGLILNKFISNYALTVNGIVFACNKNNLLQISRETLKGANFIEIININDNYIPNTVFTRASYRKTLITSDCTCVNTKNLLLQASVGRFLNKTYQWFLWSVENQNAELFIPSGMKYLGPNSQLTFINGTKSTIKIWSVYSNGMHLKSPLKTLLIGSVRRNESAQENIGKYILDAQLTRKRNQFHGLTLRGVTVIDYDNVTSNGEITVLLSERAKHSGISAFTKYYFELTNILKEHINFSIEFRIARGWAGRLDNTTFRLGFLGIMARNEADIGASGIFNRKNRFADFDIIHQGWKFETAFVYKFVPELRTKIERASFFVPFQKSVWIVIIWLITVLSLIYWLMDNITIRLKNKTPFIARGYIYKQRTRYQNKIYEYGRKYDDFIPEEQISLTNIALFPIAATCQQNFSSESKLGAVKITFLVMFLYSLLLYNYYTSSVVSGLLSSTAQGPSSVEEIISSSLEVSFEDIGYYKILFRENKSPIIIRFIATKLLPPRKENDLPIYTNIKNAVHFIKRGSHAFHCELVDAFPEIAKNFDANDICALRIVKGLMDSELMNGILHKNSQYTEIFRYTLSWSRESGLIGRILYNRLQRKPPCQAVYIVFPVNFSNITSICILLVGGILLSLLTSFAELYIYRYYFVRKYSMN

>CcapIr40a

MKVLLLNLILWLPAALVLGGITYNAERNISDVAIALSEIINALKPRQLAILSAPQFHFNTRHAPLPAAAEIPNDSQLESMQMDIDDFIYKLHKLNFKSVIYNKADLFFKFVEDSLLGSIESVNLIFSAPYELSARIQERKLSHRLSLFIFYWGAKHPPKANEVRFEEPMRAVVITRPRKKAFRIYYNQAVPDGVSNLRLVNWYDGDNLGLQKVPLLPNAASVYSNFNGRVFRVPVFHSPPWFWVNYDNDSINSTMLDDYIDSSNSYMELTEVNVTGGRDHCLLNLLAQHMNFQFVYIEAPGRTQGSLRIDDIGGENETFTGGIGLLQNGLADFLLGDVSLSWERRKAVEFSFFTLADSGAFATHAPRRLNEAFAIIRPFKRDVWPYLILTVIFSGPIFYAIIAIPYKWHLPCQGRAGSVRRTKYRKQQSRQQQQQPQERDIERANELVFHVAYIREITGDNEMAKRLLRRQRQQQQLLGETNAQQERLGDGALGMAEMPYNLFDKCIWFTVQLFLKQSCKELYHGYRAKFLMIVYWIAATYVLADVYSAQLTSQFARPPHEAPINTLQRLQTAMLRDGYQLFVEKESSSLEMLENGTEIFRQLYALMKQQNPDVEGYLIDSVEAGILLIADGLENKAVLGGRETLYFNIQQFGSKTFQLSHKLYTRYSAVAVQIGCPFLDSLNDVIIHLFEGGILDKMTNAEYATQSRMLGKEYNNQHPTSAAETNGNNEPPPSDDNRNGNGGSTDVGGKADESAGTATKSQDTQIIQPLNLRMLQGAFIVLLCGYAAATVILVLELCCHRLNWNFMERFQVRLLRRYRCVCRNFRRIAHSVFVRVFR

>CcapIr41a

MLAANLLNSFYWTRMFGVLSCLQTSLLRTTTCIIWPDNENFTINWQQANPPAAAVVNIRRHDLEASFAKDVVDFAAKRVELLNDYVVLNPFVEKLTLSIEKSHCQSFIAFQLDIPIFIDAIINASRYSIWRSANNKFLFVYNEGDLSEEHFQHRFFEDQSSILLVERSIGNPALFDLKTNKFVGSRADNPKQLYPLDRFNAESNTFQYGNDLFPDKLSDLQGREVILAAFDYRPDVVLKYYPGAPSRDRAFATNDTSGHVELDGTEERILSTFCEKHNCSVHIDTSEADDWGIAYRNMTGEAALGMLARGKAEVGMSAMYTWYADYVALDMSMYIGRSGITCVVPAPKRLASWLLPIEPFQPALWAFVFACLCVEIIALLFIDHFRPIIIALSENMRLPFKNSWARNFEYAFSTTMMLFVSQSNKGTMVNFAPLRLMLFASFLNDIVVTSIYGGGLSSILTVPSFGQAADSVERLYAFQLKWGADSEAWVAGIRDDDSEIMKGLLRNFKIYTAEELTVLAETQEMGFTIERLPFGHFAVQDHLTSSVLSKMKIMIEDIYFQYTVAFTARMWPMLDNFNEMVVMWHSSGLDKFWEWRSVADYLDGNIQKQLMASQYSNLDDIGPVKLGMSNFVGMLLLWLLGIVFAFLAFLGELLLDRMKRAKQLAALDVDEMSGGIKFN

>CcapIr60a

MRPLRNMKRIEGSAFAITHFLSTWQQCYALMNEHQETANSVIFLRPPAEMGPEPWISGIDCLEAFTKIVFTNRVRLTRSYNMLITNSYNISSPALEIQDGFIKLINEAITQSTERGRRFYQLRTVFDNEPYVKQKLGDSAVIFTDYYVIILDSVELLRVLMRDFISRMVSWNPGARFLLLYNNPLRRDSEHEIAMELFTLLQKQYYVHQIGLLYSTGRVDYSFLVMDYYNNANCRTIRVSKIGECKNGIPQPTAAEVKRKLRRFERDIEIKNCTFKMCAAVSAPFVERDCRTGLELLIVSFMRSRLNFKIQQTCEEENRGVREENGTWTGLLGKLFDRDCDFVLGGFYPDDEVNENFWASTCYLSDSYTWFIKLADPRPAWIALLHIFKEETWICFIVLLLLTWIFWYFFVNMLPEPKDHKDFSLAGINAMAVSICVTVEERPECFASRIFFLAATFYGMNVVAIYTSKLISVFTDPGYLHQIHDLEEVIAADIAFGGPDENQDWFQNEDDIWIFDKYNDSDLFIPNSENLHAVRHGERVILASRMYVLQDRIADEVFAFPENVFTTPLQIITKAGFPLIPDFNWLIGAMSDHGIFQKLENDFRYNNTYLNRISRMRPDFKESAIVLTTDHLKGSFSILFVGIVGGIILFVCELFYYWILAPRLRRGSKEKKLKETKMKHTKEKRRNKLDHEKKPAKKNKKRHLCWPFRKFAGKKIVALKKKKAKHRARLDNKTHEIVVAESLRFTPIKRRHIDLHGFY

>CcapIr64aJOI

MSHFSHFTYTTHIKASIKALFNSTYHWLLMEDYTFNRRTDNDDNVNKSNNNRNHCNTTHTDQSNESVDDNNSNGDVDDDVESAVDKFSQRKQRQQQENGKVSLPATAFTSTSAALATRTAIAAPATVNNEETMEIIEKYLEKINININTELILAKRHTRRIRDNVDVGCVVKSCRTDSESATNNTLQRTDYYQLYDVWNPGLQYGGQLNISEIGYFALDDGLQIALWYRRSTTITRRMNMKMARIRCLIVVTNKNFTDTLEHYLTTPYDTHLDSMNRFNFALLSNVRDLYNFSFVMSKTASWGYLKNGKFDGMIGALVRKQADIGGSPIFFRIERAKVIDYTTRTWVARPCFIFRHPPSTKKDRIVFLQPFSNTVWILLGLCGIFTICLLWLLTTVERKLAAAGVVRQLGIRNDTRGSVNKTNNKSKQQQAIGVNGEQQVRDGCSAWMTVSDGTMPCDGAYKNAFSSLGRITKEQQRKQKLQKLVKRDKRERRQKHEQNISFRHCMLGCGNACCGQTGTDVAQQRVGLFFESLLFYVGSICQQGLTFSTSFFSGRCIVITSLLFAFAIYQFYSASIVGTLLMEKPKTIRTLRDLIHSSLAVGVEDIPYNRDYFLRTKDPIAIELYAKKVTSVPTDNDPLSDMAGENVSTLPTVQPTFQLTEAEKAKAYRDILHSHETGAHAKTNEASNWYDPEYGVKRIRRGKFAFHVDVATAYKIIADTFNEKEICDLTEIQLFPPQKMVNIVQKGSPLRKIITYGLRRVTETGLMDYQRKVWHSPKPRCVKQIHTDDLRVDLQTFASALLVLIFGYAVSLLALSIEIMQHKLYERHEQEHEEKEEEAPEDVDDDENVEQEYE

>CcapIr68a

MLKNSIGLFLVLLKFMQLCGADDEGLSLLEAPLNDPPHRNLQHLLTTLLGAARIERCFVIITDNTYQALYDSSFFRNQRTPGAYFFIKVLPTEDLLAPNYQTVRVLKKIRAQNCEFVFVTLLNGLQVKRFLRFVEKNRLLNLQQRYVLLQDTRLLVADMWYIWSNMISTLFVKPVKNQRYLLSTIAYPEVLNGLVVTKRLMFWERGKRIKINRLYEDRTSNLKGFALPVVVFEHVPMVRRGADNVSFVGLEVEIIKALGVKLNFKASFYETSDAASAHWGKELPNGSYSGVLGDMANRNARIAIGNLHTYKLYTSVMDLSWPHSFECLTFLTPESSQDDSWRTLIQPFSGTMWASVVLSLFIVGTVFYMISCLHAILLQRRSRRTHHVLNWSEWRSKGLFRPPPRSFDTKLYRDVNFRRYLSQLKSLPRKRTDLFDEYANCLLFTYSMLMYVSLPRLPRTWSLRVLTGWYWLYCILLTVIYRASLTAILANPAARITIDTLEELSQSYVTSTAWVHENKQFFVEAFDEIAQEIGGKMEIVDNIESVAERITKGEYAYFDNEYFLRYLRMKSAQRREEQQFTEVVLHIMRQCVIQMPVALGLEKNSPLQPNVNKYLRRLGEGGLIAKWLKDAVAELPAEERTPQEAVMDLPKIWSSFVALGIGYFLGICAIAAEWLHYERVVRKHPLYDKYNKNLYYNFKRKFSNY

>CcapIr68b1

MLYLKVVIILFNYRINSLAFGYWTAGIQTEATNALLPQKACQIAAAHDSYVYIIYTGVERNNSAFALEINLLKCMATLPTHIMELRSVTKEREHTYNSFSLFVLSARPEESTAYVRRITNIMNEKQKRKHVHKYIFLWRDASLEQLRELLSAVWQRKILNAIVITGERHIYTFEPYAEPGFIIKRITDNRYFYDKLRNLHKQQLRITMFKDFLRAIPMSRAELGYTGVDGLFAGNVAAYLNATANYTQPADNENYGSCLPNGSFTGVLRDLISGTADVSFNARFTLPCAAAHVEMLYPYFKRKLYLVVPAAKMQPEYLIFIKAFTCTLWLLLSLNFFAVTAIFVLLNRWAERLPHAVKMKHGRWYEVFEMFVKTHLGEPVAGFSRISSLRQYLIAWICFSYILTTIYFGKLESSFVHPEYEAELDSLDELHKLDVPIFGVHSLFTTVQSALSVAHWGEIEKRAVSLPLYFSSFLFAVPVSVRRDWRAAFVLRGETAKDFLIKTYDVERRRPRFHVVKEYLRSMPQEYILQKGSPFRYKFQQYQARIFESGLLEHWSIKDLYYTGGTTSHEVEEFYEDLPSDTDFDLTEFTDNEGVGADRKKKRIVLNLDILQGAFWLWCFGIVFSVIGFGVEYTYWWYGKKSVKIIV

>CcapIr68b2

MAKMLCPRTVRILVLVIAKVHFLKAVTCEHFNLPEPKHYEDLSQKVCEIAEQVDAHVNLIYSSHGNAKDDNLHDLGEELLHCMSRLPSYKLQFEEFKLELYRNYSTLSVFTLAGSDSTHLRRIIHMLNTKQPYKELQKYIFVWRNAREQDMQQLFKAIWDKQILNAIVVKGANSVYTFEPFTTTGFLVRQWLAGEPYFYDKLKNLHHFQLRISMFKDFLRAIPKSSSQLGYTGVDGLMASSIVSQLNATVQYVMPEDNENYGACLENGSYTGVLRDLLGGRTHVNFNAQFVLPCVAERIETLYPYFKRILYVVVPAAEMFPEYLIFVYAYKDSVWQIFVVFFFLVVAAFALLKWAIETLRNVEKMKDGRWYDLIELFWKVQLGVPINYFSSTHTLRLFLMAWILFNYVMTSIYFAKVESIFVHASYYPEIDRLDGLHALDVPIFGVQNIFTAVKPALKPQHWRAIERHAVHLPQQFSSFQYAVPIAVRKHWRVAYLLRGETARDLLAKTYDAERRRPRFHVVKEHMLAMPQTYLLPKGSPFRYKFQEFESRVLESGIFEYWTHNAPHKRVNGASPDLEEFRHELPNDLNFEAQEIDVADDKEKKVVLNMRILQGPFYMWAFGMGISFLGFLLEHAYCWWRRPSVFEL

>CcapIr75a

MDLRLFNFIIYYFLKINMKVLVNFNCWDMETQLAFYKLAAENSFYIDSVNINNSRAIQGIEYRLIGKRPTMGIFMDLNCKSSEELLDIASRERLFNDHFFWLIYDEMANVTYFRGLFKQLNLAVDAEISYAVLNNPGSDWNEGTLAVYTLYDVYNNGYYYGGKLNMTLDREVNCDEEECKVNKYLSKLHLRNKYGNRNRLHDATLRLTVVVTKVPITSPPEQIFTFLRTVNNTSEDAIPRFGFQALSILVHILGCKTNHTFVNRWTINETHGGLIGALAVQSADLISTPFIPTPPRMEFFTIIAETSSFRSICLFRTPRNSGIQGDVFLKPFNTTVWSLFALLLLLTAVVLWSIFRLERYRMKKRYIDYMQYMPSILATFLISFGSACAQGSDMVPGSIGGRMVFFTLYLLTFLMYNYYTSIVVSSLLGSPVKSDIKTMGQLADSSLEVGLEPLPFTLTYLNNSLLPEVRRFKRKIDSTPNPQAIWMPLEKGILRVRDQPGFVFGSEASPVFLLVKRYYKPYEICDLNEILFRPEKSLYTGVHKNSSFKEVTKQRVIRILETGVQLKLHRYWVQSTLECFDSNFIVEVGMEYMAPLFMLLACTYLLVLLILLCEILHKKYWTDRKMRLESVLFGRNWHNE

>CcapIr75b

MHQMLFNFIINHFLKANIYTLLVFNCWDWQVQRQFLHKLNERSLYTRFVHIETADFSGDFESHYLRHGRPGLGAYLDMNCNRSAELMGTLSHWRLYNEHYNWLLYDRTADLDNFRRIFTDANLAVDAELTYAILKPTLPRNLTEASNISYIAYDVYNNGRFLGGKLNMTLDREFECNLESCYIKRNISALHTRTKYGNRNLLHDITMRVTVVVTKRPLSLPVPVLLDFLTSENNSDVDPISRFGYKIFLIYKDFFGCKMRYTFRAHWGLNDTHGGGVGDVALGYSDFLSTPFLISAERLRYFSPLVESGGFRLLCLFRTPRSTGMKGSAFIEPFNGSVWLVFGILLVVSAIFLWRTFALELRNFHTNLSYKPSLLSTALLAFGSACYQGSSIVPSSAGGRMAYFSLYSATFMMYNYYTSILLSTLLGTPPKSDIKTLGQLADSALNVGLEPLPYTYVFLNGSQLPDVRRFVYRKIESKKDPEKLWISVEEGILKVRDEPGFAFVLETSTSYPFLERNFLPHQICDLNEVLMRPDKSLFTQLHKNSSYKELTRLRGIRMLETGVWRKHRKHWFREHLNCVPSNYLFAVGMEYTAPLFLMLAFSYILCLVILLVEKLIKRMQERF

>CcapIr75d

MKLINFNTLCMLLIYCTILGATTGVALPNQLVSVARSSNSTAKAPTAADAAPQPHDADIFMEYFRWHGVHNIMLIVCPQDVATSEAQHKLQSFVRKFMANGFSIRIFNGQDYDDAKVNKPSSESEELNISATVSSTVEATSSTPPPPPSFGPPRTFRSDNSTRRPLRLQLPPLTYKSGFLMLHFASDCALNVLRWSAASENNYFTTNRFWLLLTADPTHISLLEDVEIFVPPDCEVRVIVRQPKLQFFTLVDVYKIAADKPLRRTLLGGASGELWSVQDMLQALREFGSAITYRQNLEGITFKTGLVIAFPDLFTNIEDISLRHIDTISKVNNRLTIELANRLNLHFNTHQVDNYGWHQPNGSFDGLMGRFQRYELDFGQMAIFMRLDRIALVDFVAETFRIRAGIMFRQPPLSAVANIFAMPFASDVWIAILLLMIFTIGIFMVELVYSPHSHEIDILDCVVFVWGAMCQQGFYANLLNRSARVIIFTTFVSTLFLYTSFSANIVALLQSPSEAIQTLSDLAQSPLEIGVQDTVYNKIYFNESTDPVTNHLYHKKIAPKGDNIFMRPTVGMEKMRTCLFAYQVELQAGYQIISNTFSEPEKCGLKELEPFQLPMIAVPTRKNFPYKELFRRQLRWQREVGLMNREELKWFPQKPKCEGGVGGFVSIGITECRYALGIFGFGILLSAFCFVLELTVKYARNIAKKIQRNKQQRKADSVAVEYFGNLKT

>CcapIr76a

MLLLSPAVNLWTRLLNFIIQTYFIDSHATCILWHHDFPFELQTPANSEFIQYVNIWPDNLSQSLQQDIYDFAGFVEEQRGYGMEPDALVQKLTIAIRQSHCESFIAFQQDIEIFARSFYNASRMSVWRSLRNKFLFVYRKDLQQDTGTKAQFDDVIFRVEAECGNCSTFTLKTNKFIGPLAEHPEQLYVVDHYDGLSGKFELGVDLYMDKASNMQGREVTTGVFDYRPFTVVDYDREPQIKDMNSENPRGSAHIDGSEVRMLMALCKVVNCTIQTDTSEDDWGTSYPNLTADGIFGMITEGKSQYAVGALYFWPDDYRFLDMSLFIGRSGVTCLVPSPQRVTSWLLPFQPFQFTLWLGVFACLGVETLALFFTRHLAPSDTEPQYGLLESFQFGYITTLKLFVSQGSDYVVNSNTVRMVLFACYMMDTIVTSVYGGGLSAILTLPSLDEAADSVERLYRHGITWSATSPDWVISLSGAGDDPMYGPLLKKFRVNTYEQLTEMAKTENMGFVLERLAFGHFGNVDFLTDESFKRLKLMVDDIYFQYCFAFVPRLWALLPKLNEVIMAVHSTGLDIFWEWEVAASYMNGQQQEEIQASMYMDFDVGPVKLDMGNFIGLVLPLVVGVILSIFAFIGELIYFKYMAQKKIHDIQIVE

>CcapIr76b

MTGFELILSAALCLSCAQQNDTSLPAGLIELDANGEVVTISPELAASEPSLDDAPLETVKTLLAKKEKIDKLKEWIKGRKLVIATLEDYPLSYTVMENDTRVGKGVAFELIDFLQDQLQFTYEVVVPEDNIIGSREDYEKSLIKMLNNSEADLAAAFIPALSEQHSFVFYSTTTLDEGEWIMVMQRPRESATGSGLMAPFDFWVWILIFISLLAVGPIIYMLIILRNRLTGDDEQKPYSLGHCAWFVYGALMKQGSTLSPIADSTRLLFATWWIFITILTSFYTANLTAFLTLSKFTLPYNTVSDILYKNKHFVSARGGGVEYAIRNTNESLSMLTNMIRNNHAVFSKSSNDTFNLQNFVEKDGYVFVRDRPAINHVLYADYRYRKTISMNDEKLHCPFAMAKEPFLKKNRSFAYPMGSNLSELFDPKLLNLVESGIIKYLSTKDLPNAEICPQNLAGTERQLRNTDLMMTYYIMFAGFATAMVVFFTELIFRYLNSRHEANKWARHGIGRTTNGLSVRAPRWLRQLETDSDKQRLTSSSNESTITPPPPYQSIFSSTNRHQENHHKDATHLSKDRSLNRWRRAGQFGNMIGGASGSQFGALAGGGGAGVLLGNGHFSGGNGGAGGVRRLINGRDYMVFRNPNGQSQLVPVRAPSAALFQYTYTE

>CcapIr84a

MSAPFGATAAKRLAFVTVSLRVRLCIAATLILFSFTCNVSTEAAANPYEFHAFTAILKQQNLQHAVITYNSEMLQSLQREYLHKDSALRTLANMAMLHFYDVHQTKRSKNYIQFQKLFYHESPRVGIYLTQLEDVLLQEHVLGTNAISVDIIDAEGYRLHAGVRFNNSQVWFMMSKQRSVEAALMNARRVLTPLPLNISADITLGVRVDDNNTIQLFDIYKIQRDWLEIEAKGNWSLSDGLQIEQKYLQSYVSRRRNFKGLTLSAGIVVRERPEDMDELQYLSSMDHKKLDPMQRKTYQLMKLMEPMFALSFEAISRPTWGAQLSNGSWDGVMALILSGRAEFSLCPMRFVLNRIHLMDYSIAVHTEFVFFIFRHPRRNSIRNIFFEPFADEVWYTTVAVIVLAISLLFLLLRHEHQFLLNKDPHFQYRLDYAILSILEAFFEQGPETNAFTATSTRMLVFTVCVFSLLLQQFYGAFIVGSLLAVAPRSITTLDALYNSSLEIGMENIPYNTESFENTMVPLGMAIYKERICKNREKHILSIEEGAERIKKGGFAFHVSANRMYILLKSLLSEKEFCELQDVPFIAPYRIGMGITKGSPFREYVTTSIIKFRTTSILQHNDNQWQLPRMDCSLSQNREVEVDLQHFLPALLLLCSAMIFSFFILFLELVYYYFESNTKLARLCPRIFPEQRLEFIN

>CcapIr85a

MSSLSLLYILIGLAAICRLATQSLVDILQPEDSTINTTIIENASTILCSFHTKVVYVYFENHTTSGYSGEMLRYLSDCGTSYITLRNRVKTAPLELIKNDGILMYLVMIFKDAQQRVDLAILRKNSTAKHLSHVIMLIKNSDTVTDAWLRRTFMIFWRIWFLNIVIMFQRDHQLHVYRYNPFADEFLIRVQLDNQTLPGMAELFPTKLPDMGGKPLRVCMYHDVVRAIFEDDKNDQQVTGSDGLLSSFVAERLNATRVIHRVSRFGNFTLRADICFKEITQGIDDVAFNIRFLSAPSFSQHAEYTFVHGLDSLYALVPKAKMATTFWNLFRSFRVPVWFSILLSIPLAYIFCNLIHQHLNKTGFSLLQLFACTLTMPLTRIPTGTPLRIFIFFWLFYGMLICNAFKGNLTSSLVFRTYLNDVNTLKELAESPYDLLTFARHTKHLDRFLDVSDPYEAIIKRKIVVVPDEELLTGIKENNLSYAYVQKYHYATYYANARIHSLRGRPLYHVMSTVLVPFHAVYIVPYGSPYLGFINRLIRSSHEYGYNAHWESIMNAAFIDSGKKTIRDVMNDDDPIVLKLFHFQAAFGIWFVGLTVAFVTLIWETFQRHDAEKVLTHSEVKKN

>CcapIr87a

MSVYTIVCLLPLLINLPCTVHGFGMRLMEVTETERSHVVCAIALLQKYFQYGEPLSGSVLSMSFTSASLCIQQDLLSAMHAQTDIPWTVVVRNPHHTKISLSTTMILHEKPQCYFVIVENLEDGDMEDVFEDWKSNINWNPLAQFVVFLASVEETDEEMTELMIEILLNFMNKKIYNVNVIGRNEENGFYYGKTVFPYHPDNNCGNRVIAVETLDICDYQDDENPKDPEGDENEAHDAADDAAEYEYLDEENGGGDEHDSVESEEYGKKDAKGVTAEEEDYVENNSKEEGVEDDSSGGKNRQSDKDEKEGNVDGISSNEENSIQNGEDVNKNEEGNASEEGNFTLDGNETSGTQHSNENWKECEQQSQRGDTNIFHKYINSKQRDGSGVDDLSGCPVVAAYRPWEPYIYKEPKEETANSNPTAEESDEDATTEVTQTEPEANDTLSVGSAEETDNDNNNYEDVDADTETEAANSYNDVDTADVDVIGVSVELTSKLSGVEYQLAQAIGERLHITIDFQVENTNLYHLFQQLIDGDIEMIIGGIDEDPSISRFVSSSIPYHQDDLTWCVARAKRKYNLFNFLQSFHISTWIFMLSFILVCSLNIFIAQKLLNIRLTHFGGYLTTFVRILGIILSQSTQLLHLPTSLHITFGSTFFLALMFINVYQSFLVSTLTTPRSLYQISHLEEIYRNRMTVTGSVENVRHLNKDGEIFKYIREKFQMCYNIVDCLNDAATDNEGLAVAVSRQHFLYNPRIKRDQLYCFDRNENLYVYLVTMLLPKKFHLLHKINPVIQHIIESGHMQKWARELDLKRRIREEIERARKVHVKSLTVEQVAGSFALHAMLSTLALLIFLLECCTHWLVVKRRTRLRLFRMLHRKFKSK

>CcapIr92a

MQLHAHTHVTTLLQLLIQRYFAQFSSVLIVHDGRVGAGSDLQREYLEAVQLAFRNLSQQGCVIGLQWIDGFITILPDTVRFLHARYFATRYAELRLKDKFYLFLCELENPTDLLSTEILQFYPHHLMVTPEIPTTQQTDNPQATTNQQRRKRSTIKPITNQATRLTTPTALTSSSETTHRDINIQLWTQKFVGASGNLDALLLDAFLPNETFARHAELFPNKLNNLRGRPIQVGAVTYIPYVVTNYVVFLENGAEDSKKNISPIAPGMGDVDALNATDYSRTVSYLGSEAEVMKSFCELHNCHLRVEQYGVDNWGYIYENETWGGVLGGIYTQNVEVAIGCIYNWYNNITETSNTIARSAVTLLGPAPALFPAWRANIMPFSSELWAFLIMTILLCASVMYLIRFTSFTLKNLRWNLKRNFKHSTAFGQAVLDMFAVFIQQPSGPTSLRSFAARFFLAMILCATITLENTYSGQLKSILTVPLFTEAVDTMEKWSKTDWAWSAPAIVWRQTIEGSNIEKEQIMAKKFVVHDYDFLYNASFRMDYGVGIERLMSGSFTFGSFISAPALETKIVPKDDLYFDWTRAVSIRGWPLMPLFDKHIRACIESGLFVHWERQIVAKYLNRKTQEIMLGLASGHINKLPPQKLTLENISGASFALLFGCLFASIVFMLELTVHHFEKFRVSFKSSLSKDKTNEMAQSSSLSPY

>CcapIr93a

MRFYATIWLFLLPFFLSLLPRNAEANDFSSFLTANASLAVVVDQEYMQRRGENILANFQKILSDTIRENLKNGGIEVKYFSWSQIRLKKDFLAAMTVTDCKSTWQFYESTQQTSILLIAITDANCPRLPLNRAIMIPIIDEGQELSQIILDIKVQQILRWKTAAVLLDQTILSDNPTLVESVVHESVKNHITPFSLLLYRIDDSLRSQKKRTVIRQMLMNFQDNVQTPRQFIVLSKFYEDIVEIAASLKLFHVYNQWLFFVLNEELRNYDAVSVTQNLEEGANIAFILNATEPTGTSSINCTIAELSMAFVTSISRMIVEEQSIYGEISDEEWEAIRYTKQEKQDEILGYMKDYIRMNSKCASCSHWKIETALTWGKSEEHRRYQTNLELRDTRNRNFEFIDVGYWTPTLGFITHEVMFPHIVHSFRNITMDILTVHSPPWQILERDSRGDIVRHSGITMEILKEMSRMLNFSYILHEVKTNPAELAAEEMQQTSNLTDDLLGSLTFNIPYQVIETMQSSRYFMAALAATIDEPDKKSFNYTVPISVQTYTFIARQPDEVSRIYLFTAPFTTEIWGCLVAIIIITAPVLYFINRLVPMEHLRITGLSTLNSCFWYIYGALLQQGGMYLPKADSGRLVIGVWWIVVIVLVTTYSGNLVAFLTFPQFQPGIDYFFQIFSSNDVRQFGLRNGSYFEKYSTQITTRDDFRDFVQRAIIYNNVQGEDIGAVQDGKRVNVDWRVNLQLIVQRQFEKNKECKFALGRDSFVDEQIGLMVPKKSPYLQLINDQIMHMFRMGFIERWHQINLPSMDKCNGHGGMRQIMNHKVNLDDMQGCFMVLLFGFMIALFVLFVEYWYRWYFVEKKKATFTS

>CcapIr100aPSE

MQELAKRFLKIFLFVSFTSEFLKAQLIETVPLWKLISSTIQTDTRNSKLNIDTLGELLRGIDYLNLHLFKESNLENDIIQQLLQQSFCPLQLSVGRIMPQPTPNQRHWALVTSSADLAMNLNGYASRKGVLIYILSDQTDKZQLLSLARRLWIERGALRIFYIIPNGSSGGFDCFFLNPFVKTGGQRGEMSRLDGQNYQRIFQNLHGYPLRTYIFHSVYSNIEVFTNETTRKIIGATGADGKVADLLANKMNFTMDLQWPDDAFFGARSSNGSYNGAIGRIVRFETDLIITGFFIKDYLTRDIAFSSPVYMDELCCYVKKASRIPQSILPLFAVNVDIWITFIFVGFVCPFIWMLLRYLNLSTLANRAAFSQPEKLKIQELRYITQSHVQQYKRIVIDAWVMWVRVNLVYYPPFTSERVFIASLSLVSVIFGALFESSLATGYIRPLHYKDINSMKELDEANIRIYIKHAAMRDDLFYGHSSKIYQNLEKKLLLIAELEERLIRTMSRGGRFASVTRASSLELDDIHYFITNKIHKIPECPKNYHIAFVFPSQSPLEKNINILLLKFVQAGFIDHWIGDMKYKAKIRTRNFSGYLEESGENWKILTLNDLQLAFYTIIFGSIMAFVVLVIEWIMQCRRKNFSKKNKSNAYI

>CcapIr101

MFKNHLIIILMCVILAESKAMMRPGADFRPNNQKRLHILVDEENTDHVPYAELALNMTERYISPKTNTLIIMENCEHCKTGLQNKHTRLLRHFLKKLTPTMALQLFFGLPEDRLWDYNLFIVDSEHAFNNLSIHIPPTSYEHQFHFFIILTLKAELEIISLHMIKIFDNCLRLDVFNVVIMIELERNTFGLYTYYPFDEECHRHHMRIVRINTYSNGNLQYDHLFPPPVRNFHGCTLNLCLRVAHPVVLDFRGNRNDPKDLQGFDRLFGIEADIFRNLAKAMNFRVDISIPEEISTIGGHNDSTGCFAELASGRSDVAIGSLTISYGLRDVFSYSISYHQSPFVFIVRRGLHFGPMKQLAQAFCGSVWLAIGVCCVGGMIFIRLVIRHANPSTCEKLIGNPRRSDCTSLIITMMGNPLQVLPRRNSARLLLMAWLLATLVLRSAYQSKLFDTLRTSQRMPVPNSIAGLVENKYILLADRYVNFYPENMTLIVLNVAERYRFVQNSESIRFATFSMLDTLTWHNQLNWNTSRLTYIPEPIYLMQVSMFFKKHSILRSIFNYRIQQLMGAGITSHIAQKHVVKKFQGMNERLQRLPAISSNMLSGLYLLYAFLMAVACLLFALELLAHRNLRVKRLIDWMHLVERA

>CcapIr102

MLVNNLVAIFVSLLLTETEAMPKPGPAPRLNYEERLSIFVDEENTDHVPYAELALNMTERYISPKTNTLIIMENCEHCKTGLQNKHTRLLRHFLKKFTPTMALQLFFGLPEDKLWDYNLFIVDSEHAFNNLSIHIPVTSYEHQFHFFVVLTLRARFEVLLLHMRRIFEYCMRMNVNNVVIMTELQHNTFGFYAFYPFDEGVCRENMHIIRINIYSNATLQSDHLFAPQERNLHGCSLNVCLRLGYPYLGFHGNQSDSQQLQDVQRLSGIEADLLKNLVLALNFSVNISIPEEKSLIGRHNNSTGCFAELASGRSDVAIGCMSISDGSRDVFSYSTTYHQSHFVFIVRRGLHFSPIKQLAQAFCGSVWLAIAVCCIGGLIFIRLIISHTNPSTCEKLVGNPKRSDCTNLIITMMGNPLQVLPRRNSARMLLMTWLLTTLVLRNAYQAKLFDTLRTSKRLCVPHTIAGLEDKSYILLADNYVEFYPQNMTQIVSNTTRRYRIVQNSESTRYTTFSMLDTLGWHNRQNWNTSRLTYVPEPIYLIQLSVFFKKHSILRSLFDHRIKQLMGAGITSHIYRKHVAKHFQMMNESSQRMPVISSNMLKGLYLMYAFLMATACTLFGLELLARNCLWVKRFMDWMHLIKRE

>CcapIr103

MRYSFVNDMWWMKVFILAYLMPYGCGHGAEGNSILAPTLQSLNMTSAIVHYYQQFFNKHTKSTTVAVRANDRKSWTYFNDLLTSVLIRAHDLKVGIDAESGKRQPISFGFYNILLIDSFGALADLDPGFHAQRNDFSEYYLIAMPPTTNSSLLNAELKRIFEYCWRHYIVNVDVLVDLSDNGIGLYTYYPFSQHKCKSTEPARLSQGKSITELSRSEIFPDKVRSFYGCTLTGVLWQVPPYVILPDQSQNVTRFGGIEGYLLNLLVDKLNFSLDYNEPPHNEQRGLVMANGTLTGAIKILAEKSGDLSTGCFRRTLERSTVLTSSISFYQNKQVLIVLTRHEPWSTYEILTYPFNIYVWSSLLIFYLLPIFLACLLQRLSVRALKFIYGVVDTRDMIFSWTGVLVAHITHKTTLPVANFARYIFVMWLLLTLILRSSYQALLYEFFNTQKVIAAPSSLDDLIKNGYKLIANRATADAVSQIKVVRDGQIELMALDVSDTGVFDVLEENPHEYYVASAPSDFLKYYMITERKPGRFHVLRDELFVHHISMFFSKHSFLQQPVNSILMSLRSGGIIEFWSELHIGSIPRVKDEGHIQKRPLTMSQLKGIFEVQYCLYGLAFLVFLVECVRGRNILLKKRIGGSKAFLELVEIET

>CcapIr104

MGALKVLYAALYLSLIVCIRAIFMKQRQLQDGPGDEEISGNLSAAAVKIVRSSFHKIHTYNLYAYVEQPERRYLFGDIMENILRTAGKNQTVRVGMGEPVPGDDLRHFNVLLVDSAASLNATYAEFAKYHLYIYGYFVIVLHTFDSAHYYDVLFEIFELNWLLGIIDANVLVYALSNLSLLYNIHPFNQFHCKGLAPTIGNRFSNLHWRHTNFYPDKLADLHGCALVCATWEDMPYRGLIKGKPKEFGTLAGIEGKLLEYLAEKLNFTMTFQWLNDYESSHTLDKKGVVFRELIENGTDFVVGAFPYKSPTKEDIFTPTFPYFLSSFNFIVKSNPEPYSPFEKLFLPFNNDIWIFLLCIYLGVFALRLCVFVCSPAASRFIFGPSNYMPGVNMFGTCLGVGLPSHQLPQRNFARYILMLWLLLTMLLRSSYQAFLYNLIKSSIGRPPPNTIAELLRQEYQLLMTAEVLDTIHDLPAISSSAQILNISRFDSFEVVRETKHRIALLTPYEIVAFYKRYNASFTKGVHVVEERVFTQQLSFYMASNSMLLSRFNHIILKYINVGLWERWSRVLLDMNWRASEELEEAVTKVTLEQLYGAWYVWFIGVALSILIFLTEKLFNSQ

>CcapIr105

MSGMDVILSEIGRRQFEETGNYTLVVAIAWAVLHAFGKFSSAPLVISQYASKRQNCVYQAELIEALMVYLQQRHYRVYIMEQETLFGDASVSEWGDFSERAIWFIDSWNAFEALAADLEDPKSTYRRSGWFILIYTGNETSRLETVKRIFNRFFELFVINVNIFLLLDSTPYVYTYFPFTHNKCHSAKPELLISFRNNQAKFQRKDNHQFFPSKVHNLYGCELSVITWHDPPFIIFDKDEQSGRVKAVEGIEGLLISVLAQAMNFAIKVVDPQPRDRGAIFENGTLTGVTRMIAEGEGNITIIYTMYEKKRAQVMDASFSYMSFPLMVAIPHGRPLSPLQRLLRPFKYIIWSLIGSNIALAVLIIYSLKFLGSKQVSSFVFGKSNRIPFSNLWASLYGNVIHNHLPYRNFSRYLLALWLLCTLVLRSAYTGQLFIILQDGRALTPLKSFKEIVANNYTISTAPVLTELLSSTLGEASIAHFDGGKNSMPNVLKRIAHGSKEALTIIEPAVLYYNYRQSTDDDRVALLPQKLIMTPLTMYMRKHSYLLWPINSILLYFIDVGIVDKFERRYRLVNEVNESQEPVKLSLFLLLGIFSLYIALITLCILVFLLELWSTRSPLSKVVMDFLNY

>CcapIr106

MHGGFVLIFILKYILKSCGSDSIEAPETVCNSTVRYNMRYALSTIIDRYFVPSSSELMISVHAKHKRSWRLMQDLITCTGPYIYNIKLQLETMGAPRLPTPTRLYNLLLIDGPQALVELDPAELTKNFDFAEQYLVLLIDPDTSLVPRLLITSILSYFYRYSLINVSVLYEIKRDYVEVYTYFPFMGNSPCKGNNVHVINAYNGSWLKPLSTHIFPEKLRDLQNCSLRVAVWDSPPYLSYYPNRSGYAQLGSFEGEMLVEFAKKLNFTLDLVEPPNNEQRGRRLENGTLTGAMQMLQDHVVDLSLGCFRYTVERCELMTGALPYYQTWQIFGVKLAGQTYSSLEIFSFPFDAETWYYLLFSFQLILLLAYVIKMRCRHSELAHIMIGYPRPRSPLTNAYSLFLGIPIQRTPHTNFARFVLVLWIIYGYVMRNAYQSFLYKLLQTDLYRIPPQNIFQLINDGYSLIMTQATYDTVRSAPLIQSGRIPIIINYDPYEWKSYEMIDEMGGKLAAVSPKDYLTYYVMSKRKRGAFFVLPDRFFAQHITIYFSKHSFLVERFNVMLMQLRSQGLVDYWAEKNLDLSYFDILDAEDNDALDMDDLWGVFAMYLILIGLAGLVFVAEVLWHRLVSAVRLLCSRW

>CcapIr107

MNITAMSLSYFNKSGEQNDQADLVAVKATMQVIQNHLLNIGTFFTLTLCCRNQHTCKFFNNLLEKLFHMWGMSMVRFVVLKENSTERVGGDRRSNLIMTDSFKAFREIHIASYTSRNNYNEFYYIFLQIRDAYLQENLQQIFEYCWAHSMINCSVQVQNSHGDITVYSYFPFTNHYCAHVEPVIINRFNGTDFERPLLFPLKLKNFHGCALRAALQHIPPFVYLDTDAQGNSRIVGGIEGHLLKQLSKLLNFTIEIKTPQEKRYHVDAVGLLEKDEVDITLGCFSQTSALMAVASYVTSYYQTSQVLCVSRQAYALNSLEAFTTPFDGTIWLLTSLTAILCWALCFALSKYWRHSFERRMQTFLNFLAVLLGMSITHTPYMLQGRLLFITWLCFTLLIRSIYQGVLFTYAHRSTIVSIPTDFEELVELNFTVVMSSVNKRLLSDVPLMQKLPSITTTRLDGVSAFDYIRHESKRNYVAVGPLDFLLFYELMHNKTGHFVVMSKDLLNFQITMYLQKHSYLIDQFEHEIWWIRSAGLISGWTLNEVGEMKIAKDEKKIEVIHLQDLSAIFYMFVIGMLASLAVFALEMLSLRLKPLRSVF

>CcapIr108

MFIIKYTLYIFIAVLLVQTYSHNITHSHGMHSLGTHAHGTTLEQAIIQYFGQKTNVVHIRLQLSGSIFWKRVVLHHLTDALRNSSNITFRITIGDRYLRDNKYFQYNLWYVDSYEALNTILPYKKKHFGSKPSVYAIIMEKFGIKNNISHIQEILEKMFALNIIDVIVVVRNRNGKGFLVYSFEPFAENHCRIVKPVIVNQFIAGHFAQSELFPNKLRDLHNCSIRVSSRNVSLYFSYHKQITDGAIAMTGLEVQLLKTIAEYLNFSIELVLEESHIYGDIYTNGTMSGPYRLLDENHVDILMGFFFYDATGIAFFEQSLSYFLSALVVIVKRRVPLSKNDWILEPFQLDTWLLLLALVGLSITFIYIAHLRYNTGNWLDIIGSIFGEPRMVKTKNYLVRFSIALWFAGFVFLSGIYQAKLFDSYNKPTCGAPHTITDLLNDNFTLLVGHYWGLQYLVSDLKFPESRIEFINSTDIDTLLETLLNSSGNVASLATVARIRYFEHQHQLDEVFDQVSETVQLSEICAYFQHHSYLAVPFNKLISTIRSSGLISKWYDNEMTESREKVKKPQAGAFKRLDMRKLTIVFILLICGEFIAFIVFVAEILIVLKRYGKY

>CcapIr109

MKYKRILEYTLAILQLAHRANGQDIQLNWTDYNLLNFLQDIRRLHHYDQLVFAHNRNATFGAAFGELSGNALDEGGVDFESFQANRTGNVDYETEFVRYMMTELPTAVVLYDELSSFELKRHFSAALLIVVYLEEELMAQSALFKALVGSLKHRMQSNILFLINIDSTTASCETAFLRELFQFSWHSKMINVAALCNDYQDTQQFYSYTHLPDFALEYKHLSAELLYRGPIFPYRLSNWQGYKLPYIIGGGDPRVIVYEYDRRRVVGGFAGHFLVTFAHKYNFTLYEPLPIKSYTKFPPSQDLIKAVRNNTVEISVALTFPNIPLDGFSYPYEQVNWCVWTPVEADIPNYDFFWIVFEGMTFLLVLGAIIVISLVLSCALWQHNSKPDLLRFVIHDACLRGVIGQSFRELSRAPFVIRFIYMQIYVLGILLTTSYNAYFAAYWTSAPKVAPLRTFDDILQSNKKIYMFAPEYNELIGRAADLRKYIPMFYIESDYHKYLATRDAYDTKYLYMIPTTKWRIYREQQKVFTTPIFRLRADLCFYYNIPLGFPIHSNSVLAKILQDMVLETAQSGLTDYWMRIGFLELIRAGRLSFEDMSRKNEYKAMAVEDLKYVWMGYGIVCVLIAFVFLAEHCYFRRERVRTFFAENLWRKCCGRKQL

>CcapIr110PSE

MLFVHNITALLGLIEFSASNVHFFKIFKDIVEVTATAQQTSTLIYASNVYTNADRVRQDEMYEYVACQLHSLFKLGPMAHITVDGSTPSTSSTPYEKQFNHNLLSVVQLSEQWKSDDQLLRTLLQNLHRNRQSGLILLFADTATQAYIAGIMKFCAEENAINVIALQPNMTVKEQSYWTLQIFPEKEIIKRKLPKFYRGIFPDHLCNMHGYPLRMIGNNFPPEMYNDTTREGKTILSGYLGKTVQEYTSRHNATIDFVFLQEGRSILDMVRDEVVNGNSDTGPVZPVHIKEDRLSFTTSISDCDTCLMVPVEKTIPKKSFYYKFLCKWFIILFFVSLALVCGILALLIQRDSQQRYRLIDHCLNINFSVLQGFVGMPFWFRTRSSRLHKFISIIISFAGINFGAAYVAYLQSYIVNSPTEAPALTIDDLIANNVQIAMSKEILSYMESGEMSKLYNKFTLFKNLTELILLRNNLDPRYAYTVTNMWTIHAEQQKYFARPLFRLSEICFKKDVSLVMPLKANSIFRQSLSHFINRIVEAGLIFHWLRESFFELVAMKWISLEDPSTRQTFTPLKLEDLEVIITMFTVLNVLSFCCFLLEFFWAKIEMFFKTMKVIIRRLKRK

>CcapIr111PSE

MLFVHNITALLGLIEFSASNVHFFKILKDIVEVTATAQQTSTLIYASTVYTKADRVRQDEMYEYVASQLHSLFKLGPMAHITVDGSTPSTSSTPYEKQFNHNLLSVVQLSEQWKSDDQLLRTLLQNLHRNRQSGLILLFADTATQAYIAGIMKFCAEENAINVIALQPNMTVKEQSYWTLQXLNVVAPCALAVDSQQRYRLIDHCLNIDFSVLQGFVGMPFWFRTRSSRLHKFISIIISFAGINFGAAYVAYLQSYIVNSPTEAPALTIDDLIANNVQIAMTKELLSYMESGEMSKLYNKFTLFKNLTELILLRNNLDPRYAYTVTNMWTIHAEQQKYFARPLFRLSEICFKKDVSLVMPLKANSIFRQSLSHFINRIVEAGLIFHWLRESFFELVAMKWISLEDPSTRQTFTPLKLEDLEVIITMFTVLNVLSFCCFLLEFFWAKIEMFFKTMKVIIRRLKRK

>CcapIr112

MLLLHNITALLGLIEFSATNEDFLKIIKDIVAVTATAQKTKTLIYASNGFAECNRAEQDKVDVALQLNSLLNPKLMALISIDGYAPTKPYKRLFSLNVLSVVQLNGEWESDQQLVRKLLMNLDRNRQSGIILLFCDAVSQTYATWIMQHCAEVGAINVIALQPNMTVKEQSYWTLQLFPEKQIVQQKLPTFYRGIFPDHLSNMHGCPLRFIASGWHPQIYNYISPAGKVRLSGYLGNTFNAYAHHRNATVLTPGSYPNGLLNYPQVRNILLNNGADMGPLWPFHLREDSLGYTSTLHRFDLCLMVPVENRMPRSTFYYKVFGEWFIILLLISLILICVLLTPAINRKGHVFLDHSFNIDISVLQGFLGMPFWFRTCSSRLHKFISIIIVFAGINVNTTYVAHLQSFIVNSPIDKPIKSIGDLVNRGIMVAMDTNVYPKQMGEEYFLENYDKFTLLKNATELLVLRDDLDTRFAFPVSNMWDVYNEQQKFFGQPLFRISNICFQKGGSLLVPLPANSIYRESLNDFIGRIRSAGLTDFWLRSSFFELVKMKRITIEDRNPLEVFKPMKVEDLQYILLIMSCLISLSFGCFALEILWMKIKKQFFSNKKVDRKLSKNPQRQAIKI

>CcapIr113PSE

MQSFNNFTAILNLIGLSTVEQHFMQILTNIVISTSLERRVNTILYTSYGNNSHLETQNKLHSAKLVNALTKTTLYRPASFLHLDSTPPIIQFESVFNTELLSIAQLNNQFVTDEQLLKALWRRLWRNRQSRLLLLLDDAASVPYVTRILHMCMKQQAFNVVALZPQMSVMEGAYWTVRIFPAQTIVQRRFALDYNNMFPKHLANMQGQPLRIYAKAWYPQIYTFTPAKGNSTLSGFLGRALLEYAKVHNATIEYPLTLHRKFYTYSEFYDFFENKTIDIGSLTPIEVTDRNVSFSVVFHRIDWCLMVPMEQPLLKSRIYYSIIQKTVIILFSVCLMLTSCLWAFIMRWQSEQPPSLIEHFFNVSLFQGLLGMPFRMKRRISGVHKIICLTISLASVLIVTAYITYLQSFSVNTPITGQLETIADLLNAGIKLAISREELSLIHNNWEYRKYIRNFTVFDNFTEFLILRDSLDTGYAFTVTDMWTIYDEQQKYFSRPLFRLSDICFSRNYPMVLPLQESSVHRHHLNGFLARLHEAGLINHWTRHSFYELLQMDWIFLKDPNQHHGFKPLKLEDLNEILVAMGILFTLCVLCFVFEYFGATINKITRLAVRIVKRRKYKK

>CcapIr114

MSNNGGNITTLLHLILQVTTVHQKYLQTLTDIVHAASQDRSINTLIYTTYEHHDVPAPLHSLQSEHILTALAKRLPTQPLIHFDHTPPVEELAQRFNKDILTIAKLNQQAELDERLLKTLWQRLLRNTQTRLILLFDDLASEMYVANLMKICVKHRAFHVIALQPRMTVTAGNYWCLRIFPKEETFKCRFATDHSNIFPKPLANMHGHPIRLLQFSWYPQIYYYTPKVGGPAQLSGFVGRGLAEYARYYNATIAPTRSYQNSVIKRSDSVQLLDNDTIDLTSSTPFYTQFLDAGLPVIYYYSELVLMVPMEQPLPKATFYYNIVQETVAVLFCLTSILYTIVWQLVFRWEHKRQLTITADIMNFSIFIGLLGLPFDGAKCKTFIQKIMLFTISIAGVIIGTAYGAYLGSFIVDAPIASPVQTIDDLLERGIKVAGSMKYLIWISNTKRYRKYANNFTIFENYSAYHLKRDELDTRYAFLVTDMWPLYEEQQKYFTRPLFRLSNICLGARYPMVLALQRNSIHREAMHKHVLYLSEAGLIDYWQRHIFVEFLDMGWLVLEDRNKREPIEPMRLEDLRLVLMEMFGLLILCVICFIFELCWKKLKHIFHHLTRIKKIIMFDKN

>CcapIr115

MQILWNYTALLSVIGLSAIHEEFLQKLTEIVVSSDHQQQLDTIIYTSYENSTFRAQSSGLESPAILQNLAKGQKKPLLCIGTKSTVKKLKNNFNLNVLSIAKLTLNEELDELILTTLWARLWRNTQTRLILLFDDSASGIYIEKILFLCIKHKAMNVVALQPRLTAINNSYWTLLIFPTHRVTKRTFPKTETTIFPQHLQNLHGHPLRINIPILGIQAYEYRPRLGGLPILSGFLGRALSWYAARYNATIEYVNCTHQPGALYNEINDLIGKGVIDIGPLMPMVPRAHQDFNVCLSNPLFTSNFCFMVPIEQSLPKAAFYHKIIHKNFVILFLAILVLISALCAIVANCQNQERQTIIDFIFKMSVLGGLLGLPVGLQNERSRLHKVIFIATSFAGIIIGTAYSAYLQSFSVNAPIASRLEKIDDLIVRNIKVALGTYEMIWIKSPPEIQKYQQYFVNFTSLMEFEEMRDKLDTNYAFGVTDKWAVYEEQQRYFTRPLFRLSNICLKKNFPMVVVMQENSLFLESFNILMLRLHEGGLIDQWRRRCFWEYLDMHMISVNRDTGSFGFKSLQVVDLHLILSGWCFLMSLSIFCFILEVYWARIRSIFLRVLRFFHKMNFTNVLN

>CcapIr116

MRFTCIVLWLLLAFCHTLNAHLTQCSQDDDGSFANFSNLIDKVRAERRIETLVVFNSYATDRACLSDGTLKSLESAGITVILLSATSNCHLQGADVNSELLIVRCLSGPIQPRLLDNMINCLGVVRDVRILFMWHDNYAQQTVSRRNELQQQLRELFLYCARAKLLNVISVYNDFGNEKYYHTYSYFPSFHLVHNTLQETCFPNRLRDMHGAAIHTLPDQNEPRSIVWRDRLGRTQIGGYMARLVEALAKHHNATLSYPRAIVPDVTYEYDEIFKMLQNETIDLLMGVTGMGWRLNASQVSTSVLSSFWAPMLPVPPLRPTRDIYVLIFVSAIGVIFLLLLLTFSCLLTWEQLCARHQSSFREVSCTLTSVTHRSLLGQPTHVRISCTFIRRFICMLLFMTGIYMSTTSSSYLNTYLTSSPKRQRVLTFDELLRHPTRIKISPGEYKGLKIFVSEAFLEKYKGVFLVTSSVNDYYQQRKSLDTSFGYTITNSLWQALVEYQTKLGRHLFYISEGMHLAANLQLGVPLSPHSIYRSALNVLIQNSISAGLMKHWENLVYSDMVAAGKLNLTVATRPILAKGLTLDDLYWIWWLYGVGLMLALVVFLLECYVGRVAKSRKQI

>CcapIr117NTE

RCIPENFKPELFEAMIGCSARKRDIRMLFIWNSEYATATMQSRLKLDKQQKLIFEYCAKMNLLNVIGIYRDYLSEGHFYTFSYFPDFHLERKPLSEDCFPDRISDLKGTAIRCIPDQIHPWSIVWADHNENVQIGGFLTKLLQQFATRNNGTLTFPVTVVPNRFVDIQSWLPLFQNNTIDMVCGIIGSLGENPYLDESATIFPVDWIIMLPIPQRMADSEVYLYMLNSALGLFLLILLFVFSFVLTLESLLLQRHTKREGCLLFISFLTNTVLRGVIGQPTSLRVPHSVRHLKRFLYITLFVSGIFMSTFISASLQSYLTSSLRYPRINSFAELRRAGMKVKISRYEYTLLPAYFEPKTLKWLERVFTVVPSFDELQNQRINLKNRFATTIISPLWSVVSRYQSNLPQPAFYTSKNIYISKGIPIAVPIQKHSIYKKTLNKMIYNAHSVGLTQLWSNQVYDDMLAAKKLNTTVNLVSRRKEQLEVRDLYWLWWLYGVGVLLSTLVFFAELWYHKRSTKKANREQSEENERVEQYELNMQTFRRVALRRQLRD

>CcapIr118

MSTKHHFLLLYAFSLLTAYSTLTWNLPPETHIPNLVQLVRKINLERKIDAILIMNALNTRKCCLSDEDIKILSTNRTVRLLQAETNCSHSFADLNEDMLFIRCVSENFAPEQLDQMVVCLENRRNVRMMFIWSSEYTEVTSPQRAELMQEQEKLFTYCASKRLLNVIGIYRDFLRDGHYYTYSYFPHFQLQRKTLAEDCFPDRVKDVKGLAIRTVPDQLEPWSLVWREDNGTVNVIGFLKKFLQEYAKRINGSISYPLPVEPENVPDMSTLISLLKNDTLDIVMSSTTGRTYDVDVTAVISLVDLTIMLPIPGQMMESELLPYLFNSLLGLAALILLFVFAFLLTAEFFLLQGYTATEILCSYSEILLNVVLRNLLGQASCTRVSVSSKLGKRLLFMALLFTGILMSTLINASLNSYLTNPISYPRLKTFEDLINSGLTVKTSPDTYSTLDNYFTADNINKSKSVFSPATSSKDLNLQRNSFDRRYGYTLLSSLWDILERYQTFLPEPLFYVSEEIFIVKRQPMAFPMQKYSIFKESLDLMIDNLQSAGIVIFWSKQTYEDMIVAKKLNATHQWRKMRRGYLGFEDLYWLWFFYIFGVGISMLVFLTEMWFYSRTKQNGKVKCSIKNFNLRKVKRKK

>CcapIr119

MHSSRQFHNFLQGEQCQHAAKMLASRKSILLLLTFPLLIAQSTDSYTWNVQQDTNFIDVGRIVGKINSERKIDTILLMNARNTSKCCLSDEDIKILSANNTVKLLWSGSNCASSTERLNSEMLLIRCVPENFAAEHLDQMVICLQNRRNVRMLFIWSSEYTKVSPQQQVKLMQEQEQLFKYCAYRRLLNVIGIYRDYLSEGHYYTYTYFPQFQLQRKSLADDCFPDRVKDIKGLAIRTVHDQLEPWSLVWYDRMGNVKITGFLTKILNEFAARINGSITYPVAVQPGSFTGMGVSVALLQNNTIDIITGATSNTTHDIGYSAVLLQLDWIFMLPVPPQIPDSELLLYLLNSVLGLFLLIFLFVFAFVLTWQNLHLRRACSVRLSLHFFCWILINVVLRSVIGQPSCTRVRVSAKFLKRFLHILLLFSGIFMSTLISASLQSCLTSPLRRPRINTFPDLIKSSISVKISPGGFSLLDGLISKRYVRDLKKVLIVNTSIDELQRQRTNMDPHYGYTLFSNLWSVLARYQANLPQPLFYVSDEIYVTKGMPMTIPMQKNSIYENALSLMINQFHSVGLIELWHRQMYDDMVEARKLNATYQGQQPRNGIFDLEDFYWLWCVYGVGVTISILVFFAEIWHHRRAMKERDGARRPGVACLSIEK

>CcapIr120

MSYYPLIVLTLYYFNGNKAMRHLPAPDVQLSDGPLHNTVLRVYKEAPFETIFLLGKANGSSFNMRESTKNLEIPLIAATDNATGEKLKTHFNSKLLAIVCLSHTQLELTLLNVLAEYLQHRRQTRIILYFAEAAPSATVLAVLSAYLVDHYMTNVIGLYGAVNKSVTSTHYLSYQPFHHSHWRLKLLNTTSWYFPTKQQDFEGKTFLTLPGQNMPRCIIIVDSSGVQRLSGYVGYLVNTFAEKYNVSFRFLYPVSPGEFIHLTTLLELVANGTLDLAITLVPASFKMTNSYHLLTYPLEVNSWFVMLPCPQPLQYAEIYKIVVTKQVINVLVLLMFLFSLLDTVMNCVCRRSYADYDLLNILVNENIIRGVFGLSFFIRPHSKLSLKILYTFLLMLGFFVTNMYSAYFQTLFTSPPLQHDILTFNDMRRRNLKLMFDRHELRLVQELFGPDYNTTIKPVLQIEDTAVFQRHRRAYDTSYAYTMPESLWSIFSAQQQTFERKLYCLAPTLRLYSMLGLSLALAENSYLMAPLNKLILRVIETGLLEHWRTMTFIDLLASKQLSLKETNQRERFHEIRIEDMQLPFYALLCGLALGSVAFASEIYLFKIKNSYRQRMNRAEAGILFL

>CcapIr121

MSYTLLFALLLYSIQISVSKLLPIAESNNNAKHTFANTLLRIYKETPYETIFVLENAGKSCFATNVLESSLKIPLIRATDAAAGEQLRTRFNSNLLTIVCLSQAQMELKLLKMLAASLQYRHQTRIVLHFAEMKPTSALLAASRDFFELNYMTNVIGFYNSTHYYRYSPFPSGNWKLESANTTRFYTPLHQLNLHGKIFNTLPDQILPRSMVYVDASGQQRLSGHVGYLMSTFASKYNLTLKFVHPVTPGNIIHLTVLFDYVNNGTIDLAISLTTASFNPTRVYPLLSYPLEIIEWFVALPCSIPLEYAEIYTIVVTTQVIALLTLLTLLFSALDGIIKYLFHKRNENFSLFNILMNENMFRGVFGLSVLIKRRTVVSSKILYIFLFLLGIFINNMYSAYFQTLFTSPPLQKEILSFDDMRRRNLKLMFDRSEVQLVREALGSQFNETFRNILVLADTATVQSHRSLYDTSYAYTMPESLWAIFSAQQETFERKIYCLAPTLKFFGLLMMGIPVAENSFLLEPLNKMIMRATETGLLLHWQAMTFIDLVATGRFSLKVTGNVVEFHDISLKDIEFPFHLLLIGLCLSSVVFIVEVSAFKLKIFHTKRVLK

>CcapIr122PSE

MSYLLHAVLLLSYFNCNNAKLLATLKSEIENRALLNIIEEVYEEETFETIFLLKEVARSCVSTNELETRTKIPLILATVNATEDKLKNRFNSKILAIVCLPDEQQPPQSQLLKVLAANLQHRRQTRILLYHPAIKASAELLALLSVYLEEHYMTKVIAFFDASSAVPFAPHFYRYHPFPSSHWZLVPLNPTQNYFLTHENNLQGRTFVTQPSQILPRCIVLVDSADVPRLSGYVGYLMNTFVAKHNITLRFLYPVKAGELVHLTTLLKYVTNGTIDFAVTLVPLFYDLSETYPLLAYPLETIRWFIVLPCPQPLAYAEIYKIVITAHVFGALLIFSLLFSLIDTVIKHLFYTSHAEFDFVNILVNENIFRGIFGLSFLIRRRPVLSLKILYLFLMLLGLFVSNMYSAYFQTLFTSPPLQEEIQTFDDMRRTGLKLMVDRNEIKSIVDSFAFISNKTFQNILQLEDTGTFQRHRRDYDNTYGYTMPESLWPIFKAQQETYDSHIFCLAPSLQLFSGMSLGAPLAENSYLTAPLSMLILRVIETGLLAHWRTRTFLDLVESKQLFFKRPAQKQLFHDISVNDVQFPFYMLLGGLFGSSILFTLE

>CcapIr123PSE

MSTKHHFLLLYAFSLLTAYSTLTWNLRPETHIPNLVQLVRKINLERKIXMQLKCCLSDEDIKILSTNRTVGLLQAETNCSHSFDDLNADMLFIRCVSENFAPEQLDQMVVCLENRRNVRMMFIWSSEYTEVTSPQRAELMQEQEKLFTYCASKRLLNVIGIYRDFLRDGHYYTYSYFPHFQLQRKTLAEDCFPDRVKDVKGLAIRTLPDQVEPWSLVWREDNGTVNDIGFLKKFLQEYAKRINGSISYPLPVEPENVPDMSTLISLLKNDTLDIVMSSTTGRTYDVDVTAVISLVDLTIMLPIPGQMMESELLPYLFNSLLGLASLILLFVFAFLLTAEFFLLRGYTAMEILCSYSEILLNVVLRNLLGQASCTRVSVSSKLGKRLLFMALLFTGILMSTLINASLNSYLTNPISYPRLKTFEDLINSGLTVKTSPDTYSTLDNYFTADNINKSKSVFSPASSSKDLNLQRNSFDRRYGYTLLSSLWDILEIYQTFLPEPLFYVSEEIFIVKRQPMAFPLQKYSIFKESLDLMIDNLHGAGIVIFWSRQTYEDMIVAKKLNATHQWRKMRRGYLGFEDLYWLWFFYIFGVGISILVFLAEMWFYSRTKQNGKGKCSIKNVNLRKVKRKK

>CcapIr124

MYFLKFSGILQISIYFISFCAGVKVQNFQETHGTNTDQSYAMDSLLAAIQNESYYSTLVLWRHQPADCFAELSDWGSENLTTKLIFDSNYTEYLNGYLNRAVLNVVCLDDFVDLFMLENLAESLQHMRETRLLFLLKNFTTQPVYELEELFNFCHAQQMLNVLAVGRDFATTGDFYTFQRFPNFTVECNQWPGTFYPNRLRDLQGYEFITMHSQSEPGSIVYTDRLGNKCSSGYVYKLISNFVQKLNGSLTYRAPVNVGASIEISVLADLTSSYEVDIPIGLQMPVENMSLYAFSNIVEISNWMPMLPRAGYIERYLIYKYIFQSNTLIIDVLVFVILSLLYTCIVNGQKRRRRSATCLITWKDIVFNDKIFRGVIGLSFQLWRERNYKFYLLYLLIFLQGLIWSTIYSAQLHAYSSRPPSGEQIKSYDDLRASNVKIAIPLEEYELLENVMPVEFMQKYRDIFLVMPDLRDFYTLRTSLDTHFAYSTHTEIWAMVERQQRYFTQRLFRVSDEVQFHRQILLAVPLAENSVYRATFNAYLLDMQAYGFWAHWTAISLYEMVKAGKLTFEDLSKTRGNEALKFVDLYYIWWIYIFGIAMGTLCFIAEVVRERFKLGEAQHYLLLIWRELREQFV

>CcapIr125PSE

MALPLAHLLLYAAVACSQQRGTTFSNLFTELNHREAFQTIVLENCARNTFISEQVWAETPKLLLDTKLHHQTELKMHFNHHMLVIVCIEAENMAIDALNALANQLQHIRQTRIILWVEHKAAYLAAVRKRQIVTELFRYAQSLQMLNVIALFGDFGDFESNNLVYTFDAFPSFKLKSAPLTTGINYFPDKTCQLYGHPLYTLPDQNQPRTLLYRDTNGKLQLSGYVGKLVQAFSQHLNATLRFSQAVTIGENIYQGDQLNRTRSGEIDFSTSLGSYKFFANTYDFSYPFEFAKWLMMLPLERELEVNEMFLYIFQSQLLRYIIVISVVVSLFLSLFDYFQVSPPRKSSRSLYAILCKREHTFRGVLGQSFPLAPYNNWRMSFIZLTIFLIGLLTNCLFSVYLKTFLTRPPTKPRIRSLEDLARSNVRCLINMREIYVFKQIYGEAGWSHYRNAFEVISSWPEFLARRLSLNTSYGYTITTSLWPIIELKQSFSQRKLFRLTTDIVMDDDLVMAIPIQENSVYKTALDRFVSAVQEAGLIHYWREMTFFDMLAVGSIKLDGLNDSRAYRVLVLGDFHWMYWLFSVSYSVSILVFMLELLAFRLSK

>CcapIr126

MLRQIFNCFVCALSLGKSAAVIKINYDNFLYGSAYPTDFTREFSNLLERVQNESAYYALFLLRNRVADCVNEDLPKDLPALPFLQMNGSQQVYVRSNGNSMLLGILCIENLRDKDYLLKFAKDMQHIRTRRVVAIHNRNLTTSTSTEIASFFQHCMQVNFINVILVHRDFGATRIYHSYDQFPDFKLEAHDTRSNSVIYPNRLADVNKTKLNILADQVEPNTMFYTSKRGNLYIAGYIGHFIASFAERYNFQLWLPDSYNATTDHVVYMEEIRQATRNGSIDVGASLSTPQKESNLHEYIYPVEFYQWLTMLPIESPLETYVFFIRFFRPAVVATLFFIIWLMCLIGTVEIELTHRGLHCNRVLLWLLIIPWDLELLRGLLCQSTTARLNSLNRRIVFISVFILGGILSNFFSTNLVKWLTVPPHEQPITTFQEVVKRKLQIQISEPDIAEVKFYRGADFWNEHRNIFKIVKTFDEYQDNIRKMDTRYAYVINTLAWPIIERRQRYFEHPLFRLSSSLYYTKGSLLSLPISENSMYKDLLSDFSLRSRESGLLDHWYEMTFFMMVLFGRFNLSDLSKIHKHEVLTLKEFEWVWMAYGVGITLSLLTFLLELYWRNSCSVGRKVL

>CcapIr127

MQCQLFKSFLCAWSFGLIAANTQLNYEQFLAAQTHNGEFTLGFTNLLQRLQVENQFVSYFLLSNRPTDCVDEELPLTFAALPYLQLNESQQVYVRGNGNSMLLAILCIADLTEKNYVLKFAKDLQHIRSRRIVAIHNRKLKEVAKREIETFFRCCQQFKLINAILIHRDFGSTHIYHSYNQFPDFKLETHDLRSSPCIYPNRLADVNETKLNILVDQVEPTTMLYEDQNDTLYIAGYIGHFIAAFATRYNFNLWLPDNYDSTTERVLYAEEIREAVRNGSIDVGASLLTPQREMNLHEYIYPIEFIQWLTMLPVETTLETYVFFITFFRPAVIVCLFLIIWLLCFVGAVQVELACRGIHCNRALIWLLLIPWDLELLRGLLCQSTAVNVKYLSRRIIFILVFILGGIVSNFFSTNLVKWLTVPPHEQPIETFRQAAARKLHIQLPEAAIADVRFFRGDKFWLENRDVFKIVKTFEEYQANIRKMETRYAYVLDTLAWPIIERRQRYFEHPLFRLSDSLYYTKGSLLSLPISENSMYKDLLSDFSLRSRESGLLDHWKDITFFMMVQFRRFSLKDLSVVPRHEVLTLKEFEWVWMAYGGGMGLSFLTFLYELYWRKFYNLLVKKS

>CcapIr128

MLSQALAVLSYLSRWQVNAAHAVDNFEKFSLLQSNTADFTRGFQRLVQRIQAENYFVSYFLLKNQPADCVDEQLPLNFALPFMQLNESETIYVRGSGNSFLLAILCIDKLTKKDLVSKFARDLQHIRTRRVLAIHNGNLTASAPSDNENFFRHCMQEKFLHVILIHRDFATTNLYHSYNQFPNFELEIRNLQSRSPIYPNRFENVGRTKLRVLPDKIAPITILHEIGNKTILEGYIGYFLKTFAARYNFQLWLPDNYVLSANRIISMEEIRQAARNNSIDVGASLSTPQKAANLHEYIYPIEFFQWLTMLPVEPPLEAYYFFITIFRPTYLLAIYIIAYCVCIIFAIEVKLRGGNFRWDRSLLLILINPWNLDLLRGFLSQSTAMRLNSATRRIIFLQIFAIVGALNYTFSRHMFNWLTVPPHGQPIGKFSDLAEHNLKILIPEPDIAEISFYRGENFWLEYGHIFKVVKTFEEFEDGLRTMDNRYAYIMDTLVWPITEQRQMYFKHPVFRLSNELYYTKGALLSLPISENSMYKDLLSAFCLRAQENGLLDHWYKMTFFTMLSLGRFNLEDTSDYRGHEMLTLKEFEWVWIAFSVGMGLSGLTFVLEGVWYKFTLILDK

>CcapIr129

MAKVQLFKCFLKFSLLTILTNLTNANLLLSNVYNKQMPNKMLELLDNVRGERGCDTIAFCLSTAQECIFEQLLPEISTPVLLIHRRLEYDFKASYSRELIILLCLADEGVATAFESFHVFRGLKQTRIIAYAPQASDLELIEKYCAAAMTADIYNVLIIQEDFSATHNYYTCNRFPLDAPPYKQKSLTDVSSAIFEHQFRDMHGMKMRTYPDQLQPRTMLYFKPDGKLVLEGYIGRLLRTFAEKRNAALTIEHPYKVGKTTYYGDLLALALNNTVDVAAGLAFPTSLKDFEVMSYPVQTLDYCFMVPLPEAVPINELFVGIIRLPTLFWIFVFIVIFAALLTHLNKGKRLTFINLFLNDKSIRGMLGQSFVAPMNPSLKVKFIIFLLCYMSTITNTTYQSYLQSFLTHPPLQPMYRSYEDMAAAGLKISFPINEQHMLTTNKSVAQHKELFLIAKDYDAWLRDRSAMSVKFVYPVSNVRWEVFDFQQSLFSRPIFYFNTDLCFFKNSFVCLPTRPDLPYGDLLDDYILRLHSSGIMNEWISLNFYILAKLKMVFFEDLSTPWQSGRPLDLNDFFWIRMQFFGSLALGFVAFLGENLYYHWNREKKEKNNSMKKIMKIGNDFNKKLIK

>CcapIr130

MQLQKCFGIIFILKLYCVKIITPLVMEASIDFGDTFRKILKEIKEEADYKTIFVQRRHIEDCVDPSILQHFNAPTVIWDETHYGDILYDYGTHLLVILCLKTVDDGDMMHQLAESIHYMRTARILCIVNESLLLLDELKISKFFEMCAQLEMINILVLHRDSFIFEKYHSYKRFPNFALETHDISRNEGGRKWYPDRLKDFVGANLTAIPNFNTHRVFIEKAVDGNVDGNVELCGSLARFAQTFCEFINASLLYTGGELYDYFKIIDAMEMGHIDIGLDFDRCAYRGQSRVLEISRMSVVVPLNVVPPLSALSSVFLNRYTLLPFALQTVVLPFVISKFLQWINGRWQPIGIWRAVAFIGDTGLRSFLGQSLNLPHQQTIRIRFLFGLLLFGGMIIQTFFAANYARLQTFPPLRPKVVDYEDAMRKGFKIYTHSEEMIDFLAIDVIKQKYKSLFFEDLDKFQKPMKLLTDAGYLVDRISHEIFDQGSLKMVFNENLDITDMILLSYMVRNGSALKQPIDDFTDIVQESGLYKYWERNPCKRRRITASHGTYEDPSAVQLTVSDLHGVWLVYVGGHIICVFVFLCELFGRRFLALFRSQH

>CcapIr131

MWHFKNLIFMILIALFGGISGQTETFIKGMGNEKREFYKNLLMKLDAEETFESCLIYGDMRRNLHLEVFLKIIVEDMQKPMLLHSVAPSYEVVKRFNRNILTIALVVNVDTELAILAETLNLMLQKRVLLICGEEQAAREREAYLTRLFRLCETKKMLNVVMIFSDFSSTKTFHSYTIFPTFHSERKSYATADVVVFPRRMSDLHGYGIRTMPDQLFPYSYADNIDGRVKVAGLMPVILGTFAASINATLTYPMPVIVGETHQQAGFDEMIKRNELDIPAARMEIFDTFDEATQPYAIGDLCIITPTQRYHSFIKFSLIYMDPTYLMVELVCVMIIHTLLHFIRRLQFFRVGKVFHYNLLTSYTEPNYMSNHLAVVHTARLPRLISLRILTFLTLIWSLCILTQFTVNLNSFITKPVLVASPKTWQDFNENGYKILLSIPSYKYISTYCGELCEDMEDAVEYDTFEEMSNNFFRLNTKYAYAIDMFAWLYIKLKMEMRGEKIFTLSNICVRTHIFHSVQLHPNSVFKDSLNLFLGRAFDHGLQKAWIDFSYLAALKAGKMNQTCHGSAHINRPLDMDFLSFVGPMFGSLWTLSILVFAFEMFKAKKDRK

>CcapIr132

MAQRNIRNLLLLLAVLLRLKTVQTTLLAENVMEREREFYDIFLVKINAEERFDNCLLYGETSTSETLEILLKTVTQSLDKPILLQNNACSEYRVIDMFNSNMLILVRLENLEYDLSSLAETLHYIRHKRIILVGGTPKSLRDHEEYLTRLFRLCAAQKMLNVVAIFEDFWRTRIFYSYTIFPTFEIERKSFDAVGGVVVFPRRMNDLHGYGIRTITDQMFPFSFAYEQVGEVKVTGYLPGLLETFASSINATLHYPKPMTLDDYYRPAELINMTSNNEMDIPASRMFVQVTKTFDHASKPYAISGVCIMTPVQWYYTFRQFCSLYTDGAYGFILFSCVWLTYCLFYYCRRLQYFRAYRVFLVNFWTAWIEPNHFAFNIGAGLPYTLPTLATLRILIFLTLIWALCVETDYAASLNTFITKPAVMPVPRNWAELSKSNYKILFGWSFYNYISQWCGESCRDIEKSAEIVETAQEFRSQLHNLNTNYGYPVDLFSWHFVQLQMKLLVKPIFHLTNICLRRQIFHCFPLHPNSVFREPLNLFLTKFLAYGFHEFWIDATYLEAKRYGRIHDVSPVNRGEQGPLDMEYMSFMWPLFGAAWTLSFGIFACELGMARYHRLKRNVKKS

>CcapIr133

MRVTLAEFNLNYLNLSSAHDNTRNSKSIKEKVMDFNAIIILVLLSAVIVTQPCPMGDATTTKSIMKMERVKFYQKFMAKIWAEERFESCLLYGDLQASETLQIFLQILFEDLSTPILLQTTNSSFQIANIFNRNVLSLVRLEDFERELPKIGATLELLRQKRIILIGGEQQVTNADDQYLTKLFRLCETKKMLNVLVVDGDFWQTETYYSYTIFPHFRLERKHLETWNDLVFPPRLSNLHGYGIRTLPDLTAAWTFLYLQDGRVKVGGYLSCLLTIFAETINATLIYPMQVRVGEIINSTTIDNFMQNNLLDIPMKHIFLETANTIDMSSRPYGISDICIVAPVVWYHSVTDFLLTYFDGKFSALIFISYFTTIILLHLCIRLRRLRAGRPLHGCILASYLEPSYMAFNYGPAQPRGVQSLVTLKILISLSLVWFLCIQTHYAANLKTFVTKPVEMPVPKSWEDLNNVGIKVMMSRHFYSYKRFWCYESCARYERFLKISESQEITRKYMESLNTSYAYPTDLFTWHFIKFNMRSLSQRVFRWTDICLKRQTLQGFLLPPNSAFKDSLDLFLTRIADSGLANHWIEVTYAEAVRLKKINKLAFANRDWTQPIGMKSISFMWSMFAFGWTIALAVFICEIALAHWKKPLTRRNK

>CcapIr134

MLCIPQWVGAIKCVRKLRRTVFKSVNLENLTVSYHRKGATLRAVQEMLLIFKLRALLLSYACLNSLLQPVQLWSVEYNLNLLCKMGKMVRTQELIWYVSERLDTADSAGIEEFIRALHDCLGITQTVLSNRTDWRFIQTENRKNSLSVIFCTAPDDPIIYVYDRTQLGRHFYFGWLIYMNTVSDFVVVDQLLSALSEHSFHNTMLYYYSTNETNEVFASTLFPEYHHVNRSDFLVYLATMFGKLVAGGMDLQGYKFYTPLQQDLPHVFSYMPRDSPISWRGSAYNLLKLFLNYGNATLARYEMPKDRLGGDVIDMKAALELIRRKKIAVMAHAYALFKEDDEQSKSYPLMVVRWCLMVPIWNSVTTMFYPLKPFDDFIWFCMLCVFVALVFIRCLWCCWQGTHQLAARLSDNILQSFCLSIGMATSHFLGAPSVFDFLIFTTIFFYGFFLTANYTSLLGSIFTVTLFHAQINTMQDLIETNISVMIIDYELEFLRSTGDDLPVNFTRLILPVDAATFTQHQVQFNSSFAYFVTEEKWRFLDLQQKYLKQRLFKFTDICFGSYHLAYPLQPDSFLYRNLEYYIYRMHSSGMLSHYESTAFDYAVSAGLMKRLADSSEYTSAGMQHLFVVFLMLLVMCGLGVVVFMGELMKYKSSRHRTVSARM

>CcapIr135PSE

IENFTVLHTPFYDDLPRTFVRDDSKMSEETLFGPYAQTIWNFAYTHQMQLTFDRNISVGNRAIYERIYSGECNLSMNGGTIVRHASADIQFSYPLYEVKNCVMVPMGAELPKYWYIVWPFGRYIYMCILFGIFYVAILMRYIEYRTNETKPTRSITRNILHSSAIIMFSSNMNVQLKNAHVRVLIFYTLLFVLGFILAAYHXVNTVEDLLAANIKVFITLNDFMEIKHNSVENMLGLSPLLQEIPKQKFENIFDNLNRNYAYVITEYQWKFLNRQQRVLVQPYFHLSNICFGTVFKAIPVRCGAAWFDALNLFILIVHETGFWIQWEERAFNEALRTKDAQILKDAYPLRPLNLAFFTIAWIVLAVGLTLSCCIFVFEYYLKDGNRGRYLYDK

>CcapIr136

MFTAPQVITGTFLLATLLVLAPYAHTWDRAYVRKYLLAMMPYIQPQEMAWFVSEQCNTEQISDVTEFMRTLNGELGLTQTVLTNRTDIHFIETTMKTHIVSVVFTTGPSDPIMNVQAHSLRDRHLTFNLIIMLNVVSDFDVIRQFLYELALRGFHKPLLYYVSADNGNEVYGYTIFPVFTILNRVNYSAELEAEFTQYIAGGLNVRGYKLRTPLRQDLPGVFRARDTCNDTKCMQGTTFNLLSLYIDYINATLVSYPMPQDRLGGNVIDMKAAFDLLRRKEISFLAHAYALFYDDESISLSYPVGVVRWCLMVPIWNSVSTMFYLLEPFDRMTWFGIIFVFGALLLQQWLWCCLQGTNELVMRLSDSVLRSLCLCTGINIPHSFSAPSALEFLIFTTFFFYGFFLTANYTSLLGSILAVTSFRAQFNTMDDLVAANLSVLIIDYELEFLHASDIVLPANFSRLIQPVDAATFIALQYSFNTNYAYFVTEDKWHFLDLQQQYLKQGIYKFSKICFGSFFVAFPMRRDSFFYRSLEYYIFRMHSSGLLAHYERTAFDYAVQAGLVKRLTYNSEYTSAGMQHLMVVFFMLIAMHMLSLLVFLSELLYHRAAGMRRGRVSEQRTGSDERSTSVKKTVSQ

>CcapIr137PSE

MSIENFTVLHTPFYDDLPRTFVRDDSKMSEETLFGPYAQTIWNFAYTHQMQLTFDRNISVGNRAIYERIYSGECNLSMNGGTIVRHASADIQFSYPLYEVKNCVMVPMGAELPKYWYIVWPFGRYIYMCILFGIFYVAILMRYIEYRTNETKPTRSITPEECTRTRTHLLYASLCARFHFGRVSCALPPVNTVEDLLAANIKVFITLNDFMEIKHNSVENMLGLSPLLQEIPKQKFENIFDNLNRNYAYVITEYQWKFLNRQQRVLVQPYFHLSNICFGTVFKAIPVRCGAAWFDALNLFILIVHETGFWIQWEERAFNEALRTKDAQILKDAYPLRPLNLAFFTIAWIVLAVGLTLSCCIFVFEYYLKDGNRGRYLYDK

>CcapIr138

MWTLTLLFASTLPLHANSAYDNVSLSAATTFDAVLQRTLRDHRIANVAIFASDNGDNYTGFTALMASLDRHLLAPRYIFTAADNSMPPRNTLRHHIDNDALSVVLCAASTDRIWHVVDQRLRKLRQIKMIVVPTSRACTVQLTAVFAKLWQLQFLHVVLLCNSTIYRYTPFPHLRVNQVANRSADLFPPASTDLQGYTISTPAENDLPRIFFVRPQPGKQYNIRGFAFHIFTNFLRRLNMTLRISNPQQVHDITSSVDTSKIVHQIASQELEISMHPYTSIGEQQGIMSYPIFKLECCLIVPVQNEIPRCFYPIRPLKLGSWLVIFGAVVYISVALAWTSPCVREDAPFLTKLSRTFLESIAQVVFLPSTYQSHQPSLRYLLVYLQLALFGFLLTSWYNNLLGSFFTTILVGEQLDTFESLIAAQLPILTKFHEIDMVLEQVPPELVTKVRKLIVGSNSSVQMAHLRKFNNSYAYPVTEERWSFLSLQEQYSNKPINRFSKICLGSPCIGFPMRLDSHLEPHLTHFILDVQMAGLDFYWLTSDFKDALKAGYVKLVNNVLPFKALDLYTLYVAWCLLIAGLCLSTFVFVIEIRGSCARRKNRYSESSAP

>CcapIr139

MDPESANAYQLNTTFVIEVLHAIYEFYLFKNVVLYISEDYERTESAANFFNEYFVTFPLVPSIILISGEHLDPNDYRMNMLLSKPALSMVFTTGPNDTVMSLAAESLKHLHVLKTIFILTTNTEEANAYWDNGSAECDISTLINETYAWIWAEEFLNTILLTAQDNVFILDPYPELQVINKTGNWQVKDFFIDYYDDLKGYVLNTVIRYDLPRVFNHRHVNNRLQLSGTSGKLFTTFMKSINATIGDFDMANSPYEPAVMGEVIELVANRTIELSPHSLTALFAIDNIGTSYPIGINDWCIMVPFYNSSPEHLYLLHSFQNSTWLLVLFALIYISIALWLCTPQRPREYSTALLQALCSMLSIAPTTFFNTHNLRLSFLFFYLFVLGFITSNWYTTKIASYLITSLPSAQLHTVDDVVRAQLRIKVFDYEYKRLETMPEQYPQRFLAQVDIVDKQFMDQHRETMNTSYGYSIQTDRWEFLNMQQQFLQKPLFRLSEICMGPFHHVFPMSHDSHLQIPLKFFIMHATQSGLLTYWQKSAFTDALSLRLVKIMLIHEDPRPLSMSFLRPIWCVWVLGLILAFIIFVCELKNRFLVRLKRKLLLELG

>CcapIr140

MLWFKPHVSAALLQLIANDTMEELTHQRRRDAIDRIIATVQAQRAIFSIAYFGAISDIEPYLTAANTTPKFVITHGCNDPLGGATVADKFSSNIFYIAVGQLLRNPMWQRMNDVLADIQSRVRGLFVVPRHLTRRQVPLGQLDEHFEWCWRNGFINAMVLVDDGAAKDVERPFAVYGYDRFPQLSVHPMPSHAVWFSDKMKNFQGSSLRSTYQYDPPRVFQIRKSWAENDYQTVGYAAEMLMALVKTHNATLDTMPLNNSSRYNIMSIVDQLRRGQLDISMNPFFFYPGVRLSYPVRMLQFAIVVPSSGEIERFHYFVRPFQTHTWICFMVGLLYLSLVRRLSEHISVYWQAQMRPSFGRACLEIWRLLLFLPINTPIERRCFHWHAASTFILTALLGFILTNLYQASLTSFLATSVFRPQLNTLMALLSHNVGINTNANELEFIRNSSSLPKDFTRLLIAREPTELRDELLALQPRAYTACADVVHFALEQQLYMDRKPLHECEESITTLPFGFMLPNRSPYEHIVNRFILRAEAAGLTVKWLEGSKTDGFRAGILRKWIRNLPLKRPLNLEYFQFAWLVWTVGLLCALCGFVLENLRHMMQQKKRAMVRYCLNGILRILEKA

>CcapIr141

MKIKVFLIISLQIVIFQTVCALKTCDVIDKLNIDLRLELNIFLNITEDSADELCKHSGLTPKIILDYEHADLLELNGWATENVLTVAFVGVKNKTKPISTLVKLLWQLHYTNVLFVYKSSMSGVQLYELFALCWQHGFVNVLILIQQTLYTYHPFPAIKVLELRTLSAYYDKSHLEDFQGHPLRSSISNNAPRVYHYTDDDGNLANGGYLYALLLTFIRQHNGTFQEVKMPTYDLNLTQTIAAFNNRDIDILAELLFMYPEYAHSAVVCIYRTLLMAPYAKPLQSYLYILQPLTRFIWLLIIIAFCYSLIAQMLLSRLRTRRFNFGAALLRIVSGILYLPTFYYRNCARTQIICAIMLLASGFLLTNFYQTSLASMFITRLYEPQINTIADIAHTNFRLPITKTDAAYLNSLAGVPEIINDRLIEMNPLETYTLLRDLNTSYVLSSMEDKIMFFMYQQKFLRIPRVKVIDEELAEVPLFIALAHGSPFIQQLNRFLGQIFDTGIFQKMVSDSAEEGILTNEIRFFRTVSAKFYPLSVQNFSMIFVAWAFGLMCATVCFLIEWKFLKK

>CcapIr142PSE

SIKPAINGIRNVILILSAFKLSAWVKNSLITSKVMLDTEHLDLQLIGRVIGKVLTNVFMSANATQRTMARMVKLLWKLLYGNYYTLHKCATSEDVELMZKLFRRCWQHDFVHVLAANKLKLYKYHPFPAVKVAELRTLNEYYDMSHLHNFHGYPLRMIVTSNAPHMZRREDVNGKTVTAGYFYALLMLFVKHHNGIFQEIPRPLSPPLRANISKYFDESLIDKSTDSFZIKPDYSYRNIICLINTLLMALYDKPIKSYILRPITAYMLCLIGPLYVYVILVQLLIPRLQGSTFNLTALALCNYSSMLDLPTTNCRNRKRAQKZCALLVLGFLMMRCYQSLLTNILITRLCDTRINTLADFSKFLTSIDADYIRSTNTLPKYHSDRILATSYQZRQELNTNFIDSGLEDKVLFIPLFRVIAESLQQMSLYICLPQGSPFILQLNISRFLENEVKAF

>CcapIr143

MWTMDVEFKVIWLICLLSLPFEGSAHGNITAIREKVKELNEHGNIETNIFIYNKSEVQIQEYINKGTPTILVNRQKTNFTLIYNYNKSLLAIVLLSEQNKTELLLILNELLMRLRHLKVLLILPATLSHEQSQLELFRWCWQSGYTQVMSLMAHENTLTLTSYSPFPAVHLVAVERTADYFRSNLPHDFQGQILRTPLAYDPPSVFRYTDRYGRQQTSGMMYKLFLHFVQIHNATIEEVILPPPYPGTLVYENIFEAMRAGKIDISVHVYFTDVAKWHVSETPFLFPHYFRVPYARPLASRDYIVRPFRPEVWRLLFSYLLLAAMLFTFATVIAVKIKRLRRIAQCQRRGEIRIKQKKSLSSLNGQTSKVRLCFLQLRRTLLRYLFTLIDITMELFALLQQSSNCYGYQYHNHFCLTLMYACHNMLAFILINYYNKLIVSFLTTGVFEAQLNSVEDILASPYAIQLTRTDTPYFIDDPRLLRKVKIVDRNELWQQHISLNNSVILLSSMLYYNFFAAQQDYLRAKRVRIFLQEVIWDVSCGMFMPLESPYVEPLKETLLWSFNTGLISKDYHEAKSEAFTAGMLQFMREENVWGSSLKMDFFCWAWCVCAVGYACGGIILIFELIWHKMVK

>CcapIr144

MWTMDVEFKVIWLICLLSLPFEGSAHGNITVIREKVKELNEHGNIETNIFIYNKSEVQIQEYINNGTPTILVNRQKTNFTLIYNYNKSLLAIVLLSEQNKTELLLILNELLMRLRHLKVLLILPATLSDEQSQLELFRWCWQSGYTQVMSLMAHENTLTLTSYSPFPAVHLVAVERTADYFRSNLPHDFQGQILRTPLAYDPPSVFRYTDRYGRQQMSGMMYKLFLHFVQIHNATIEEVILPPPYPGTLVYENIFEAMRAGKIDISVHVYFTDVAKWHVSETPFLFPHYFRVPYARPLASRDYIVRPFRPEVWRLLFSYLLLAAMLFTFATVIAVKIKRLRRIAQCQRRGEIRIKQKKSLSSLNGQTSKVRLCFLQLRRTLLRYLFTLIDITMELFALLQQSSNCYGYQYHNHFCLTLMYACHNMLAFILINYYNKLIVSFLTTGVFEAQLNSVEDILASPYAIQLTRTDTPYFIDDPRLLRKVKIVDRNELWQQHISLNNSVILLSSMLYYNFFAAQQDYLRAKRVRIFLQEVIWDVSCGMFMPLESPYVELLKETLLWSFNTGLISKAYHESKSEGFTAGMLQFMREENVWGSSLKMDFFCWAWCVCAVGYACGGIILIFELIWHKMVK

>CcapIr145

MQSCSRIFLVLLFSNLYVKVCASELNILIKNYTAGIGAGTCIYYNYAVEAHNESSETWQQCSAQIVLNKQPSIPFFEYFHSNILNVVFLNSAPVETQLENFKYATQRWHSRDVLFVVAKNGNSALSVYTTAYALFNWCWHNGYSNVLLTDASGNELLTYQPFPAVRLSNITLYAYLNKRNAWWRNFYGYPLRAVSGTQPPRAIIHTDEHGVQQISGLKPLIIKLFAERYNATLNFTVDPNPDYDCIQCIPEVLDGLYDICADSGFYRAYNKPLSKPLHLDVAYFAVRFPQPLAKFRYFLAPFQAATWWLFIITIAYITLLLAVSNWYVRGHWEVGQYLLDVISSFINASVHANLINERYGVCVFALLTVAGFIISNYYLAFLSSLLSTNLYEYPIENVEDIIAANITVLTVPSYDLVLHNYSAFKALRIKYIEAGALKVYRDRLDPDFAYINMLDIWQFLLSQQRFLLRPRLKLLKKPLLSYVSGMPLPRYWPFEQLFNRHVQQLFEFGFHQYFMRCTTEFAIKTGYLRFFQTEYNRVVPLNLYDFEMPALLLVVGYLLALMVFLMELVWCRLQGWRV

>CcapIr146

MNAECGKLKTSLENILITFSTPSQYTFSAVLVKYTNFREGMYRFLLLFTFFVLHSQVEATNIVELVNNLSQELGVTTNIIYAEEGATHLPEIASELTQPKIIINANISCKVRETFDTPLLAVVLLNNEFSSEHGFHKIVLSTLVRLQHTDVIFYAPHELTAATENWLTLMTLYLNSGFHNLLIADKRAQLQTIQTFPQLQLMRTSFDAYLQQRQTWWRDLKGLRLRISYAHDPPRTLIYRQRSTGQRQYTGAAPQIFAAFAQRYNATIEPWIAPNSADFNIDHVCSERLRARVVDACSDWGVYSAESVVSMPLQLYNCYLVVRYAPPLSKLYYFQVPFQPAVWLLLGVSIFFVTVVTSLLVRLQCGEWHLGRLGLNVCASFLYLAFDLRPLGWHLRSQLFLTLFVGGFMLSNLYIGYLSSILGKSVYEPQIRTLEDFRRSNITVMAHEYQDFVFQQYGVPAVIADRLLIVSYEEFLVHRNNFDTQYAYMNPEITQELFMYQQKLLRRPLMRKLPNPILVALAGHAIKEGWPLEEAFNKHSLEMFAAGLYKRLKEEANAKTILLGYVDFAKRERLDVKPLGMEYIAMPALLLGCGYSIALITLLLELLRDKMLARKKLY

>CcapIr147

MRKLLLLLGGFFIILYTHDGEATKLLDIINTFNAKINATATSFFLNPYTSTTYERALRQEAPPKIIFSLKSPAKLVKELRNAYMLYVVFLPPPYAEQQQQLNNLLHAIGRISVKFFLFVSTEYQDTRWLFKWCWEKKILNALLTTNDTPMLLTTYRPFPHLQVYNTTADAYFEQFHKKLDLRGYPIRYTGGLNMPRSLIFKKQNDEWEVHGYLVQVFELFAANFNGSLQHVIRTENYTFEDCAKLTEEHQVDLCAELYPFGGLMHLSKPLHMTGLYILAPRARRLSIFYYFSAPFTIELWLLIALTFISLTAVLALMAKYRYSDWYFGRLLLNLVASFIFLPFHLRPFTGCAHCLLYIILFTTGFLFSTLYLAFLSSIFSADIDEQQISDVAGLKKANVSILIDETDVAILQLYNAPQALLERARVELFSVIRYRQLALDTRYAYLCIVDKCSLLMSQQKFMLHGKVYMITPGVMSIFVGVVMAKDTFFDVHFNRHLQRVFEAGIMARLQEQSKEEAIRLSITKYFPTEKRLIESALTLEYMHMPMMLMVIGFVIAFMVFCMELLIHRWRKPRANVLELSHRVC

**References**

1. Leal WS: **Odorant reception in insects: roles of receptors, binding proteins, and degrading enzymes.** *Annu Rev Entomol* 2013, **58:**373-391.

2. Vieira FG, Rozas J: **Comparative genomics of the odorant-binding and chemosensory protein gene families across the Arthropoda: origin and evolutionary history of the chemosensory system.** *Genome Biol Evol* 2011, **3:**476-490.

3. Kwon JY, Dahanukar A, Weiss LA, Carlson JR: **The molecular basis of CO2 reception in Drosophila.** *Proc Natl Acad Sci U S A* 2007, **104:**3574-3578.

4. Rytz R, Croset V, Benton R: **Ionotropic receptors (IRs): chemosensory ionotropic glutamate receptors in Drosophila and beyond.** *Insect Biochem Mol Biol* 2013, **43:**888-897.

5. Koh TW, He Z, Gorur-Shandilya S, Menuz K, Larter NK, Stewart S, Carlson JR: **The Drosophila IR20a clade of ionotropic receptors are candidate taste and pheromone receptors.** *Neuron* 2014, **83:**850-865.

6. Forêt S, Maleszka R: **Function and evolution of a gene family encoding odorant binding-like proteins in a social insect, the honey bee (Apis mellifera).** *Genome Res* 2006, **16:**1404-1413.

7. Findlay GD, Yi X, Maccoss MJ, Swanson WJ: **Proteomics reveals novel Drosophila seminal fluid proteins transferred at mating.** *PLoS Biol* 2008, **6:**e178.

8. Scolari F, Gomulski LM, Ribeiro JM, Siciliano P, Meraldi A, Falchetto M, Bonomi A, Manni M, Gabrieli P, Malovini A, et al: **Transcriptional profiles of mating-responsive genes from testes and male accessory glands of the Mediterranean fruit fly, Ceratitis capitata.** *PLoS One* 2012, **7:**e46812.

9. Hekmat-Scafe DS, Scafe CR, McKinney AJ, Tanouye MA: **Genome-wide analysis of the odorant-binding protein gene family in Drosophila melanogaster.** *Genome Res* 2002, **12:**1357-1369.

10. Scott JG, Warren WC, Beukeboom LW, Bopp D, Clark AG, Giers SD, Hediger M, Jones AK, Kasai S, Leichter CA, et al: **Genome of the house fly, Musca domestica L., a global vector of diseases with adaptations to a septic environment.** *Genome Biol* 2014, **15:**466.

11. Siciliano P, Scolari F, Gomulski LM, Falchetto M, Manni M, Gabrieli P, Field LM, Zhou JJ, Gasperi G, Malacrida AR: **Sniffing out chemosensory genes from the Mediterranean fruit fly, Ceratitis capitata.** *PLoS One* 2014, **9:**e85523.

12. Kim MS, Repp A, Smith DP: **LUSH odorant-binding protein mediates chemosensory responses to alcohols in Drosophila melanogaster.** *Genetics* 1998, **150:**711-721.

13. Jones WD, Cayirlioglu P, Kadow IG, Vosshall LB: **Two chemosensory receptors together mediate carbon dioxide detection in Drosophila.** *Nature* 2007, **445:**86-90.

14. Ai M, Min S, Grosjean Y, Leblanc C, Bell R, Benton R, Suh GS: **Acid sensing by the Drosophila olfactory system.** *Nature* 2010, **468:**691-695.

15. Min S, Ai M, Shin SA, Suh GS: **Dedicated olfactory neurons mediating attraction behavior to ammonia and amines in Drosophila.** *Proc Natl Acad Sci U S A* 2013, **110:**E1321-1329.

16. Grosjean Y, Rytz R, Farine JP, Abuin L, Cortot J, Jefferis GS, Benton R: **An olfactory receptor for food-derived odours promotes male courtship in Drosophila.** *Nature* 2011, **478:**236-240.

17. Clyne PJ, Warr CG, Freeman MR, Lessing D, Kim J, Carlson JR: **A novel family of divergent seven-transmembrane proteins: candidate odorant receptors in Drosophila.** *Neuron* 1999, **22:**327-338.

18. Robertson HM, Warr CG, Carlson JR: **Molecular evolution of the insect chemoreceptor gene superfamily in Drosophila melanogaster.** *Proc Natl Acad Sci U S A* 2003, **100 Suppl 2:**14537-14542.

19. Hallem EA, Carlson JR: **Coding of odors by a receptor repertoire.** *Cell* 2006, **125:**143-160.

20. Syed Z, Kopp A, Kimbrell DA, Leal WS: **Bombykol receptors in the silkworm moth and the fruit fly.** *Proc Natl Acad Sci U S A* 2010, **107:**9436-9439.

21. Kurtovic A, Widmer A, Dickson BJ: **A single class of olfactory neurons mediates behavioural responses to a Drosophila sex pheromone.** *Nature* 2007, **446:**542-546.

22. Ronderos DS, Lin CC, Potter CJ, Smith DP: **Farnesol-detecting olfactory neurons in Drosophila.** *J Neurosci* 2014, **34:**3959-3968.

23. Jang EB, Light DM, Dickens JC, McGovern TP, Nagata JT: **Electroantennogram responses of mediterranean fruit fly,Ceratitis capitata (Diptera: Tephritidae) to trimedlure and itstrans isomers.** *J Chem Ecol* 1989, **15:**2219-2231.

24. Siciliano P, He XL, Woodcock C, Pickett JA, Field LM, Birkett MA, Kalinova B, Gomulski LM, Scolari F, Gasperi G, et al: **Identification of pheromone components and their binding affinity to the odorant binding protein CcapOBP83a-2 of the Mediterranean fruit fly, Ceratitis capitata.** *Insect Biochem Mol Biol* 2014, **48:**51-62.

25. Bellmann D, Richardt A, Freyberger R, Nuwal N, Schwärzel M, Fiala A, Störtkuhl KF: **Optogenetically Induced Olfactory Stimulation in *Drosophila* Larvae Reveals the Neuronal Basis of Odor-Aversion behavior.** *Front Behav Neurosci* 2010, **4:**27.

26. Lu T, Qiu YT, Wang G, Kwon JY, Rutzler M, Kwon HW, Pitts RJ, van Loon JJ, Takken W, Carlson JR, Zwiebel LJ: **Odor coding in the maxillary palp of the malaria vector mosquito Anopheles gambiae.** *Curr Biol* 2007, **17:**1533-1544.

27. Peñalva-Arana DC, Lynch M, Robertson HM: **The chemoreceptor genes of the waterflea Daphnia pulex: many Grs but no Ors.** *BMC Evol Biol* 2009, **9:**79.

28. Chipman AD, Ferrier DE, Brena C, Qu J, Hughes DS, Schröder R, Torres-Oliva M, Znassi N, Jiang H, Almeida FC, et al: **The first myriapod genome sequence reveals conservative arthropod gene content and genome organisation in the centipede Strigamia maritima.** *PLoS Biol* 2014, **12:**e1002005.

29. Saina M, Busengdal H, Sinigaglia C, Petrone L, Oliveri P, Rentzsch F, Benton R: **A cnidarian homologue of an insect gustatory receptor functions in developmental body patterning.** *Nat Commun* 2015, **6:**6243.

30. Robertson HM, Kent LB: **Evolution of the gene lineage encoding the carbon dioxide receptor in insects.** *J Insect Sci* 2009, **9:**19.

31. Erdelyan CN, Mahood TH, Bader TS, Whyard S: **Functional validation of the carbon dioxide receptor genes in Aedes aegypti mosquitoes using RNA interference.** *Insect Mol Biol* 2012, **21:**119-127.

32. Obiero GF, Mireji PO, Nyanjom SR, Christoffels A, Robertson HM, Masiga DK: **Odorant and gustatory receptors in the tsetse fly Glossina morsitans morsitans.** *PLoS Negl Trop Dis* 2014, **8:**e2663.

33. Fujii S, Yavuz A, Slone J, Jagge C, Song X, Amrein H: **Drosophila sugar receptors in sweet taste perception, olfaction, and internal nutrient sensing.** *Curr Biol* 2015, **25:**621-627.

34. Kent LB, Robertson HM: **Evolution of the sugar receptors in insects.** *BMC Evol Biol* 2009, **9:**41.

35. Miyamoto T, Slone J, Song X, Amrein H: **A fructose receptor functions as a nutrient sensor in the Drosophila brain.** *Cell* 2012, **151:**1113-1125.

36. Zhao C, Escalante LN, Chen H, Benatti TR, Qu J, Chellapilla S, Waterhouse RM, Wheeler D, Andersson MN, Bao R, et al: **A massive expansion of effector genes underlies gall-formation in the wheat pest Mayetiola destructor.** *Curr Biol* 2015, **25:**613-620.

37. Wanner KW, Robertson HM: **The gustatory receptor family in the silkworm moth Bombyx mori is characterized by a large expansion of a single lineage of putative bitter receptors.** *Insect Mol Biol* 2008, **17:**621-629.

38. Richards S, Gibbs RA, Weinstock GM, Brown SJ, Denell R, Beeman RW, Gibbs R, Bucher G, Friedrich M, Grimmelikhuijzen CJ, et al: **The genome of the model beetle and pest Tribolium castaneum.** *Nature* 2008, **452:**949-955.

39. Thorne N, Amrein H: **Atypical expression of Drosophila gustatory receptor genes in sensory and central neurons.** *J Comp Neurol* 2008, **506:**548-568.

40. Xiang Y, Yuan Q, Vogt N, Looger LL, Jan LY, Jan YN: **Light-avoidance-mediating photoreceptors tile the Drosophila larval body wall.** *Nature* 2010, **468:**921-926.

41. Ni L, Bronk P, Chang EC, Lowell AM, Flam JO, Panzano VC, Theobald DL, Griffith LC, Garrity PA: **A gustatory receptor paralogue controls rapid warmth avoidance in Drosophila.** *Nature* 2013, **500:**580-584.

42. Lee Y, Moon SJ, Montell C: **Multiple gustatory receptors required for the caffeine response in Drosophila.** *Proc Natl Acad Sci U S A* 2009, **106:**4495-4500.

43. Miyamoto T, Amrein H: **Suppression of male courtship by a Drosophila pheromone receptor.** *Nat Neurosci* 2008, **11:**874-876.

44. Bray S, Amrein H: **A putative Drosophila pheromone receptor expressed in male-specific taste neurons is required for efficient courtship.** *Neuron* 2003, **39:**1019-1029.

45. Benton R, Vannice KS, Gomez-Diaz C, Vosshall LB: **Variant ionotropic glutamate receptors as chemosensory receptors in Drosophila.** *Cell* 2009, **136:**149-162.

46. Croset V, Rytz R, Cummins SF, Budd A, Brawand D, Kaessmann H, Gibson TJ, Benton R: **Ancient protostome origin of chemosensory ionotropic glutamate receptors and the evolution of insect taste and olfaction.** *PLoS Genet* 2010, **6:**e1001064.

47. Stewart S, Koh TW, Ghosh AC, Carlson JR: **Candidate ionotropic taste receptors in the Drosophila larva.** *Proc Natl Acad Sci U S A* 2015, **112:**4195-4201.

48. Abuin L, Bargeton B, Ulbrich MH, Isacoff EY, Kellenberger S, Benton R: **Functional architecture of olfactory ionotropic glutamate receptors.** *Neuron* 2011, **69:**44-60.

49. Terrapon N, Li C, Robertson HM, Ji L, Meng X, Booth W, Chen Z, Childers CP, Glastad KM, Gokhale K, et al: **Molecular traces of alternative social organization in a termite genome.** *Nat Commun* 2014, **5:**3636.

50. Silbering AF, Rytz R, Grosjean Y, Abuin L, Ramdya P, Jefferis GS, Benton R: **Complementary function and integrated wiring of the evolutionarily distinct Drosophila olfactory subsystems.** *J Neurosci* 2011, **31:**13357-13375.
